# Supplementary material for: Impacts of abiotic factors and pesticide on the development, phenology, and reproductive biology of pink bollworm, Pectinophora gossypiella (Saunders) (Lepidoptera: Gelechiidae)
Source: PeerJ. 2024 Nov 29;12:e18399. doi: 10.7717/peerj.18399 (PMC11610462; doi:10.7717/peerj.18399)
Supplement: Supplemental Information 1 [file peerj-12-18399-s001.docx]

Basic Information of the project:

| Name of the project | A comprehensive integrated scientific approach for the development of sustainable management strategies of Pink Bollworm (*Pectinophora gossypiella)* | |
| --- | --- | --- |
| Project period ( from-to) | 01-01-18 | 31-12-20 |
| Total project duration | 36 Months (3 years) |  |
| Total Project cost | Rs. 12.54605 Millions | |
| Total Expenditures |  | |
| Name of the Project  Manager with designation  Phone and Email | Dr. Muhammad Jalal Arif  Professor  Cell #: 0322 7641232 Email: jalalarif807@yahoo.com | |
|  |  |  |
|  |  |  |
| Host Institute  Name and Designation of  the Team Leader with  Name of the Collaborating  Institute | Department of Entomology, University of Agriculture, Faisalabad | |
|  | Dr. Muhammad Jalal Arif  Professor  Cell #: 0322 7641232 Email: [jalalarif807@yahoo.com](mailto:jalalarif807@yahoo.com) | |
|  |  |  |
|  |  |  |
|  |  |  |
|  |  |  |
|  |  |  |
| Overseas cooperating  scientist and organization |  | |
|  |  |  |

Executive Summary

The UAF component of this project consisted of eight objectives-1) Survey, collection, identification and population dynamics of PBW from different cotton growing districts; 2) Mass-rearing of PBW; 3) Determination of alternate host plants of PBW; 4) To study diapausing behaviour of Pink Bollworm; 5) BT gene efficacy evaluation through chemicals/ phytohormones application; 6) Monitoring of insecticide-efficacy and insecticide-resistance development through conventional bioassays for insecticides used on cotton crop for Pink bollworm; 7) Assessment of enzyme activity in resistant strains of PBW; 8) Evaluate the role of insecticides mixture and synergists in resistance management. Various activities under these objectives were completed and salient achievements/results have been described here.

The results of various activites of objective-1 depict that no PBW adult captures was observed from January to 1^st^ week of April, however, PBW adult captures fluctuated from few to many male-moths/trap/fortnight (May to October) with peak captures during September. The infestation data indicate that infestation of PBW on cotton started from mid of July with peak infestation in the month of September in treated block. However, mean infestation of 2.5-50% was recorded in control block (field) during July-September in selected districts. From January-September, the adult captured in pheromone traps and reared from infested bolls/flowers were collected, preserved in 70% alcohol and brought into laboratory for morphological identification and molecular confirmation. The larvae and pupae of the collected population from different districts were preserved in 70% alcohol and brought into laboratory for morphological identification and molecular confirmation. The result of morphological identification and PCR sequencing and phylogenetic analysis indicated that the individuals (adults, larvae and pupae) of PBW samples collected from all selected districts were of same species of *P. gossypiella* as samples had 99-100% identity with NCBI submitted specimens of *P. gossypiella* reported from other countries.

Under the objective-1, different development and research activities were carried out. Under development activities of this objective, five team scientists (Dr. Waqas Wakil, Dr. M. Dildar Gogi, Dr. M. Arshad, Dr. Abid Ali and Dr. Zain ul Abdin,) got **training on pink bollworm rearing** in **International workshop on “lab establishment and rearing techniques of Pink Bollworm”** organized by Department of Entomology, MNSUAM in collaboration with Department of Entomology, UAF, Hubei Academy of Agricultural Sciences (HAAS), China. In this workshop, the Chinese scientists/experts, Dr. Wang Ling and Dr. Shengbao, trained the participants on PBW Lab development and its rearing on artificial diet. Department of Entomology, UAF organized a **One Day Seminar/training-workshop on “Pink Bollworm”** May 25, 2018 at UAF during the visit of Chinese scientists, Dr. Wang Ling and Dr. Shengbao to Department of Entomology UAF. In this event, the delegations and participants from AARI, NIBGE and MNSUAM. **MoU between UAF and Hubei Academy of Agricultural Sciences, Wuhan, China** has been drafted, approved from UAF Dean Committee and will be signed soon. Under this MoU, There will be exchange opportunity for faculty, students and technical staff. **A training program on PBW rearing**, handling, lab-management, bioassay-techiques and resistance monitoring techniques The team scientists (Prof. Dr. Jalal Arif, Dr. M. D. Dildar Gogi, Dr. M. Arshad and Dr. Abid Ali) have tentatively been finalized for training during April-May, 2019 under this MoU. A **PBW Rearing Laboratory** has been established in the Department of Entomology, UAF where PBW-populations from six different districts (Multan, Bahawalpur, Khaniwal, Sahiwal, Faisalabad and Vehari) are going to be managed on natural as well as artificial diet. The results of research activities indicate that among different larval diets (wheat-germ standard artificial diets, Okra (natural) diet, chickpea-artificial diet), Okra-diet was found the most appropriate diet for PBW rearing followed by chickpea-artificial diet and wheat-germ standard artificial diets. The effect of different adult-diets (honey, sucrose and glucose at 5%, 10% and 15% concentration each) indicate that the highest longevity of *P. gossypiella* was recorded on the 10% honey solution (12.8 days) followed by sucrose solution (10.83 days) and glucose solution (9.88 days). The maximum eggs / life span of *P. gossypiella* was recorded on the 10% honey solution (44.7 eggs/ life span of P. gossypiella) followed 10% sucrose solution (38.3 eggs) and 10% glucose solution (31.00 eggs). Overall, 10% honey solution is an ideal diet for adult PBW (more survival and fecundity). The results of rough texture tissue papers of different colors (Green, Red, White) as egg receptacles reveal that artificial oviposition substrate of white colour showed the highest oviposition (33.67 eggs/female) followed by green color (29.67 eggs/female) and red color (27.01 eggs/female). The results of white colored artificial substrates (like Nappy liner, Gouache paper and “Vinda Kitchen Towel super absorbent) of the same colour but of different texture with and without cotton leaves and its extracts as egg receptacles indicate that artificial oviposition substrate of kitchen towel showed the highest oviposition (32.67 eggs/female) followed by nappy liner (31.01 eggs/female) and gouache paper (29.02 eggs/female).

Under objective-3, Four (4) alternate host plants (okra, tomato, gulekhaira, lucern) and Cotton (bt and non-bt as host plant) were investigated. The results depicted that no PBW adults and larvae were observed or recorded. Cotton crop found as only host plant of PBW in Punjab, Pakistan.

The results of objective-4 reveal that diapausing in PBW was stimulated by decrease in temperature (<20 °C) and photoperiod (more dark period and less light period). Last larval instar of September-October population exhibited diapause more in double seed (approx. 70%) and less in single seed (approx. 30%) during November-February. Diapausing behavior of the 4th instar larvae of pink bollworm was found inconsistent between its diapausing months. The cage study indicate that all the 4^th^ instar larvae (100%) underwent pupation and no larvae were found in diapausing condition during the months of August and September. In October, less than 50% of the total larvae experienced diapause while 100% of the 4^th^ instar larvae demonstrated diapause in the months of November, December and January; while no larvae exhibited pupation neither in soil nor in bolls (single or double seed). During the month of October, about 20%, 16-18% and 8-10% of the diapausing larvae were found in diapause in double-seed, single-seed and soil, respectively. In the month of November, about 73%, 13% and 14% of the diapausing larvae experienced diapause in double-seed, single-seed and soil, respectively. In the month of December 61-64, 30-32% and 5-6% of the diapausing larvae were found in diapause in double-seed, single-seed and soil, respectively (Figure 25). During the month of January, more 4^th^ instar larvae were found in diapause in single seed (54-63%) as compared to those diapausing in double seed (37-46%). In the months of August and September, all the 4^th^ instar larvae underwent pupation out of which more than 90% were found pupated in soil and 5-7% were found pupated in bolls. The percent larvae pupated in bolls were found only in double seed (100%) and none of the larvae were found pupated in single seed (0%) inside the bolls. In the month of October, 51-54% larvae were found in pupation while 45-48% larvae were found in diapause. Out of pupating larvae, about 80% and 20% larvae pupated in soil and bolls, respectively. Out of the total larvae pupated in bolls, 88-90% and 10-12% were found pupated in double and single seed, respectively. Diets having less proportion of protein, carbohydrate, oil and vitamin than standard-diet exhibited less diapausing behavior of PBW. Among these diapausing variants, oil-content proved more important. The larvae fed on diets with least amount of oil exhibited least diapsuse (app. 50 less than standard-diet). The rest 50 % diapausing larvae fed at diets with least diapausing-variant-contents exhibited short diapause. The larvae fed on standard diet exhibited long diapause.

The results of objective-5 indicate that Jasmonic acid @ 0.3 mM found better plant activator that enhanced Bt gene expression and more mortality of PBW larvae

The results of objective-6 demonstrate that overall, Betacyfluthrin + Triazophos 41.7 EC, Deltamethrin + Triazophos 36 EC and Profenophos+Lamdacyhalothrin 61.5 EC proved highly effective followed by Triazophos and Alphacypermethrin against adults and larvae of PBW under laboratory conditions. The results of field study demonstrate that Betacyfluthrin + Triazophos 41.7EC, Deltamethrin + Triazophos 36EC and Profenophos+Lamdacyhalothrin 61.5 EC proved highly effective followed by Gamcyhalothrin 60 CS, Emmamectinbenzoate 5 EC, Alphacypermethrin 10 EC, Quinalphos and Triazophos 40 EC, 25 EC against adults and larvae of PBW. Resistance level of PBW strains was Multan-Strain > Bahawalpur-Strain > Vehari-Strain > Khaniwal-Strain > Sahiwal-Strain > Faisalabad-Strain. The larvae fed on diets admixed with Vitamin-B, Vitamin-C and Vitamin-AD exhibited approximately 2.5 time less mortality than the larvae fed on diet without these vitamins against cypermethrin and triazophos treatment. Among vitamins, vitamin-B and vitamin-C proved more important as larvae fed on diets admixed with these two vitamins exhibited least (statistically similar) mortality against cypermethrin and triazophos treatment.

The results of objective-6 demonstrate that as compared to susceptible lab strain, fourth instar larvae of FSD-strain, SWL-strain, and KWL-strain showed significantly lower specific activity of Mixed Function Oxidases (MFOs) enzyme against Triazophos and Cypermethrin treatment. However, an increased activity of MFOs was observed in fourth instar larvae of VH-strain, BWP-strain, and MLN-strain against Triazophos and Cypermethrin treatment as compared to susceptible lab strain. Unlikely, fourth instar larvae of FSD-strain, SWL-strain, KWL-strain, VH-strain, BWP-strain, and MLN-strain showed significantly lower specific activity of MFOs against treatment with two insecticide-mixtures (COMBO® and FORTRESS®) as compared to susceptible lab strain. These results indicate that insecticide mixture reduced specific activity of MFOs and enhanced susceptibility of 4^th^ instar larvae of PBW.

The results of objective-6 demonstrate that COMBO® 61.5% EC (Profenophos 60% and Lamda cyhalothrin 1.5% and FORTRESS® 36% EC (Deltamethrin 1% and Triazophos 35% proved effective mixture against PBW strains which exhibited less level of MFOs detoxifying enzymes, less LC_50_ values and more susceptibility when treated with these mixtures. PBO (Piperonyl butoxide), when admixed with deltamethri, bifenthrin, cypermethrin and lamda-cyhalothrin, demonstrated many fold reduction in the LC_50_ and LC_90_ values of these insecticides when applied alone. PBO proved effective in management of resistance in PBW against insecticides.

PROGRESS OF RESEARCH WORK

1. **Introduction:**

Cotton is an important cash crop providing raw material for textile industry. It’s share is 1.0 percent in country’s GDP with 5.1 percent share in agriculture value addition. During fiscal year 2015-16, production of cotton declined 27.8 percent as compared to 2014-15 i.e. (10.074 million vs 13.960 million bales). Pink bollworm, *Pectinophora gossypiella* (Saunders) infestation was identified at top among other possible causes of decline in cotton yield (Economic Survey of Pakistan 2015-16, pages 24-26). Pink bollworm is the most destructive insect pest of cotton, caterpillars feed on the fruiting parts of the plant (flowers, squares and bolls) hence responsible for direct yield loss. It is found in almost all cotton growing areas of the world. Our farmers are relying on the studies conducted at other countries while situations differ from area to area. There is dire need to investigate the possibility of resistance development in PBW in Pakistan.

Pink bollworm (*Pectinophora gossypiella*) is the most damaging pest throughout the world and has become real threat to conventional and Bt cotton varieties in Pakistan. It is difficult to control this pest because of its cryptic feeding behaviour. This pest completes its four generations on cotton crop and the larvae of fifth generation live in the leftover bolls and seed cotton in ginning factories after final picking in diapausing stage (Ahmed, 2013). Mostly farmers relied on chemicals to manage this pest; but due to indiscriminate use of synthetic pesticides resistance have been developed in pest against all group of insecticides (Ahmad et al., 1995). Transgenic cotton technology considered to be very favourable and offered a high level of resistance against the bollworms including *Helicoverpa armigera* (Hubner), *Earias vittella* (Fabricius) and *P. gossypiella* both in laboratory as well as field conditions (Ghosh, 2002; Kranthi, 2002; Kranthi and Kranthi, 2004). Most of the field studies have shown that bollworm larvae consistently survive and damage the transgenic cotton and the farmers have to apply the chemicals to control the cotton bollworms (Wu et al. 2002; Burd et al. 2003; Jakson et al. 2003; Tabashnik et al. 2003). However, loss of target pest susceptibility as a result of resistance was foreseen to be the greatest biological problem of transgenic crops (Mellon and Rissler, 1998). This might be due to the problem of number of parallel *Pectinophora gossypiella* generations exposed to the similar toxins; the mortality level depends on the degree of resistance or susceptibility of pest or the number of susceptible moths available for mating with moths carrying the resistance genes.

There is not a single control tactic which claimed to suppress PBW population despite IPM strategies. Integrated pest management is recommended for PBW. Some advance techniques of PBW control are being employed along with conventional methods of pest control in different parts of the world. These include: (1) Use of sex pheromones, Gossyplure-baited traps for prediction of moth emergence in early season detections and study of population trends of pink bollworm throughout the season. PB ropes are used in mating disruption of moths employed the male annihilation and male confusion techniques. (2) Use of transgenic cultivars of cotton like Bt cotton expressing Cry1Ac and Cry2Ab genes (3) Cultural practices like destruction of cotton sticks and remnants of plant parts by the use of different machines (5) Use of bio-control agents have also been reported to some parts of the world (6) Use of insecticide sprays for chemical control of pink bollworm.

Similarly, due to insect’s resistance against chemicals prompt the use of sex pheromones to manage pink bollworm has become dire need in the cotton growing areas worldwide. In several studies, mating disruption and mass trapping was considered effective against the target pests, e.g., pink bollworm, *Pectinophora gossypiella* (Saunders) (Ahmad and Attique, 1993). The estimated annual production of lures for monitoring and mass trapping is on the order of tens of millions, covering millions of hectares worldwide (Witzgall et al. 2010). Once identified and synthesized, sex pheromones can be used to bait traps that provide simple, specific tools for monitoring and to control insect pests. Unlike conventional pesticides, pheromones, do not damage other animals, nor do they pose health risks to people. They also can be used to lure the pests into traps that help farmers track insect population growth and stages of development. A number of studies showed variation in efficacy of Bt toxin against the pink bollworm in different cotton growing areas of the world. The frequency of pink bollworm resistance to Cry1Ac, though surprisingly high in 1997 collections, declined from 1998 to 2004 (Tabashnik et al. 2000, Tabashnik et al. 2005). So in the present scenario of Bt cotton cultivation at large scale, resistance monitoring for Bt toxin in *P*. *gossypiella* is essential. So in the present study baseline susceptibility of pink bollworm population against Bt cotton will be assessed from different cotton growing areas of Punjab Pakistan. The use of eco- friendly management methods are suitable for control of this pest by reducing the number of toxic pesticides applications (Khuhro, 2014). The study will provide the back ground for developing high level resistance of transgenic Bt cotton varieties against *P*. *gossypiella*. Additionally, screening and identification of efficient sex pheromones will be assessed by monitoring, mass trapping and mating disruption of pink bollworm population.

Bollworms if neglected can cause heavy damage to cotton resulting in 20-30% of yield reduction (Ahmad, 1980; Ali et al., 2016), however, these; losses can be significantly reduced with the adoption of Bt cotton. Benefits of Bt crops expressing Cry toxins are enormous, however, without adopting appropriate resistance management strategies, life expectancy of Bt crops may be short. Resistance development in target insect pests against Bt toxins is considered the major threat to long-term efficacy of Bt crops (Soberon et al., 2007; Fabrick et al., 2014 & 2015).

Transgenic crops containing *Bacillus thuringenesis* (Bt) toxin Cry1Ac kill insect pests especially bollworms of cotton, commercialized in 1996. It had been reported that transgenic crops were grown on more than 58 million hectares in 2010, which increased to 76 million hectares till 2013, all over the world (Wei, et al. 2015, James et al. 2013). Bt cotton was introduced in Pakistan in 2005 and cultivated on more than 95% area in 2015. Evolution of resistance against Bt toxins is a major threat to long term efficacy of toxins all over the world (Tabashnik, 1994; Gould, 1998; Tabashnik et al. 2009 and Carrie`re, 2010). Almost eight species of insect pests infesting different transgenic crops have been reported to develop resistance against Bt toxins. (Tabashnik et al. 1990; Janmaat and Myers, 2003; Tabashnik et al. 2008; Kruger et al. 2009; Storer et al. 2010; Dhurua and Gujar, 2011; Gassmann et al. 2011; Zhang et al. 2011). Different mechanisms of resistance had been identified from different regions and area wide management tactics had been recommended in different parts of the world. In Pakistan, Bt cotton with Cry1Ac had been introduced since 2005 and it was very effective in controlling bollworms till 2014.

Resistance to Bt toxins have been reported in *H. armigera*, in a laboratory-selected strain and in field-selected populations from northern China that were exposed intensively to Bt cotton expressing Cry1Ac (Yang et al. 2007). Field evolved pink bollworm resistance to Bt cotton has recently been reported in India and China (Dhurua and Gujar, 2011; Wan et al., 2012; Fabrick et al. 2014 & 2015). During 2010, Bt cotton was formally approved for commercial cultivation in Pakistan (Robert et al. 2012), which has provided better protection against bollworms. In Pakistan, most of the Bt cotton varieties approved for general cultivation have comparatively low expression level of Cry1Ac (Ullah et al., 2014) and even most of the farmers do not follow appropriate refuge plans. Widespread adoption of Bt crops without proper refuges can impose high selection pressure for Bt toxin resistance in insect pest populations (Tabashnik et al., 1994; Gujar et al., 2007). Despite of Bt cotton cultivation, pink bollworm has emerged as one of the major pest of cotton in Pakistan. Due to various reasons, cotton production fell from 14 to 9 million bales during recent cotton season and pink bollworm has significant share to this damage of 5 million bales (Anonymous 2016a, b). Therefore, there is a growing concern that pink bollworm in Pakistan may also has developed resistance against Bt cotton. However, there is not a single study conducted for the detection of resistance against Bt in Pakistan so far whereas, it had been studied and reported in USA, India, China before taking any action. Replacement of Bollgard I ^(R)^ containing only Cry1Ac gene with Bollgard II^(R)^ containing two genes of Cry1Ac and Cry2Ab could be better option along with other management options as described by different scientists **(**Wei et al. 2015; Tabashnik, et al. 2008; 2009).

Interaction of Bt Cry toxins with insect midgut epithelial receptors is an important determinant of toxin specificity and insect resistance. According to pore-formation model, primary receptor (cadherin) binds and facilitates protease-activated Cry monomers to form an oligomeric Cry toxin. Then toxin oligomers bind to secondary receptors, including glycosylphosphatidylinositol (GPI)-anchored proteins, aminopeptidase N (APN) and alkaline phosphatases (FAL) and then insert into epithelial cells to create pores that cause osmotic shocks and cell death (Bravo et al. 2004; Soberon et al. 2009). Disruption of Bt toxin binding to midgut receptors is the most common mechanism of insect resistance. Mutations in cadherins that bind Cry1Ac, cause resistance in some lepidopteran (Soberon et al. 2007).

Cadherin promotes Bt toxicity by facilitating toxin oligomerization. Resistance to Cry1Ac is associated with reduced oligomer formation and insertion. It has been reported that modified Cry1A toxins lacking 56 amino acids at the amino-terminus including helix α-1 formed oligomers in vitro without cadherin and killed *P. gossypiella* larvae harboring cadherin mutations linked with >1000-fold resistance to native Cry1Ac. Native Cry1A toxins required cadherin to form oligomers, but modified Cry1A toxins lacking one α-helix did not. Therefore, it is suggested that engineering modified Bt toxins can be helpful to counter insect resistance and we can broaden options for pest control by using modified Cry1A along with native Cry1A and other toxins like Cry2 and Vip3 that have not been used as extensively as Cry1A (Soberon et al., 2007; Ocelotl et al., 2015).

According to classical mode of action models, Bt proteins are produced as inactive protoxins that require conversion to a smaller activated form to exert toxicity (de Maagd et al., 2001). Cry toxins have been improved through genetic engineering by using codons compatible to plants and removing the protoxin C-terminal region (Schuler et al., 1998; Mukhtar et al. 2006). However, as per recently proposed model of dual mode of action of Bt proteins, Cry1Ac protoxin is generally more potent than the corresponding activated toxin against resistant insects. Protoxins and activated toxins kill insects via different pathways (Tabashnik et al., 2015). This information is very critical and helpful to modify and engineer Bt genes that may help to enhance and sustain the efficacy of transgenic Bt crops.

RNAi mediated insect control by dsRNA expression in transgenic plants is a viable method, because plants encode gene silencing pathways that lead to RNAi, and have the natural ability to silence genes originating in organisms from multiple kingdoms^16^. Several reports have demonstrated transgenic plant-mediated pest control by expression of dsRNA homologous to important insect genes essential for livelihood (Huvenne, H. & Smagghe, G. 2010). Transgenic *Arabidopsis thaliana* targeting the *Rack1* gene- and transgenic *Nicotiana benthamiana* targeting the *MPC002* gene of *Myzus persicae* have been reported to affect the insect life span (Pinto et al. 2011). Silencing of the *CYP6AE14c* gene impaired *Helicoverpa armigera* larval resistance to gossypol in *Gossypium hirsutum* (Mao et al. 2007)*.* The Western corn rootworm was controlled using transgenic *Zea mays* producing dsRNA against a *V-ATPase-A* gene (Bolognesi et al., 2012).

Development of resilient cotton varieties is one of the most economical and user-friendly method for combating biotic and abiotic stresses. Using conventional breeding assays, progress towards the development of breeding improved cotton cultivars was hampered by the complexities and interactions of environment with genotype—making the breeding prediction unreliable, and also by the non-availability of desirable traits in the cotton germplasm. Recent introduction of DNA markers in selecting the correct genotypes has really made it possible to accelerate the breeding progress by design—thus can overcome the environmental interactions. Marker-assisted backcrossing has been deployed in introgressing Ascochyta Blight resistance with double-podding traits in chickpea (Varshney *et al*., 2013), submergence tolerance in rice (Neeraja *et al*., 2007) and rust resistance in wheat (Mallick *et al.,* 2014). In cotton resistance to verticillium wilt (caused by the pathogen *Verticillium dahliae*) is introgressed into *G. hirsutum* L. using interspecific chromosome segment introgression lines (CSILs) from *G. barbadense* Wang *et al.,* 2014). Moreover, two RIL populations and two corresponding backcross populations were applied to elucidate the genetic basis of oil content, seed index and yield heterosis in Upland cotton Shang *et al*. 2016a, 2016 b). Furthermore, fiber traits were improved by introgressing QTLs linked to improved fiber from *G. barbadense* L. into *G. hirsutum* L. (Cao *et al*., 2013) through MAS. Comparatively fewer approach marker assisted recurrent selection (MARS) can be a strategy for transferring QTLs or several epistatic QTLs that control complex traits. Marker-assisted background selection can yield rapid recovery of recurrent parent genome in a short span of 2–3 backcross generations (Varshney *et al.,* 2011).

1. Project Objective:

Outputs planned for the project:

| **OBJECTIVE OF THE** | |
| --- | --- |
| **Component-1**  University of Agriculture, Faisalabad | - - Survey and collection of PBW from different cotton growing areas   - Morphological and DNA-barcoding based identification and characterization of field collected PBW population   - Rearing of PBW from field collection population.   - Determination of on- and off-season prevalence of PBW on alternative host plants   - Determine diapausing behaviour of PBW   - Application of chemicals/ phytohormones on Bt cotton   - Monitoring of insecticide field efficacy against PBW   - Assessment of enzyme activity in resistant strains of PBW   - Evaluate the contact toxicity of insecticides mixtures in field against PBW   - Farmer training workshops / seminars / public awareness campaigns |
| **Component-2**  Director Entomological Research Institute, AARI, Faisalabad | - Rearing technology of pink bollworm - Efficacy of different insecticides against pink bollworm in field and lab condition. - Impact of pesticides on the crop physiology/shape/canopy. - Behavior of pink bollworm against different varieties in green house and fields. - Optimum timing and stage of spray against PBW |
| **Component-3**  Cotton Research Institute, Multan | - Rearing technology of pink bollworm - Efficacy of different insecticides against pink bollworm in field and lab condition. - Impact of pesticides on the crop physiology/shape/canopy. - Behavior of pink bollworm against different varieties in green house and fields. - Optimum Bt toxin required for PBW control in existing cotton varieties. - Optimum timing and stage of spray against PBW. - Topping fifty days before last picking and its impact on PBW infestation. |
| **Component-4**  MNSUA, Multan | - Efficacy of different insecticides against PBW in field and lab conditions. - Bt Resistance Monitoring - Identification /import and rearing of PBW predators and parasites - Study behavior on BT and non BT paired plots |
| **Component-5**  National Institute for Biotechnology and Genetic Engineering (NIBGE) Faisalabad | - Characterization of pink bollworm and development of transgenic cotton germplasm against pink bollworm |
| **Component-6**  Central Cotton Research Institute, Multan | - Rearing technology - Diapausing and cyclic behavior of PBW - Efficacy of different insecticides against PBW in field and lab conditions. - Identification /import and rearing of PBW predators and parasites. - .Impact of pesticides on the crop physiology/shape/canopy - Optimum BT toxin required for PBW control in existing cotton varieties. - Optimum timing and stage of spray against PBW. - Topping fifty days before last picking and its impact on PBW infestation - Bt Resistance Monitoring - Study behavior on BT and non BT paired plots - Modelling of PBW epidemiology dynamics. - Onset of PBW attack - Weather variables and relationship of PBW |

1. Detailed component wise methodology adopted, data analyzed and results obtained (Attach raw data as annexure) Scientific/technical methodology (give details):

| **Objective-1:** Survey, collection, identification and population dynamics of PBW from different cotton growing districts | | | |
| --- | --- | --- | --- |
| Item | Description | 3 Years Targets | 3 Years Achievements |
| Out Put -1 | Confirmation of the species diversity and population dynamics of PBW in cotton growing areas | In total 48 samples from four districts (Faisalabad, Sahiwal, Multan, Bahawalpur) on fortnightly basis for each of two years (2018 and 2019) and morphological as well as molecular identification of pink bollworm specimens on larval, pupal and adult basis |  |
| Activity-1 | Survey and collection of PBW from different cotton growing areas | In total 48 samples from four districts (Faisalabad, Sahiwal, Multan, Bahawalpur) on fortnightly basis for each of two years (2018 and 2019) and morphological as well as molecular identification of pink bollworm specimens on larval, pupal and adult basis | - no PBW adult captures was observed from January to 1^st^ week of April - However, PBW adult captures fluctuated from few to many male-moths/trap/fortnight (May to October) with peak captures during September. - The infestation data indicate that infestation of PBW on cotton started from mid of July with peak infestation in the month of September in treated block. - However, mean infestation of 2.5-50% was recorded in control block (field) during July-September in selected districts. |
| Activity-2 | Morphological and DNA-barcoding based identification and characterization of field collected PBW population | Morphological as well as molecular identification of pink bollworm specimens on larval, pupal and adult basis of the 48 samples collected from four districts (Faisalabad, Sahiwal, Multan, Bahawalpur) | - From January-September, the adult captured in pheromone traps and reared from infested bolls/flowers were collected, preserved in 70% alcohol and brought into laboratory for morphological identification and molecular confirmation. - The larvae and pupae of the collected population from different districts were preserved in 70% alcohol and brought into laboratory for morphological identification and molecular confirmation. - The result of morphological identification and PCR sequencing and phylogenetic analysis indicated that the individuals (adults, larvae and pupae) of PBW samples collected from all selected districts were of same species of *P. gossypiella* as samples had 99-100% identity with NCBI submitted specimens of *P. gossypiella* reported from other countries. |
| **Objective-2:** Mass-rearing of PBW | | | |
| Out Put -2 | PBW mass culturing in the laboratory will be established |  |  |
| Activity-1 | Field collection of all the pest stages and rearing on the natural diet. |  | - To achieve this objective following activities were carried out and accomplished: - Five team scientists (Dr. Waqas Wakil, Dr. M. Dildar Gogi, Dr. M. Arshad, Dr. Abid Ali and Dr. Zain ul Abdin,) got **training on pink bollworm rearing** in **International workshop on “lab establishment and rearing techniques of Pink Bollworm”** organized by Department of Entomology, MNSUAM in collaboration with Department of Entomology, UAF, Hubei Academy of Agricultural Sciences (HAAS), China. In this workshop, the Chinese scientists/experts, Dr. Wang Ling and Dr. Shengbao, trained the participants on PBW Lab development and its rearing on artificial diet. - Department of Entomology, UAF organized a **One Day Seminar/training-workshop on “Pink Bollworm”** May 25, 2018 at UAF during the visit of Chinese scientists, Dr. Wang Ling and Dr. Shengbao to Department of Entomology UAF. In this event, the delegations and participants from AARI, NIBGE and MNSUAM. - **MoU between UAF and Hubei Academy of Agricultural Sciences, Wuhan, China** has been drafted, approved from UAF Dean Committee and will be signed soon. Under this MoU, There will be exchange opportunity for faculty, students and technical staff. - **A training program on PBW rearing**, handling, lab-management, bioassay-techiques and resistance monitoring techniques The team scientists (Prof. Dr. Jalal Arif, Dr. M. D. Dildar Gogi, Dr. M. Arshad and Dr. Abid Ali) have tentatively been finalized for training during April-May, 2019 under this MoU. - A **PBW Rearing Laboratory** has been established in the Department of Entomology, UAF where PBW-populations from six different districts (Multan, Bahawalpur, Khanewal, Sahiwal, Faisalabad and Vehari) are going to be managed on natural as well as artificial diet. |
| Activity-2 | Laboratory rearing of the PBW larvae on the artificial diets and culturing for continuous insect populations |  | - During 2019, Larval rearing on different larval diets was assessed. PBW was reared on wheat-germ standard artificial diets, Okra (natural) diet, chickpea-artificial diet. Okra-diet was found the most appropriate diet for PBW rearing followed by chickpea-artificial diet and wheat-germ standard artificial diets. - During 2019, effect of different adult-diets (honey, sucrose and glucose at 5, 10 and 15% concentration each) was assessed. These diets were given in form of solution. The cotton was dipped in the solution and placed in the center of chimney for the feeding of adults. The diet was changed after every 2 days. Survival rate of adults male and female were observed. The highest longevity of P. gossypiella was recorded on the 10% honey solution (12.8 days) followed by sucrose solution (10.83 days) and glucose solution (9.88 days). The maximum eggs / life span of *P. gossypiella* was recorded on the 10% honey solution (44.7 eggs/ life span of *P. gossypiella*) followed 10% sucrose solution (38.3 eggs) and 10% glucose solution (31.00 eggs). - 10% honey solution is an ideal diet for adult PBW (more survival and fecundity) - Rough texture tissue papers of different colors (Green, Red, White) were assessed as egg receptacles. Artificial oviposition substrate of white colour showed the highest oviposition (33.67 eggs/female) followed by green color (29.67 eggs/female) and red color (27.01 eggs/female). - White colored artificial substrates like Nappy liner, Gouache paper and “Vinda Kitchen Towel super absorbent of the same colour but of different texture with and without cotton leaves and its extracts were used as egg receptacles. Artificial oviposition substrate of kitchen towel showed the highest oviposition (32.67 eggs/female) followed by nappy liner (31.01 eggs/female) and gouache paper (29.02 eggs/female) |
| **Objective-3:** Determination of alternate host plants of PBW | | | |
| Out Put-3 | Off- and on-season prevalence of PBW on alternative host plants |  |  |
| Activity-1 | Determination of on- and off-season prevalence of PBW on alternative host plants |  | - Four (4) alternate host plants (okra, tomato, gulekhaira, lucern) are part of this activity. Okra, Cotton (bt and non-bt), tomato, gulekhaira and lucern while tomato, gulekhaira and lucern [planted as per their sowing times (Mid October)] were investigated. The results depicted that no PBW adults and larvae were observed or recorded. - Cotton crop is the only host plant of PBW in Punjab, Pakistan. |
| **Objective-4:** To study diapausing behaviour of Pink Bollworm | | | |
| Out Put-4 | Year round biology and diapausing behaviour of the pest will be studied |  |  |
| Activity-1 | Different life stages of the PBW will be studied under different temperature regimes in the laboratory |  | - Diapausing in PBW is stimulated by decrease in temperature (<20 °C) and photoperiod (more dark period and less light period). Last larval instar of September-October population exhibit diapause more in double seed (approx. 70%) and less in single seed (approx. 30%) during November-February. |
| Activity-2 | Different life stages of the PBW will be studied under natural conditions by performing caged studies |  | - Diapausing behavior of the 4^th^ instar larvae of pink bollworm was found inconsistent between its diapausing months. - All the 4^th^ instar larvae (100%) underwent pupation and no larvae were found in diapausing condition during the months of August and September. - In October, less than 50% of the total larvae experienced diapause while 100% of the 4^th^ instar larvae demonstrated diapause in the months of November, December and January; while no larvae exhibited pupation neither in soil nor in bolls (single or double seed). - During the month of October, about 20, 16-18 and 8-10% of the diapausing larvae were found in diapause in double-seed, single-seed and soil, respectively. - In the month of November, about 73, 13 and 14% of the diapausing larvae experienced diapause in double-seed, single-seed and soil, respectively. - In the month of December 61-64, 30-32 and 5-6% of the diapausing larvae were found in diapause in double-seed, single-seed and soil, respectively (Figure 25). - During the month of January, more 4^th^ instar larvae were found in diapause in single seed (54-63%) as compared to those diapausing in double seed (37-46%). - In the months of August and September, all the 4^th^ instar larvae underwent pupation out of which more than 90% were found pupated in soil and 5-7% were found pupated in bolls. The percent larvae pupated in bolls were found only in double seed (100%) and none of the larvae were found pupated in single seed (0%) inside the bolls. - In the month of October, 51-54% larvae were found in pupation while 45-48% larvae were found in diapause. Out of pupating larvae, about 80% and 20% larvae pupated in soil and bolls, respectively. Out of the total larvae pupated in bolls, 88-90% and 10-12% were found pupated in double and single seed, respectively. |
| Activity-3 | Effect of protein, carbohydrate, oil and vitamin variant diets on diapausing behavior of PBW under laboratory conditions. |  | - Diets having less proportion of protein, carbohydrate, oil and vitamin than standard-diet exhibited less diapausing behavior of PBW. Among these diapausing variants, oil-content proved more important. The larvae fed on diets with least amount of oil exhibited least diapsuse (app. 50 less than standard-diet). The rest 50 % diapausing larvae fed at diets with least diapausing-variant-contents exhibited short diapause. - The larvae fed on standard diet exhibited long diapause. |
| **Objective-5:** BT gene efficacy evaluation through chemicals/ phytohormones application | | | |
| Out Put -5 | Chemicals/phytohormones compounds enhancing Bt gene efficacy |  |  |
| Activity-1 | Application of chemicals/ phytohormones on Bt cotton against PBW |  | - Jasmonic acid @ 0.3 mM is better plant activator that enhances Bt gene expression and more mortality of PBW larvae |
| **Objective-6:** Monitoring of insecticide-efficacy and insecticide-resistance development through conventional bioassays for insecticides used on cotton crop for Pink bollworm | | | |
| Out Put-6 | - Effective Insecticides - The resistant PBW strains collected from different localities |  |  |
| Activity-1 | Monitoring of insecticide efficacy against PBW under laboratory and field conditions |  | Betacyfluthrin + Triazophos 41.7 EC, Deltamethrin + Triazophos 36 EC and Profenophos+Lamdacyhalothrin 61.5 EC proved highly effective followed by Triazophos and Alphacypermethrin against adults and larvae of PBW under laboratory and field conditions |
| Activity-2 | Monitoring of Insecticide resistant strains on the basis of LC_50_ values through conventional bioassay methods |  | Resistance level of PBW strains was Multan-Strain > Bahawalpur-Strain > Vehari-Strain > Khaniwal-Strain > Sahiwal-Strain > Faisalabad-Strain |
| Activity-3 | Effect of different vitamin admixed diets on resistance of pink bollworm larvae against field recommended doses of cypermethrin and triazophose |  | - The larvae fed on diets admixed with Vitamin-B, Vitamin-C and Vitamin-AD exhibited approximately 2.5 time less mortality than the larvae fed on diet without these vitamins against cypermethrin and triazophos treatment. - Among vitamins, vitamin-B and vitamin-C proved more important as larvae fed on diets admixed with these two vitamins exhibited least (statistically similar) mortality against cypermethrin and triazophos treatment. |
| **Objective-7:** Assessment of enzyme activity in resistant strains of PBW | | | |
| Out Put-7 | Report on the activity of different enzymes involved in insecticide resistance development in PBW |  |  |
| Activity-1 | Assessment of enzyme activity in resistant strains of PBW |  | - As compared to susceptible lab strain, fourth instar larvae of FSD-strain, SWL-strain, and KWL-strain showed significantly lower specific activity of Mixed function oxidases (MFOs) enzyme against Triazophos and Cypermethrin treatment. - However, an increased activity of MFOs was observed in fourth instar larvae of VH-strain, BWP-strain, and MLN-strain against Triazophos and Cypermethrin treatment as compared to susceptible lab strain. - Unlikely, fourth instar larvae of FSD-strain, SWL-strain, KWL-strain, VH-strain, BWP-strain, and MLN-strain showed significantly lower specific activity of MFOs against treatment with two insecticide-mixtures (COMBO® and FORTRESS®) as compared to susceptible lab strain. - These results indicate that insecticide mixture reduced specific activity of MFOs and enhanced susceptibility of 4^th^ instar larvae of PBW. |
| **Objective-8:** Evaluate the role of insecticides mixture and synergists | | | |
| Out Put-8 | List of efficient insecticides and their mixtures will be reported |  |  |
| Activity-1 | Evaluate the role of insecticides mixture/ synergists |  | COMBO AND FORTRESS proved effective mixture against PBW strains which exhibited less level of MFOs detoxifying enzymes, less LC_50_ values and more susceptibility when treated with these mixtures |
| **Objective-9:** Farmer training workshops/ seminars/ public awareness campaigns for PBW | | | |
| Out Put-9 | Technology transfer to stakeholders |  |  |
| Activity-1 | Farmer training workshops / seminars / public awareness campaigns |  |  |

**METHODOLOGY ADOPTED, DATA ANALYZED AND RESULTS OBTAINED (ATTACH RAW DATA AS ANNEXURE) SCIENTIFIC/TECHNICAL METHODOLOGY (GIVE DETAILS):**

**Objective-1:** Survey, collection, identification and population dynamics of PBW from different cotton growing districts

**Activity-1:** Survey and collection of PBW from different cotton growing areas (Faisalabad, Sahiwal, Multan, Bahawlpur)

**METHODOLOGY (YEAR 2018)**

Six different districts (Multan, Bahawalpur, Khanewal, Sahiwal, Faisalabad and Vehari) were selected for survey and collection of PBW as well as for the study PBW population dynamics. Fixed survey method was used. The data on PBW adults capture per trap was collected on fortnightly basis from 01-01-2018 to 31-12-2018. Similarly, PBW infestation data (percentage of boll and flower infestation) was also collected during flowering and boll formation stages of cotton from July 1, 2018 to 01-10-2018. In total 25 plants were selected randomly from an acre and total number of infested and uninfested flowers and bolls per plant were counted from each district on fortnightly basis. The collected data were then transformed into percentage infestation. The infested bolls/flowers were brought to laboratory and placed in adult rearing cages till the adult emergence. The larvae, pupae and emerging adults from these infested bolls were later on used for morphological identification and DNA barcoding studies.

For the collection of above mentioned adult-trapping and infestation data, following three sampling sites/units, farmers’ fields and ginning factories were selected in each district.

**1: Cotton field** (A cotton field of 1 acre maintained by the farmer at local recommended production and protection practices was selected where two PBW-pheromone traps were installed with the help of bamboo poles at the height of 1.8 m in the middle of the field)

**2: Cotton Stick-heaps** (Sticks-heaps were selected on farm house or in village where sticks were stored for fuel purpose, where one PBW-pheromone traps was installed with the help of bamboo poles at the height of 1.8 m near the stick-heaps)

**3: Cotton ginning factory** A Cotton ginning factory was selected in each district of respective districts where one PBW-pheromone trap was installed with the help of bamboo poles at the height of 1.8 m.

**COTTON FIELD AND GINNING FACTORY SURVEY LOCATIONS**

**Sahiwal**

1. Khan Faiz Khan, Chak No. 105/9L Raja Wala, Tehsil Cheechawatni, District Sahiwal
2. Rana M. Khan and Co cotton Ginning and Oil Mil.
3. Asif Patwari and Abdul Majeed, Chak No. 186/187-9L, Tehsil and District Sahiwal
4. Chamba Industries Cotton Harappa Station, Sahiwal

**Vehari:**

1. Aurangzaib gujar, Chak No. 128-WB Garha Mour, Multan Road Vehari
2. Bilal Cotton Industry, Multan Road Vehari

**Khanewal:**

1. Arayain Farm, Haji Muhammad Shfqat, Kot Islam, Tehsiel kabirwala, District Khanewal
2. Imran Ginning Factory, Bagur Pul, Tehsil kabirwala, District Khanewal

**Faisalabad**

1. Pind Dogran Chak No. 39-GB, Tehsil Gojra, district Faisalabad

**Multan**

1. Naveed Zia, 363-W/B, Dunyia Pur, Multan
2. Tayyaba Ginning Factory, Multan

**Bahawalpur**

1. Aslam Shahzad Ghouri, Sui Wala Village, Jalalpur Per Wala road, District Bahawalpur
2. Muzamil Cotton Factory, Lodhran Bahawalpur Road

The overall results of above survey reveal that PBW adult captures fluctuated between a range of 0.0 male-moths/trap/fortnight (January to April) to 5.0-75 male-moths/trap/fortnight (May to October) with peak captures of 75 male-moths/trap/fortnight during September. The infestation data indicate that infestation of PBW on cotton started from mid of July with infestation level of 2.5% and fluctuated between a ranges of about 2.5-10% with peak infestation of about 10% in September in treated block. However, mean infestation of 2.5-50% was recorded in control block (field) during July-September in selected districts.

**RESULTS**

**Vehari-1**

**Number of male adults captured per trap**

The number of male adults captured per trap tend vary throughout the sampling period among the cotton field, stick heaps and ginning factories.

**In the cotton field**, the adults appeared on 30^th^ April 2018 and the number of adults maximum (68 adults per trap) on 16^th^ July 2018. Then decrease in number was recorded and was highest (75 adults per trap) on 16^th^ September 2018. There was decline in the number of adults captured per trap after16^th^ September 2018 and again attained a peak (61 adults per trap) at 30^th^ October 2018 then it declined gradually up to 16 November 2018 and was no capture on 30^th^ November 2018.

**In the stick heaps**, adults were observed on 16^th^ March 2018 and keeps on fluctuating. The number of adults captured per trap was highest (31 adults per trap) twice the sampling period on 30^th^ June and 16^th^ August 2018 and declined on 16^th^ November 2018.

**In the ginning factories**, the number of adults captured per trap was least as compared to the number of adults captured per trap recorded in cotton field and stick heaps. The adults were observed on 16^th^ March 2018 and was maximum (23 adults per trap) on 16^th^ November 2018 while recorded no trapping on 16^th^ December 2018.

**Percent infested flowers**

The infestation of flowers was taken as percentage. Percent infestation was observed from 16^th^ June to 16^th^ November 2018. The percentage increased gradually and was maximum (29%) on 16^th^ November 2018.

**Percent unopened bolls infestation**

The infestation of unopened bolls was also taken in term of percentage. Percent unopened infested bolls were observed on 30^th^ July 2018 and infestation continue to increase and was at peak (15%) on 16^th^ October 2018 and again at peak (18%) on 16^th^ November 2018.

**Percent opened bolls infestation**

The infestation of opened bolls was also taken in term of percentage. Percent infested opened bolls were observed on 16^th^ August and it increased gradually and was at peak (37%) on 16^th^ November 2018.

**Khanewal**

**Number of male adults captured per trap**

The number of male adults captured per trap tend vary throughout the sampling period among the cotton field, stick heaps and ginning factories.

**In the cotton field**, the adults appeared on 5^th^ May 2018 and the number of adults increased gradually and highest (43 adults per trap) on 20^th^ July 2018 and form second peak (49 adults per trap) on 20^th^ August 2018 and was highest (69 adults per trap) on 20^th^ September 2018. The gradual decrease in number was recorded up to 5^th^ December 2018.

**In the stick heaps**, adults were observed on 5^th^ May 2018 and keeps on fluctuating. The number of adults captured per trap was at peak (33 adults per trap) on 20^th^ June 2018 and recorded highest (39 adults per trap) on 20^th^ July 2018 then declined gradually and again at peak on 20^th^ September 2018 and then declined.

**In the ginning factories**, the number of adults captured per trap was least as compared to the number of adults captured per trap recorded in cotton field and stick heaps. The adults were observed on 5^th^ May 2018 and peaked (5 adults per trap) on 5^th^ August 2018 and then the number decline gradually.

**Percent infested flowers**

The infestation of flowers was taken as percentage. Percent infestation was observed from 5^th^ June to 20^th^ November 2018. The percentage increased gradually and peaked (3.8%) on 5^th^ July 2018 and then declined with sharp increase and was at peak (7.8%) on 5^th^ August 2018. The percent infested flowers was highest (10%) on 5^th^ October 2018 and then declined gradually.

**Percent unopened bolls infestation**

The infestation of unopened bolls was also taken in term of percentage. Percent unopened infested bolls were observed on 5^th^ July 2018 and was at peak (8%) on 5^th^ August 2018, infestation continue to increase and was highest (22%) on 5^th^ October 2018 and then decline up to 16^th^ August 2018 and was again at peak (20%) on 5^th^ November 2018.

**Percent opened bolls infestation**

The infestation of opened bolls was also taken in term of percentage. Percent infested opened bolls were observed on 5^th^ August and there was sharp increase and was highest (73%) on 5^th^ September 2018 and then keep on fluctuating with peaks (57 and 53%) on 5^th^ October and 5^th^ November 2018 respectively.

**Bahawalpur**

**Number of male adults captured per trap**

The number of male adults captured per trap tend vary throughout the sampling period among the cotton field, stick heaps and ginning factories.

**In the cotton field**, the adults appeared on 7^th^ May 2018 and the number of adults increased gradually and maximum (41 adults per trap) on 7^th^ August 2018 and was highest (63 adults per trap) on 22^th^ September 2018. The gradual decrease in number was recorded up to 7^th^ November 2018.

**In the stick heaps**, adults were observed on 22^th^ March 2018 and keeps on fluctuating. The number of adults captured per trap was at peak (26 adults per trap) on 22^th^ May 2018 and recorded highest (33 adults per trap) on 22^th^ June 2018 then decreased and again at peak (29 adults per trap) on 22^th^ July 2018 and then declined gradually up to 22^th^ October 2018.

**In the ginning factories**, the number of adults captured per trap was least as compared to the number of adults captured per trap recorded in cotton field and stick heaps. The adults were observed on 3^rd^ March 2018 and increased gradually and was highest (10 adults per trap) on 7^th^ July 2018 and then the number declined gradually up to 7^th^ October 2018.

**Percent infested flowers**

The inf estation of flowers was taken as percentage. Percent infestation was observed from 7^th^ June to 7^th^ November 2018. The percentage increased gradually and peaked (9%) on 7^th^ July 2018 and then keep on fluctuating and was highest (11.5%) on 7^th^ September 2018 and again at peak (11%) on 7^th^ October 2018. A sharp decline was observed up to 7^th^ November 2018.

**Percent unopened bolls infestation**

The infestation of unopened bolls was also taken in term of percentage. Percent unopened infested bolls were observed on 7^th^ July 2018 and was at peak (5%) on 7^th^ August 2018, infestation continue to increase and was highest (6.5%) on 7^th^ September 2018 and then declined gradually and was at peak (3.9%) 7^th^ November 2018 and then declined.

**Percent opened bolls infestation**

The infestation of opened bolls was also taken in term of percentage. Percent infested opened bolls were observed on 7^th^ August 2018 and was highest (8%) on 7^th^ October 2018 and then declined up to 7^th^ November 2018.

**Multan**

**Number of male adults captured per trap**

The number of male adults captured per trap varied throughout the sampling period among the cotton field, stick heaps and ginning factories.

**In the cotton field**, the adults appeared on 2^nd^ May 2018 and the number of adults increased gradually and were maximum (32 adults per trap) on 17^th^ June 2018 and was highest (69 adults per trap) on 17^th^ July 2018. There was a sharp decline and again was at peak (55 and 65 adults per trap) on 2^nd^ September and 2^nd^ October 2018 respectively. Gradual decrease in number was recorded up to 2^nd^ December2018.

**In the stick heaps**, adults were observed on 17^th^ March 2018 and keeps on fluctuating. The number of adults captured per trap was at peak (18 adults per trap) on 2^nd^ June 2018 and recorded highest (47 adults per trap) on 17^th^ July 2018 then decreased and again at peak (31 adults per trap) on 17^th^ September 2018 and then declined gradually up to 2^nd^ December 2018.

**In the ginning factories**, the number of adults captured per trap was least as compared to the number of adults captured per trap recorded in cotton field and stick heaps. The adults were observed on 17^th^ March 2018 and was at peak (13 adults per trap) on 2^nd^ June 2018 and was highest (15 adults per trap) on 2 August 2018 and then the number decline gradually up to 2^nd^ December 2018.

**Percent infested flowers**

The infestation of flowers was taken as percentage. Percent infestation was observed from 17^th^ June to 17^th^ October 2018. The percentage increased gradually and was highest (9%) on 2^nd^ October 2018. A sharp decline was observed up to 17^th^ October 2018.

**Percent unopened bolls infestation**

The infestation of unopened bolls was also taken in term of percentage. Percent unopened infested bolls were observed on 2^nd^ July 2018 and was at peak (4.5%) on 17^th^ July 2018, infestation continue to fluctuate and was again at peak (4.9%) on 2^nd^ October 2018 and highest (5.7%) on 17^th^ October 2018 and then declined gradually.

**Percent opened bolls infestation**

The infestation of opened bolls was also taken in term of percentage. Percent infested opened bolls were observed on 17^th^ August 2018 and was highest (9%) on 17^th^ September 2018 and then declined and again at peak (8.2%) on 2^nd^ November 2018.

**Sahiwal**

**Number of male adults captured per trap**

The number of male adults captured per trap varied throughout the sampling period among the cotton field, stick heaps and ginning factories.

**In the cotton field**, the adults appeared on 15^th^ May 2018 and the number of adults increased gradually and peaked (39 adults per trap) on 30^th^ June 2018 and was highest (81 adults per trap) on 15^th^ July 2018. There was a gradual decline and again was at peak (51 adults per trap) on 15^th^ September 2018. Gradual decrease in number was recorded up to 15^th^ November 2018.

**In the stick heaps**, adults were observed on 15^th^ March 2018 and keeps on fluctuating. The number of adults captured per trap was at peak (19 adults per trap) on 15^th^ June 2018 and again at peak (30 adults per trap) on 30^th^ June 2018 and recorded highest (32 adults per trap) on 30^th^ August 2018 then decreased and again at peak (29 adults per trap) on 30^th^ September 2018 and then declined gradually up to 15^th^ November 2018.

**In the ginning factories**, the number of adults captured per trap was least as compared to the number of adults captured per trap recorded in cotton field and stick heaps. The adults were observed on 30^th^ March 2018 and was at peak (4 adults per trap) twice on 30^th^ May and 30^th^ June 2018 respectively and then the number decline gradually up to 15^th^ December 2018.

**Percent infested flowers**

The infestation of flowers was taken as percentage. Percent infestation was observed from 15^th^ June to 15^th^ October 2018. The percentage increased gradually and was at peak (10%) on 15^th^ July 2018 and was highest (18.5%) on 15^th^ September 2018. A sharp decline was observed up to 15^th^ November 2018.

**Percent unopened bolls infestation**

The infestation of unopened bolls was also taken in term of percentage. Percent unopened infested bolls were observed on 15^th^ August 2018 with a sharp increase recorded highest (12%) on 15^th^ September 2018 and then declined gradually up to 15^th^ November 2018.

**Percent opened bolls infestation**

The infestation of opened bolls was also taken in term of percentage. Percent infested opened bolls were observed on 30^th^ August 2018 and was at peak (6%) on 15^th^ September 2018 and was highest (7.5%) on 15^th^ November 2018.

**Faisalabad**

**Number of male adults captured per trap**

The number of male adults captured per trap varied throughout the sampling period among the cotton field, stick heaps and ginning factories.

**In the cotton field**, the adults appeared on 10^th^ May 2018 and the number of adults increased gradually and peaked (15 adults per trap) on 10^th^ June 2018 and then decreased. Its peak was again attained (28 adults per trap) again on 10^th^ August 2018 and was highest (50 adults per trap) on 10^th^ October 2018. Gradual decrease in number was recorded up to 10^th^ December 2018.

**In the stick heaps**, adults were observed on 10^th^ May 2018 and keeps on fluctuating. The number of adults captured per trap was highest (25 adults per trap) on 25^th^ May 2018 and then declined and again attained peak (21 adults per trap) on 25^th^ July 2018. The number of adults captured per trap then keep on fluctuating up to 10^th^ November 2018.

**In the ginning factories,** no record from Faisalabad district as no ginning factory.

**Percent infested flowers**

The infestation of flowers was taken as percentage. Percent infestation was observed from 10^th^ June to 10^th^ November 2018. The percentage increased gradually and was at peak (12.5%) on 1o^th^ July 2018 and was highest (19%) on 10^th^ October 2018.

**Percent unopened bolls infestation**

The infestation of unopened bolls was also taken in term of percentage. Percent unopened infested bolls were observed on 10^th^ July 2018 and keep fluctuating throughout the sampling period and was highest (26%) on 25^th^ October 2018 and then declined up to 10^th^ November 2018.

**Percent opened bolls infestation**

The infestation of opened bolls was also taken in term of percentage. Percent infested opened bolls were observed on 25^th^ August 2018 and was at peak (4%) on 25^th^ September 2018 and was highest (19.5%) on 25^th^ October 2018.

**FIGURES-1:** Number of male adults captured per trap, flower-infestation, opened-boll-infestation and unopened-boll-infestation at fortnightly interval at the chemically managed farmer field in Vehari, Sahiwal, Multan, Bahwalpur and Faisalabad for the year 2018

**Vehari-1**

**Vehari-2**

**Khanewal**

**Bahawalpur**

**Multan**

**Sahiwal**

**Faisalabad**

**METHODOLOGY (YEAR 2019)**

Six different districts (Multan, Bahawalpur, Khanewal, Sahiwal, Faisalabad and Vehari) were selected for survey and collection of PBW as well as for the study PBW population dynamics. Fixed survey method was used. The data on PBW adults capture per trap was collected on fortnightly basis from 01-01-2019 to 31-12-2019. Similarly, PBW infestation data (% boll infestation and % flower infestation) was also collected during flowering and boll formation stages of cotton from July 1, 2019 to 01-10-2019. Twenty five (25) plants were selected randomly from an acre and total number of infested and uninfested flowers and bolls per plant were counted from each district on fortnightly basis. The collected data were then transformed into percentage infestation. The infested bolls/flowers were brought to laboratory and placed in adult rearing cages till the adult emergence. The larvae, pupae and emerging adults from these infested bolls were later on used for morphological identification and DNA barcoding studies.

For the collection of abovementioned adult-trapping and infestation data, following three sampling sites/units, farmers’ fields and ginning factories were selected in each district.

**1: Cotton field** (A cotton field of 1 acre maintained by the farmer at local recommended production and protection practices was selected where two PBW-pheromone traps were installed with the help of bamboo poles at the height of 1.8 m in the middle of the field)

**2: Cotton Stick-heaps** (Sticks-heaps were selected on Farm house or in village where stick are stored for fuel purpose, where one PBW-pheromone traps was installed with the help of bamboo poles at the height of 1.8 m near the stick-heaps)

**3: Cotton ginning factory** (A Cotton ginning factory was selected in each district of respective districts where one PBW-pheromone traps was installed with the help of bamboo poles at the height of 1.8 m)

**COTTON FIELD AND GINNING FACTORY SURVEY LOCATIONS**

**Sahiwal**

1. Khan Faiz Khan, Chak No. 105/9L Raja Wala, Tehsiel Cheechawatni, District Sahiwal
2. Rana M. Khan and Co cotton Ginning and Oil Mil.
3. Asif Patwari and Abdul Majeed, Chak No. 186/187-9L, Tehsil and District Sahiwal
4. Chamba Industries Cotton Harappa Station, Sahiwal

**Vehari:**

1. Aurangzaib gujar, Chak No. 128-WB Garha Mour, Multan Road Vehari
2. Bilal Cotton Industry, Multan Road Vehari

**Khanewal:**

1. Arayain Farm, Haji Muhammad Shfqat, Kot Islam, Tehsiel kabirwala, District Khanewal
2. Imran Ginning Factory, Bagur Pul, Tehsil kabirwala, District Khanewal

**Faisalabad**

1. Pind Dogran Chak No. 39-GB, Tehsil Gojra, district Faisalabad

**Multan**

1. Naveed Zia, 363-W/B, Dunyia Pur, Multan
2. Tayyaba Ginning Factory, Multan

**Bahawalpur**

1. Aslam Shahzad Ghouri, Sui Wala Village, Jalalpur Per Wala road, District Bahawalpur
2. Muzamil cotton Factory, Lodhran Bahawalpur Road

**RESULTS**

**Vehari-1**

**Number of male adults captured per trap**

The number of male adults captured per trap tend vary throughout the sampling period among the cotton field, stick heaps and ginning factories.

**In the cotton field**, the adults appeared on 30^th^ May 2019 and the number of adults peaked (65 adults per trap) on 30^th^ July 2019. Then decrease in number was recorded and was highest (80 adults per trap) on 16^th^ September 2019. There was decline in the number of adults captured per trap after 16^th^ September 2019 and again attained a peak (59 adults per trap) at 30^th^ October 2019 then it declined gradually up to 16 November 2019 and was recorded zero on 30^th^ November 2019 (Fig. 1.1)

**In the stick heaps**, adults were observed on 16^th^ May 2019 and keeps on fluctuating. The number of adults captured per trap was highest (48 adults per trap) twice the sampling period on 16^th^ July and 30^th^ September 2019 and declined on 16^th^ November 2019 (Fig. 1.1)

**In the ginning factories**, the number of adults captured per trap was least as compared to the number of adults captured per trap recorded in cotton field and stick heaps. The adults were observed on 16^th^ May 2019 and peaked (30 adults per trap) on 30^th^ November 2019 while recorded zero on 16^th^ December 2019 (Fig. 1.1)

**Percent infested flowers**

The infestation of flowers was taken as percentage. Percent infestation was observed from 16^th^ June to 16^th^ November 2019. The percentage increased gradually and peaked (34 %) on 16^th^ November 2019 (Fig. 1.2)

**Percent unopened bolls infestation**

The infestation of unopened bolls was also taken in term of percentage. Percent unopened infested bolls were observed on 16^th^ July 2019 and infestation continue to increase and was at peak (17 %) on 16^th^ October 2019 and again at peak (28 %) on 16^th^ November 2019 (Fig. 1.3)

**Percent opened bolls infestation**

The infestation of opened bolls was also taken in term of percentage. Percent infested opened bolls were observed on 16^th^ July and it increased gradually and was at peak (163 %) on 30^th^ October 2019 (Fig. 1.4)

**Figure 1.1:** Pink bollworm adult population captured in traps (adult/trap/fortnight) installed in cotton fields, near sticks heaps and inside the ginning factories in district Vehari during 2019.

**Figure 1.2**: Total-flowers/plant, infested-bolls/plant and percent flower infestation (flowers infestation per plant) recorded in cotton field during 2019.

**Figure 1.3:** Total un-opened-bolls/plant, infested-unopened-bolls/plant and percent infestation of unopened-bolls/plant recorded during 2019.

**Figure 1.4:** Total opened-bolls/plant, infested opened-bolls/plant and percent infestation of opened-bolls/plant recorded during 2019.

**Khanewal**

**Number of male adults captured per trap**

The number of male adults captured per trap tend vary throughout the sampling period among the cotton field, stick heaps and ginning factories.

**In the cotton field**, the adults appeared on 05^th^ May 2019 and the number of adults increased gradually and peaked (45 adults per trap) on 20^th^ July 2019 and form second peak (41 adults per trap) on 20^th^ August 2019 and was highest (62 adults per trap) on 5^th^ September 2019. The gradual decrease in number was recorded up to 05^th^ December 2019 (Fig. 1.5)

**In the stick heaps**, adults were observed on 5^th^ May 2019 and keeps on fluctuating. The number of adults captured per trap was at peak (37adults per trap) on 20^th^ June 2019 and recorded highest (39 adults per trap) on 20^th^ July 2019 then declined gradually and again at peak on 20^th^ September 2019 and then declined (Fig. 1.5)

**In the ginning factories**, the number of adults captured per trap was least as compared to the number of adults captured per trap recorded in cotton field and stick heaps. The adults were observed on 20^th^ May 2019 and peaked (05adults per trap) on 20^th^ July 2019 and then the number decline gradually (Fig. 1.5)

**Percent infested flowers**

The infestation of flowers was taken as percentage. Percent infestation was observed from 5^th^ June to 20^th^ November 2019. The percentage increased gradually and peaked (3.3%) on 5^th^ June 2019 and then declined with sharp increase and was at peak (6.3%) on 5^th^ August 2019. The percent infested flower was highest (8%) on 5^th^ September 2019 and then declined gradually (Fig. 1.6)

**Percent unopened bolls infestation**

The infestation of unopened bolls was also taken in term of percentage. Percent unopened infested bolls were observed on 20^th^ July 2019 and was at peak (6.8%) on 20^th^ August 2019, infestation continue to increase and was highest (21%) on 5^th^ October 2019 and then decline up to 5^th^ September 2019 and was again at peak (46%) on 05^th^ November 2019 (Fig. 1.7)

**Percent opened bolls infestation**

The infestation of opened bolls was also taken in term of percentage. Percent infested opened bolls were observed on 5^th^ August and there was sharp increase and was highest (62%) on 5^th^ September 2019 and then keep on fluctuating with peaks (52 and 66%) on 5^th^ October and 5^th^ November 2019 respectively (Fig. 1.8)

**Figure 1.5:** Pink bollworm adult population captured in traps (adult/trap/fortnight) installed in cotton fields, near sticks heaps and inside the ginning factories in district Khanewal during 2019.

**Figure 1.6**: Total-flowers/plant, infested-bolls/plant and percent flower infestation (flowers infestation per plant) recorded in cotton field during 2019.

**Figure 1.7:** Total un-opened-bolls/plant, infested-unopened-bolls/plant and percent infestation of unopened-bolls/plant recorded during 2019.

**Figure 1.8:** Total opened-bolls/plant, infested opened-bolls/plant and percent infestation of opened-bolls/plant recorded during 2019.

**Bahawalpur**

**Number of male adults captured per trap**

The number of male adults captured per trap trend vary throughout the sampling period among the cotton field, stick heaps and ginning factories.

**In the cotton field**, the adults appeared on 22^nd^ April 2019 and the number of adults increased gradually and peaked (42 adults per trap) on 22nd July 2019 and was highest (50 adults per trap) on 22^nd^ September 2019. The gradual decrease in number was recorded up to 7^th^ November 2019 (Fig. 1.9).

**In the stick heaps**, adults were observed on 22^th^ April 2019 and keeps on fluctuating. The number of adults captured per trap was at peak (32 adults per trap) on 22^nd^ June 2019 and recorded highest (38 adults per trap) on 7^th^ July 2019 then decreased and again at peak (35 adults per trap) on 7^th^ August 2019 and then declined gradually up to 22^th^October 2019 (Fig. 1.9).

**In the ginning factories**, the number of adults captured per trap was least as compared to the number of adults captured per trap recorded in cotton field and stick heaps. The adults were observed on 7^th^ May 2019 and increased gradually and was highest (12 adults per trap) on 7^th^ July 2019 and then the number decline gradually up to 22^nd^ October 2019 (Fig. 1.9).

**Percent infested flowers**

The infestation of flowers was taken as percentage. Percent infestation was observed from 7^th^ June to 22^nd^ October 2019. The percentage increased gradually and peaked (171%) on 22^nd^ June 2019 and then keep on fluctuating and was highest (10%) on 22^nd^ July 2019 and again at peak (12%) on 22^nd^ September 2019. A sharp decline was observed up to 22^nd^ October 2019 (Fig. 1.10).

**Percent unopened bolls infestation**

The infestation of unopened bolls was also taken in term of percentage. Percent unopened infested bolls were observed on 7^th^ July 2019 and was at peak (20%) on 22^nd^ July 2019, and then declined gradually and was at peak (3.3%) 7^th^ October 2019 and then declined (Fig. 1.11).

**Percent opened bolls infestation**

The infestation of opened bolls was also taken in term of percentage. Percent infested opened bolls were observed on 07^th^ August 2019 and was highest (14.4%) on 22^nd^ August 2019 and then declined up to 07^th^ November 2019 (Fig. 1.12).

**Figure 1.9:** Pink bollworm adult population captured in traps (adult/trap/fortnight) installed in cotton fields, near sticks heaps and inside the ginning factories in district Bahawalpur during 2019.

**Figure 1.10**: Total-flowers/plant, infested-bolls/plant and percent flower infestation (flowers infestation per plant) recorded in cotton field during 2019.

**Figure 1.11:** Total un-opened-bolls/plant, infested-unopened-bolls/plant and percent infestation of unopened-bolls/plant recorded during 2019.

**Figure 1.12:** Total opened-bolls/plant, infested opened-bolls/plant and percent infestation of opened-bolls/plant recorded during 2019.

**Multan**

**Number of male adults captured per trap**

The number of male adults captured per trap tend vary throughout the sampling period among the cotton field, stick heaps and ginning factories.

**In the cotton field**, the adults appeared on 2^nd^ May 2019 and the number of adults increased gradually and peaked (35 adults per trap) on 17^th^ June 2019 and was highest (45 adults per trap) on 17^th^ July 2019. There was a sharp decline and again was at peak (32 and 70 adults per trap) on 17^th^ August and 2^nd^ October 2019 respectively. Gradual decrease in number was recorded up to 02^nd^ December 2019 (Fig. 1.13).

**In the stick heaps**, adults were observed on 2^nd^ May 2019 and keeps on fluctuating. The number of adults captured per trap was at peak (26 adults per trap) on 2^nd^ June 2019 and recorded highest (41 adults per trap) on 2^nd^ August 2019 then decreased and then declined gradually up to 2^nd^ December 2019 (Fig. 1.13).

**In the ginning factories**, the number of adults captured per trap was least as compared to the number of adults captured per trap recorded in cotton field and stick heaps. The adults were observed on 17^th^ May 2019 and was at peak (13 adults per trap) on 2^nd^ June 2019 and was highest (15 adults per trap) on 2 August 2019 and then the number decline gradually up to 2^nd^ December 2019 (Fig. 1.13).

**Percent infested flowers**

The infestation of flowers was taken as percentage. Percent infestation was observed from 17^th^ June to 2^nd^ November 2019. The percentage increased gradually and was highest (9.3%) on 2^nd^ August 2019. A sharp decline was observed up to 17^th^ October 2019 (Fig. 1.14).

**Percent unopened bolls infestation**

The infestation of unopened bolls was also taken in term of percentage. Percent unopened infested bolls were observed on 2^nd^ July 2019 and was at peak (10 %) on 17^th^ July 2019, infestation continue to fluctuate and was again at peak (4.7%) on 17^th^ September 2019 and highest (6.1%) on 2^nd^ November 2019 and then declined gradually (Fig. 1.15).

**Percent opened bolls infestation**

The infestation of opened bolls was also taken in term of percentage. Percent infested opened bolls were observed on 17^th^ August 2018 and was highest (8.9%) on 17^th^ September 2018 and then declined and again at peak (8.4%) on 2^nd^ November 2018 (Fig. 1.161.).

**Figure 1.13:** Pink bollworm adult population captured in traps (adult/trap/fortnight) installed in cotton fields, near sticks heaps and inside the ginning factories in district Multan during 2019.

**Figure 1. 14**: Total-flowers/plant, infested-bolls/plant and percent flower infestation (flowers infestation per plant) recorded in cotton field during 2019.

**Figure 1.15:** Total un-opened-bolls/plant, infested-unopened-bolls/plant and percent infestation of unopened-bolls/plant recorded during 2019.

**Figure 1.16:** Total opened-bolls/plant, infested opened-bolls/plant and percent infestation of opened-bolls/plant recorded during 2019.

**Sahiwal**

**Number of male adults captured per trap**

The number of male adults captured per trap tend vary throughout the sampling period among the cotton field, stick heaps and ginning factories.

**In the cotton field**, the adults appeared on 15^th^ May 2018 and the number of adults increased gradually and peaked (41 adults per trap) on 30^th^ June 2018 and was highest (60 adults per trap) on 15^th^ July 2018. There was a gradual decline and again was at peak (56 adults per trap) on 15^th^ September 2018. Gradual decrease in number was recorded up to 15^th^ November 2018 (Fig. 1.17).

**In the stick heaps**, adults were observed on 15^th^ May 2019 and keeps on fluctuating. The number of adults captured per trap was at peak (30 adults per trap) on 15^th^ June 2019 and again at peak (50 adults per trap) on 15^th^ July 2019 and again recorded peak (36 adults per trap) on 30^th^ August 2019 then decreased and again at peak (33 adults per trap) on 30^th^ September 2019 and then declined gradually up to 15^th^ November 2019 (Fig. 1.17).

**In the ginning factories**, the number of adults captured per trap was least as compared to the number of adults captured per trap recorded in cotton field and stick heaps. The adults were observed on 30^th^ May 2019 and was at peak (12 adults per trap) twice on 30^th^ June and 30^th^ July 2019 respectively and then the number decline gradually up to 15^th^ December 2019 (Fig. 1.17).

**Percent infested flowers**

The infestation of flowers was taken as percentage. Percent infestation was observed from 15^th^ June to 15^th^ October 2019. The percentage increased gradually and was at peak (17%) on 15^th^ July 2019 and was highest (19.5%) on 30^th^ August 2019. A sharp decline was observed up to 15^th^ November 2019 (Fig. 1.18).

**Percent unopened bolls infestation**

The infestation of unopened bolls was also taken in term of percentage. Percent unopened infested bolls were observed on 15^th^ July 2019 with a sharp increase recorded highest (16%) on 30^th^ August 2019 and then declined gradually upto 15^th^ November 2019 (Fig. 1.19).

**Percent opened bolls infestation**

The infestation of opened bolls was also taken in term of percentage. Percent infested opened bolls were observed on 15^th^ July 2019 and was at peak (16%) on 30^th^ July 2019 and was highest (10.11%) on 15^th^ November 2019 (Fig. 1.20).

**Figure 1.17:** Pink bollworm adult population captured in traps (adult/trap/fortnight) installed in cotton fields, near sticks heaps and inside the ginning factories in district Sahiwal during 2019.

**Figure 1.18**: Total-flowers/plant, infested-bolls/plant and percent flower infestation (flowers infestation per plant) recorded in cotton field during 2019.

**Figure 1.19:** Total un-opened-bolls/plant, infested-unopened-bolls/plant and percent infestation of unopened-bolls/plant recorded during 2019.

**Figure 1.20:** Total opened-bolls/plant, infested opened-bolls/plant and percent infestation of opened-bolls/plant recorded during 2019.

**Faisalabad**

**Number of male adults captured per trap**

The number of male adults captured per trap tend vary throughout the sampling period among the cotton field, stick heaps and ginning factories.

**In the cotton field**, the adults appeared on 10^th^ May 2019 and the number of adults increased gradually and peaked (18 adults per trap) on 10^th^ June 2019 and then decrease ant at peak (32 adults per trap) again on 10^th^ August 2019 and was highest (54 adults per trap) on 10^th^ October 2019. Gradual decrease in number was recorded up to 10^th^ December 2019 (Fig. 1.21).

**In the stick heaps**, adults were observed on 10^th^ May 2019 and keeps on fluctuating. The number of adults captured per trap was highest (33 adults per trap) on 30^th^ May 2019 and then declined and again at peak (35 adults per trap) on 10^th^ July 2019. The number of adults captured per trap then keep on fluctuating up to 10^th^ November 2019 (Fig. 1.21).

**In the ginning factories,** no record from Faisalabad district as no ginning factory (Fig. 1.21).

**Percent infested flowers**

The infestation of flowers was taken as percentage. Percent infestation was observed from 10^th^ June to 10^th^ November 2019. The percentage increased gradually and was at peak (20%) on 10^th^ July 2019 and was highest (31.25%) on 10^th^ November 2019 (Fig. 1.22).

**Percent unopened bolls infestation**

The infestation of unopened bolls was also taken in term of percentage. Percent unopened infested bolls were observed on 25^th^ May 2019 and keep fluctuating throughout the sampling period and was highest (36%) on 10^th^ November 2019 and then declined up to 10^th^ November 2019 (Fig. 1.23).

**Percent opened bolls infestation**

The infestation of opened bolls was also taken in term of percentage. Percent infested opened bolls were observed on 10^th^ August 2019 and was at peak (17 %) on 10^th^ October 2019 and was highest (28.5 %) on 10^th^ November 2019 (Fig. 1.24).

**Figure 1.21:** Pink bollworm adult population captured in traps (adult/trap/fortnight) installed in cotton fields, near sticks heaps and inside the ginning factories in district Faisalabad during 2019.

**Figure 1.22**: Total-flowers/plant, infested-bolls/plant and percent flower infestation (flowers infestation per plant) recorded in cotton field during 2019.

**Figure 1.23:** Total un-opened-bolls/plant, infested-unopened-bolls/plant and percent infestation of unopened-bolls/plant recorded during 2019.

**Figure 1.24:** Total opened-bolls/plant, infested opened-bolls/plant and percent infestation of opened-bolls/plant recorded during 2019.

**ACTIVITY-2: MORPHOLOGICAL AND DNA-BARCODING BASED IDENTIFICATION AND CHARACTERIZATION OF FIELD COLLECTED PBW POPULATION**

**YEAR 2018**

**A. Morphology based identification**

**Collection of Specimen (Larva)**

Larvae of Pink bollworm were collected from different varieties of cotton in 2018 from different locations of cotton growing belt of Punjab, province of Pakistan. For example Bahawalpur, Bhakkar, Dera Ghazi Khan, Faisalabad, Lodhran, Multan, Okara, Rahim Yar Khan, Rajin Pur, Sahiwal, and Vehari. The bolls having larvae were collected from the field and brought to the laboratory in the paper envelopes after writing the name of locality, the larvae were removed from the bolls and preserved in 75% alcohol in vials.

**Collection of Specimen (Adult)**

Adults of Pink bollworm were collected from different varieties of cotton in 2018 from different locations of cotton growing belt of Punjab, province of Pakistan. The adults were collected from the field and brought to the laboratory after writing name of the locality.

**Killing**

Poison bottles were used to kill the larvae and adults of Pink bollworm. A wide opening bottle, contains a thin layer of potassium cyanide at the bottom. A layer of pours material like dry plaster of paris was placed on potassium cyanide. Finally, half inch layer of wet plaster of paris was added. Both larvae and adults were killed by using this bottle.

**Labelling and Preservation**

After killing the adult specimens were pinned with No.16 pins. After that specimens were spread on setting board that having a cork layer for 10 to 12 hours, then shifted into wooden boxes that contains naphthalene bolls. A dry label from white stiff paper was used. These contain all important information regarding collection.

After killing the larvae of Pink bollworm were shifted into small vials which contains preservative. For wet preservation a liquid that contains ethyl alcohol formaldehyde and glycerin was used. Wet labels were also used for immature collection, these are written by lead pencil and finally placed in the liquid.

**Identification**

Collected specimens were identified upto species level by using suitable taxonomic keys. Different characters of larva, pupa and adult were described in detail for their future identification. **Genus *Pectinophora***

Type: *Gelechia gossypiella* Saunders

Labial palpi is long, recurved upward, terminal segments long and pinted. Maxillary palpi is minute. Thorax is at level or smooth, front wing is long pointed at the end and smooth, hind wing is broader than front wing and trapezoidal shaped, apex is pointed. Specimens with a fringe of large hairs on wings, outer margin of hind wing is slightly curved, mostly 2A in front wing is forked at the base.

***Pectinophora gossypiella* Saunders**

**Fig. 1-8 (larvae)**

*Pectinophora gossypiella* Saunders, 1843. Trans. Ent. Soc. London, 3: 284-285

**Body:** Larva is 11-13 mm long, width is 2-3.5 mm, body color is light pinkish, and dorsally 14 segments are visible excluding head. Morphologically two types of specimens were present first with a dark head with prothoracic shield backed by transverse pink band, second one with head prothoracic and body color is light pink

**Head:** Black in color, AF2 seta is at the top of front near epicranial suture. Ad frontal setae have widely separated, four teeth are visible on mandible, first three teeth are larger as compare to last one. Head is 2-4 mm long and 0.75 mm wide, antenna is short, stemmate and genal area is visible, frons is triangle shaped, clypeus is short and narrow from center, labrum with 15 to 20 setae, galea is pointed.

**Thorax:** Three thoracic segments are visible, prothoracic shield is present on first segment, crescent shaped markings are visible in prothoracic shield, markings not visible in specimen with black shield. Triangle shaped projection from prothoracic shield. First thoracic segment is visible, six setae are present on prothoracic shield, D1, D2, XD1, XD2, SD1 and SD2 are present, L1, L2, L3, spiracle is present on latrum, SV1 and SV2 are present on latero-ventral position, meso and meta thorax with 2 to 3 band like structure. Central band is narrow from middle and broad from sides, setae D1, D2, SD1, SD2, L1, L2, L3 and SVI present, thoracic shield absent, Terminal portion of prologs blackish in color.

**Abdomen**: Two reddish bands are present on all abdominal segments, first band is broad and second is narrow. First abdominal segment with setae D1, D2, SD1, SD2 (reduced) L1, L2, L3, SV, SV2, V1, spiracle is present. Second abdominal segment with setae D1, D2, SD1, SD2, L1, L2, L3, SV1, SV2, SV3 (addition) V1, spiracle are visible. Segments no 3 to 6 all with abdominal legs visible. Crochets are present in incomplete circle, 17 crochets are present on each abdominal leg. Third abdominal segment with setae D1, D2, SD1, SD2 L1, L2, L3, SV1, SV2, SV3 are present, V1 is absent, spiracle is visible, Seventh abdominal segment D1, D2, SD1, SD2, L1, L2, L3, SV1, SV2, V1 and spiracle present, SV3 is absent. The 8^th^ abdominal segment with D1, SD1. A8 is dorsad to the spiracle, D2, SD1, SD2, L1, L2, L3, SV1, V1, spiracle visible, SV2 and SV3 absent. 9^th^ abdominal segment with D1, D2, SD1, L1, L2, SV1 and V1 present. SD1 is seta form, SD2, L3, SV2. Spiracle are absent, anal shield present. 10^th^ abdominal segment, D1, D2, SD1 SD2 and anal proleg is present, 11 to 14 crochets are present on each anal leg, crochets filling almost half of anal proleg.


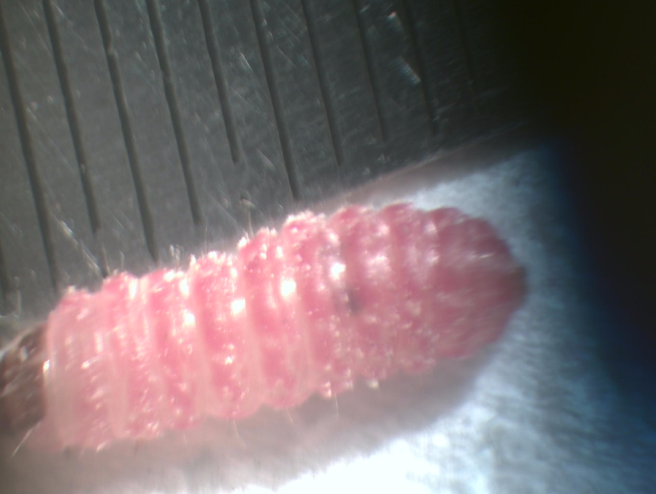

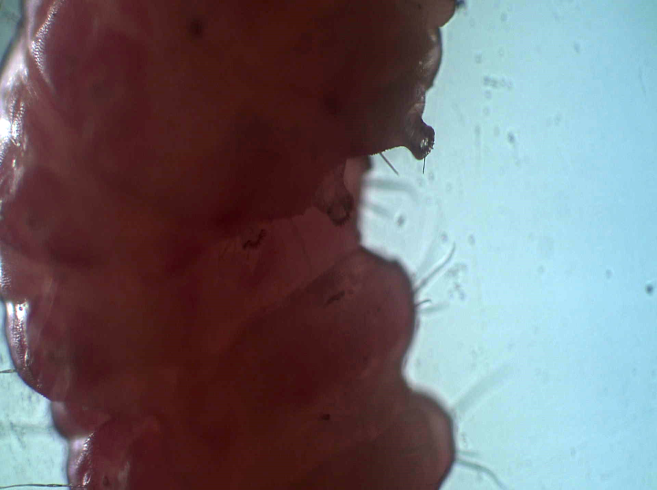


Fig. 1 Fig. 2


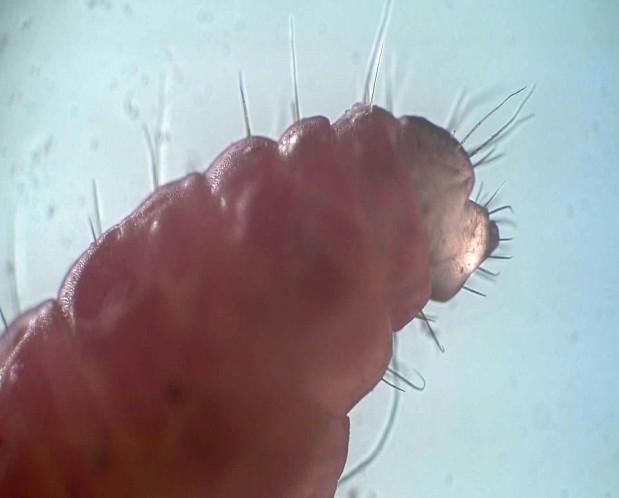

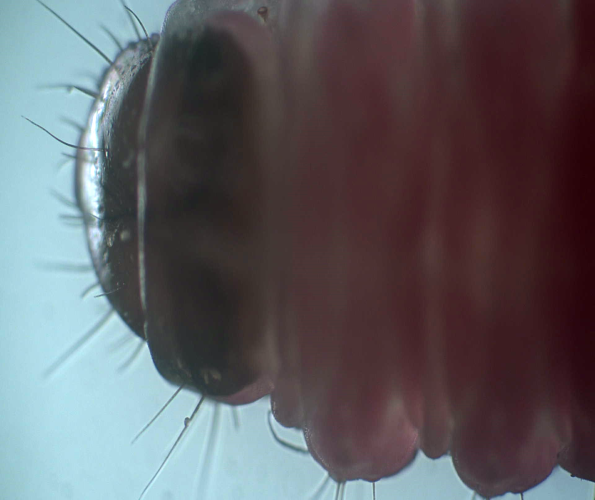


Fig.3 Fig.4


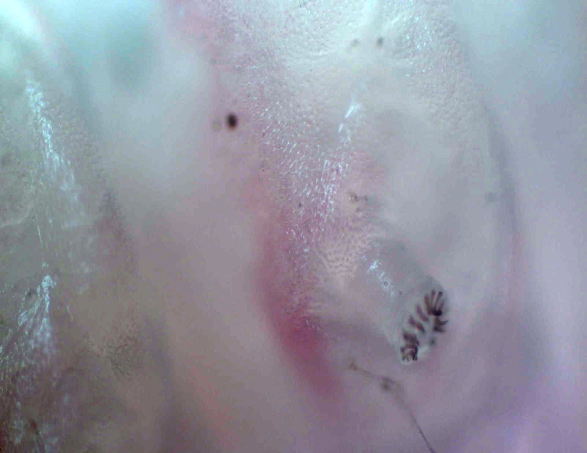

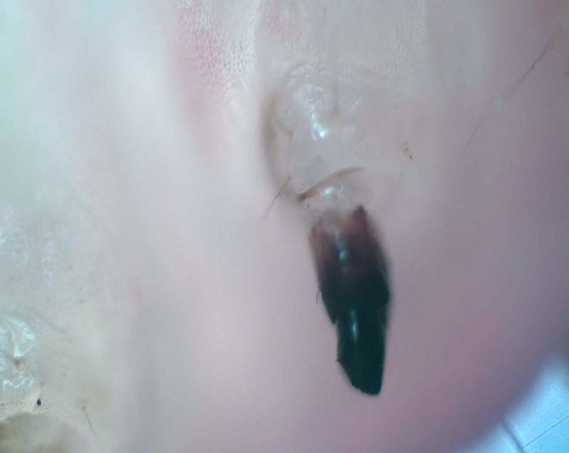


Fig. 5 Fig. 6


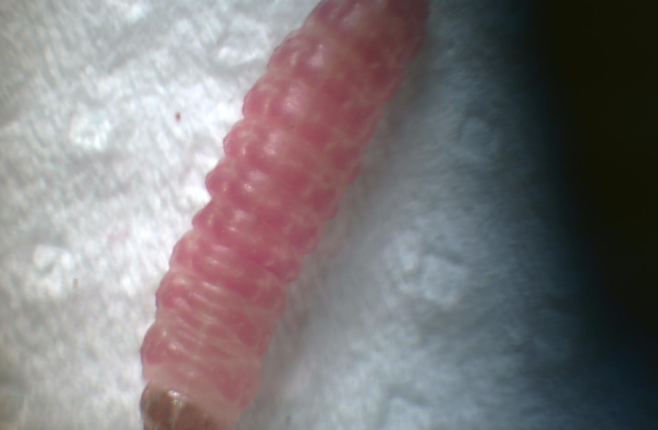

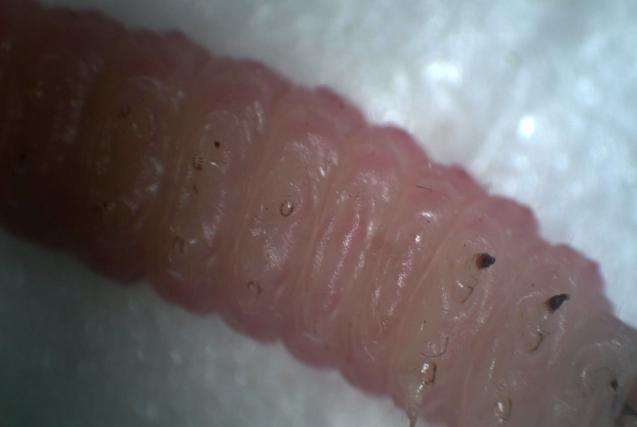


Fig. 7 Fig. 8

***Pectinophora gossypiella***

**Fig. 9-12 (pupa)**

*Pectinophora gossypiella* Saunders, 1843. Trans. Ent. Soc. London, 3: 284-285

Pupa is obtect or chrysalis, appendages are firmly glued to the body, Pupa is 9 to 11 mm, smooth body, and reddish brown color. Pupal eyes are visible, mostly 3 to 4 setae are visible on vertex. All thoracic and abdominal segments with small yellowish setae, pair of setae are present near the spiracles, mostly hooked like setae are present on 5^th^ to 9^th^ abdominal segment, slit like anal opening. 8 to 12 hooked like setae are present near anal opening, antenna is also glued and reaching at the tip of wings. Vertical suture is extending up to the end of third thoracic segment. First thoracic segment is short, second is broad and long, transverse or m shaped suture is present on second thoracic segment, 3^rd^ thoracic segment is long and extending up to the sides of 3^rd^ abdominal segment, first abdominal segment is shorter, 2^nd^, 3^rd^and 4^th^ abdominal segments are equal in length, 5^th^ to 10^th^ abdominal segments are narrowing towards end. All parts of antenna, labrum, front, clypus, gena, maxillary palpus, maxilla, three legs, wings and anal opening are visible.


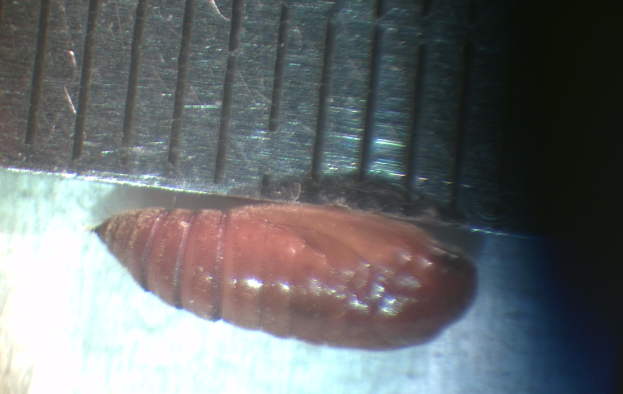

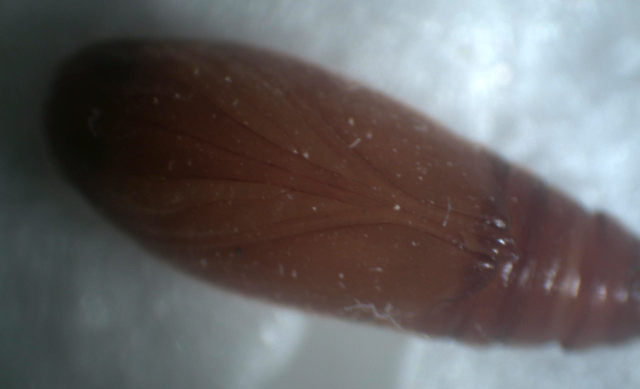


Fig.9 Fig.10


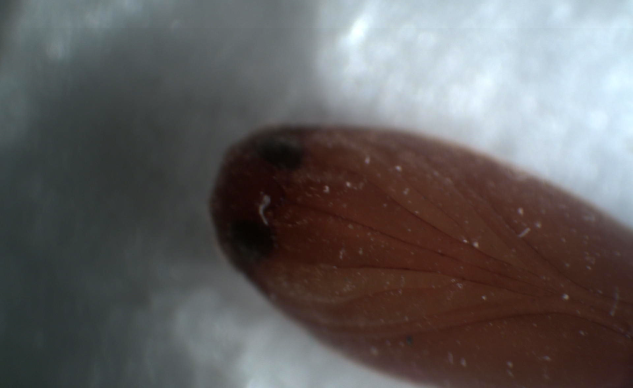

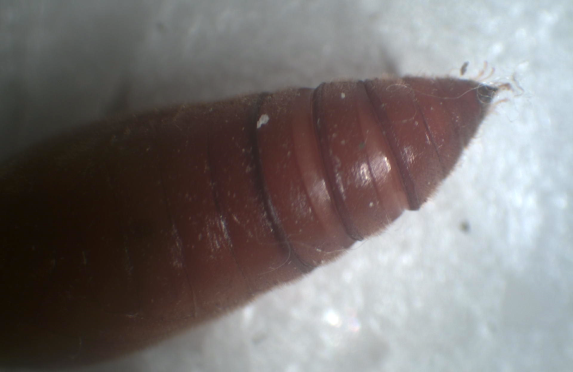


Fig.11 Fig.12

***Pectinophora gossypiella***

**Fig. 13-21(Adult)**

*Pectinophora gossypiella* Saunders, 1843. Trans. Ent. Soc. London, 3: 284-285

**Head:** Labial palpi is yellowish brown, two white patches are present near base, diffused black spots are absent, labial palpi upward curved, narrowing towards apex. Antenna is 4 to 5 mm in length, 0.1 mm wide, slightly narrowing towards apex, covered with scales, yellowish in color, pedicel with irregular black patch, 1^st^ segment of flagellum is more than half cover with black scales, black scales are not visible from segment no 2 to 5, reddish and yellowish scales are present, 6 to 7 blacks spots are present from segment no 6 to 11, 3^rd^ terminal segment is also with black spot. Eyes are visible and less than 1mm wide, slightly protruded from head. Head covered with black, brown and orange scales, white and black scales on posterior side of vertex forming two semi-circle shaped spots,

**Thorax:** Reddish brown with sparkling of black and white scales, patagium (covers wing joint) are pad like and visible, basal portion is fully covered with black scales and rest with black and white scales.

**Wings:** Front wing is 9-11 mm long**,** 2-3 mm wide, ovate, smooth and pointed tip, 12 longitudinal veins are present in front wing, costa is independent and terminating up to the half-length of front wing. Costa is branched at base, 10 veins are emerging from a longitudinal cell, last anal vein is highly bifurcate at base, apical portion with long, ocherous or blackish hairs, front wings darker brown with irregular ill-defined, black spots, scales on wings are slightly broader at top, basal portion densely dusted with black scales, middle portion is lighter in colour, apical portion again with black scale, Hind wings is broader than forewing, it is dark fuscous, iridescent, lightest towards base, apical portion is pointed with long hairs, 8 veins are present in hindwing, costa is highly deflected from the middle, subcosta and radius are almost fused at base, frenulum is visible, simple in males, frenulum triple in the females.

**Legs:** Coxa with alternate bands of black and white scales, basal portion of femur is dusted with black scales, middle portion is lighter in col, hind portion is reddish or orange in color, tibia is long, reddish scales are present in wavy lines, long hairs are present at the junction of tibia and tarsal segments, tarsal segments with white strips.

**Abdomen:** 6 to 7 mm long, 2 to 3 mm wide abdomen is flattened, laterally ocherous dark brown, underside suffused with black scales at the joints. Abdomen is similar in male and female, white scales are present between segments, segments are narrowing posteriorly. Female ovipositor is weakly chitinized, fully covered with stiff and hairs, genital plate is cordate shaped, male genitalia is small, aedeagus short, thick, terminal hook is visible.

**Specimens collected from the cotton belt of Punjab tally with the description of Saunders (1843).**


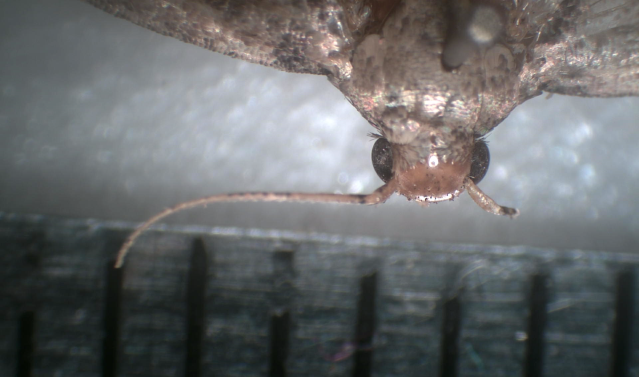

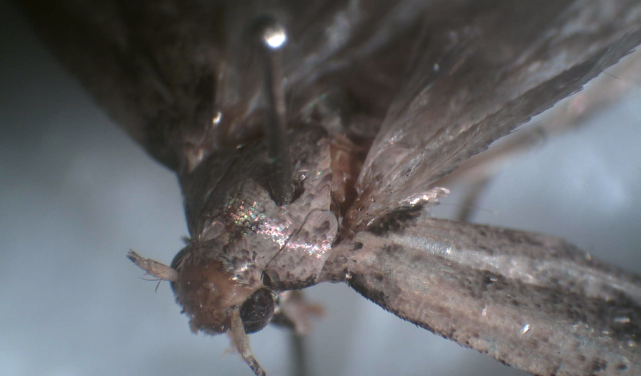


Fig. 13 Fig. 14


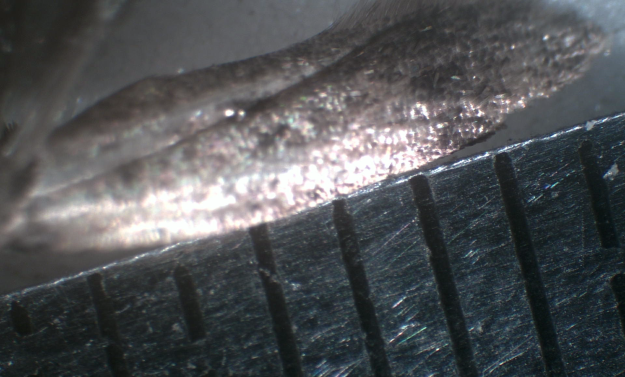
 **
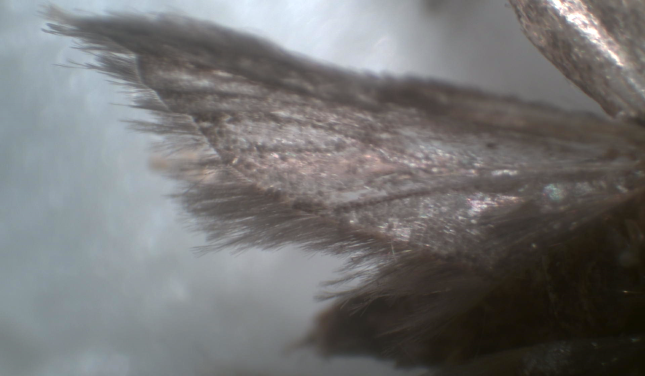
**

Fig. 15 Fig. 16

**
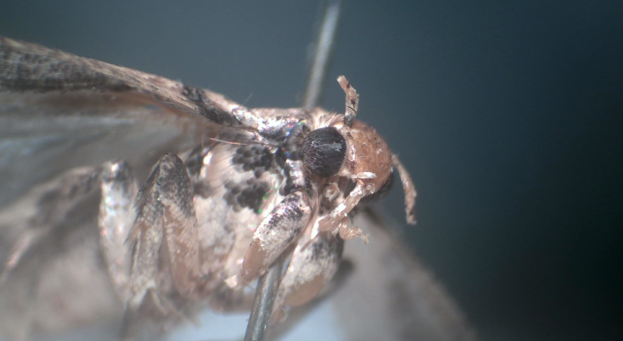
**
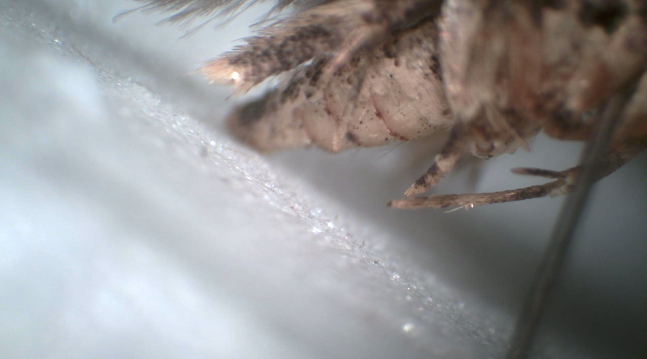


Fig. 17 Fig. 18


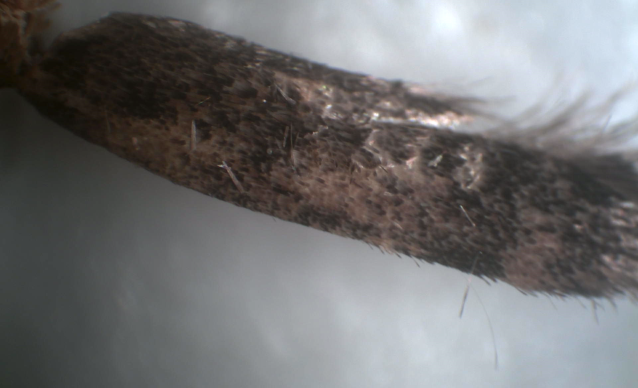

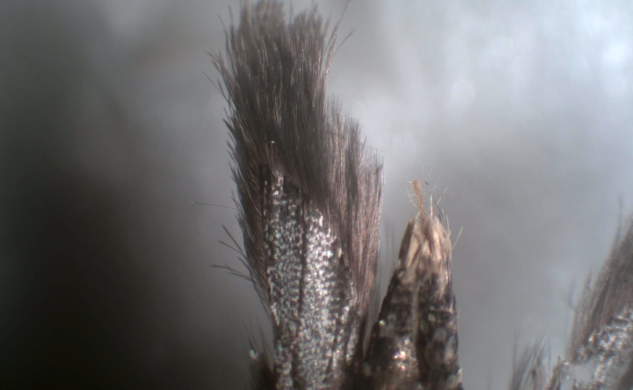


Fig. 19 Fig. 20


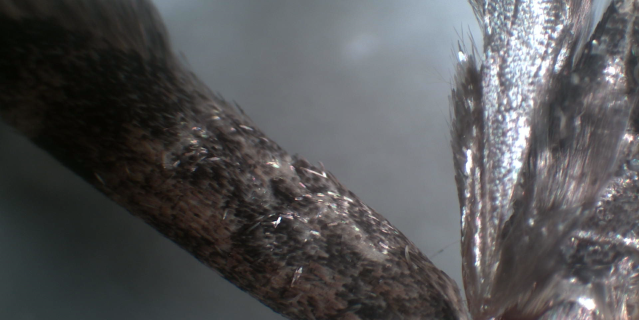


Fig. 21

**Material examined**

30 larvae, on Cotton, Faisalabad, 25-09-2018, M. Tayyib; 5 adults, on Cotton, Faisalabad, 25-09-2018, M. Tayyib; 11 larvae, on Cotton, Faisalabad, 10-10-2018, M. Tayyib; 3 adults, on Cotton, Faisalabad, 10-10-2018, M. Tayyib; 13 larvae, on Cotton, Jhang, 08-09-2018, M. Tayyib; 4 adults, on Cotton, Jhang, 08-09-2018, M. Tayyib; 8 larvae, on Cotton, Jhang, 15-09-2018, M. Tayyib; 13 adults, on Cotton, Jhang, 15-9-2018, M. Tayyib; 16 larvae, on Cotton, Jhang, 8-10-2018, M. Tayyib; 2 adults, on Cotton, Jhang, 08-10-2018, M. Tayyib; 7 larvae, on Cotton, Jhang, 15-10-2018, M. Tayyib; 4 adults, on Cotton, Jhang, 15-10-2018, M. Tayyib; 6 larvae, on Cotton, Bahawalpur, 22-08-2018, M. Tayyib; 4 adults, on Cotton, Bahawalpur, 22-08-2018, M. Tayyib; 1 larvae, on Cotton, Bahawalpur, 07-09-2018, M. Tayyib; 3 adults, on Cotton, Bahawalpur, 07-09-2018, M. Tayyib; 9 larvae, on Cotton, Bahawalpur, 22-09-2018, M. Tayyib; 3 adults, on Cotton, Bahawalpur, 22-09-2018, M. Tayyib; 7 larvae, on Cotton, Bahawalpur, 07-10-2018, M. Tayyib; 3 adults, on Cotton, Bahawalpur, 07-10-2018, M. Tayyib; 17 larvae, on Cotton, Bahawalpur, 22-10-2018, M. Tayyib; 6 adults, on Cotton, Bahawalpur, 22-10-2018, M. Tayyib; 12 larvae, on Cotton, Bhakkar, 18-09-2018, M. Tayyib; 6 adults, on Cotton, Baukkar, 18-09-2018, M. Tayyib; 2 larvae, on Cotton, Baukkar, 25-09-2018, M. Tayyib; 3 adults, on Cotton, Baukkar, 25-09-2018, M. Tayyib; 1 larvae, on Cotton, Baukkar, 02-10-2018, M. Tayyib; 5 adults, on Cotton, Baukkar, 02-10-2018, M. Tayyib; 2 larvae, on Cotton, Baukkar, 10-10-2018, M. Tayyib; 1 adults, on Cotton, Baukkar, 10-10-2018, M. Tayyib; 2 larvae, on Cotton, D.G. Khan, 15-10-2018, M. Tayyib; 1 adults, on Cotton, D.G. Khan, 15-10-2018, M. Tayyib; 12 larvae, on Cotton, D.G. Khan, 22-10-2018, M. Tayyib; 11 adults, on Cotton, D.G. Khan, 22-10-2018, M. Tayyib; 8 larvae, on Cotton, Rajanpur, 05-10-2018, M. Tayyib; 11 adults, on Cotton, Rajinpur, 05-10-2018, M. Tayyib; 4 larvae, on Cotton, Rajinpur, 18-10-2018, M. Tayyib; 11 adults, on Cotton, Rajinpur, 18-10-2018, M. Tayyib; 2 larvae, on Cotton, Rajinpur, 28-10-2018, M. Tayyib; 11 adults, on Cotton, Rajinpur, 28-10-2018, M. Tayyib; 2 larvae, on Cotton, Lodhran, 22-08-2018, M. Tayyib; 11 adults, on Cotton, Lodhran, 22-08-2018, M. Tayyib; 2 larvae, on Cotton, Lodhran, 07-09-2018, M. Tayyib; 1 adults, on Cotton, Lodhran, 07-09-2018, M. Tayyib; 12 larvae, on Cotton, Lodhran, 22-09-2018, M. Tayyib; 1 adults, on Cotton, Lodhran, 22-09-2018, M. Tayyib; 14 larvae, on Cotton, Multan, 02-09-2018, M. Tayyib; 12 adults, on Cotton, Multan, 02-09-2018, M. Tayyib; 1 larvae, on Cotton, Multan, 17-09-2018, M. Tayyib; 12 adults, on Cotton, Multan, 17-09-2018, M. Tayyib; 3 larvae, on Cotton, Multan, 02-10-2018, M. Tayyib; 1 adults, on Cotton, Multan, 02-10-2018, M. Tayyib; 13 larvae, on Cotton, Vehari, 16-09-2018, M. Tayyib; 1 adults, on Cotton, Vehari, 16-09-2018, M. Tayyib; 1 larvae, on Cotton, Vehari, 30-09-2018, M. Tayyib; 1 adults, on Cotton, Vehari, 30-09-2018, M. Tayyib; 7 larvae, on Cotton, Rahim Yar Khan, 16-10-2018, M. Tayyib; 6 adults, on Cotton, Rahim Yar Khan, 16-10-2018, M. Tayyib; 6 larvae, on Cotton, Rahim Yar Khan, 30-10-2018, M. Tayyib; 6 adults, on Cotton, Rahim Yar Khan, 30-10-2018, M. Tayyib;

**B. DNA-barcoding based identification and characterization of field collected *Pectinophora gossypiella* from cotton fields of Punjab, Pakistan**

**ABSTRACT**

*Pectinophora gossypiella* (Lepidoptera: Gelichidae) is monophagus and cause serious damage to cotton crops worldwide. Therefore, they have ability to survive on host plant in the ecosystem, because of their high movement, fecundity rate and capability for development of insecticides resistance against wide range of pesticides. In this study, by using mitochondrial cytochrome oxidase I (COI) gene, molecular identification and phylogenetic relationship of *pectinophora gossypiella* was studied. The total genomic DNA was extracted and PCR was done using COI based primer pairs. Amplified PCR products was purified and sequenced. The alignment of the PCR amplified DNA fragments from COI for various bollworms of cotton were performed through ClustalW. The maximum likelihood analysis was done using MEGA6 software. The result of PCR sequencing and phylogenetic analysis indicated that the studied *P. gossypiella* samples have 99-100% identity with NCBI submitted specimens reported from other countries.

**VI. Materials and Methods**

**Insect Culture**

The samples of cotton bollworms *Pectinophora gossypiella* were collected from different fields of cotton growing farmers of Punjab and research institutes fields, University of Agriculture Faisalabad (UAF), Ayyub Agricultural Research Institute (AARI), Faisalabad, Central Cotton Research Institute, Multan (CCRI), during Sep 2017-18. The transparent jars were utilized for their maintenance using fresh cotton leaves and bolls. Adult insects were examined for correct species identification.

**Molecular Study**

The DNAeasy DNA extraction Kit (Quiagen, Germany) or CTAB method was used to extract DNA from Meta legs of adult insects, according to DNA extraction method used by Fukova et al. (2008). The insects used for mtCO1 analysis was deposited at IGCDB laboratory. The extracted total genomic DNA was observed visually using 0.8 to 1% agarose gel. It will be quantified on picodrop using standard procedures of Nucleic acid quantification. DNA samples was diluted using ddH2O depending upon the required concentration, to get a working solution (10-30 ng/μL). A quantity of the total DNA was preserved in ten percent glycerol at -80 °C. The CO1 region was amplified using primers pairs LCO 1480 and HCO 1298 (Folmer et al. 1994). The PCR conditions for 25 μL reaction volume was (2.5 μL of 10 X PCR buffer) with 25 mM (2 μL) of MgCl2, (0.5 μL) of 10 mM dNTPs, (0.5 μL) each of forward and reverse primer, IU of TaqDNA polymerase, 17 μL of ddH2O (Invitrogen). The alignment of all sequences was done using BioEdit 4.0 program of using ClustalW 1.8 (Thompson *et al.*, 1994) then, for sequences identity confirmation. The alignment was further studied employing MEGA 5.0. Phylogenetic analysis and diversity analysis for all sequences of targeted insect was used. Then, sequences graphically were displayed by tree construction through Maximum Likelihood (ML) using program MEGA 5.05 (Tamura *et al.,* 2011).

**RESULTS**

**Polymerase Chain Reaction Analysis (PCR)**

The amplification of DNA after genomic DNA confirmation was done from insects species using COI (F/R) based primers LCO-1490/ HCO-2198 and C1J2195/ TL2N3014 of cytochrome oxidase I (COI) gene. Total DNA samples were used during PCR amplification. The PCR reaction mixture up to 20 µl was prepared for PCR amplification and 30-50 ng quantity of each DNA sample was used for PCR amplification. Using COI primers, all DNA samples of *H. armigera* were amplified successfully and size was measured with 1 kb DNA ladder (GeneMark Company) on 2% agarose gel. The expected 710 bp DNA fragment was successfully visualized in 1.5% agarose gel as depicted in Fig. 1.25. The amplified DNA fragments were eluted and then DNA fragments samples were sent to M/s. Macrogen Company (Korea) for the sequencing.

**Sequence alignment and phylogenetic analysis**

The nucleotide sequencing of DNA fragments obtained from M/s Macrogen (Korea) was (Seq >180319-003_O09-40 PBW1 LCO 1490 (Pak-Seq PBW**) (Fig. 1.26)** analyzed and aligned through BLASTn (Basic Local Alignment Search Tool) with sequence data *P. gossypiella* previously reported in NCBI (National Centre of Biotechnology Information) site. The reference accession numbers of sequences used for alignment based on LCO-1490/ HCO-2198 primers were as; *P. gossypiella* ([KM289071.1](https://www.ncbi.nlm.nih.gov/nucleotide/MG437191.1?report=genbank&log$=nucltop&blast_rank=1&RID=26R1G5BA015), [KX863147.1](https://www.ncbi.nlm.nih.gov/nucleotide/AB620129.1?report=genbank&log$=nucltop&blast_rank=10&RID=26R1G5BA015), [KF643170.1](https://www.ncbi.nlm.nih.gov/nucleotide/AB643667.1?report=genbank&log$=nucltop&blast_rank=24&RID=26R1G5BA015), KF 491994 Aand GQ853429.1), *P. endema* ([KF393092.1](https://www.ncbi.nlm.nih.gov/nucleotide/KJ930516.1?report=genbank&log$=nucltop&blast_rank=13&RID=26R1G5BA015)), *S. exigua* (KJ634295.1) *S. frugiperda* (KJ634298.1), *H. armigera* (KP210095.1) *S. litura* (KX863232.1, KX862420.1 and KF153858.1 ) as shown in **Fig. 1.27**. In base pair sequence alignment, homology of *P. gossypiella* was compared with previously reported NCBI database through BLAST option. Pairwise alignment of dendrogram tree indicated that our sample (Seq >180319-003_O09-40 PBW1 LCO 1490 (Pak-Seq PBW) being clubbed in same cluster as **(***P. gossypiella* ([KM289071.1](https://www.ncbi.nlm.nih.gov/nucleotide/MG437191.1?report=genbank&log$=nucltop&blast_rank=1&RID=26R1G5BA015), [KX863147.1](https://www.ncbi.nlm.nih.gov/nucleotide/AB620129.1?report=genbank&log$=nucltop&blast_rank=10&RID=26R1G5BA015), [KF643170.1](https://www.ncbi.nlm.nih.gov/nucleotide/AB643667.1?report=genbank&log$=nucltop&blast_rank=24&RID=26R1G5BA015), KF 491994.1) and GQ853429.1 as outgroup) with 99% with good query coverage 82-97%. The other insects indicated similarity of 96-90 % respectively and formed different clusters in phylogenetic tree (Fig. 1.28).


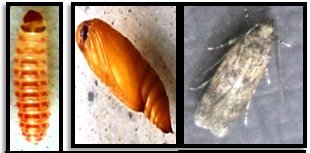


**Fig 1.25**: Pink bollworm collection (*P. gossypiella*) in cotton infested field. From left to right, Larva, Pupa and adult.


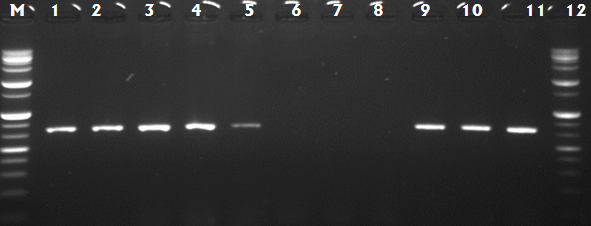


**Fig 1.26**: DNA amplification of *P. gossypiella* using mCOI (F/R) primers in PCR; Lane 6-8 (- control); Lanes 1-5, 9-12 DNA from *P. gossypiella of various regions of cotton zones*. M, 1 kb DNA ladder Marker (GeneMark)

**CATAAAGATATTGGAACTTTATACTTTATTTTTGGAATTTGAGCTGGAATAGTAGGTATATCTTTAAGTTTATTAATTCG** **TTTATTAATTCG**

**AGCTGAATTAGGTAACCCAGGATCTTTAATTGGTGATGATCAAATTTATAATACTATTGTCACTGCTCATGCTTTTATTA**

**AGCTGAATTAGGTAACCCAGGATCTTTAATTGGTGATGATCAAATTTATAATACTATTGTCACTGCTCATGCTTTTATTA**

**TAATTTTCTTCATAGTTATACCAATTATAATTGGAGGATTCGGAAATTGATTAGTACCTTTAATATTAGGAGCCCCTGAT**

**TAATTTTCTTCATAGTTATACCAATTATAATTGGAGGATTCGGAAATTGATTAGTACCTTTAATATTAGGAGCCCCTGAT**

**ATAGCTTTTCCTCGAATAAATAATATAAGTTTTTGACTTTTACCCCCCTCATTAACTCTTTTAATTTCAAGAAGAATTGT**

**ATAGCTTTTCCTCGAATAAATAATATAAGTTTTTGACTTTTACCCCCCTCATTAACTCTTTTAATTTCAAGAAGAATTGT**

**AGAAAATGGAGCAGGAACCGGATGAACAGTTTACCCCCCACTTTCATCTAATATTGCTCATGGAGGAAGTTCAGTAGATC**

**AGAAAATGGAGCAGGAACCGGATGAACAGTTTACCCCCCACTTTCATCTAATATTGCTCATGGAGGAAGTTCAGTAGATC**

**TGGCAATTTTTTCTTTACATTTAGCAGGTATTTCATCAATTTTAGGAGCAATTAACTTTATTACTACAATTATTAATATA**

**TGGCAATTTTTTCTTTACATTTAGCAGGTATTTCATCAATTTTAGGAGCAATTAACTTTATTACTACAATTATTAATATA**

**CGAATTAATGGTTTATCATTCGATCAAATACCATTATTTGTTTGAGCTGTAGGAATTACAGCCTTATTATTACTTTTATC**

**CGAATTAATGGTTTATCATTCGATCAAATACCATTATTTGTTTGAGCTGTAGGAATTACAGCCTTATTATTACTTTTATC**

**ATTACCTGTTTTAGCAGGAGCTATTACTATATTACTAACAGATCGAAATTTAAATACTTCATTTTTTGATCCAGCTGGTG**

**ATTACCTGTTTTAGCAGGAGCTATTACTATATTACTAACAGATCGAAATTTAAATACTTCATTTTTTGATCCAGCTGGTG**

**GAGGAGATCCAATCCTATACCAACACTTATTTTGATTTTTTGGTCACCCT**

**GAGGAGATCCAATCCTATACCAACACTT**

**Fig. 1.27.** Multiple alignments of DNA sequences through CLUSTAL OMEGA from DNA amplification of *P. gossypiella* (>180319-003_O09-43PBW (Pak-Seq PBW) with NCBI GenBank submitted sequences, (KM289071.1 PBW)


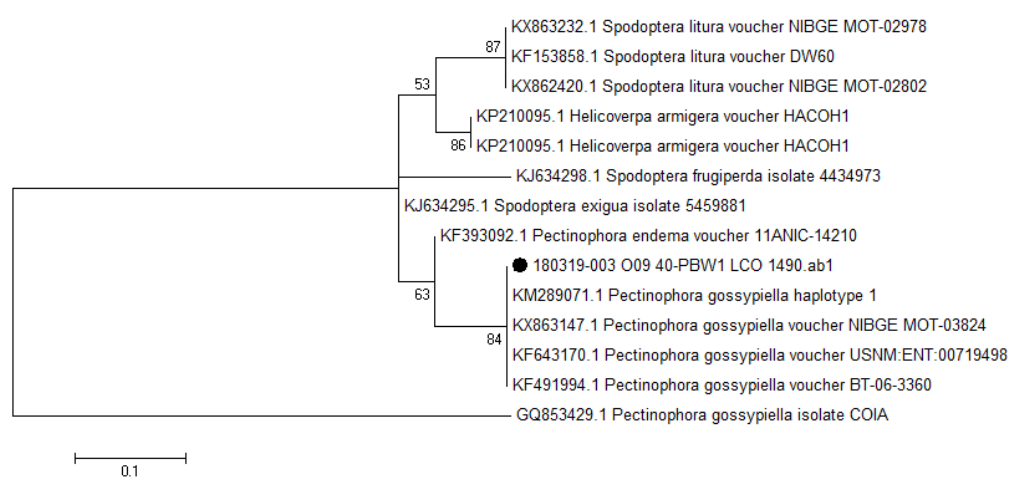


**Fig. 1.28.** Molecular phylogenetic analysis by Maximum Likelihood Method. Phylogenetic Tree produced from DNA amplification of *P. gossypiella* using mitochondrial Cytochrome oxidase I (mCOI) (F/R) gene based primers.

**YEAR 2019**

**A. Morphology based identification**

**Collection of Specimen (Larva)**

Larvae of Pink bollworm were collected from different varieties of cotton in 2019 from different locations of cotton growing belt of Punjab, province of Pakistan. For example Bahawalpur, Bhakkar, Dera Ghazi Khan, Faisalabad, Lodhran, Multan, Okara, Rahim Yar Khan, Rajin Pur, Sahiwal, and Vehari. The bolls having larvae were collected from the field and brought to the laboratory in the paper envelopes after writing the name of locality, the larvae were removed from the bolls and preserved in 75% alcohol in vials.

**Collection of Specimen (Adult)**

Adults of Pink bollworm were collected from different varieties of cotton in 2019 from different locations of cotton growing belt of Punjab, province of Pakistan. The adults were collected from the field and brought to the laboratory after writing name of the locality.

**Killing**

Poison bottles were used to kill the larvae and adults of Pink bollworm. A wide opening bottle, contains a thin layer of potassium cyanide at the bottom. A layer of pours material like dry plaster of paris was placed on potassium cyanide. Finally half inch layer of wet plaster of paris was added. Both larvae and adults were killed by using this bottle.

**Labelling and Preservation**

After killing the adult specimens were pinned with No.16 pins. After that specimens were spread on setting board that having a cork layer for 10 to 12 hours, than shifted into wooden boxes that contains naphthalene bolls. A dry label from white stiff paper was used. These contain all important information regarding collection.

After killing the larvae of Pink bollworm were shifted into small vials which contains preservative. For wet preservation a liquid that contains ethyl alcohol formaldehyde and glycerin was used. Wet labels were also used for immature collection, these are written by lead pencil and finally placed in the liquid.

**Identification**

Collected specimens were identified upto species level by using suitable taxonomic keys. Different characters of larva, pupa and adult were described in detail for their future identification.

**Genus *Pectinophora***

Type: *Gelechia gossypiella* Saunders

Labial palpi is long, recurved upward, terminal segments long and pinted. Maxillary palpi is minute. Thorax is at level or smooth, front wing is long pointed at the end and smooth, hind wing is broader than front wing and trapezoidal shaped, apex is pointed. Specimens with a fringe of large hairs on wings, outer margin of hind wing is slightly curved, mostly 2A in front wing is forked at the base.

***Pectinophora gossypiella* Saunders**

**Fig. 1-8 (larvae)**

*Pectinophora gossypiella* Saunders, 1843. Trans. Ent. Soc. London, 3: 284-285

**Body:** Larva is 11-13 mm long, width is 2-3.5 mm, body color is light pinkish, and dorsally 14 segments are visible excluding head. Morphologically two types of specimens were present first with a dark head with prothoracic shield backed by transverse pink band, second one with head prothoracic and body color is light pink (Fig. 1.1.1).

**Head:** Black in color, AF2 seta is at the top of front near epicranial suture. Ad frontal setae have widely separated, four teeth are visible on mandible, first three teeth are larger as compare to last one. Head is 2-4 mm long and 0.75 mm wide, antenna is short, stemmate and genal area is visible, frons is triangle shaped, clypeus is short and narrow from center, labrum with 15 to 20 setae, galea is pointed (Fig. 1.1.4).

**Thorax:** Three thoracic segments are visible, prothoracic shield is present on first segment, crescent shaped markings are visible in prothoracic shield, markings not visible in specimen with black shield. Triangle shaped projection from prothoracic shield. First thoracic segment is visible, six setae are present on prothoracic shield, D1, D2, XD1, XD2, SD1 and SD2 are present, L1, L2, L3, spiracle is present on latrum, SV1 and SV2 are present on latero-ventral position, meso and meta thorax with 2 to 3 band like structure. Central band is narrow from middle and broad from sides, setae D1, D2, SD1, SD2, L1, L2,L3 and SVI present, thoracic shield absent, Terminal portion of prologs blackish in color (Fig. 1.1.2).

**Abdomen**: Two reddish bands are present on all abdominal segments, first band is broad and second is narrow. First abdominal segment with setae D1, D2, SD1, SD2 (reduced) L1, L2, L3, SV, SV2, V1, spiracle is present. Second abdominal segment with setae D1, D2, SD1, SD2, L1, L2, L3, SV1, SV2, SV3 (addition) V1, spiracle are visible. Segments no 3 to 6 all with abdominal legs visible. Crochets are present in incomplete circle, 17 crochets are present on each abdominal leg. Third abdominal segment with setae D1, D2, SD1, SD2 L1, L2, L3, SV1, SV2, SV3 are present, V1 is absent, spiracle is visible, Seventh abdominal segment D1, D2, SD1, SD2, L1, L2, L3, SV1, SV2, V1 and spiracle present, SV3 is absent. The 8^th^ abdominal segment with D1, SD1. A8 is dorsad to the spiracle, D2, SD1, SD2, L1, L2, L3, SV1, V1, spiracle visible, SV2 and SV3 absent. 9^th^ abdominal segment with D1, D2, SD1, L1, L2, SV1 and V1 present. SD1 is setaform, SD2, L3, SV2. Spiracle are absent, anal shield present. 10^th^ abdominal segment, D1, D2, SD1 SD2 and anal proleg is present, 11 to 14 crochets are present on each anal leg, crochets filling almost half of anal proleg (Fig. 1.1.3).


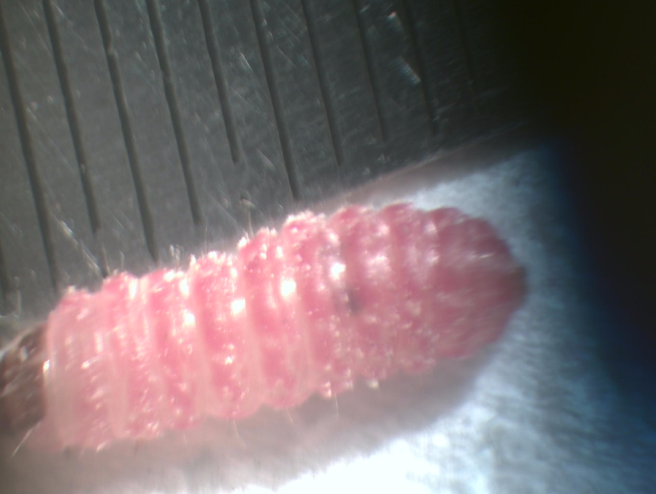

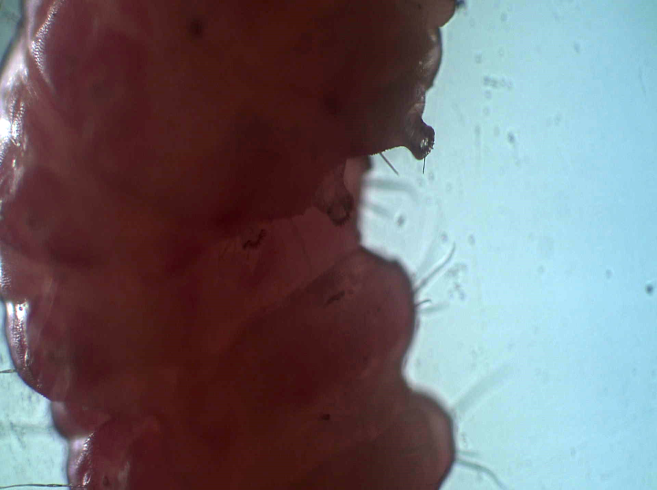


Fig. 1.1.1 Fig. 1.1.2


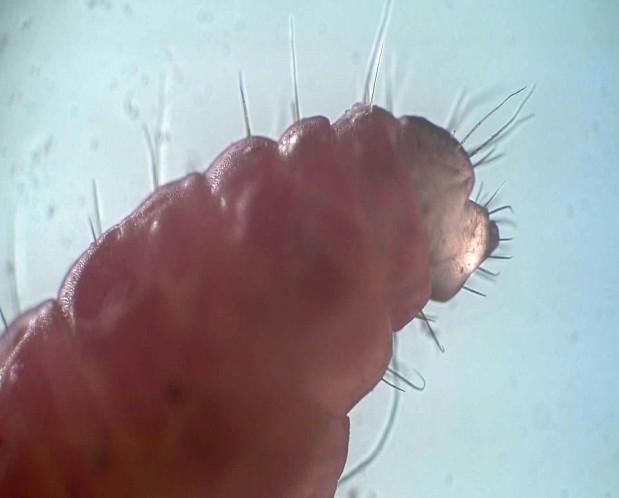

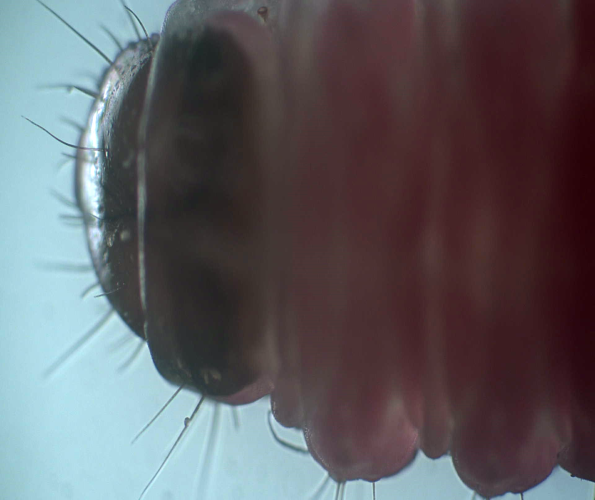


Fig.1.1.3 Fig.1.1.4


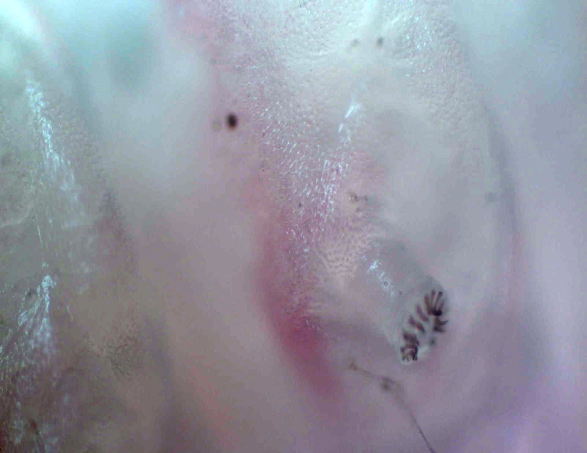

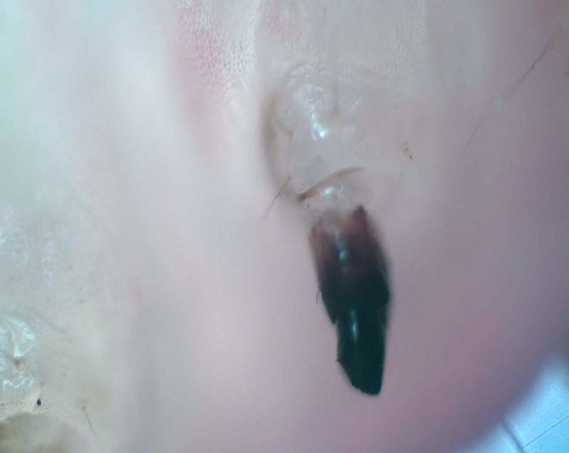


Fig. 1.1.5 Fig. 1.1.6


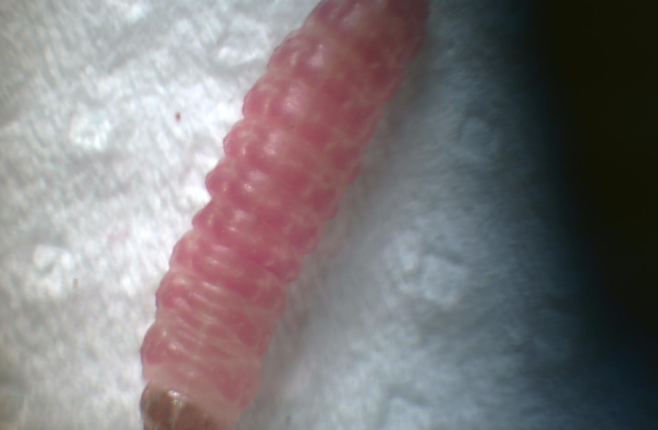

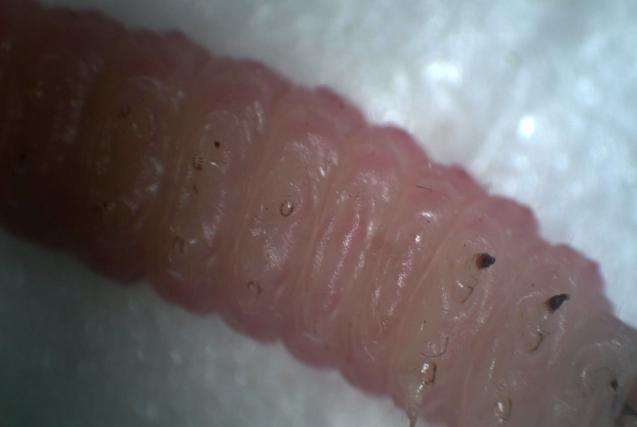


Fig. 1.1.7 Fig. 1.1.8

***Pectinophora gossypiella***

**Fig. 9-12 (pupa)**

*Pectinophora gossypiella* Saunders, 1843. Trans. Ent. Soc. London, 3: 284-285

Pupa is obtect or chrysalis, appendages are firmly glued to the body, Pupa is 9 to 11 mm, smooth body, and reddish brown color (Fig. 1.1.9).. Pupal eyes are visible, mostly 3 to 4 setae are visible on vertex. All thoracic and abdominal segments with small yellowish setae, pair of setae are present near the spiracles (Fig. 1.1.10)., mostly hooked like setae are present on 5^th^ to 9^th^ abdominal segment (Fig. 1.1.12)., slit like anal opening. 8 to 12 hooked like setae are present near anal opening, antenna is also glued and reaching at the tip of wings(Fig. 1.1.11).. Vertical suture is extending up to the end of third thoracic segment. First thoracic segment is short, second is broad and long, transverse or m shaped suture is present on second thoracic segment, 3^rd^ thoracic segment is long and extending up to the sides of 3^rd^ abdominal segment, first abdominal segment is shorter, 2^nd^, 3^rd^and 4^th^ abdominal segments are equal in length, 5^th^ to 10^th^ abdominal segments are narrowing towards end. All parts of antenna, labrum, front, clypus, gena, maxillary palpus, maxilla, three legs, wings and anal opening are visible.


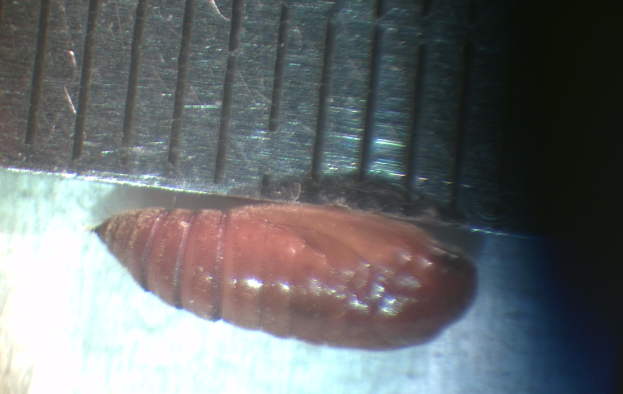

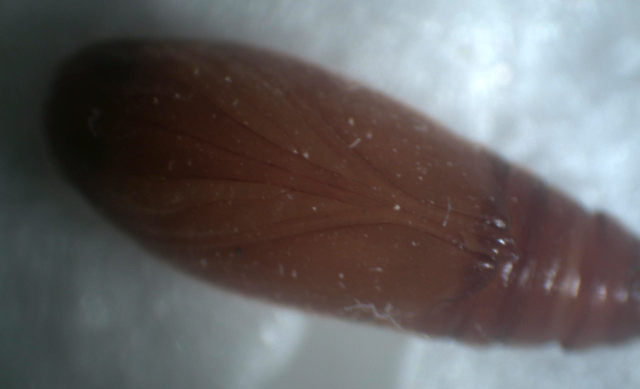


Fig.1.1.9 Fig.1.1.10


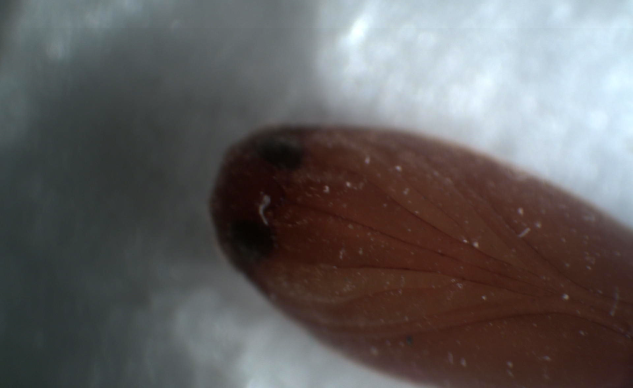

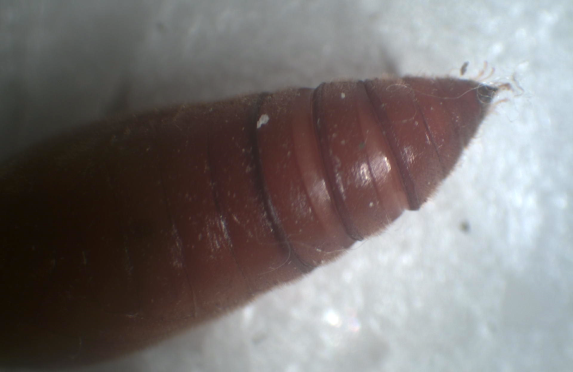


Fig.1.1.11 Fig.1.1.12

***Pectinophora gossypiella***

**Fig. 13-21(Adult)**

*Pectinophora gossypiella* Saunders, 1843. Trans. Ent. Soc. London, 3: 284-285

**Head:** Labial palpi is yellowish brown, two white patches are present near base, diffused black spots are absent, labial palpi upward curved, narrowing towards apex. Antenna is 4 to 5 mm in length, 0.1 mm wide, slightly narrowing towards apex, covered with scales, yellowish in color, pedicel with irregular black patch, 1^st^ segment of flagellum is more than half cover with black scales, black scales are not visible from segment no 2 to 5, reddish and yellowish scales are present, 6 to 7 blacks spots are present from segment no 6 to 11, 3^rd^ terminal segment is also with black spot. Eyes are visible and less than 1mm wide, slightly protruded from head. Head covered with black, brown and orange scales, white and black scales on posterior side of vertex forming two semi-circle shaped spots (Fig. 1.1.13).

**Thorax:** Reddish brown with sparkling of black and white scales, patagium (covers wing joint) are pad like and visible, basal portion is fully covered with black scales and rest with black and white scales (Fig. 1.1.14).

**Wings:** Front wing is 9-11 mm long**,** 2-3 mm wide, ovate, smooth and pointed tip, 12 longitudinal veins are present in front wing, costa is independent and terminating up to the half-length of front wing. Costa is branched at base, 10 veins are emerging from a longitudinal cell, last anal vein is highly bifurcate at base, apical portion with long, ocherous or blackish hairs, front wings darker brown with irregular ill-defined, black spots, scales on wings are slightly broader at top, basal portion densely dusted with black scales, middle portion is lighter in colour, apical portion again with black scale, Hind wings is broader than forewing, it is dark fuscous, iridescent, lightest towards base, apical portion is pointed with long hairs, 8 veins are present in hindwing, costa is highly deflected from the middle, subcosta and radius are almost fused at base, frenulum is visible, simple in males, frenulum triple in the females (Fig. 1.1.16).

**Legs:** Coxa with alternate bands of black and white scales, basal portion of femur is dusted with black scales, middle portion is lighter in col, hind portion is reddish or orange in color, tibia is long, reddish scales are present in wavy lines, long hairs are present at the junction of tibia and tarsal segments, tarsal segments with white strips (Fig. 1.1.17).

**Abdomen:** 6 to 7 mm long, 2 to 3 mm wideabdomen is flattened, laterally ocherous dark brown, underside suffused with black scales at the joints. Abdomen is similar in male and female, white scales are present between segments, segments are narrowing posteriorly. Female ovipositor is weakly chitinized, fully covered with stiff and hairs, genital plate is cordate shaped, male genitalia is small, aedeagus short, thick, terminal hook is visible (Fig. 1.1.18).

**Specimens collected from the cotton belt of Punjab tally with the description of Saunders (1843).**


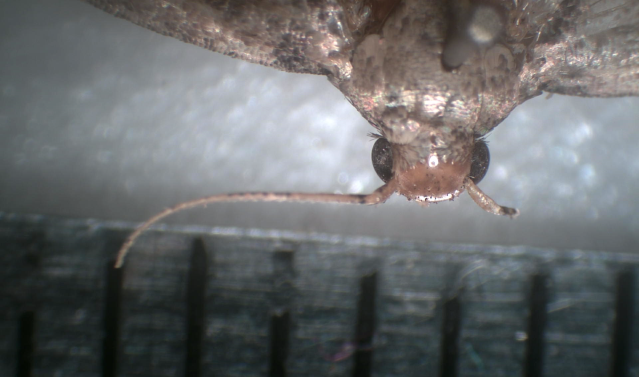

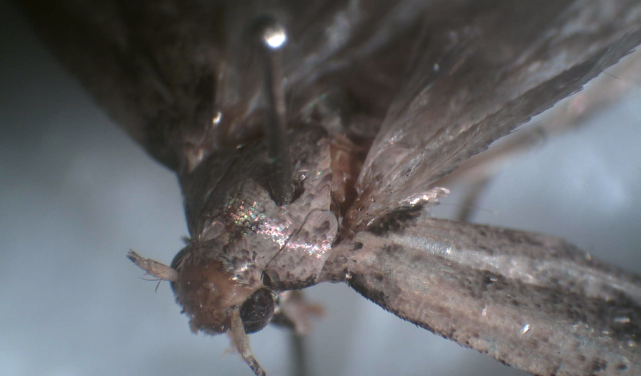


Fig. 1.1.13 Fig. 1.1.14


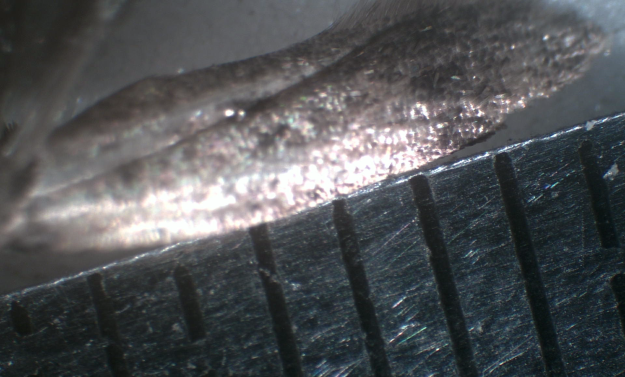
**
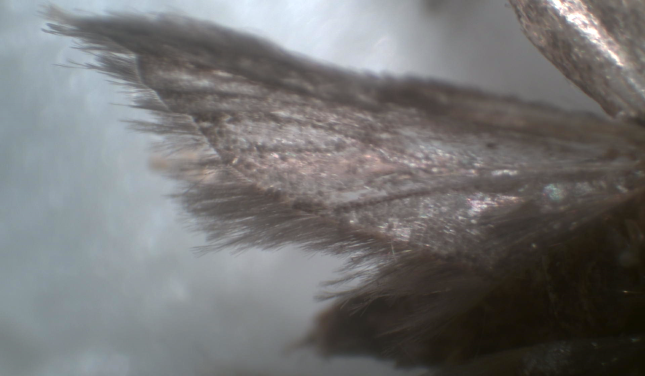
**

Fig. 1.1.15 Fig. 1.1.16

**
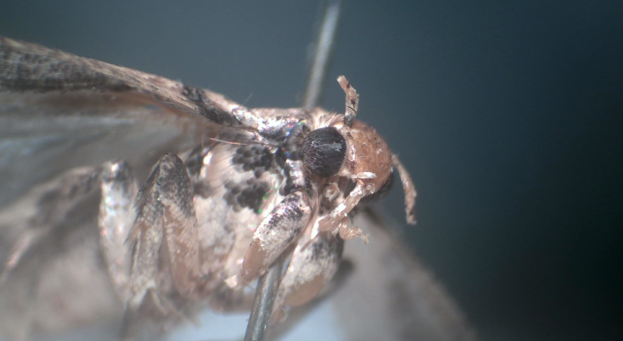
**
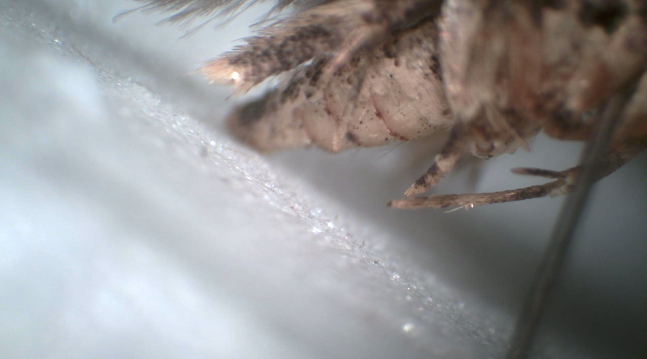


Fig. 1.1.17 Fig. 1.1.18


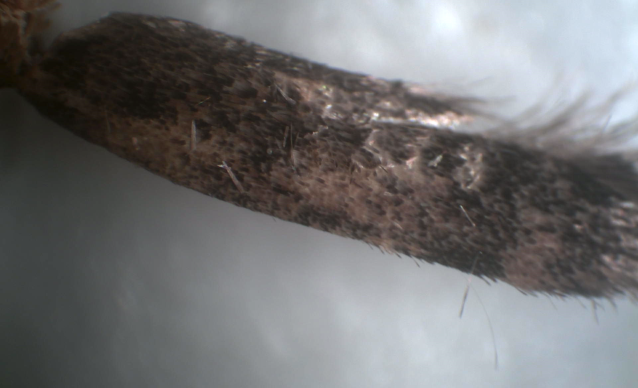

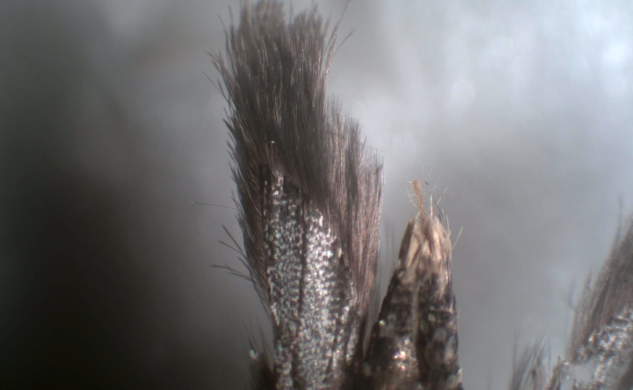


Fig. 1.1.19 Fig. 1.1.20


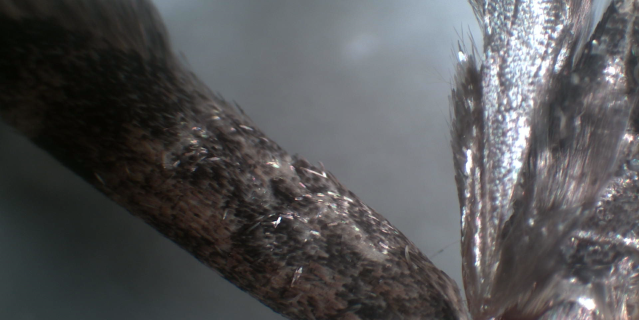


Fig. 1.1.21

**Material examined**

30 larvae, on Cotton, Faisalabad, 25-09-2019, M. Tayyib; 5 adults, on Cotton, Faisalabad, 25-09-2019, M. Tayyib; 11 larvae, on Cotton, Faisalabad, 10-10-2019, M. Tayyib; 3 adults, on Cotton, Faisalabad, 10-10-2019, M. Tayyib; 13 larvae, on Cotton, Jhang, 08-09-2019, M. Tayyib; 4 adults, on Cotton, Jhang, 08-09-2019, M. Tayyib; 8 larvae, on Cotton, Jhang, 15-09-2019, M. Tayyib; 13 adults, on Cotton, Jhang, 15-9-2019, M. Tayyib; 16 larvae, on Cotton, Jhang, 8-10-2019, M. Tayyib; 2 adults, on Cotton, Jhang, 08-10-2019, M. Tayyib; 7 larvae, on Cotton, Jhang, 15-10-2019, M. Tayyib; 4 adults, on Cotton, Jhang, 15-10-2019, M. Tayyib; 6 larvae, on Cotton, Bahawalpur, 22-08-2019, M. Tayyib; 4 adults, on Cotton, Bahawalpur, 22-08-2019, M. Tayyib; 1 larvae, on Cotton, Bahawalpur, 07-09-2019, M. Tayyib; 3 adults, on Cotton, Bahawalpur, 07-09-2019, M. Tayyib; 9 larvae, on Cotton, Bahawalpur, 22-09-2019, M. Tayyib; 3 adults, on Cotton, Bahawalpur, 22-09-2019, M.Tayyib; 7 larvae, on Cotton, Bahawalpur, 07-10-2019, M.Tayyib; 3 adults, on Cotton, Bahawalpur, 07-10-2019, M.Tayyib; 17 larvae, on Cotton, Bahawalpur, 22-10-2019, M. Tayyib; 6 adults, on Cotton, Bahawalpur, 22-10-2019, M. Tayyib; 12 larvae, on Cotton, Bhakkar, 18-09-2019, M. Tayyib; 6 adults, on Cotton, Baukkar, 18-09-2019, M. Tayyib; 2 larvae, on Cotton, Baukkar, 25-09-2019, M. Tayyib; 3 adults, on Cotton, Baukkar, 25-09-2019, M. Tayyib; 1 larvae, on Cotton, Baukkar, 02-10-2019, M.Tayyib; 5 adults, on Cotton, Baukkar, 02-10-2019, M. Tayyib; 2 larvae, on Cotton, Baukkar, 10-10-2019, M. Tayyib; 1 adults, on Cotton, Baukkar, 10-10-2019, M. Tayyib; 2 larvae, on Cotton, D.G. Khan, 15-10-2019, M. Tayyib; 1 adults, on Cotton, D.G. Khan, 15-10-2019, M. Tayyib; 12 larvae, on Cotton, D.G. Khan, 22-10-2019, M.Tayyib; 11 adults, on Cotton, D.G. Khan, 22-10-2019, M. Tayyib; 8 larvae, on Cotton, Rajanpur, 05-10-2019, M. Tayyib; 11 adults, on Cotton, Rajinpur, 05-10-2019, M. Tayyib; 4 larvae, on Cotton, Rajinpur, 18-10-2019, M. Tayyib; 11 adults, on Cotton, Rajinpur, 18-10-2019, M. Tayyib; 2 larvae, on Cotton, Rajinpur, 28-10-2019, M. Tayyib; 11 adults, on Cotton, Rajinpur, 28-10-2019, M. Tayyib; 2 larvae, on Cotton, Lodhran, 22-08-2019, M. Tayyib; 11 adults, on Cotton, Lodhran, 22-08-2019, M. Tayyib; 2 larvae, on Cotton, Lodhran, 07-09-2019, M. Tayyib; 1 adults, on Cotton, Lodhran, 07-09-2019, M. Tayyib; 12 larvae, on Cotton, Lodhran, 22-09-2019, M. Tayyib; 1 adults, on Cotton, Lodhran, 22-09-2019, M. Tayyib; 14 larvae, on Cotton, Multan, 02-09-2019, M. Tayyib; 12 adults, on Cotton, Multan, 02-09-2019, M. Tayyib; 1 larvae, on Cotton, Multan, 17-09-2019, M. Tayyib; 12 adults, on Cotton, Multan, 17-09-2019, M. Tayyib; 3 larvae, on Cotton, Multan, 02-10-2019, M. Tayyib; 1 adults, on Cotton, Multan, 02-10-2019, M. Tayyib; 13 larvae, on Cotton, Vehari, 16-09-2019, M. Tayyib; 1 adults, on Cotton, Vehari, 16-09-2019, M. Tayyib; 1 larvae, on Cotton, Vehari, 30-09-2019, M. Tayyib; 1 adults, on Cotton, Vehari, 30-09-2019, M. Tayyib; 7 larvae, on Cotton, Rahim Yar Khan, 16-10-2019, M. Tayyib; 6 adults, on Cotton, Rahim Yar Khan, 16-10-2019, M. Tayyib; 6 larvae, on Cotton, Rahim Yar Khan, 30-10-2019, M. Tayyib; 6 adults, on Cotton, Rahim Yar Khan, 30-10-2019, M. Tayyib;

**B. DNA-barcoding based identification and characterization of field collected *Pectinophora gossypiella* from cotton fields of Punjab, Pakistan**

**ABSTRACT**

*Pectinophora gossypiella* (Lepidoptera: Gelichidae) is monophagus and cause serious damage to cotton crops worldwide. Therefore, they have ability to survive on host plant in the ecosystem, because of their high movement, fecundity rate and capability for development of insecticides resistance against wide range of pesticides. In this study, by using mitochondrial cytochrome oxidase I (COI) gene, molecular identification and phylogenetic relationship of *pectinophora gossypiella* was studied. The total genomic DNA was extracted and PCR was done using COI based primer pairs. Amplified PCR products was purified and sequenced. The alignment of the PCR amplified DNA fragments from COI for various bollworms of cotton were performed through ClustalW. The maximum likelihood analysis was done using MEGA6 software. The result of PCR sequencing and phylogenetic analysis indicated that the studied *P. gossypiella* samples have 99-100% identity with NCBI submitted specimens reported from other countries.

**VI. Materials and Methods**

**Insect Culture**

The samples of cotton bollworms *Pectinophora gossypiella* were collected from different fields of cotton growing farmers of Punjab and research institutes fields, University of Agriculture Faisalabad (UAF), Ayyub Agricultural Research Institute (AARI), Faisalabad, Central Cotton Research Institute, Multan (CCRI), during Sep 2017-18 (Fig. 1.2.1). The transparent jars were utilized for their maintenance using fresh cotton leaves and bolls. Adult insects were examined for correct species identification.

**Molecular Study**

The DNAeasy DNA extraction Kit (Quiagen, Germany) or CTAB method was used to extract DNA from Meta legs of adult insects, according to DNA extraction method used by Fukova *et al.* (2008). The insects used for mtCO1 analysis was deposited at IGCDB laboratory. The extracted total genomic DNA was observed visually using 0.8 to 1% agarose gel. It will be quantified on picodrop using standard procedures of Nucleic acid quantification. DNA samples was diluted using ddH2O depending upon the required concentration, to get a working solution (10-30 ng/μL). A quantity of the total DNA was preserved in ten percent glycerol at -80 °C. The CO1 region was amplified using primers pairs LCO 1480 and HCO 1298 (Folmer *et al.* 1994). The PCR conditions for 25 μL reaction volume was (2.5 μL of 10 X PCR buffer) with 25 mM (2 μL) of MgCl2, (0.5 μL) of 10 mM dNTPs, (0.5 μL) each of forward and reverse primer, IU of TaqDNA polymerase, 17 μL of ddH2O (Invitrogen). The alignment of all sequences was done using BioEdit 4.0 program of using ClustalW 1.8 (Thompson *et al.* 1994) then, for sequences identity confirmation. The alignment was further studied employing MEGA 5.0. Phylogenetic analysis and diversity analysis for all sequences of targeted insectwas used. Then, sequences graphically were displayed by tree construction through Maximum Likelihood (ML) using program MEGA 5.05 (Tamura *et al.* 2011).

**RESULTS**

**Polymerase Chain Reaction Analysis (PCR)**

The amplification of DNA after genomic DNA confirmation was done from insects species using COI (F/R) based primers LCO-1490/ HCO-2198 and C1J2195/ TL2N3014 of cytochrome oxidase I (COI) gene. Total DNA samples were used during PCR amplification. The PCR reaction mixture up to 20 µl was prepared for PCR amplification and 30-50 ng quantity of each DNA sample was used for PCR amplification. Using COI primers, all DNA samples of *H. armigera* were amplified successfully and size was measured with 1 kb DNA ladder (GeneMark Company) on 2% agarose gel. The expected 710 bp DNA fragment was successfully visualized in 1.5% agarose gel as depicted in (Fig. 1.2.2). The amplified DNA fragments were eluted and then DNA fragments samples were sent to M/s. Macrogen Company (Korea) for the sequencing.

**Sequence alignment and phylogenetic analysis**

The nucleotide sequencing of DNA fragments obtained from M/s Macrogen (Korea) was (Seq>180319-003_O09-40 PBW1 LCO 1490 (Pak-Seq PBW**) (Fig. 1.29)** analyzed and aligned through BLASTn (Basic Local Alignment Search Tool) with sequence data *P. gossypiella* previously reported in NCBI (National Centre of Biotechnology Information) site. The reference accession numbers of sequences used for alignment based on LCO-1490/ HCO-2198 primers were as; *P. gossypiella* ([KM289071.1](https://www.ncbi.nlm.nih.gov/nucleotide/MG437191.1?report=genbank&log$=nucltop&blast_rank=1&RID=26R1G5BA015), [KX863147.1](https://www.ncbi.nlm.nih.gov/nucleotide/AB620129.1?report=genbank&log$=nucltop&blast_rank=10&RID=26R1G5BA015), [KF643170.1](https://www.ncbi.nlm.nih.gov/nucleotide/AB643667.1?report=genbank&log$=nucltop&blast_rank=24&RID=26R1G5BA015), KF 491994 Aand GQ853429.1), *P. endema* ([KF393092.1](https://www.ncbi.nlm.nih.gov/nucleotide/KJ930516.1?report=genbank&log$=nucltop&blast_rank=13&RID=26R1G5BA015)), *S. exigua* (KJ634295.1) *S. frugiperda* (KJ634298.1), *H. armigera* (KP210095.1) *S. litura* (KX863232.1, KX862420.1 and KF153858.1 ) as shown in **Fig. 1.30**.In base pair sequence alignment, homology of *P. gossypiella* was compared with previously reported NCBI database through BLAST option. Pairwise alignment of dendrogram tree indicated that our sample (Seq>180319-003_O09-40 PBW1 LCO 1490 (Pak-Seq PBW) being clubbed in same cluster as **(***P. gossypiella* ([KM289071.1](https://www.ncbi.nlm.nih.gov/nucleotide/MG437191.1?report=genbank&log$=nucltop&blast_rank=1&RID=26R1G5BA015), [KX863147.1](https://www.ncbi.nlm.nih.gov/nucleotide/AB620129.1?report=genbank&log$=nucltop&blast_rank=10&RID=26R1G5BA015), [KF643170.1](https://www.ncbi.nlm.nih.gov/nucleotide/AB643667.1?report=genbank&log$=nucltop&blast_rank=24&RID=26R1G5BA015), KF 491994.1) and GQ853429.1 as outgroup) with 99% with good query coverage 82-97%. The other insects indicated similarity of 96-90 % respectively and formed different clusters in phylogenetic tree (Fig. 1.31).


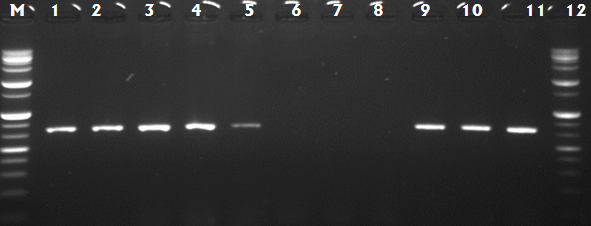


**Fig 1.29**: DNA amplification of *P. gossypiella* using mCOI (F/R) primers in PCR; Lane 6-8 (- control); Lanes 1-5, 9-12 DNA from *P. gossypiella of various regions of cotton zones*. M, 1 kb DNA ladder Marker (GeneMark)

**CATAAAGATATTGGAACTTTATACTTTATTTTTGGAATTTGAGCTGGAATAGTAGGTATATCTTTAAGTTTATTAATTCGTTTATTAATTCG**

**AGCTGAATTAGGTAACCCAGGATCTTTAATTGGTGATGATCAAATTTATAATACTATTGTCACTGCTCATGCTTTTATTA**

**AGCTGAATTAGGTAACCCAGGATCTTTAATTGGTGATGATCAAATTTATAATACTATTGTCACTGCTCATGCTTTTATTA**

**TAATTTTCTTCATAGTTATACCAATTATAATTGGAGGATTCGGAAATTGATTAGTACCTTTAATATTAGGAGCCCCTGAT**

**TAATTTTCTTCATAGTTATACCAATTATAATTGGAGGATTCGGAAATTGATTAGTACCTTTAATATTAGGAGCCCCTGAT**

**ATAGCTTTTCCTCGAATAAATAATATAAGTTTTTGACTTTTACCCCCCTCATTAACTCTTTTAATTTCAAGAAGAATTGT**

**ATAGCTTTTCCTCGAATAAATAATATAAGTTTTTGACTTTTACCCCCCTCATTAACTCTTTTAATTTCAAGAAGAATTGT**

**AGAAAATGGAGCAGGAACCGGATGAACAGTTTACCCCCCACTTTCATCTAATATTGCTCATGGAGGAAGTTCAGTAGATC**

**AGAAAATGGAGCAGGAACCGGATGAACAGTTTACCCCCCACTTTCATCTAATATTGCTCATGGAGGAAGTTCAGTAGATC**

**TGGCAATTTTTTCTTTACATTTAGCAGGTATTTCATCAATTTTAGGAGCAATTAACTTTATTACTACAATTATTAATATA**

**TGGCAATTTTTTCTTTACATTTAGCAGGTATTTCATCAATTTTAGGAGCAATTAACTTTATTACTACAATTATTAATATA**

**CGAATTAATGGTTTATCATTCGATCAAATACCATTATTTGTTTGAGCTGTAGGAATTACAGCCTTATTATTACTTTTATC**

**CGAATTAATGGTTTATCATTCGATCAAATACCATTATTTGTTTGAGCTGTAGGAATTACAGCCTTATTATTACTTTTATC**

**ATTACCTGTTTTAGCAGGAGCTATTACTATATTACTAACAGATCGAAATTTAAATACTTCATTTTTTGATCCAGCTGGTG**

**ATTACCTGTTTTAGCAGGAGCTATTACTATATTACTAACAGATCGAAATTTAAATACTTCATTTTTTGATCCAGCTGGTG**

**GAGGAGATCCAATCCTATACCAACACTTATTTTGATTTTTTGGTCACCCT**

**GAGGAGATCCAATCCTATACCAACACTT**

**Fig. 1.30.** Multiple alignments of DNA sequences through CLUSTAL OMEGA from DNA amplification of *P. gossypiella* (>180319-003_O09-43PBW (Pak-Seq PBW) with NCBI GenBank submitted sequences, (KM289071.1 PBW)


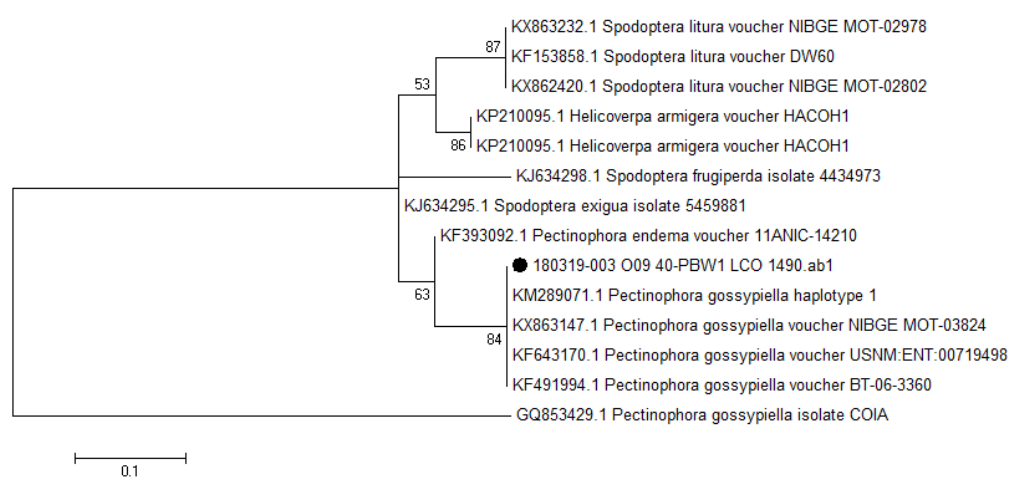


**Fig. 1.31.** Molecular phylogenetic analysis by Maximum Likelihood Method. Phylogenetic Tree produced from DNA amplification of *P. gossypiella* using mitochondrial Cytochrome oxidase I (mCOI) (F/R) gene based primers

**OBJECTIVE-2: MASS-REARING OF PBW**

- **ACTIVITY-1: FIELD COLLECTION OF ALL THE PEST STAGES AND REARING ON THE NATURAL DIET.**
- **ACTIVITY-2: LABORATORY REARING OF THE PBW LARVAE ON THE ARTIFICIAL DIETS AND CULTURING FOR CONTINUOUS INSECT POPULATIONS**

**MATERIALS AND METHODS**

For rearing and biology of pink bollworm *P. gossypiella,* a study was carried out under controlled conditions in Pink Bollworm Rearing Laboratory at Department of Entomology, University of Agriculture Faisalabad.

**Collection of pink bollworm adults**

For the establishment of pink bollworm culture, open cotton bolls containing pupa were collected during months of March to May from cotton field areas of UAF campus and then transferred to glass cages (60×60×60cm^3^ diameter) having one opening in front while ventilated squared windows covered with fine mesh at both side of cages for adult collection. Adult emergence cages (Fig. 2.1) were then maintained at laboratory conditions of 27±2°C temperature and 70 ± 10 % RH that facilitated pupal development to adult emergence.


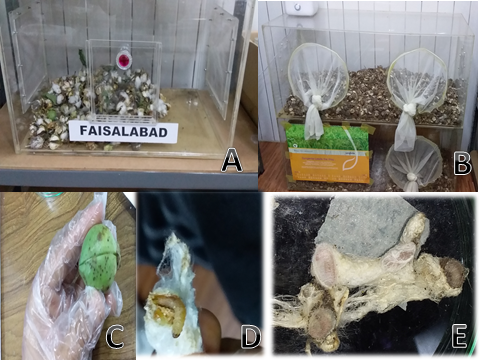
**Fig 2.1: A-B:** PBW moth collection cages. **C:** Infested cotton boll due to larval penetration of *P. gossypiella*. **D:** Larva feed inside on seed**. E:** Diapause larva

**Eggs collection and observation**

For mating, 10 pink bollworm pairs in 1:1 ratio of male and female were collected from glass cages with the help of polystyrene vials and released per egg laying glass chimney shield with black cloth maintained at 27±2°C temperature and 60 ± 10 % RH. For oviposition, each glass chimney at the top covered with white towel tissue paper as a substrate which was held in place with screen wire disc of 2-3 inches in diameter to ensure even contact over entire surface of egg laying substrate and to prevent adult escape. Each glass chimney was provided with glass vial stoppered with cotton soaked in a mixture of decavitamin drop (1ml) and 10% honey solution (water 90ml: 10ml honey) for adult diet. After 3 days of preoviposition period, ventral side of tissue paper in each chimney for egg collection was observed and replaced it with a new tissue. Changed adult diet after every two days for adult mortality reduced with the increased in fecundity followed by counted eggs and noted egg color. Collected eggs from each chimney till the egg deposition of pink bollworm was completed by the tenth day (Fig.2.2). **
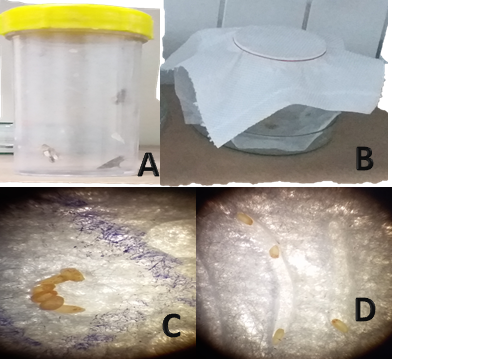
**

**Fig 2.2: A:** PBW moth collection vial. **B:** Egg laying glass chimney covered with oviposition substrate (groves). **C:** Eggs laid in batches. **D**: Singly egg laid pattern in groves in towel tissue.

After naked eye observation, eggs were examined under microscope to check any damage by predatory mites or might be parasitic wasps then before transferred tissue paper containing eggs into each transparent labelled plastic cup (25cm) for neonate culture, while removing egg predator and parasite with the camel hair brush to prevent further damage to eggs.

**Starter Culture**

In maintained laboratory conditions of temperature and relative humidity, eggs were hatched in 2-3 days. Before shifted neonate to rearing cups, noted hatchability percentage because many damaged eggs were not hatched that can effected larval culture. Plastic cups containing neonate were exposed to torch light because neonate attracted towards light that can facilitate their transferred to rearing cups (3.8×3.4×3cm^3^) using camel hair brush. Daily check egg hatchability to build-up larval culture for rearing and transferred hatched larvae to rearing cups so to reduced larval mortality (Fig.2.3)


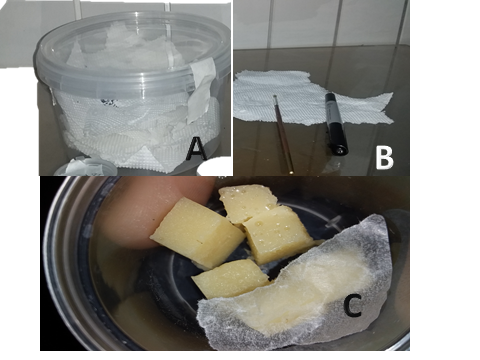


**Fig 2.3: A:** Plastic cup possessed many neonates. **B:** Shifting of neonates using camel hair brush. **C:** Rearing cup possessed larvae from starter culture

**Preparation of experimental diets**

Treatment diets including standard diet (wheat germ as a main ingredient), okra diet and chickpea diet symbolized as T1, T2 and T3 whereas each diet had different methodology of preparation according to their suggested formulations.

**Standard diet (wheat germ) preparation technique**

For successful rearing of pink bollworm larvae, required equipment was sterilized with 5% ethanol solution (25ml water) and autoclaved to avoid any contamination during diet preparation. Standard diet was prepared by using ingredients like wheat germ meal, casein, agar, sucrose, Brewer’s yeast, alpha cellulose, potassium sorbate, nipalgin, decavitamins, choline chloride, maize oil, honey and distilled water as suggested by Wu *et al.* (2008). Firstly all ingredients were accurately weighed using electronic balance according to Table-2.1.1. Three fractions (A, B and C) of ingredients were made so well mixed and product was obtained.

**Table 2.1.1: Composition of artificial standard medium with quantity**

| Components of Fraction A | Quantity (g.kg^-1^ or ml. L^-1^) |
| --- | --- |
| Wheat germ meal | 34.5g |
| Casein | 30.0g |
| Sucrose | 10.0g |
| Brewer’s yeast | 5.0g |
| Alpha-cellulose | 1.0g |
| Potassium sorbate | 1.5g |
| Nipalgin | 0.5g |
| Choline chloride | 0.06g |
| Maize oil | 3.3g |
| Honey | 2.0g |
| Distilled Water | 230ml |
| Components of Fraction B (Decavitamins: 0.01ml) | Quantity (mg.ml^-1^) |
| Calcium pentothenate | 0.12mg |
| Niacin | 0.06mg |
| Riboflavin | 0.03mg |
| Folic acid | 0.03mg |
| Thiamine | 0.015mg |
| Pyridoxine hydrochloride | 0.015mg |
| Biotin | 0.0093mg |
| Vitamin B_12_ | 0.00012mg |
| Components of Fraction C | Quantity(g.kg^-1^ or ml. L^-1^) |
| Agar-agar | 20.0g |
| Distilled Water | 500ml |

Firstly, fraction A ingredients were stirred well in 230 ml of distilled water in a 1000 ml of measuring beaker. Then fraction B comprised of decavitamins (0.0l ml) separately mixed in 10 ml of water in a measuring cylinder to make vitamin solution and fraction C comprised of agar as a thickening agent separately well stirred in 500ml of distilled water in a 1000 ml of measured beaker.

After making fraction solutions, firstly added fraction A solution into blender so all ingredients well mixed followed by blended 3.3 ml corn oil and 2 ml honey into a blender mixture then fraction B contain vitamin mixture added into fraction A with continuous blending. Fraction C containing agar was boiled in an oven at 30 sec interval with continued stirred for 25 mints until beads started to form and color change of agar with elevated viscosity that indicate uniform mixture was obtained. In last step dissolved thick agar was added into fraction A with continuous blending and entire mixture blend for about 2 minutes until homogenous color was obtained.


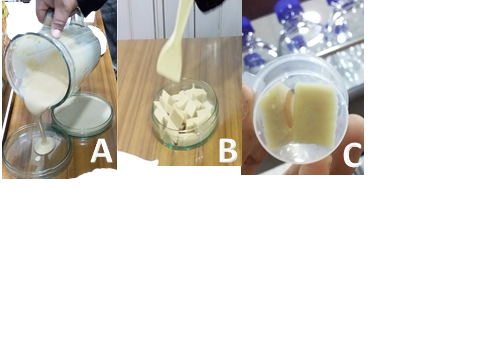


**Fig 2.4: A:** Poured hot wheat germ diet into glass petri dishes. **B:** Cut diet with spatula into cubes. **C:** Diet cubes possessed reared larva.

After blending, hot mixture poured into transparent glass petri dishes (150 mm ×15mm), allowed to cool for 10 minutes before use. Soon after pouring hot medium into petri dishes, petri dishes covered with brown wrapping paper to prevent contamination with microorganisms and allowed successive moisture to evaporate. Cut medium with spatula into small cubes of ¼ inches in size and placed 3-4 diet cubes in each transparent plastic cups for rearing of pink bollworm larvae at laboratory controlled conditions of 27±2°C temperature and 60 ± 10 % relative humidity (Fig. 2.4).

**Okra diet preparation technique**

Collected fresh okra fruits from different field areas of UAF campus followed by transferred to PBW rearing laboratory for pink bollworm rearing purpose. Firstly, washed okra fruit with distilled water proceeded to drying then cut 15cm okra with cutter into 2.5cm okra pieces and placed 5 okra pieces in each labelled transparent plastic cup for larval rearing (Fig.2.5) under laboratory controlled conditions of 27±2°C temperature and 50 ± 10 % relative humidity. Larvae were fed with soft and fresh pods of okra pieces under controlled conditions. Changed okra pieces for every 3 days to prevent fungal contamination.


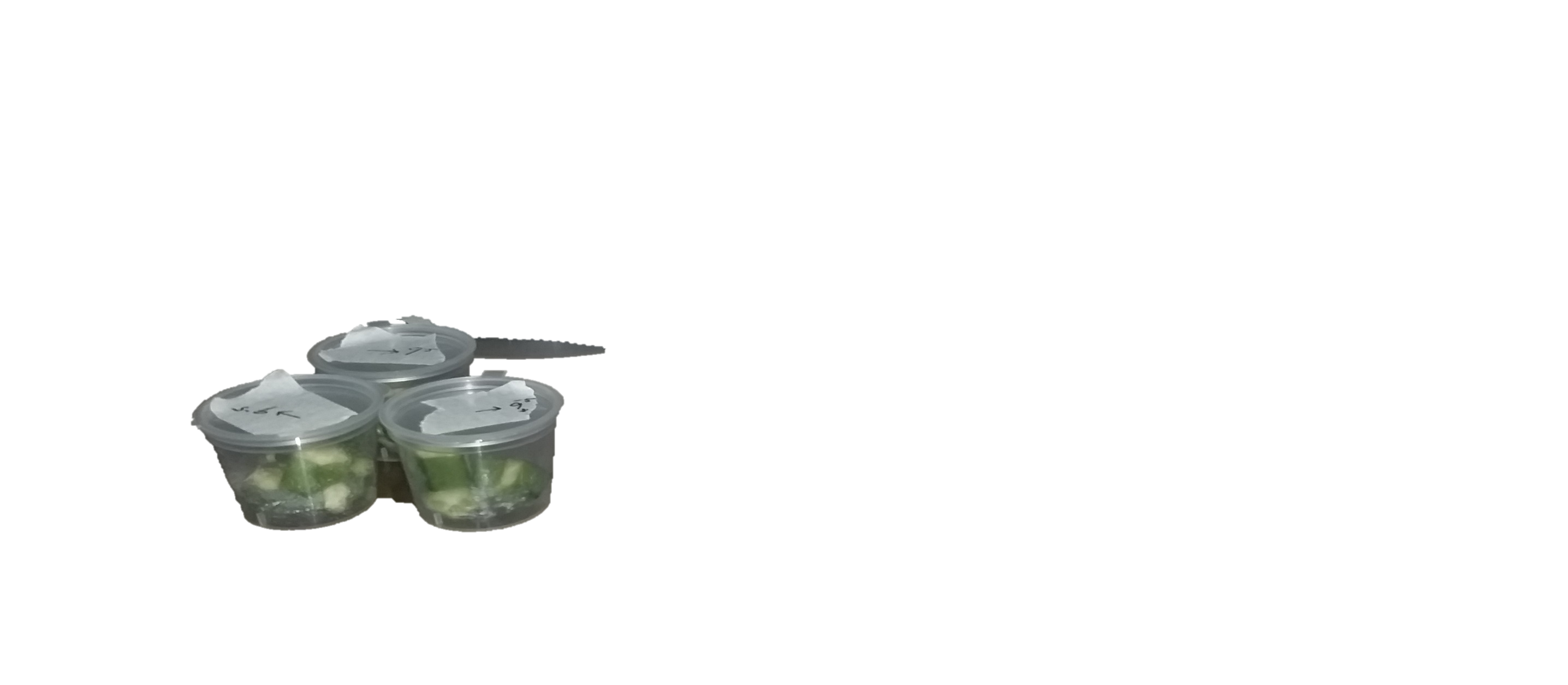


**Fig 2.5:** Rearing cups possessed okra diet pieces for larval rearing

**Chickpea diet preparation technique**

Chickpea diet ingredients were same as developed by Dhara Jothi *et al.*, 2016 for successful laboratory rearing of pink bollworm larva. Primary constituent of diet was chickpea in fraction A with sucrose while agar as thickening agent constituent of fraction B and other ingredients comprised of carbohydrate, protein, fat, multivitamins, anti-microbial agents in fraction C. All ingredients were weighed accurately on electronic balanced in controlled conditions (Table 2.1.2).

Firstly, mixed fraction A ingredients in 200ml ml of distilled water, warm to 60°C with continuous stirred proceeded to cool then added dissolved solution into blender and mixed thoroughly. Then fraction B comprised agar was boiled in 200 ml of distilled water in an oven followed by continued stirred until beading consistency obtained then blended dissolved viscous agar into fraction A. Finally, fraction C ingredients were added into fraction A mixture with continuous blending until homogenous mixture obtained.

At the end, prepared diet was poured into glass petri dishes to a depth of 2cm and allowed to solidify (Fig.2.6) under controlled conditions of 27±2°C temperature and 30 ± 10 % relative humidity followed by sliced diet with spatula into 2cm × 0.2cm × 0.5cm small cubes for pink bollworm rearing.

**Table 2.1.2: Ingredients of chickpea medium along with their quantity**

| Components of Fraction A | Quantity (g. ml^-1^) |
| --- | --- |
| Chick pea flour | 35 |
| Sucrose | 15 |
| Distilled Water | 200 |
| Components of Fraction B | |
| Agar-agar | 19 |
| Distilled Water | 200 |
| Components of Fraction C | |
| Dried yeast powder | 8.0 |
| Ascorbic acid | 1.2 |
| Methyl 4-hydoxy benzoate | 1.6 |
| Multivitamin solution | 1.0 |
| Streptomycin sulphate | 0.2 |
| Bavistin | 2.0 |
| Casein | 10 |
| Cystiene | 0.1 |
| Wesson’s salt | 2.5 |
| Sorbic acid | 0.5 |
| Cholesterol | 0.5 |


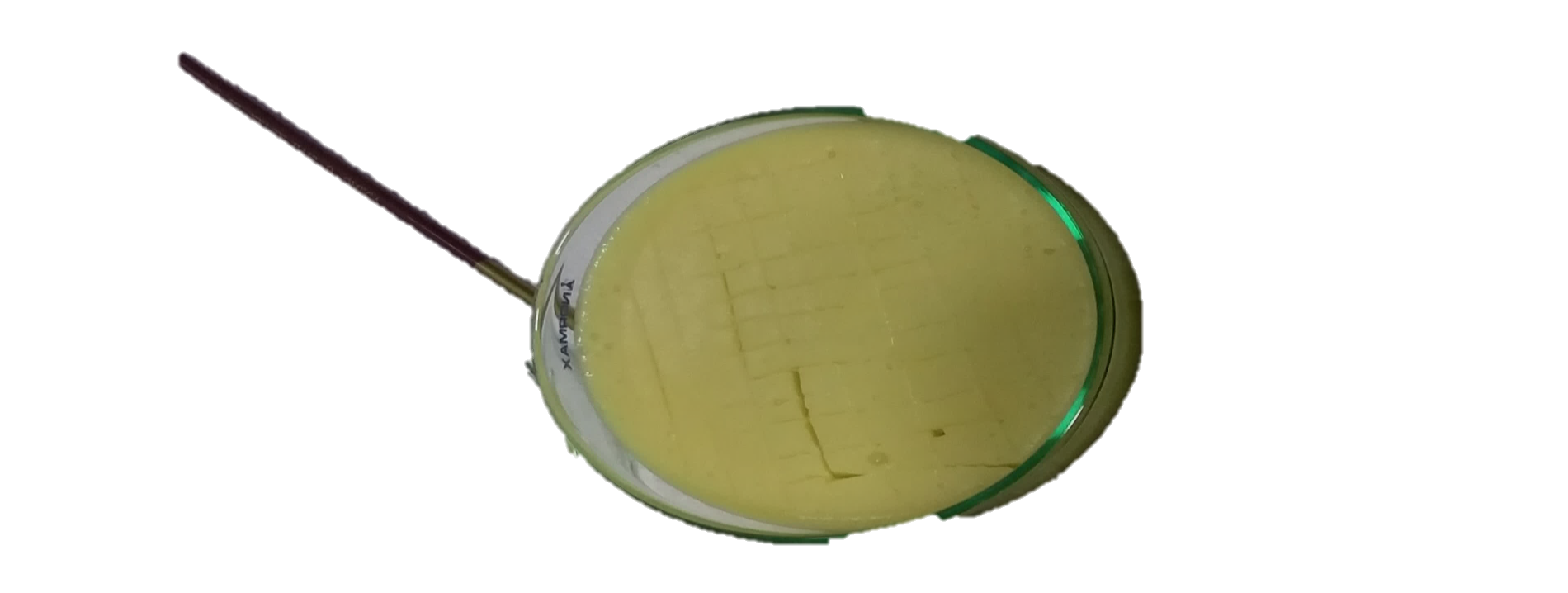


**Fig 2.6:** Prepared chickpea diet solidfied

**Experimental layout**

Experiment was laid down in completely randomized design comprised of three treatment diets while each treatment replicated 10 times (Fig. 2.7) possessed two larvae per replication (60 observations). In second experiment, each treatment replicated 6 times according to their respective relative humidity percent to study their effects on some humidity dependent parameters of pink bollworm. In third experiment, Kept 5 replications of each treatment diet for studied their effects on weight parameter of insect.


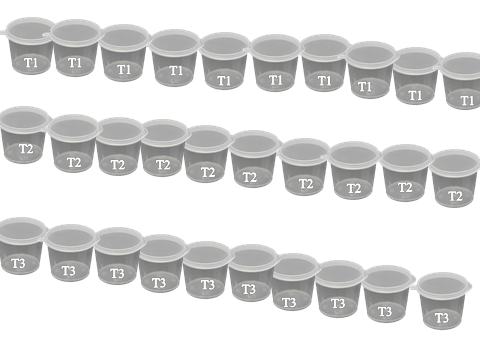


**Fig 2.7:** CRD layout in experiment No. 1

**Rearing procedure**

After obtaining successful culture of neonates, there was a need to shift them on prepared diet for successful rearing. For rearing purpose, different types of containers (Fig. 2.8) were used including glass vials, 24-well plates, waxed paper cups and small sized plastic cups but successful rearing was proceeded in small sized plastic cups while different problems faced during rearing in other containers due to various reasons like difficulty in handling neonates in vials, larval escape from wells of 24-well plate and diet contamination in wax paper cups.

The labelled transparent plastic cups were used as rearing container at pink bollworm rearing laboratory under controlled conditions of temperature and relative humidity followed by used camel’s hair brush for transferred two neonates per rearing cup with the closed lid of cup to prevent larval escape, diet contamination and predatory or parasitic entry into rearing cup which can caused larval mortality.


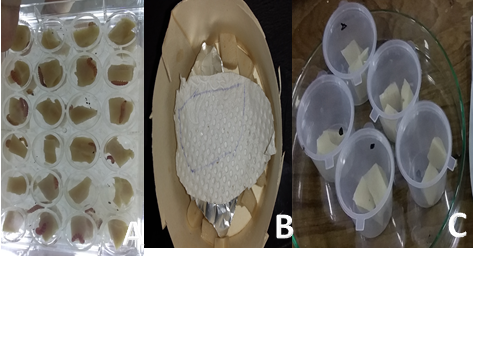


**Fig 2.8: Larval Rearing methods; A:** 24-well culture plate. **B:** waxed paper rearing cup comprised of aluminium sheet to prevent dry out of diet **C:** small sized labelled plastic cup for rearing

For successful larval development, larvae shifted on fresh diet that change every third day. Daily observed larval stage and increased diet according to larval stage. When larvae at fourth stage then separate them as male and female for adult paring.

After larvae pupate, transferred matured dark brown pupa in wide-mouthed specimen jars separately labeled as male and female for adult emergence and collection. After pupation period, released emerged adult in pairs with polystyrene vials to medium sized round glass chimneys for matting and egg laid purpose (Fig.2.9).


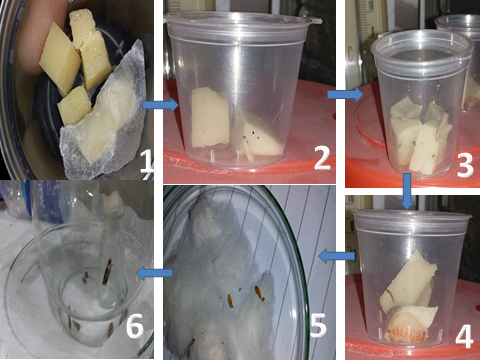


**Fig 2.9**: Neonate larva shifted on diet **(1).** Second instar larva feed on prepared diet **(2).** Third instar **(3).** Fully grown fourth instar ready to pre-pupate **(4).** Male and female dark brown pupae **(5)** Emerged PBW adult (**6)**

Wide-mouthed round glass chimneys used because prevented excessive flight activity, crowding of adults, helps to preserve scales and facilitate mating among adults

**Problems in rearing**

Main problem during rearing procedure was contamination of larval diet due to fluctuated laboratory conditions of temperature and relative humidity that effected larval development period, pupal emergence, mating, fecundity and egg hatching to larval percentage. Other than these problems, specific difficulty facing was egg damaged by predatory and parasitic attacked of mites and wasps. These problems can be minimized if maintaining aseptic and controlled laboratory conditions with daily removal of predatory mites and parasitic wasps resulted in normal larval development. If one wanted to completely eliminate these problems, then complete handling of diet from preparation to placing in cups should be mechanically done with the exception of handling of egg laying culture.

**Observations**

Observations on biological parameters including incubation period, larval period, pupal period, larval weight, pupal weight, egg hatching into larval percentage, adult emergence percentage, sex ratio, fecundity, pre-oviposition period, post-oviposition period, adult longevity and adult total life span were recorded.

**Statistical analysis**

Observations on biological parameters including incubation period, larval period, pupal period, larval weight, pupal weight, egg hatching into larval percentage, adult emergence percentage, sex ratio, fecundity, pre-oviposition period, post-oviposition period, adult longevity and adult total life span were subjected to ANOVA technique using statistics 8.1 software. The means of significant treatments were compared by Tukey’s HSD test.

**RESULTS**

**Developmental period of pink bollworm reared on three treatment diets**

Growth and development of pink bollworm reared on different treatment diets.

**Incubation period**

Statistical analysis indicated that incubation period showed significant difference among treatment diets. According to table 4.40, egg hatching period was significantly higher on standard diet (5.3a) which differ non-significantly from egg hatching period on chickpea diet (4.8a). Incubation period on okra diet (2.9b) significantly differed from incubation period on wheat germ and chickpea (P=0.05).

**Table 2.1.3:** ANOVA parameters for effect of treatment diets on incubation period (days) of *P. gossypiella*

| **Larval Diets** | **Incubation period ( Mean±SE days)** |
| --- | --- |
| **Wheat germ diet** | 5.3±0.2134a |
| **Okra diet** | 2.9±0.2769b |
| **Chickpea diet** | 4.8±0.2494a |
| **CVC** | 0.8696 |

**Table 2.1.4 Incubation period of pink bollworm on three treatment diets**

| Source of Variation  (S. O. V) | DF | SS | MS | F-Value |
| --- | --- | --- | --- | --- |
| **Treatments** | 2 | 32. 0667 | 16. 0333 | 26.1^**^ |
| **Error** | 27 | 16. 6000 | 0. 6148 |  |
| **Total** | 29 | 48. 6667 |  |  |
| **** Highly significance difference at 1% probability level** | | | | |

**Mean instar period**

First instar period of *P. gossypiella* differ significantly (P<0.05) across three treatment diets which had high significant effect on first larval stage of pink bollworm (P=0.000). Instar period vary depend on the type of diet on which larvae reared. Instar period of newly hatched larvae reared on standard-diet, okra-diet and chickpea-diet was 3.6±0.163, 3.4±0.163, and 2.40±0.163days, respectively. A shortest instar period of newly hatched larvae was observed on chickpea-diet followed by okra-diet. Longest instar period of neonate was recorded on standard-diet.

Table 2.1.3 indicated second instar period of *P. gossypiella* differ significantly (*P*<0.05) across treatment-diets. Three treatment diets had significant effect on second larval stage of pink bollworm (*P*=0.0156) Second instar duration of larvae reared on wheat germ diet varied from 4.7000±0.2603days while ranged from 3.5000±0.2236 days when reared on okra diet. Instar period of second stage larvae ranged from 4.3000±0.3350days when reared on chickpea diet which was in accordance with the Zinzuvadiya *et al.*, 2017 findings on chickpea diet who recorded it 4.31±0.76days. Noted shortest second instar period of larvae reared on okra diet followed by larvae reared on chickpea diet and longest second instar duration observed on wheat germ diet (Fig.2.1-2.5).

Table 2.1.7 indicated that third instar period of PBW differed significantly (P<0.05) across treatment-diets. Three treatment diets had high significant effect on second larval stage of pink bollworm (P=0.0004). Third instar period varied from 4.9000±0.2769days when reared on wheat germ diet, 3.5000±0.1667days when reared on okra diet while 4.6000±0.2211days on chickpea diet which was in agreement with the Zinzuvadiya *et al.*, 2017 results. Shortest third instar duration was observed on okra diet followed by chick pea diet and longest instar period noted on wheat germ diet (Fig.2.1- 2.5).

Fourth instar period of male and female pink bollworm differed significantly (P<0.05) across treatment. The diets had high significant effect on fourth larval stage of male and female pink bollworm (P=0.000). Male fourth instar duration observed on wheat germ diet varied form 6.2000±0.1333days, on okra diet varied from 3.7000±0.2603 days while on chickpea diet varied from 6.2000±0.2494 days which were synchronism with Zinzuvadiya *et al.*, 2017 observations who reported male instar duration of 6.40±0.52 days. Shortest period of male fourth instar was observed on okra diet followed by wheat germ diet and longest instar period noted on chickpea diet (Fig.2.1.25). Female fourth instar period observed on wheat germ diet varied form 8.4000±0.1633day while instar duration observed on okra diet varied from 4.2000±0.2906 days and on chickpea diet varied from 8.3000±0.1528 days. Present findings of female instar period on three different diets were in contrast with the results recorded by Zinzuvadiya *et al.,* 2017 who reported it 5.60±0.68 days on chickpea diet. Observed shortest period of female fourth instar on okra diet followed by chickpea die and longest instar period noted on wheat germ diet (Fig.2.12.5).

**Table 2.1.5: ANOVA parameters for effect of treatment diets on 1^st^ instar period (days) of *P. gossypiella***

| Source of Variation | DF | SS | MS | F-Value |
| --- | --- | --- | --- | --- |
| **Treatments** | 2 | 8.2667 | 4. 13333 | 15.5^**^ |
| **Error** | 27 | 7.2000 | 0.26667 |  |
| **Total** | 29 | 15. 4667 |  |  |
| **** Highly significance difference at 1% probability level** | | | | |

**Table 2.1.6: ANOVA parameters for effect of treatment diets on 2^nd^ instar period (days) of *P. gossypiella***

| Source of Variation | DF | SS | MS | F-Value |
| --- | --- | --- | --- | --- |
| **Treatments** | 2 | 7.4667 | 3. 73333 | 4.87^**^ |
| **Error** | 27 | 20. 7000 | 0.76667 |  |
| **Total** | 29 | 28. 1667 |  |  |
| **** Highly significance difference at1% probability level** | | | | |

**Table 2.1.7: ANOVA parameters for effect of treatment diets on 3^rd^ instar period (days) of *P. gossypiella***

| Source of Variation | DF | SS | MS | F-Value |
| --- | --- | --- | --- | --- |
| **Treatments** | 2 | 10.8667 | 5.43333 | 10.6^**^ |
| **Error** | 27 | 13. 8000 | 0.51111 |  |
| **Total** | 29 | 24. 6667 |  |  |
| **** Highly significance difference at 1% probability level** | | | | |

**Table 2.1.8: ANOVA parameters for effect of treatment diets on male 4^rth^ instar period (days) of *P. gossypiella***

| Source of Variation | DF | SS | MS | F-Value |
| --- | --- | --- | --- | --- |
| **Treatments** | 2 | 41. 6667 | 20. 8333 | 42.3^**^ |
| **Error** | 27 | 13. 3000 | 0.4926 |  |
| **Total** | 29 | 54. 9667 |  |  |
| **** Highly significance difference at 1% probability level** | | | | |

**Table 2.1.9: ANOVA parameters for effect of treatment diets on female 4^rth^ instar period (days) of *P. gossypiella***

| Source of Variation | DF | SS | MS | F-Value |
| --- | --- | --- | --- | --- |
| **Treatments** | 2 | 114. 867 | 57. 4333 | 128^**^ |
| **Error** | 27 | 12. 100 | 0.4481 |  |
| **Total** | 29 | 126. 967 |  |  |
| **** Highly significance difference at 1% probability level** | | | | |

**Table 2.1.10: Longevity (days) of different instars of *P. gossypiella* reared on different diets**

| **Diets** | **Mean±SE**  **1^st^ Instar(days)** | **Mean±SE**  **2^nd^ Instar(days)** | **Mean±SE**  **3^rd^ Instar(days)** | **Mean±SE**  **4^rth^ Male Instar(days)** | **Mean±SE**  **4^rth^ Female Instar(days)** |
| --- | --- | --- | --- | --- | --- |
| **Wheat germ diet** | 3.6000±0.1633a | 4.7000±0.2603a | 4.9000±0.2769a | 6.2000±0.1333a | 8.4000±0.1633a |
| **Okra diet** | 3.4000±0.1633a | 3.5000±0.2236b | 3.5000±0.1667b | 3.7000±0.2603b | 4.2000±0.2906b |
| **Chickpea diet** | 2.4000±0.1633b | 4.3000±0.3350ab | 4.6000±0.2211a | 6.2000±0.2494a | 8.3000±0.1528a |
| **CVC** | 0.5727 | 0.9710 | 0.7929 | 0.7784 | 0.7424 |

**Average total larval period**

Male (Table 2.1.9) and female (Table 2.1.10) larval period demonstrate significant difference (P<0.05) across different treatment diets indicating that different treatment diets had higher significant effect on PBW male larval period (P=0.000).After the mean instar period completed, recorded total larval period as male and female by separately rearing them on treatment diets. Female larval period is longer than male larval period. Total larval developmental period (Table 2.1.4) of male and female larva varied from 19.400±0.4522days and 21.600±0.4989days when separately reared on wheat germ diet. Male larval period varied from 14.000±0.3651days and female larval period varied from 14.500±0.5217days on okra diet. Male larval period varied from 17.500±0.6368 days. which was in confirmation with Zinzuvadiya *et al.,* 2017 reported 17.5±1.95 days while female larval period varied from 19.600±0.7483 days which was in contrast with the Zinzuvadiya *et al.,* 2017 recorded 8.15±2.18 days when both sexes separately rearing on chickpea diet. In present results, larval period recorded on chickpea diet was in conformation with Malthankar and Gujar (2014) results recorded total larval period range from 18.26-18.96 days on seed powder of cotton cultivars.

Present findings of larval period on three different diets were in contrast with the earlier studies of Cacayorin *et al.,*1992 reported 11.33±0.64 days; Vennila *et al.*, 2007 recorded 9 to14 days in hotter region; Shah *et al.,* 2013 noted 9 days at 35± 1ºC and 13 days at 27± 1 ºC as well as Dharajothi *et al.*, 2016 who found it to be 25.10± 0.994 days when reared on artificial medium.

Shortest larval period of male and female observed on okra diet which was in contrast with the Muralimohan *et al.*, 2009 who reported shortest instar period of 21.34±2.61 days on two phase diet (cotton seed flour and okra) followed by intermediate larval period of both sexes on chickpea diet and longest larval period observed when both sexes reared separately on wheat germ diet (Fig. 2.10).

**Table 2.1.11: ANOVA parameters for effect of treatment diets on male larval period (days) of *P. gossypiella***

| Source of Variation | DF | SS | MS | F-Value |
| --- | --- | --- | --- | --- |
| **Treatments** | 2 | 150. 067 | 75. 0333 | 30.3^**^ |
| **Error** | 27 | 69. 900 | 2. 4778 |  |
| **Total** | 29 | 216. 967 |  |  |
| **** Highly significance difference at 1% probability level** | | | | |

**Table 2.1.12: ANOVA parameters for effect of treatment diets on female larval period (days) of *P. gossypiella***

| Source of Variation | DF | SS | MS | F-Value |
| --- | --- | --- | --- | --- |
| **Treatments** | 2 | 268. 067 | 134. 033 | 37.2^**^ |
| **Error** | 27 | 97. 300 | 3. 604 |  |
| **Total** | 29 | 365. 367 |  |  |
| **** Highly significance difference at 1% probability level** | | | | |

**Figure 2.10:** Male and female larval duration of PBW reared at three different artificial diets. Bars represent larval period.

**Mean pupal period**

Pupal period of *P. gossypiella* differed significantly (P<0.05) across treatment diets which showed that three treatment diets had significant effect on pupal period (P=0.0261) (Table 2.1.13). After the completion of larval development, fourth instar larva of pink bollworm turns into pre-pupal stage. Prepupal stage short that turns into resting stage called pupal stage in which developmental structures formed. Pupal period varied depend on type of diet on which larvae reared. Recorded from table 2.1.14, pupal duration on standard diet recorded 8.6000±0.4000 days which was in agreement with Shah *et al.*, 2013 results reported 8 days pupal period at 35± 1ºC and also slightly in accord with Adkisson *et al.,* 1960 findings reported 8.8 days on wheat germ diet. Pupal period varied from 7.7000±0.2603 days when larvae reared on okra diet. Pupal period from larvae reared on chickpea diet varied from 7.3000±0.3000 days which was in accordance with the Zinzuvadiya *et al.*, 2017 findings on chickpea die and more or less in agreement with Cacayorin *et al.,* 1992 who found pupal period of 7.42± 0.20 days.

In contrast to present findings, El-Syed (1960) recorded pupal period as 16.7 days at 25ºC, Henneberry and Clayton (1986) found 3.5 days pupal delayed at varied temperature of 18 to 35ºC in comparison to development noted at constant temperature of 26º C, Muralimohan *et al.*, 2009 reported shortest pupal period of 7.96± 1.37 days when larvae reared on two phase diet (cotton seed flour and okra), Dharajothi *et al.*, 2016 recorded pupal period of 7.9± 0.88 days when reared on artificial medium, Malthankar and Gujar (2014) noted pupal period range from 5.76-6.48 days when artificially reared larva on seed powder of cotton cultivars.

Shortest pupal period observed in larvae reared on chickpea diet followed by okra diet and pupation delay observed in larvae reared on wheat germ diet but present results were in contrast with the Muralimohan *et al.*, 2009 findings who found shortest pupal period of 7.96± 1.37 days on two phase diet.

**Table 2.1.13: ANOVA parameters for effect of treatment diets on pupal period (days) of *P. Gossypiella***

| Source of Variation | DF | SS | MS | F-Value |
| --- | --- | --- | --- | --- |
| **Treatments** | 2 | 8.8667 | 4.43333 | 4.19^**^ |
| **Error** | 27 | 28. 6000 | 1. 05926 |  |
| **Total** | 29 | 37. 4667 |  |  |
| **** Highly significance difference at 1% probability level** | | | | |

**Table 2.1.14: Pupae period of pink bollworm on different treatment diets**

| Diet | Pupae period ( Mean±SE days) |
| --- | --- |
| Wheat germ | 8.6000±0.4000a |
| Okra | 7.7000±0.2603ab |
| Chickpea | 7.3000±0.3000b |
| **CVC** | 1.1414 |

Means within column sharing same letters are not significantly different at p= 0.05, CVC: Critical value for comparisons.

**Mean longevity of pink bollworm adult**

Male (Table 2.1.15) and female (Table 2.1.16) life span differ significantly (P<0.05) across treatment diets which explain that three diets had high significant effect on male longevity (P=0.000).

Longevity of mated male and female *P. gossypiella* moth depends on type of diet on which larvae reared. Generally, male life span shorter than female life span. Recorded from Table 2.1.17, longevity of male adult from larvae reared on control diet varied from 19.200±0.3266 days whereas longevity of female adult on standard diet varied from 21.000±0.6667days. Male and female life span varied from 15.200±0.7860days and 18.500±0.8596days when reared on okra diet. Female life span on wheat germ and okra diet was in agreement with the Malthankar and Gujar (2014) results who reported 21.42± 0.43 days (AKA-8) and 18.500± 0.38 days (Pusa,1752) life span on cotton cultivars.

Average duration of life span of male and female moth varied from 7.8000±0.4899 days and 12.100±0.8226days when reared from chickpea diet was in agreement with the findings of Zinzuvadiya *et al.*, 2017 reported 7.70± 2.11 days and 13.70± 2.16 days of male and female life span when reared on chickpea diet; in accord with the longevity recorded by Shah *et al.*, 2013 as 10 and 12 days of male and female moth at 35± 1ºC; in accordance with Cacayorin *et al.,* 1992 findings who reported female longevity of 11.70± 0.48 days. In contrast to present findings, Adkission (1960) reported female longevity of 15.4 to 17.2 days when reared from various diets.

Minimum life span of male and female moth observed on okra diet followed by chickpea diet. Maximum longevity of male and female moth observed on wheat germ diet was equivalent with Malthankar and Gujar (2014) conclusions noted 21.42± 0.43 days (AKA-8) life span.

**Table 2.1.15: ANOVA parameters for effect of treatment diets on male longevity period (days) of *P. gossypiella***

| Source of Variation | DF | SS | MS | F-Value |
| --- | --- | --- | --- | --- |
| **Treatments** | 2 | 669. 067 | 334. 533 | 104^**^ |
| **Error** | 27 | 86. 800 | 3. 215 |  |
| **Total** | 29 | 755. 867 |  |  |
| **** Highly significance difference at 1% probability level** | | | | |

**Table 2.1.16: ANOVA parameters for effect of treatment diets on female longevity period (days) of *P. gossypiella***

| Source of Variation | DF | SS | MS | F-Value |
| --- | --- | --- | --- | --- |
| **Treatments** | 2 | 421. 400 | 210.700 | 34.0^**^ |
| **Error** | 27 | 167. 400 | 6.200 |  |
| **Total** | 29 | 588.800 |  |  |
| **** Highly significance difference at 1% probability level** | | | | |

**Table 2.1.17: Adult lifespan of pink bollworm on three treatment diets**

| **Diet** | **Male longevity( Mean±SE days)** | **Female longevity( Mean±SE days)** |
| --- | --- | --- |
| **Wheat germ diet** | 19.200±0.3266a | 21.000±0.6667a |
| **Okra diet** | 15.200±0.7860b | 18.500±0.8596a |
| **Chickpea diet** | 7.8000±0.4899c | 12.100±0.8226b |
| **CVC** | 1.9884 | 2.7614 |

Means within column sharing same letters are not significantly different at p= 0.05, CVC: Critical value for comparisons.

**Mean life cycle period of male and female pink bollworm adult**

Male (Table 2.1.18) and female (Table 2.1.19) life cycle period showed significant difference (P<0.05) across treatment diets which explained that three diets had high significant effect on male total life cycle (P=0.000).

The average life cycle period of female and male from egg to adult varied from 56.500±1.2042days and 52.500±0.8333days when reared on standard diet (Table 2.1.20) but only female total ;life span was in agreement while male life cycle period was deviated from Zinzuvadiya *et al.*, 2017 findings who reported 56.30± 9.84 and 38.40± 4.48 days on chickpea diet. Records indicate that female live longer than male. Developmental period of female and male from egg to adult varied from 43.600±1.2401 days and 39.800±1.2092 days on okra diet which was in confirmation with Gebremedhin and Tadesse (1974) conclusions who reported mean generation time from egg to egg as 37.8± 3.8 days. Total life span of female and male moth varied from 43.900±1.5948days and 37.500±1.1279 days on chickpea diet which was in accordance with Vennila *et al.*, 2007 results who noted female developmental period of 21 to 43 days. Present findings was contrary to earlier studies of Noble (1969) who described total life cycle period of 25-30 days and Shah *et al.*, 2013 reported 30-32 days at 35± 1º C

Observed shortest and fastest life cycle period of female adult on okra diet comparable to those reared on chickpea diet. Fast total life cycle period of male adult observed on chickpea diet followed by okra diet. In case of both sexes, longest and slowest life cycle period observed on standard diet.

| Source of Variation | DF | SS | MS | F-Value |
| --- | --- | --- | --- | --- |
| **Treatments** | 2 | 1305. 27 | 652.633 | 57.1^**^ |
| **Error** | 27 | 308. 60 | 11. 430 |  |
| **Total** | 29 | 1613. 87 |  |  |
| **** Highly significance difference at 1% probability level** | | | | |

**Table 2.1.18: ANOVA parameters for effect of treatment diets on male life cycle period (days) of *P. gossypiella***

**Table 1.1.19: ANOVA parameters for effect of treatment diets on female life cycle (days) of *P. gossypiella***

| Source of Variation | DF | SS | MS | F-Value |
| --- | --- | --- | --- | --- |
| **Treatments** | 2 | 1084. 20 | 542. 100 | 29.4^**^ |
| **Error** | 27 | 497. 80 | 18. 437 |  |
| **Total** | 29 | 1582. 00 |  |  |
| **** Highly significance difference at 1% probability level** | | | | |

**Table 1.1.20: Total life span of pink bollworm adults on three treatment diets**

| **Diet** | **Mean±SE Female life cycle (days)** | **Mean±SE Male life cycle (days)** |
| --- | --- | --- |
| **Wheat germ diet** | 56.500±1.2042a | 52.500±0.8333a |
| **Okra diet** | 43.600±1.2401b | 39.800±1.2092b |
| **Chickpea diet** | 43.900±1.5948b | 37.500±1.1279b |
| **CVC** | 3.7493 | 4.7619 |

Means within column sharing same letters are not significantly different at p= 0.05, CVC: Critical value for comparisons.

**4.4 Mean male and female number of pink bollworm on three treatment diets**

Male number (Table 2.1.21) and female number (Table 2.1.22) of PBW did not differ significantly across three treatment diets which showed that diets had no significant effect on male and female sex number (P>0.05). Sex ratio of male: female of *P.gossypiella* vary depend on type of diet on which larvae reared. Average male and female number (Table 2.1.23) from larvae reared on standard diet varied from 8.7000±0.6675 and 11.300±0.6675. Mean male and female sex number on okra diet observed to be 7.5000±0.8975 and 12.500±0.9458 while on chickpea diet varied from 7.2000±0.7272 and 12.800±0.7272. Present findings were in conformity with Zinzuvadiya *et al.,*2017 findings while in contrast to Dharajothi *et al.,* 2016 findings who reported 1:2 sex ratio of male to female.

Less male number observed on chickpea diet followed by okra diet. Less female number of *P.gossypiella* observed on okra diet followed by chickpea diet. Higher male and female number observed on wheat germ diet (Fig. 2.1112).

**Table 2.1.21: ANOVA parameters for effect of treatment diets on male number of PBW**

| Source of Variation | DF | SS | MS | F-Value |
| --- | --- | --- | --- | --- |
| **Treatments** | 2 | 12. 600 | 6.30000 | 1.06^NS^ |
| **Error** | 27 | 160.200 | 5.93333 |  |
| **Total** | 29 | 172. 800 |  |  |
| **NS: no significant difference** | | | | |

**Table 2.1.22: ANOVA parameters for effect of treatment diets on female number of PBW**

| Source of Variation | DF | SS | MS | F-Value |
| --- | --- | --- | --- | --- |
| **Treatments** | 2 | 12. 600 | 6.30000 | 1.01^NS^ |
| **Error** | 27 | 168. 200 | 6.22963 |  |
| **Total** | 29 | 180. 800 |  |  |
| **NS: no significant difference** | | | | |

**Table 2.1.23: Sex number of pink bollworm on different treatment diets**

| **Diet** | **Number of Male produced (Mean±SE)** | **Number of Female produced (Mean±SE)** |
| --- | --- | --- |
| **Wheat germ diet** | 8.7000±0.6675a | 11.300±0.6675a |
| **Okra diet** | 7.5000±0.8975a | 12.500±0.9458a |
| **Chickpea diet** | 7.2000±0.7272a | 12.800±0.7272a |
| **CVC** | 2.7014 | 2.7680 |

Means within column sharing same letters are not significantly different at p= 0.05, CVC: Critical value for comparisons.

**Mean fecundity of pink bollworm on three treatment diets**

Ten pairs of freshly emerged adult moths confined in each oviposition chimneys to find out fecundity of female moth. Fecundity of female moth from larvae reared on standard diet observed 98.200±12.433 (Table 2.1.24) was in correspondence with Adkisson (1961) results described 98.1 eggs of pink bollworm when reared on cotton squares. Fecundity was 77.100±8.7247 eggs per female when reared on okra diet. Female reared on chickpea diet laid 110.70±10.883 eggs was in agreement with Zinzuvadiya *et al.,* 2017 findings who reported 110± 39.75 fecundity of female and Shah *et al* (2013) found 100-200 egg number. Fecundity noted on three diets were correspondence with the Malthankar and Gujar (2014) results who noted 70-110 no of eggs on seed powder of cotton cultivars.

**Table 2.1.24: Fecundity of pink bollworm on different treatment diets**

| **Diet** | **Fecundity Mean±SE** |
| --- | --- |
| **Wheat germ diet** | 98.200±12.433a |
| **Okra diet** | 77.100±8.7247a |
| **Chickpea diet** | 110.70±10.883a |
| **CVC** | 37.833 |

Means within column sharing same letters are not significantly different at p= 0.05. CVC: Critical value for comparisons

In contrast to present findings, El-Sayed (1960) recorded 224 eggs at 25ºC when rearing larvae on natural diet; Adkisson (1961) recorded 204.3 eggs from adults reared on cotton bolls, 336.7 eggs from moths reared on 1% cotton seed meal diet, 302.1 eggs when reared on 5% cotton seed meal diet while noted 312.2 eggs from wheat germ diet; Cacayorin *et al.,* 1992 recoded it to be 70 eggs while Attique (2004) noted 225 eggs in 9 days of oviposition period.

Oviposition records indicate (Table 2.1.24) that female reared on chickpea diet laid more numbers of eggs than reared on standard diet. Observed less number of eggs laid by female reared on okra diet.

**Mean larval and pupal weight of pink bollworm on different treatment diets**

Table 4.17 indicated effect of treatment diet on larval weight of pink bollworm and found that larval weight were not differ significantly when reared on three treatment diets shows that three diets had no significant effect on larval weight of PBW (P>0.05). Table 4.18 indicated effect of treatment diet on pupal weight of pink bollworm and found that pupal weight that is the indicator of food conversion during larval stages differ significantly across the treatment diets so treatments effect highly significant on pupal weight of *P. gossypiella* (P<0.05) which was inconsistent with the results recorded by Muralimohan *et al* .,2009 as pupal weight was significantly differed on cotton seed flour + Okra, cotton seed flour + chickpea flour and southland pink bollworm diet premix.

Pupal weight is an indicator of food conversion efficiency during larval stages. According to table 4.10, larval and pupal weight of *P.gossypiella* reared on standard diet varied from 17.600±0.5099 mg and 21.200±0.3742 mg, on okra diet varied from 19.600±0.5099 mg and 16.400±0.2449 mg. Larval and pupal weight of *Pectinophora gossypiella* reared on chickpea diet range from 19.400±0.7483 mg and 17.400±0.7483 mg.

Present findings on standard diet were in contrast with the Dharajothi *et al.,* 2017 results while larval and pupal weight on okra and chickpea diet were in conformation with Dharajothi *et al.*, 2017 findings who reported 21.40± 3.63 and 18.00± 2.73mg weight. Larval weight observed highest on okra and chickpea diet followed by wheat germ diet. Pupal weight observed highest on standard diet followed by okra diet and chickpea diet (Fig. 2.12).

**Table 2.1.25: ANOVA parameters for effect of treatment diets on pink bollworm larval weight**

| Source of Variation (S. O. V) | DF | SS | MS | F-Value |
| --- | --- | --- | --- | --- |
| **Treatments** | 2 | 12.1333 | 6.06667 | 3.37^NS^ |
| **Error** | 12 | 21.6000 | 1.8000 |  |
| **Total** | 14 | 33.7333 |  |  |
| **NS: no significant difference** | | | | |

| Diet | Larval weight (Mean±SE mg) | Pupal weight (Mean±SE mg) |
| --- | --- | --- |
| **Wheat germ diet** | 17.600±0.5099a | 21.200±0.3742a |
| **Okra diet** | 19.600±0.5099a | 16.400±0.2449b |
| **Chickpea diet** | 19.400±0.7483a | 17.400±0.7483b |
| **CVC** | 2.2700 | 1.9042 |

**Table 2.1.26: ANOVA parameters for effect of treatment diets on pink bollworm pupal**

| Source of Variation (S. O. V) | DF | SS | MS | F-Value |
| --- | --- | --- | --- | --- |
| **Treatments** | 2 | 64.1333 | 32. 0667 | 25.3****** |
| **Error** | 12 | 15. 2000 | 1.2667 |  |
| **Total** | 14 | 79. 3333 |  |  |
| **** Highly significance difference at 1% probability level** | | | | |

**Table 2.1.27: Larval and pupae weight of pink bollworm on different treatment diet**

Means within column sharing same letters are not significantly different at p= 0.05, CVC: Critical value for comparisons.

**Biological parameters of pink bollworm reared on treatment diets with their specific relative humidity percentage**

Because of every diet have its own relative humidity levels and pink bollworm show better growth on treatment diet of specific relative humidity levels.

**4.7.1 Mean fecundity of pink bollworm on treatment diets of their respective relative humidity percentage**

Fecundity of female moth depends on type of diet on which adult reared, age of insect and humidity levels (Table 2.1.28). Mean fecundity of female moth of *P.gossypiella* was 87.167±15.875 eggs when reared on standard diet at 60-70% relative humidity percentage. Female produced 76.167±12.729 eggs when reared on okra diet at 50-60%. Mean fecundity was 103.67±14.317 eggs per female when reared on chickpea diet at 40-50% relative humidity (Table 2.1.29).

Highest number of eggs laid by female moth when larvae reared on chickpea diet at 40-50% relative humidity followed by fecundity of female moth reared on standard diet at 60-70% RH and less number of eggs laid by female reared on okra diet at 50-60% RH (Table 2.1.29).

**Table 2.1.28: ANOVA parameters for effect of treatment diets on fecundity of PBW**

| Source of Variation | DF | SS | MS | F-Value |
| --- | --- | --- | --- | --- |
| **Treatments** | 2 | 2299.0 | 1149.50 | 0.93^NS^ |
| **Error** | 15 | 18571.0 | 1238.07 |  |
| **Total** | 17 | 20870.0 |  |  |
| **NS: no significant difference** | | | | |

**Table 2.1.29: Fecundity of pink bollworm female moth on three treatment diets at certain relative humidity percentage**

| **Diet** | **Fecundity Mean±SE** |
| --- | --- |
| **Wheat germ diet** | 87.167±15.875a |
| **Okra diet** | 76.167±12.729a |
| **Chickpea diet** | 103.67±14.317a |
| **CVC** | 37.833 |

Means within column sharing same letters are not significantly different at p= 0.05. CVC: Critical value for comparisons.

**Mean egg hatchability to adult emergence percentage of pink bollworm on different treatment diets of their respective relative humidity percent**

Table 2.1.30 show egg hatchability percentage differ highly significantly (P<0.05) across treatment diets which indicate that diets had high significant effects on egg hatching (P= 0.0015). Larval survival percentage during instar period indicates significant difference (P<0.05) across treatment diets which explain that diets had significant effect on larval percentage of PBW (P=0. 0128) (Table 2.1.31). Adult emergence percentage showed non-significant difference (P>0.05) across three treatment diets and diets had non-significant effect on adult emergence percentage of PBW (P=0.0647) (Table 2.1.32). Egg hatchability, larval survival and adult emergence percentage depend on the type of diet on which larvae rear, humidity percentage and temperature. From egg hatchability to adult percentage records (Table 2.1.33) the mean egg hatchability percentage of female moth was 91.852±1.9181 when reared on standard diet at 60-70% relative humidity level. Egg to hatching percentage was 93.219±1.3301 when female moth reared on okra diet at 50-60% RH was in agreement with Dharajothi *et al.*, 2016 conclusions reported 95.56% egg hatchability of moth reared on chickpea diet. Average egg hatchability percentage was 78.601±3.6646 when female moth reared on chickpea diet at 40-50% relative humidity level. Egg hatchability per cent on standard and chickpea diet was similar with Malthankar and Gujar (2014) result who recorded 67.84± ±22.66 (G-27) and 60.41± 18.94(Gh-BHV-824) per cent on cotton cultivars.

Observed maximum number of eggs hatched into neonate when female moth reared on okra diet at 50-60% relative humidity levels followed by standard diet (60-70%RH) and less eggs hatchability percentage observed on chickpea diet (40-50%RH) match with Malthankar and Gujar(2014) results recorded lowest egg hatchability percent of 60.41± 18.94(Gh-BHV-824).

Larval survival during instar period was measured in percentage depend on moisture and vitamin content of diet and humidity levels in laboratory controlled conditions. Average of 93.134±2.0242 percentage of eggs originally hatched into larvae when reared on standard diet at 60-70% of RH. Average percentage of eggs survive into larvae was 98.066±1.0185 when pink bollworm adult reared on okra diet at 50-60% RH. Mean egg hatchability to larval percentage on chickpea diet was recorded 90.881±1.3114. Present findings were dissimilar with Shah et al (2013) outcomes discovered 69.5% survival of larvae reared on natural diet of cotton bolls, squares and flowers but larval recovery per cent noted on wheat germ diet was in agreement with the Dharajothi *et al.*, 2017 reported 95.56 % larval survival on chickpea diet. Malthankar and Gujar (2014) findings about larval survival per cent (35.65-40.98%) on cotton cultivars were contrast with present results. Maximum larval survival percentage recoded on okra diet at 50-60% RH followed by standard diet (60-70%RH), compared to reared on chickpea diet (40-50%RH) on which lower survival percentage of larvae was recorded.

**Table 2.1.30: ANOVA parameters for effect of treatment diets on egg hatchability per cent of *P. gossypiella***

| Source of Variation | DF | SS | MS | F-value |
| --- | --- | --- | --- | --- |
| **Treatments** | 2 | 782.23 | 391.117 | 10.4** |
| **Error** | 15 | 566.32 | 37.755 |  |
| **Total** | 17 | 1348.55 |  |  |
| **** Highly significance difference at 1% probability level** | | | | |

**Table 2.1.31: ANOVA parameters for effect of treatment diets on larval survival per cent of PBW**

| Source of Variation | DF | SS | MS | F-value |
| --- | --- | --- | --- | --- |
| **Treatments** | 2 | 162.021 | 81. 0106 | 5.91^*^ |
| **Error** | 15 | 205.636 | 13. 7091 |  |
| **Total** | 17 | 367. 657 |  |  |
| *** Significance difference at 1% probability level** | | | | |

**Table 2.1.32: ANOVA parameters for effect of treatment diets on adult emergence per cent of PBW**

| Source of Variation | DF | SS | MS | F-value |
| --- | --- | --- | --- | --- |
| **Treatments** | 2 | 186.758 | 93. 3790 | 3.30^NS^ |
| **Error** | 15 | 423. 910 | 28. 2607 |  |
| **Total** | 17 | 610. 668 |  |  |
| **NS: no significant difference** | | | | |

**Table 2.1.33: Egg hatchability to adult emergence percentage of pink bollworm on different treatment diets at certain relative humdity percent**

| **Diet** | **Mean±SE**  **Hatchability %** | **Mean±SE**  **Larval survival %** | **Mean±SE**  **Adult emergence %** |
| --- | --- | --- | --- |
| **Wheat germ diet** | 91.852±1.9181a | 93.134±2.0242ab | 95.542±1.1334a |
| **Okra diet** | 93.219±1.3301a | 98.066±1.0185a | 99.537±0.4630a |
| **Chickpea diet** | 78.601±3.6646b | 90.881±1.3114b | 91.647±3.5541a |
| **CVC** | 9.2186 | 5.555 | 7.9757 |

Emergence of adult from pupa depend on fat content in diet as more fat content in diet delay pupation and emergence. Average 95.542±1.1334 percentage of larvae grows into adult when reared on standard diet of 60-70% RH was in correspondence with the Dharajothi *et al.* (2016) results recorded 95.56% adult emergence on chickpea diet. Originality of 99.537±0.4630 percentage of larvae emerged into adult when reared on okra diet at 50-60% RH. Mean adult emergence percentage was 91.647±3.5541 when larvae reared on chickpea diet at 40-50% of RH was interrelated with adult emergence of 91.66% recoded by Muralimohan *et al.,* 2009 when larvae reared on two phase diet.

Maximum adult emergence was recorded on okra diet (50-60% RH) which was similar with Muralimohan *et al.* (2009) outcomes who found maximum adult emergence (91.66%) on two phase diet followed by standard diet (60-70% RH) compared with a chickpea diet (40-50%RH) on which less percentage of larvae originally transform into adult.

**Mean pre-oviposition and oviposition period of pink bollworm on three treatment diets at their respective relative humidity percentage**

Mating and egg laying period of *P. gossypiella* depend on temperature, photoperiod, humidity levels, larval and adult diet. It was found that pre-oviposition period differed significantly (P<0.05) across three treatment diets which showed that diets had high significant effect (P=0.0043) on pre-oviposition period of PBW (Table 2.1.34). Whereas, oviposition period showed no significant difference (P>0.05) across three treatment diets which indicate that diets had non-significant effect on oviposition period of pink bollworm (P=0.3734) (Table 2.1.35). According to table 2.1.36, average pre-oviposition and oviposition period of pink bollworm adult range from 4±0.2582 and 8.6667±0.3333days when reared on standard diet. Pre-oviposition and oviposition period of *Pectinophora gossypiella* on okra diet was found 3.1667±0.3073 and 8.6667±0.3333 days, respectively. Mean pre-oviposition and oviposition period of pink bollworm adult reared on chickpea diet was 2.5±0.2236 and 8±0.4472 days, respectively which was in agreement with Zinzuvadiya *et al.,* 2017 described preoviposition and oviposition period of 2.91±0.70 and 8.00±1.54days o chickpea diet.

**Table 2.1.34: ANOVA parameters for effect of treatment diets on pre-oviposition period (days) of *P. gossypiella***

| Source of Variation | DF | SS | MS | F-value |
| --- | --- | --- | --- | --- |
| **Treatments** | 2 | 6.7778 | 3.38889 | 8.03^**^ |
| **Error** | 15 | 6.3333 | 0.42222 |  |
| **Total** | 17 | 13.1111 |  |  |
| **** Highly significance difference at 1% probability level** | | | | |

**Table 2.1.35: ANOVA parameters for effect of treatment diets on oviposition period (days) of PBW**

| Source of Variation | DF | SS | MS | F-value |
| --- | --- | --- | --- | --- |
| **Treatments** | 2 | 1.7778 | 0.8889 | 1.05^NS^ |
| **Error** | 15 | 12.6667 | 0.84444 |  |
| **Total** | 17 | 14.4444 |  |  |
| **NS: no significant difference** | | | | |

**Table 2.1.36: Preoviposition period and Oviposition period of pink bollworm on different treatment diets at certain relative humdity percent**

| **Diets** | **Preoviposition period**  **(Mean±SE days)** | **Oviposition period**  **( Mean±SE days)** |
| --- | --- | --- |
| **Wheat germ diet** | 4±0.2582a | 8.6667±0.3333a |
| **Okra diet** | 3.1667±0.3073ab | 8.6667±0.3333a |
| **Chickpea diet** | 2.5±0.2236b | 8±0.4472a |
| **CVC** | 0.9749 | 1.3787 |

Means within column sharing same letters are not significantly different at p= 0.05, CVC: Critical value for comparisons

**ACTIVITY-3: EVALUATION OF DIFFERENT COLORS AND TEXTURES FOR OVIPOSITION PREFERENCE IN PINK BOLLWORM**

**Experiment #1**

In the first experiment rough texture tissue papers of different colors (Green, Red, White) were used as egg receptacles. In this experiment three replications were used. The colored tissue-papers were placed as a lid of the jar because adults mothfly upward and lay eggs. Cotton soaked with 5% honey solution was kept inside the jar for feeding of pink bollworm. Iron net was used to restrict the adults to come outside from the jar. Egg receptacles changed on daily basis to check oviposition preference. Ten adult pairs were collected from the cages and then transferred into the plastic jars and glass light chimneys to check the oviposition preference of PBW.

**Experiment # 2**

In the second experiment, same above mentioned process was repeated but by using different egg receptacles. White colored artificial substrates e.g., (Nappy liner, Gouache paper and “Vinda Kitchen Towel super absorbent”) as shown in figures 2.11 and 2.11, of the same colour but of different texture with and without cotton leaves and its extracts were used ( Figure 2.11 & 2.11).


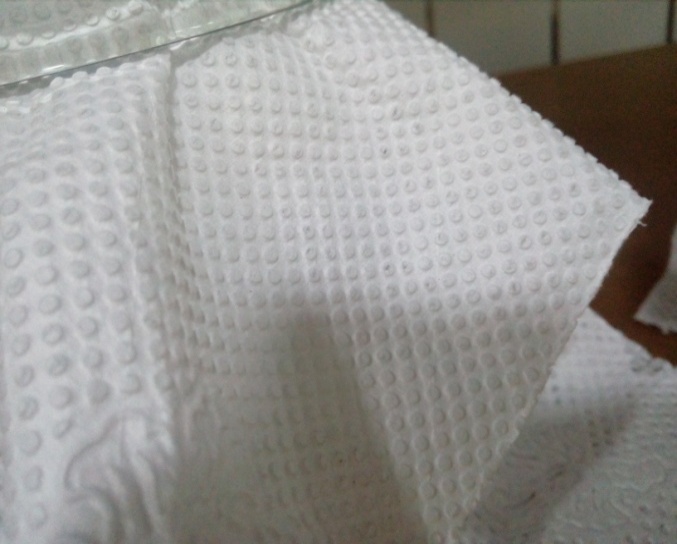

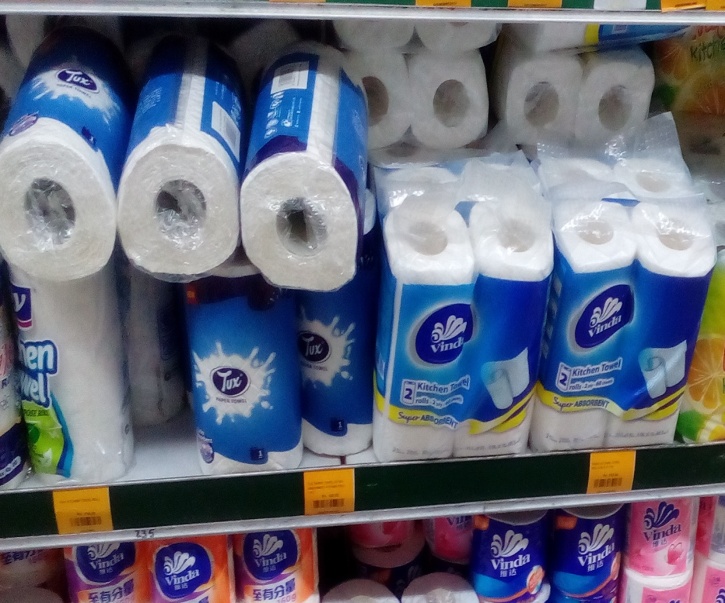


**Fig. 2.11:** Texture # 3 (‘Vinda Kitchen Towel super absorbent’) used in the second experiment.


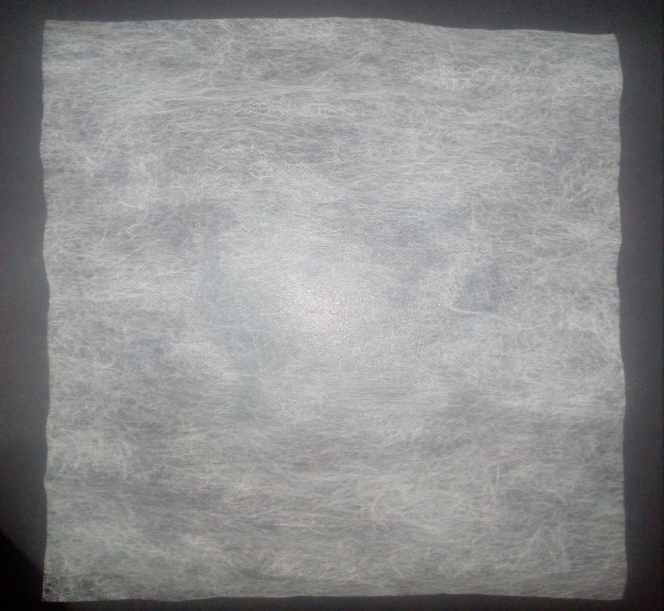

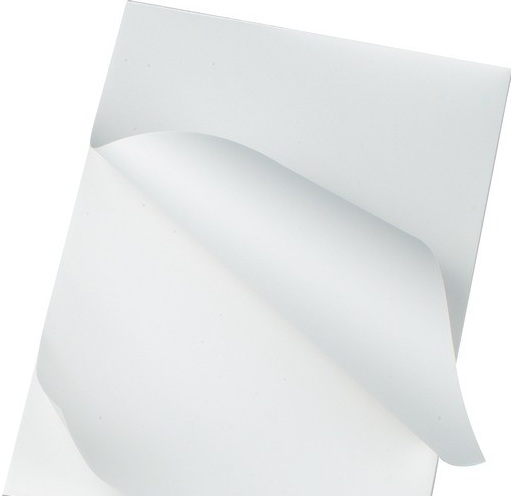


**Fig. 2.12:** Nappy liner (left) and Gouache paper (right) used in the 2^nd^ experiment.


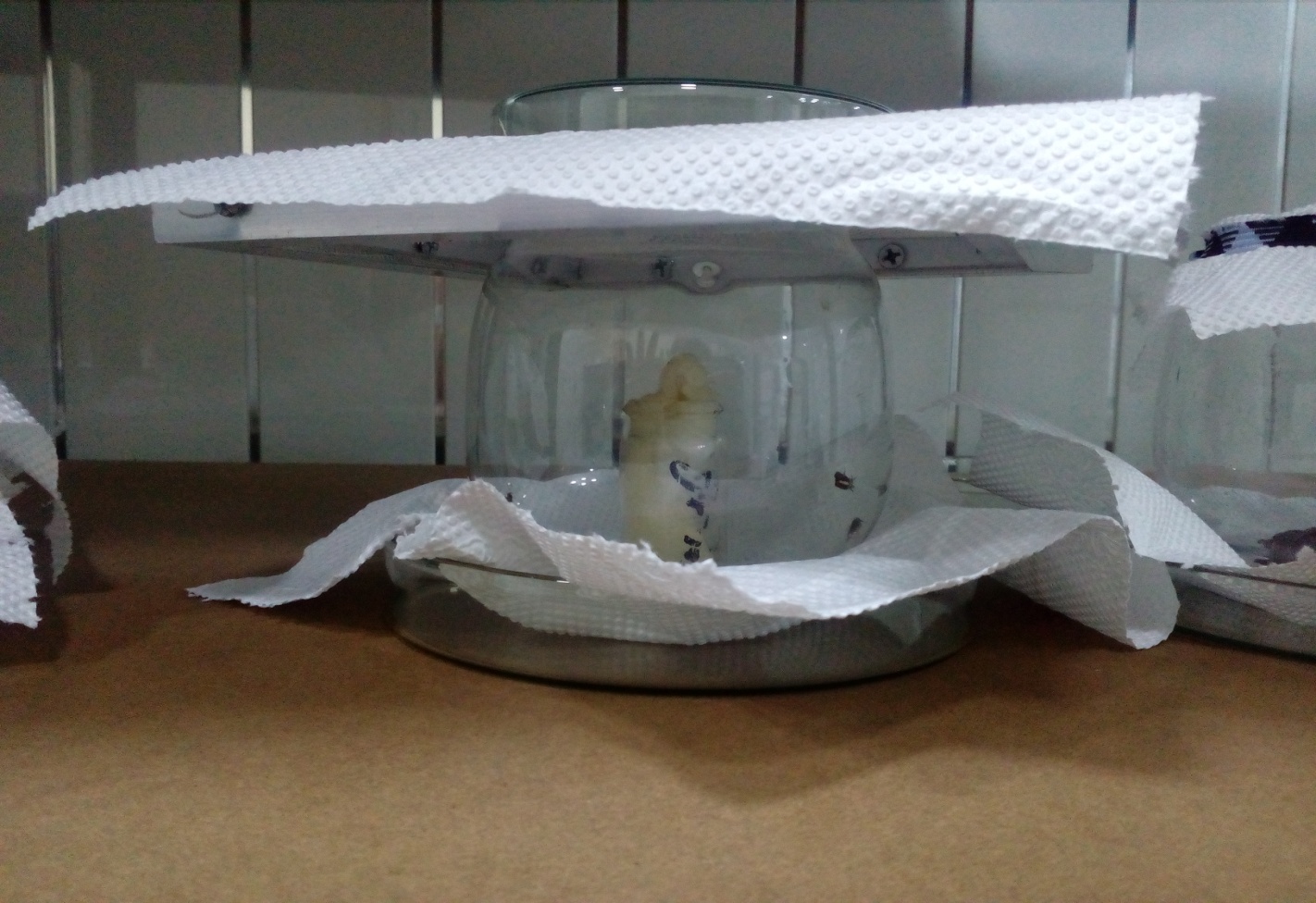


**Fig.2.13:** 2^nd^ experiment, to analyze the oviposition behaviour by using kitchen towel, was performed.


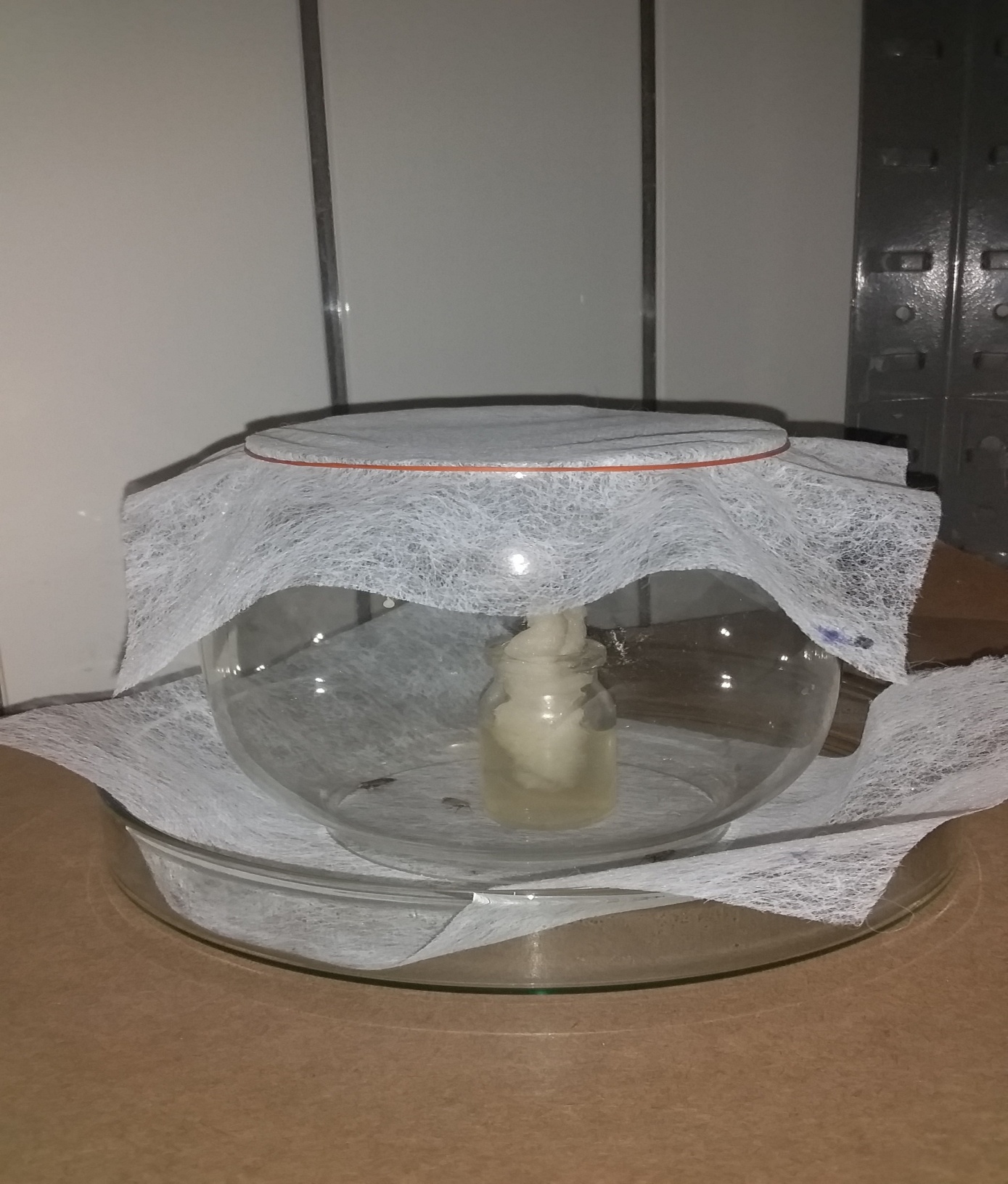


**Fig. 2.14:** 2^nd^ experiment performed by using Nappy liner in the laboratory.


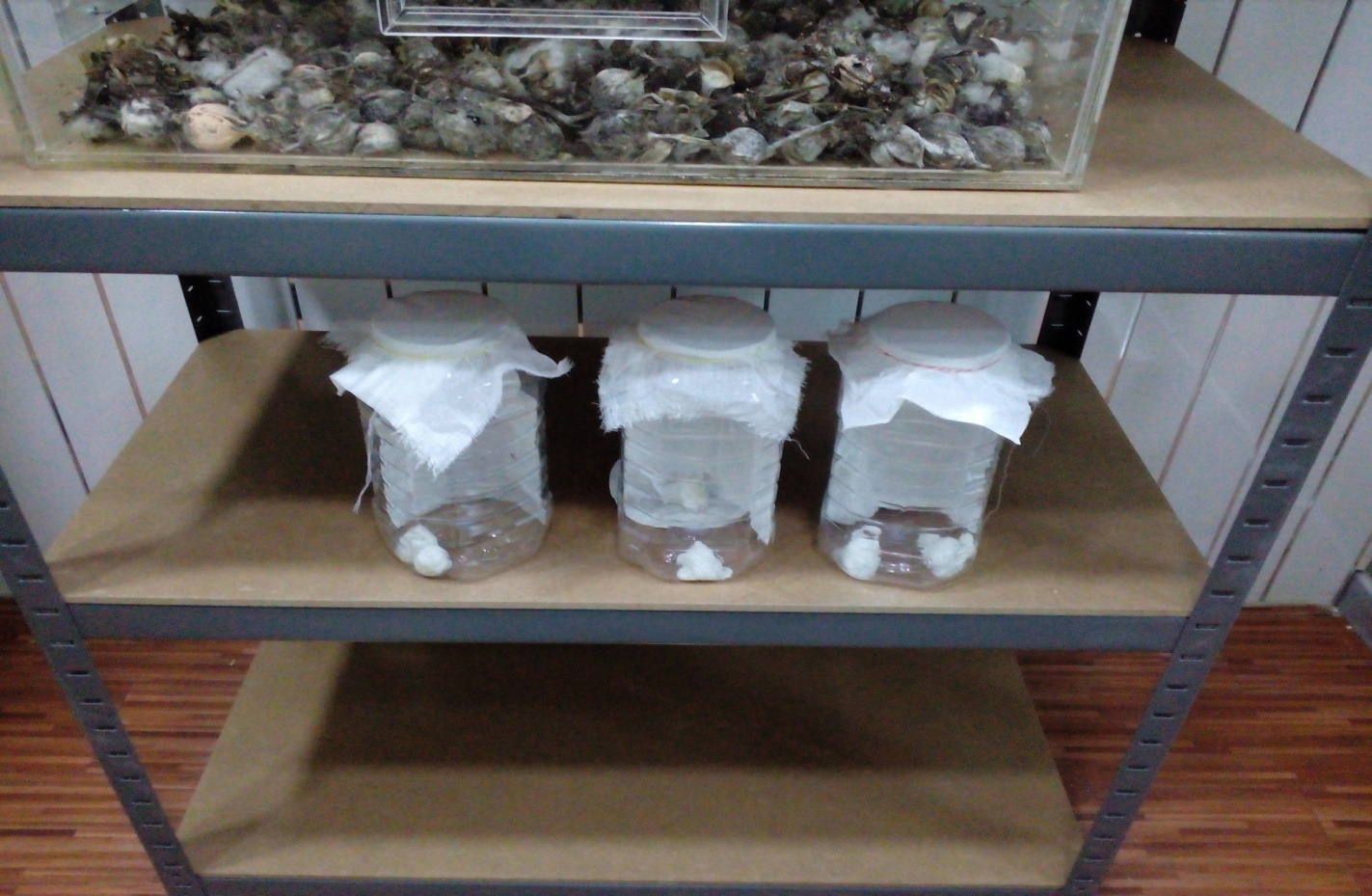


**Fig. 2.15:** Gouache paper at the top of jar that provides space for egg laying.


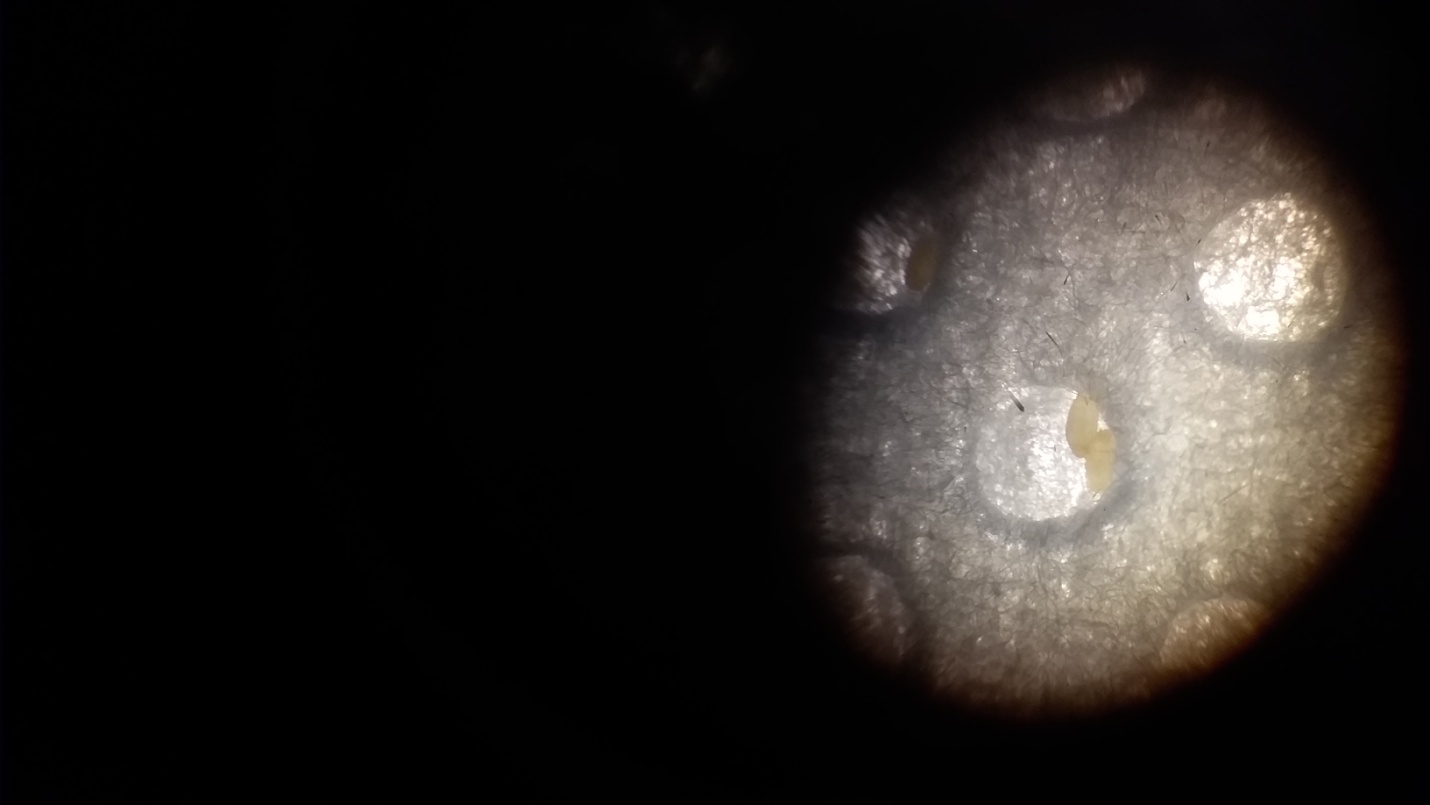


**Fig. 2.16:** Gouache paper under microscope with laid eggs.

**
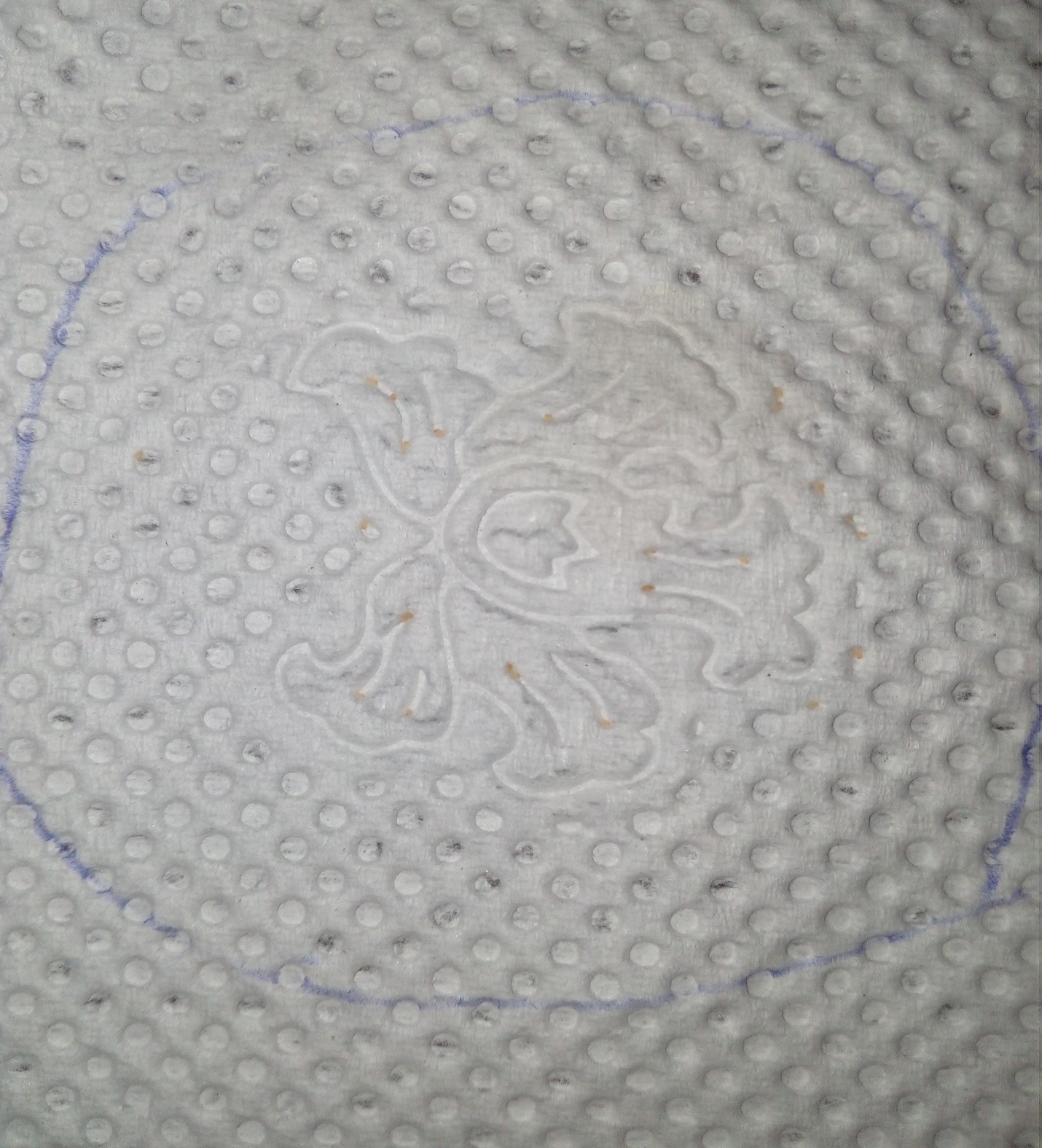
**

**Fig. 2.17:** Eggs (light pinkish) of pink bollworm inside the ridges of kitchen towel (artificial egg receptacle).

**
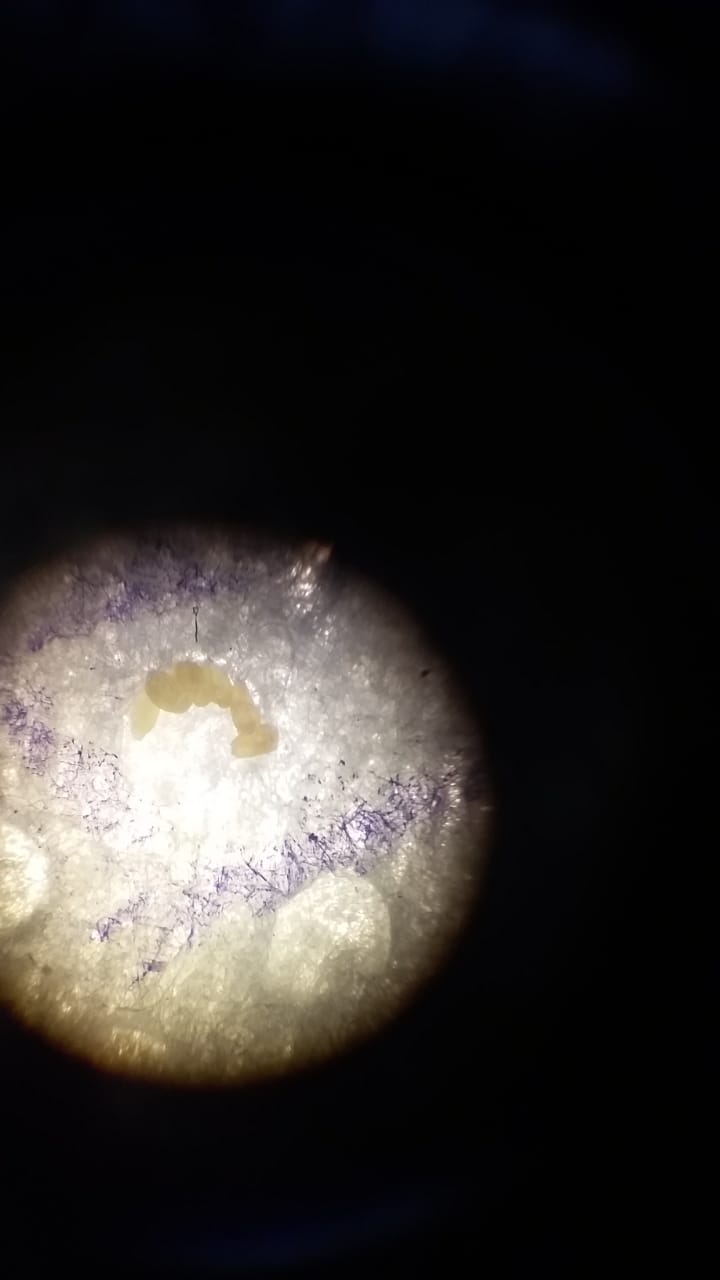
**

**Fig. 2.18:** Nappy liner under microscope with laid eggs.

**
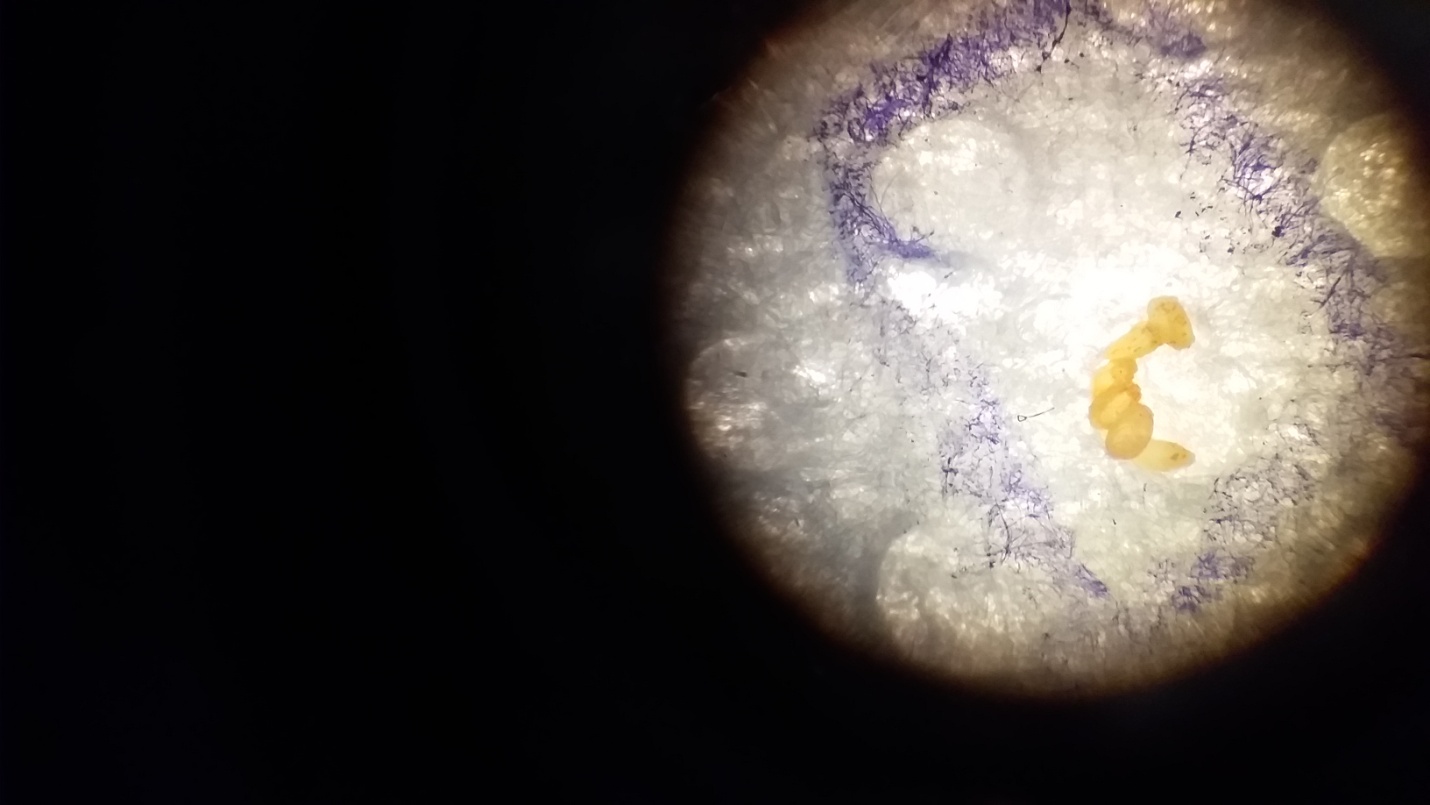
**

**Fig. 2.19:** Eggs of pink bollworm on nappy liner and kitchen towel under Stereo microscope.

**
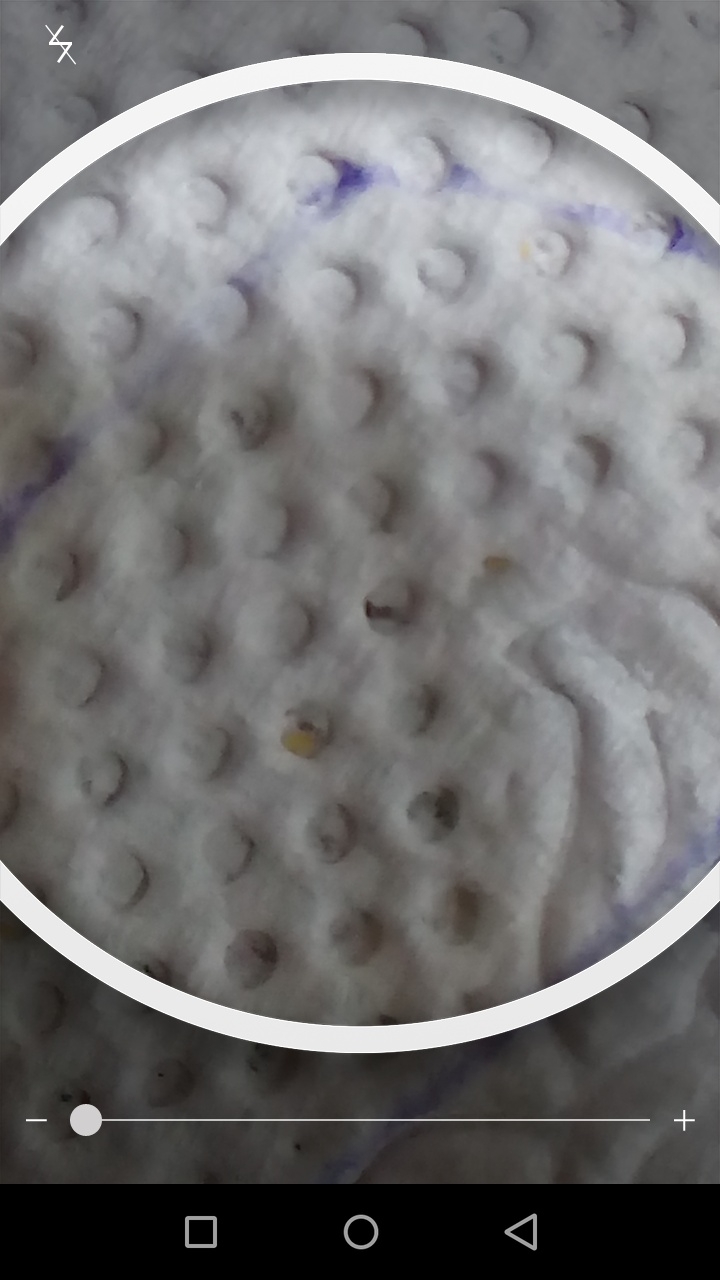
**

**Fig. 2.110:** Eggs of pink bollworm.

**Statistical Analysis**

For statistical analysis, the collected data was subjected to analysis of variance and Tukey’s HSD test was used to separate the means.

**RESULTS**

**Experiment # 1: Oviposition preference for colors of oviposition substrate**

Analysis of variance parameters depicted that color of artificial oviposition substrate significantly affected the oviposition behaviour of pink bollworm (P<0.05) (Table 2.3.1). Artificial oviposition substrate of white colour showed the highest oviposition (33.67eggs/female) followed by green color (29.67eggs/female) and red color (27.01eggs/female) (Table 2.3.2).

**Table: 2.3.1. ANOVA parameters regarding single spawn oviposition of pink bollworm on different colored oviposition substrate.**

| **SOV** | **df** | **SS** | **MS** | **F** | **P** |
| --- | --- | --- | --- | --- | --- |
| Color of artificial egg receptacle | 2 | 1917.56 | 958.78 | 332 | 0.0000^**^ |
| Error | 6 | 17.33 | 2.89 |  |  |
| Total | 8 | 1934.89 |  |  |  |

Grand Mean = 127.11 CV = 1.34 ^**^Highly Significant (P<0.05)

**Table: 2.3.2.** **Oviposition of pink bollworm (Mean** ±**SE) on different colored egg substrate.**

| **Artificial egg receptacle** | **Oviposition± S.E/female** |
| --- | --- |
| White | 33.67 ± 1.42a |
| Green | 29.67 **±** 1.38b |
| Red | 27.01 **±**1.31c |

**Experiment # 2: Oviposition preference for textures of oviposition substrate**

**Oviposition of pink bollworm on different textured egg substrates**

Analysis of variance parameters depicted that texture of artificial oviposition substrate significantly affected the oviposition behaviour of pink bollworm (P<0.05) (Table 2.3.3). Artificial oviposition substrate of kitchen towel showed the highest oviposition (32.67eggs/female) followed by nappy liner (31.01eggs/female) and gouache paper (29.02eggs/female (Table 2.3.4).

**Table: 2.3.3.** ANOVA parameters regarding oviposition of pink bollworm on different textured oviposition substrate.

| **SOV** | **Df** | **SS** | **MS** | **F** | **P** |
| --- | --- | --- | --- | --- | --- |
| Texture of artificial egg receptacle | 2 | 1937.56 | 968.78 | 60.1 | 0.0001^**^ |
| Error | 6 | 96.67 | 16.12 |  |  |
| Total | 8 | 2034.22 |  |  |  |

Grand Mean = 136.56 CV = 2.94 ^**^Highly Significant (P<0.05)

**Table: 2.3.4.** Oviposition of pink bollworm (Mean ±SE) on different textured egg substrate.

| **Artificial egg receptacles** | **Oviposition± S.E/female** |
| --- | --- |
| Gouache paper | 29.02 ± 3.25a |
| Nappy liner | 31.01**±** 3.27b |
| Kitchen Towel | 32.67**±**3.28c |

**ACTIVITY-4: ASSESSMENT OF SURVIVAL AND REPRODUCTION POTENTIAL OF PINK BOLLWORM (*PECTINOPHORA GOSSYPIELLA*) (SAUNDERS) ON DIFFERENT ADULT DIETS**

**METHODOLOGY**

Newly emerged adults were collected from the plastic cages with the help of plastic voile. These adults were then placed in each glass chimneys with the ratio of 3 male : 3 female. The glass chimneys were covered with the tissue paper tightly from the top for the oviposition and to prevent the adults to escape from the chimney. The chimneys were placed at the temperature of 29 ^0^C and 70% RH.

These adults were fed upon the three different adult diets (honey, sucrose and glucose) and these diets were given in the solution form by making their different concentration levels (5%, 10% and 15%) given below (Table 3.1). The cotton were dipped in the solution and placed in the center of chimney for the feeding of adults (Fig 3.1). The diet were changed after every 2 days. Survival rate of adults male and female were observed at each concentration of each diet. The longevity observed at different diet was in between the 12-20 days. The chimneys were observed on the daily bases for the eggs production.

**Table: 2.4.1. Different adult diets and their concentrations given to adults.**

| **Treatments** | **Adult-diet-1** | **Adult-diet-2** | **Adult-diet-3** |
| --- | --- | --- | --- |
| Honey solution | 5% | 10% | 15% |
| Sucrose Solution | 5% | 10% | 15% |
| Glucose Solution | 5% | 10% | 15% |

**Collection of eggs**

Females of pink bollworms were laid the eggs singally or in the form of batch at the tissue paper which also used for the sealing of chimney. Tissue paper were observed on the daily bases under the microscope to ensure the presence of eggs. Tissue paper having the eggs were removed from the top of chimney and placed in another plastic jar and the glass chimney were covered with another tissue paper. The eggs from the each concentration were kept in separate plastic jar. Eggs were initially white in the color but later they turn in to the orange color. Eggs incubation period were in between 3-4.5 days. Eggs with in the plastic jars were maintained at the 29 ^0^C and 60±10% RH.

**
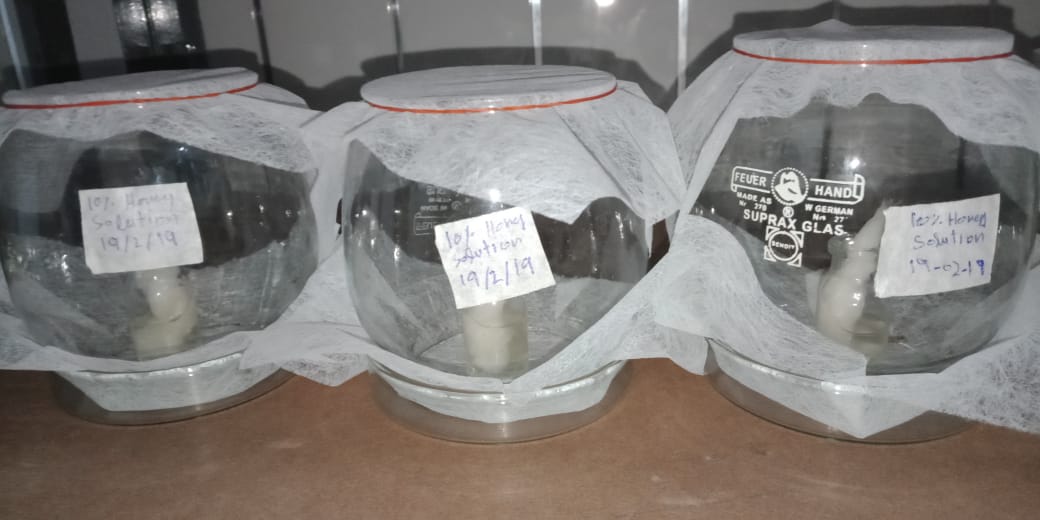
**

**Figure 2.21:** Adults of pink bollworm on the 10% of honey solution.


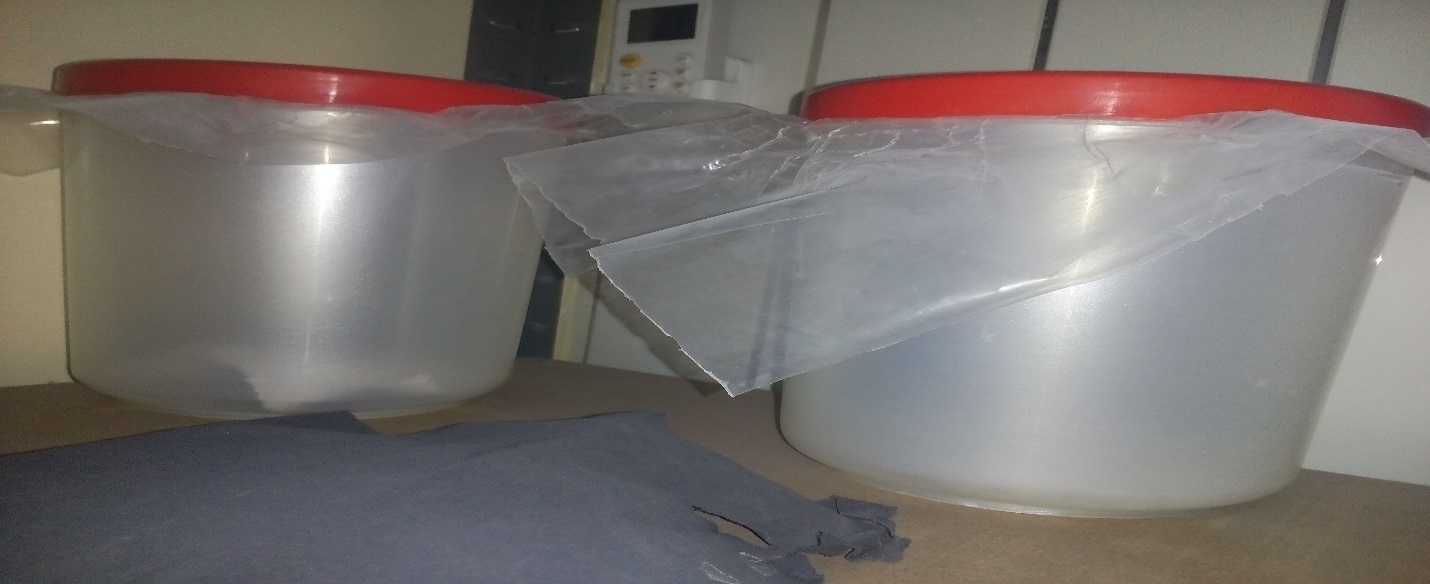


**Figure 2.22:** Tissue paper containing pink bollworm eggs transferred to sealed jar.

**Collection of larva**

Newly hatched larvae were white in color with brown head and later it turns in the pink color when it reaches at 4^th^ instar. Newly emerged larvae were swapped with the help of camel brush and placed in the culture plates. The culture plates were covered with filter paper and lid. Culture plates tied with the rubber bands to avoid the escaping of larvae from the culture plates. Each larva which emerged from different adults diet eggs were kept separately. All larvae which emerge from the different concentrations of different adult diets were fed upon the standard artificial medium.


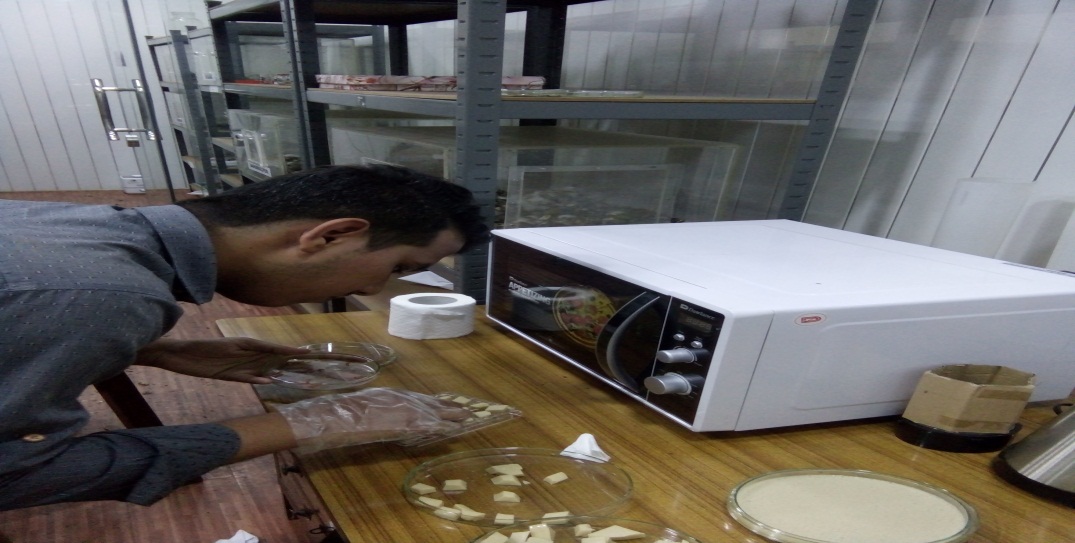


**Figure 2.23:** Shifting of pink bollworm larva on wheat germ diet.

**Statistical analysis of data**

The experiment was laid out under completely randomized design. The results were analyzed by using the Statistix 8.1 software. Means of treatments and concentrations were compared by appealing Least Significance Difference (LSD) test.

**RESULTS**

**Longevity of *Pectinophora gossypiella* adults on different adult diets.**

Analysis of variance table shows that longevity of the *P. gossypiella* was significantly affected by different concentrations of different adult diets. The treatment and concentrations also had highly significant effect on the longevity of *P. gossypiella* P < 0.01 but the interaction between the treatment and concentrations had non-significant effect on longevity P > 0.05 (Table 4.4.2).

Mean comparison test of longevity on different adults diet showed that highest longevity of *P. gossypiella* was recorded on the honey Solution 11.94 days. Longevity of *P. gossypiella* on the sucrose solution was 10.83 days. Lowest longevity was observed in *P. gossypiella* on glucose solution 9.88 days. However irrespective of diet highest longevity was observed on the 10% solution 12.61 days followed by the longevity on 15% solution 11.00 days and 5% solution 9.05 days (Table 4.4.3).

The result of interaction between the treatment and concentrations indicated that longevity of *P. gossypiella* adults on honey solution 5%, 10% and 15% was 10.00 days, 12.83 days and 12.00 days respectively. Similarly, longevity of *P. gossypiella* adults was recorded 9.00 days at 5%, 12.50 days at 10% and 11.00 days on 15% sugar solution, likewise the longevity of *P. gossypiella* adults was 8.16 days at 5%, 11.50 days at 10% and 10.00 days at 15% of glucose starch. The comparison indicated that 10% honey solution demonstrated the highest longevity and 5% glucose solution exhibited the minimum longevity of *P. gossypiella*adults (Table 4.4.3).

**Table 2.4.2: ANOVA parameters regarding the effect of different adult diets, concentrations and their interaction on the longevity of *Pectinophora gossypiella* adults.**

| **SOV** | ***Df*** | **SS** | **MS** | **F** | **P** |
| --- | --- | --- | --- | --- | --- |
| Treatment (diets) | 2 | 19.0556 | 9.5278 | 33.06 | 0.0000** |
| Concentrations | 2 | 57.0556 | 28.5278 | 98.99 | 0.0000** |
| Treat*Conc | 4 | 0.22222 | 0.0556 | 0.19 | 0.9386^NS^ |
| Error | 18 | 4.6111 | 0.2882 |  |  |
| Total | 26 | 81.1667 |  |  |  |

If P ≤ 0.05= Significant, If P ≥ 0.05= Non significant (NS) **= Highly significant

**Table 2.4.3 Longevity (days) of *Pectinophora gossypiella* adults on different concentrations of honey, sucrose and glucose in adult diets.**

| **Treatment** | **Longevity (days) of *Pectinophora gossypiella* adults** | | | **Mean** |
| --- | --- | --- | --- | --- |
|  | **5% concentration** | **10% concentration** | **15% concentration** |  |
| Honey | 10.00e | 12.83a | 12.00bc | **11.94a** |
| Sucrose | 9.00f | 12.50b | 11.00d | **10.83b** |
| Glucose | 8.16f | 11.50cd | 10.00e | **9.88c** |
| **Mean** | **9.05c** | **12.61a** | **11.00b** |  |

Values having the same alphabets are not significantly different.

**Eggs / life span of *Pectinophora gossypiella* adults fed on different adult diets.**

Analysis of variance showed that eggs / life span of *P. gossypiella*adults were significantly affected by the different concentrations of different adult diets. The treatment, concentration and interaction between treatment and concentration also had highly significant effect on the eggs / life span of *P. gossypiella* adults P < 0.01 (Table 4.4.4).

Mean comparison test of eggs / life span on different adults diet showed that maximum eggs / life span of *P. gossypiella* was recorded on the honey Solution 34.55 eggs. Eggs / life span of *P. gossypiella* on the sucrose solution were 30.11 eggs. Lowest eggs / life span were observed in *P. gossypiella* on glucose solution 26.00 eggs. However irrespective of diet highest eggs / life span was observed on the 10% solution 38.00 eggs followed by eggs / life span on 15% solution 29.88 eggs and 5% solution 22.77 (Table 4.4.5).

The result of interaction between the treatment and concentrations indicated that eggs / life span of *P.gossypiella* adults on honey solution 5%, 10% and 15% were 24.66 eggs, 44.66 eggs and 34.33 eggs. Similarly, eggs / life span of *P. gossypiella* adults was recorded 22.66 eggs at 5%, 38.33 eggs at 10% and 29.33 on 15% sugar solution, likewise the eggs / life span of *P. gossypiella* adults was 21.00 eggs at 5%, 31.00 eggs at 10% and 26.00 eggs at 15% of glucose starch. The comparison indicated that 10% honey solution demonstrated the highest eggs / life span and 5% glucose solution exhibited the minimum eggs / life span of *P. gossypiella*adults (Table 4.4.5).

**Table 2.4.4: ANOVA parameters regarding the effect of different adult diets, concentrations and their interaction on the eggs / life span of *Pectinophora gossypiella* adults.**

| Sov | *Df* | SS | MS | F | P |
| --- | --- | --- | --- | --- | --- |
| Treatment (diets) | 2 | 329.56 | 164.778 | 45.63 | 0.0000** |
| Concentrations | 2 | 1044.22 | 522.111 | 114.58 | 0.0000** |
| Treat*Conc | 4 | 76.89 | 19.222 | 5.32 | 0.0064** |
| Error | 18 | 57.78 | 3.611 |  |  |
| Total | 26 | 1512.67 |  |  |  |

If P ≤ 0.05= Significant, If P ≥ 0.05= Non significant (NS) **= Highly significant

**Table 2.4.5: Eggs / life span of *Pectinophora gossypiella* adults on different concentrations of honey, sucrose and glucose in adult diets.**

| Treatment | **Eggs / life span of *Pectinophora gossypiella* adults** | | | **Mean** |
| --- | --- | --- | --- | --- |
|  | **5% concentration** | **10% concentration** | **15% concentration** |  |
| Honey | 24.66ef | 44.66a | 34.33c | **34.55a** |
| Sucrose | 22.66fg | 38.33b | 29.33d | **30.11b** |
| Glucose | 21.00g | 31.00d | 26.00e | **26.00c** |
| **Mean** | **22.77c** | **38.00a** | **29.88b** |  |

The values having the same alphabet are not significantly different.

**Incubation period (days) of *Pectinophora gossypiella* on different adult diets.**

Analysis of variance indicated that incubation period of *Pectinophora gossypiella* was significantly affected by the different concentration of different adult diets. The treatment and concentration also had highly significant effect on incubation period of *P. gossypiella* P < 0.01 but interaction between the treatment and concentration had non-significant effect P > 0.05 (Table 2.4.6).

Mean comparison test of incubation period on different adult diets indicated that they were significantly different from each other. Incubation period of *P. gossypiella* eggs were high on the honey solution 5.33 days. Incubation period of *P. gossypiella* eggs on the sucrose solution were 4.50 days. Lowest incubation period were observed in *P. gossypiella* on glucose solution 3.95 days. Mean comparison test of *P. gossypiella* on different concentration also significantly different from each other. However, irrespective of diet longest incubation period of *P. gossypiella* eggs were on the 10% solution 5.50, followed by the incubation period 4.66 days on 15% solution and lowest on 5% solution 3.62 days (Table 4.4.7).

The result of interaction between the treatment and concentration of *P. gossypiella* indicated that incubation period of eggs are not much affected by both factors. Incubation period of *P.gossypiella* eggs on honey solution were 4.33 days, 6.33 days and 5.33days on 5%, 10% and 15% solution respectively. Similarly incubation period of *P. gossypiella* eggs on sucrose solution were 3.33 days, 5.50 days and 4.66 days on 5%, 10% and 15% solution. Likewise, incubation period of *P. gossypiella* eggs were 3.20 days, 4.66 days and 4.00 days on 5%, 10% and 15% solution of glucose. The comparison indicated that 10% honey solution demonstrated the longest incubation period and 5% glucose solution exhibited the smallest incubation period of *P. gossypiella* eggs (Table 4.4.7).

**Table 2.4.6: ANOVA parameters regarding the effect of different adult diets, concentrations and their interaction on the incubation period of *Pectinophora gossypiella* eggs.**

| Sov | *df* | SS | MS | F | P |
| --- | --- | --- | --- | --- | --- |
| Treatment (diets) | 2 | 8.6674 | 4.33370 | 15.83 | 0.0000** |
| Concentrations | 2 | 15.9341 | 7.96704 | 29.11 | 0.0000** |
| Treat*Conc | 4 | 0.4681 | 0.11704 | 0.43 | 0.7866 ^NS^ |
| Error | 18 | 4.3793 | 0.27370 |  |  |
| Total | 26 | 29.6296 |  |  |  |

If P ≤ 0.05= Significant, If P ≥ 0.05= Non significant (NS) **= Highly significant

**Table 2.4.7: Incubation period (days) of *Pectinophora gossypiella* eggs on different concentrations of honey, sucrose and glucose in adult diets.**

| Treatment | **5% concentration** | **10% concentration** | **15% concentration** | **Mean** |
| --- | --- | --- | --- | --- |
| Honey | 4.33c | 6.33a | 5.33b | **5.33a** |
| Sucrose | 3,33d | 5.50ab | 4.66bc | **4.50b** |
| Glucose | 3.20d | 4.66bs | 4.00cd | **3.95c** |
| **Mean** | **3.62c** | **5.50a** | **4.66b** |  |

Mean values having the same alphabets are not significantly different.

**Egg hatching (%) of *Pectinophora gossypiella* on different adult diets.**

Analysis of variance table indicated that % of egg-hatching of *P. gossypiella* was significantly affected by the different concentration of different adult diets. The treatment and concentration also had highly significant effect on egg-hatching P < 0.01 however, interaction between treatment and concentration had significant effect P < 0.05 (Table 2.4.8).

Mean comparison test for the egg-hatching of *P. gossypiella* on different treatments indicating that they were significantly different from each other. Egg-hatching of *P. gossypiella* was higher on sucrose solution 86.81%. Egg-hatching of *P. gossypiella* on honey solution was 77.83% and egg-hatching was minimum on glucose solution 67.03%. Mean table indicating that concentrations were not significantly different from one another. However, irrespective of diet maximum egg-hatching of *P. gossypiella* was observed on 10% solution 82.30%, followed by the 15% of solution 76.89% and 5% solution 72.47% (Table 2.4.9).

The result of interaction between the treatment and concentration indicated that egg-hatching of *P. gossypiella* was affected by these both of factors. Egg-hatching of *P. gossypiella* was recorded 72.99%, 82.82% and 73.63% on 5%, 10% and 15% of honey solution respectively. Similarly egg-hatching of *P. gossypiella* was recorded 80.92%, 93.18% and 86.33% on 5%, 10% and 15% of sucrose solution respectively. Likewise egg-hatching was 63.51%, 70.90% and 66.68% when adults fed on 5%, 10% and 15% of glucose starch. The comparison indicated that 10% of sucrose solution demonstrated the maximum egg-hatching and 5% of glucose exhibited the minimum egg-hatching of *P. gossypiella* (Table 2.4.9).

**Table 2.4.8: ANOVA parameters regarding the effect of different adult diets, concentrations and their interaction on theegg-hatching (%) of *Pectinophora gossypiella*.**

| Sov | *Df* | SS | MS | F | P |
| --- | --- | --- | --- | --- | --- |
| Treatment (diets) | 2 | 1765.56 | 882.781 | 596.53 | 0.0000** |
| Concentrations | 2 | 436.29 | 218.147 | 147.41 | 0.0000** |
| Treat*Conc | 4 | 18.00 | 4.500 | 3.04 | 0.0483 |
| Error | 18 | 23.68 | 1.480 |  |  |
| Total | 26 | 2245.57 |  |  |  |

If P ≤ 0.05= Significant, If P ≥ 0.05= Non significant (NS) **= Highly significant

**Table 2.4.9: Egg-hatching (%) of *Pectinophora gossypiella* on different concentrations of honey, sucrose and glucose in adult diets.**

| Treatment | **Egg-hatching (%)** | | | **Mean** |
| --- | --- | --- | --- | --- |
|  | **5% concentration** | **10% concentration** | **15% concentration** |  |
| Honey | 72.99e | 82.82c | 77.67d | **77.83b** |
| Sucrose | 80.92c | 93.18a | 86.33b | **86.81a** |
| Glucose | 63.51g | 70.90e | 66.68f | **67.03c** |
| **Mean** | **72.47c** | **82.30a** | **76.89c** |  |

Mean values having the same alphabets are not significantly different.

**OBJECTIVE-3: DETERMINATION OF ALTERNATE HOST PLANTS OF PBW**

- **Activity-1: Determination of on- and off-season prevalence of PBW on alternative host plants**

**Methodology and Results:**

Different reviews revealed that pink bollworm is polyphagous. According to this review, four host plants Okra, Tomato, Lucern and Gul e Khaira were selected and grown in Young wala, University of Agriculture, Faisalabad. Okra were grown at an area of five marla on 9-May-2018 and 9-May-2019 and the remaining host plants have winter growing season, So the other crops were grown at an area of one malra each on 01-December-2018 and 01-December-2019. Due to cooled winter, these host plants were covered with polythene sheets to protect from harsh environment. After 45 days of okra sowing, data was taken to check the presence of pink bollworm on okra. Data was taken fortnightly but not the single larvae were found on okra till crop harvest on 26-September-2018 and 26-September-2019. Lucern, Tomato and Gul-e-Khaira were sown on 01-December-2018 and 01-December-2019 as a nursery plants and covered with polythene sheets due to cooled winter. There was no pink bollworm infestation observed on alternate host plants (Appendix-III & IV).

**OBJECTIVE-4:** TO STUDY DIAPAUSING BEHAVIOUR OF PINK BOLLWORM

**4.1. Activity-1: Different life stages of the PBW will be studied under different types of artificial diets and different temperature regimes in the laboratory**

**4.1.1 INFLUENCE OF DIFFERENT LARVAL DIETS AND TEMPERATURE REGIMES ON THE DIAPAUSING BEHAVIOUR AND OTHER BIOLOGICAL PARAMETERS OF *PECTINOPHORA GOSSYPIELLA* (GELECHIIDAE: LEPIDOPTERA)**

Two experiments were carried out to study influence of different larval diets and temperature regimes on the diapausing behaviour of *Pectinophora gossypiella* (Gelechiidae: Lepidoptera). The details, methodologies and results are discussed below.

**MATERIALS AND METHODS**

**Rearing of pink bollworm**

Bolls infested with pink bollworm was collected and placed in the adult emergence cages, till moth emergence. The adult moths were aspired and shifted into egg collection cages. The emerged virgin adults with sex ratio of 1:1 (male: female) was shifted in each jar where they were fed with 8% honey solution. The duration of pre-oviposition period was usually 2 to 3 days. The eggs collected by releasing the adults in a jar covered with gouache paper. The eggs attached with gouache paper was placed in sealed glass bottle. The glass bottles wasmanipulated at 29±1 ºC and 60±10% RH. Artificial diet was cut in cubes of sizes 1×1×1 cm^3^ pieces and dispended in 24-well culture plates. The newly hatched larvae was gently swapped to artificial diet with a fine camel hair brush and covered with blow molding paper and lid. The culture plates were tied with rubber band and put in insectary maintained at 29±1ºC, 40±10% RH until larvae transformed into pupae. After larvae becomes pupae in 24-well culture plates. Pupae was collected from culture plates with a tweezers and put in a cylindrical box (6cm in diameter and 4cm in height) till the adult emergence.

Adult was released in cylindrical egg collection cages with diameter of 11cm and a height of 12cm at 29±1ºC, 70±10% RH and fed with 5% honey solution. The top of each cage is covered with gouache paper for oviposition. Egg paper was harvested daily during the entire oviposition period and placed in glass bottle until egg hatching starts. Adults was released into cylindrical egg cages with a diameter of 11 cm and a height of 12 cm at 29 ± 1 ºC and a relative humidity of 70 ± 10% and offered with 5% honey solution. The top of each cage is covered with gouache paper for egg laying. The eggs from egg paper was collected daily during the whole egg laying period and placed in a glass bottle till egg hatching

**
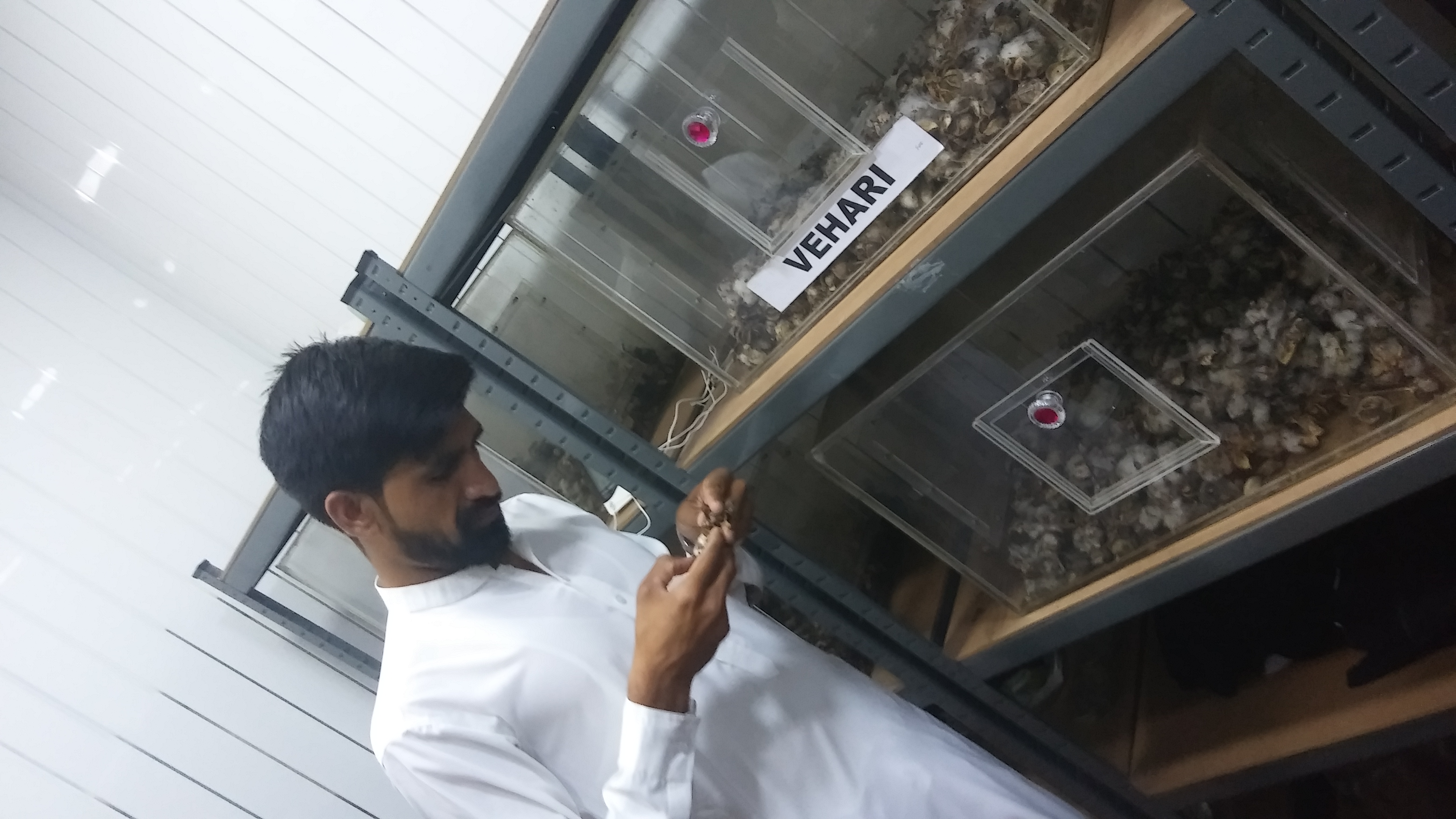
**

**Pupal observation of pink bollworm inside the infested bolls of cotton**


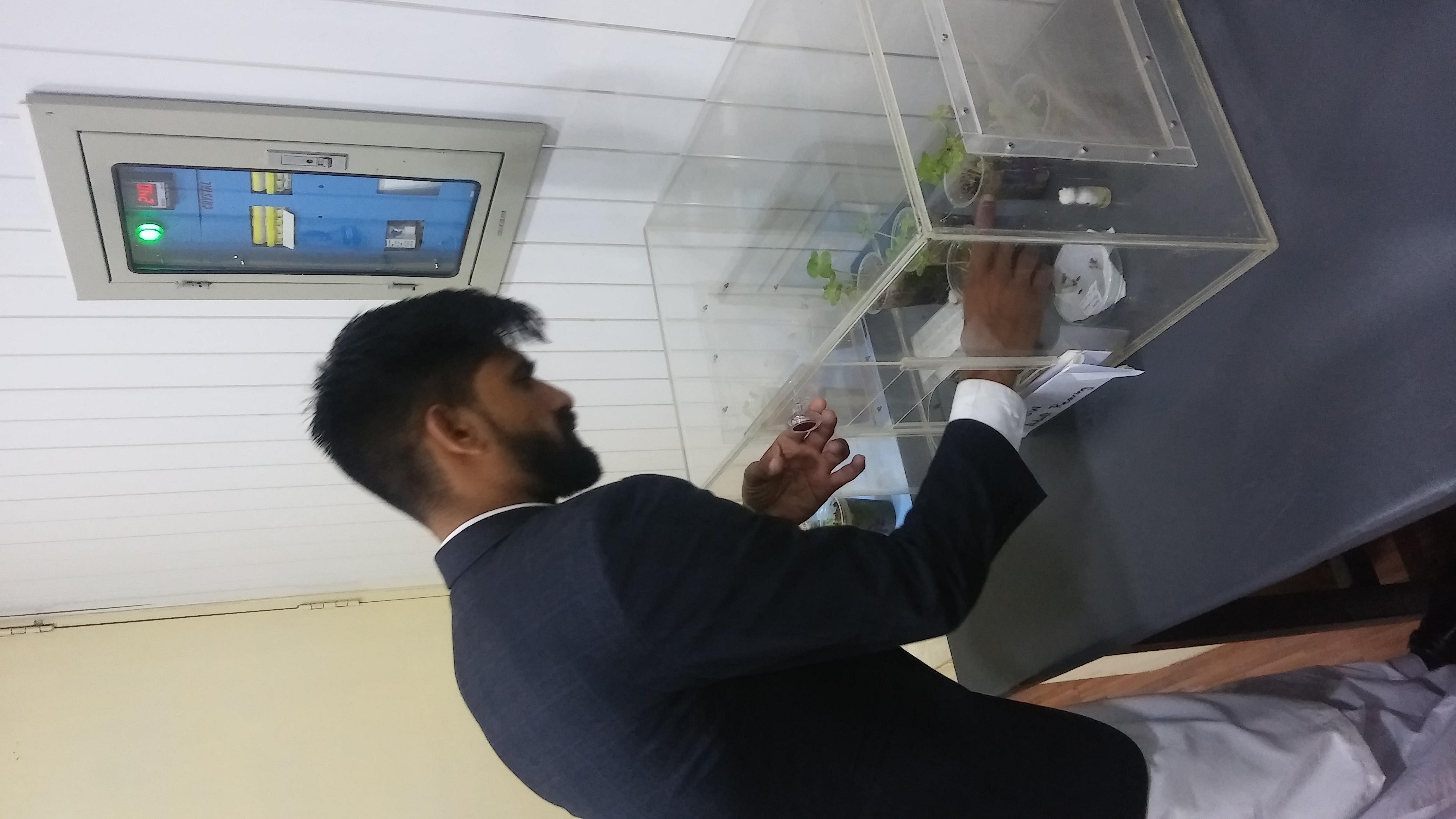


**Eggs observation of pink bollworm on the potted cotton plants placed inside the adult cages**

**Preparation of artificial diet**

One standard and eight modified diets was prepared by using the procedure and materials described by Ali *et al.* (2018). Standard diet was prepared by mixing wheat germ 34.5 g, casein 30 g, sucrose 10 g, brewer’s yeast 5g, alpha cellulose 1g, Potassium Sorbate 1.5 g, wipalgin 0.5g, daca vitamin 0.01g, choline chloride 0.06g, maize oil 3.3ml and 2ml honey in 500 ml water and stirring it completely then 230 ml water is taken in a glass beaker and added 20g agar and heating it in an oven until its color become clear and allowed to cool at room temperature then mixing both solution in a mixer to get final paste and this diet is incubated at 27°C in an incubator and cut into small cubes (1×1×1 cm^3^) for larval feeding. The modified diet was prepared by using the above mentioned procedure and materials except corn oil casein sucrose which are source of fats, protein and carbohydrate, as these nutrients act as a diapause inducing variants. All the ingredients of both standard and modified diets was mixed according to the quantities given in a Table 4.1.

**Procedure for Experiment 1: Evaluate eight modified artificial diets and a standard artificial diet for their effects on diapausing-behavior and other biological parameters of pink bollworm**

This experiment was carried out to evaluate eight modified artificial diets and a standard artificial diet for their effects on diapausing behavior and other biological parameter of pink bollworm. The prepared diets were cut into small cubes (1×1×1 cm^3^) and shifted into 24 well culture plates. Separate 24 well culture plates were used for each diet. A set of three culture plates was used for each diet and that will serve as, three replications. In each plate twenty-four (24) larvae of same age were released on the diet in each culture plates. Same age larvae were released to nine different type of diets (1 standard and 8 modified artificial diets). The culture plate having diet and larvae was covered with filter paper and lid of the culture plate and tighten with rubber band. Thus prepared set of cultured plates were placed inside the incubation chamber maintained at conducing temperature (29+1°C), relative humidity (40+10) and photoperiod (14:10 hours) and was observed after 15 to 30 days of treatment application. The diapausing (non-pupated larvae) and non diapausing (pupated larvae) were counted and transformed into percent diapausing larvae. The entire experiment was carried out in CRD with three replications.

**Table 4.1: Different ingredients of nine diets and compositional variations (by weight or volume) of these ingredients.**

| **Ingredients** | **Standard**  **Diets-5**  **g/Kg or ml/L** | **MD-1**  **g/Kg or ml/L** | **MD-2**  **g/Kg or ml/L** | **MD-3**  **g/Kg or ml/L** | **MD-4**  **g/Kg or ml/L** | **MD-6**  **g/Kg or ml/L** | **Vitamins** | | |
| --- | --- | --- | --- | --- | --- | --- | --- | --- | --- |
|  |  |  |  |  |  |  | **MD-7**  **g/Kg or ml/L** | **MD-8**  **g/Kg or ml/L** | **MD-9**  **g/Kg or ml/L** |
| Wheat germ | 34.5 | 44.5 | 39.5 | 29.5 | 24.5 | 19.5 | 34.5 | 34.5 | 34.5 |
| Casein | 30 | 40 | 35 | 25 | 20 | 15 | 30 | 30 | 30 |
| Agar | 20 | 20 | 20 | 20 | 20 | 20 | 20 | 20 | 20 |
| Sucrose | 10 | 14 | 12 | 08 | 06 | 04 | 10 | 10 | 10 |
| Brewer,s yeast | 5.0 | 9.0 | 7.0 | 3.0 | 1.0 | 5.0 | 5.0 | 5.0 | 5.0 |
| Alpha cellulose | 1.0 | 1.0 | 1.0 | 1.0 | 1.0 | 1.0 | 1.0 | 1.0 | 1.0 |
| Potassium Sorbate | 1.5 | 1.5 | 1.5 | 1.5 | 1.5 | 1.5 | 1.5 | 1.5 | 1.5 |
| Wipalgin | 0.5 | 0.5 | 0.5 | 0.5 | 0.5 | 0.5 | 0.5 | 0.5 | 0.5 |
| Daca vitamin | 0.01 | 0.01 | 0.01 | 0.01 | 0.01 | 0.01 | 0.02 | 0.005 | 0.0025 |
| choline Chloride | 0.06 | 0.06 | 0.06 | 0.06 | 0.06 | 0.06 | 0.06 | 0.06 | 0.06 |
| Maize Oil | 3.3 | 5.3 | 4.3 | 2.3 | 1.3 | 0.3 | 3.3 | 3.3 | 3.3 |
| Honey | 2.0 | 2.0 | 2.0 | 2.0 | 2.0 | 2.0 | 2.0 | 2.0 | 2.0 |
| Water | 730 | 730 | 730 | 730 | 730 | 730 | 730 | 730 | 730 |


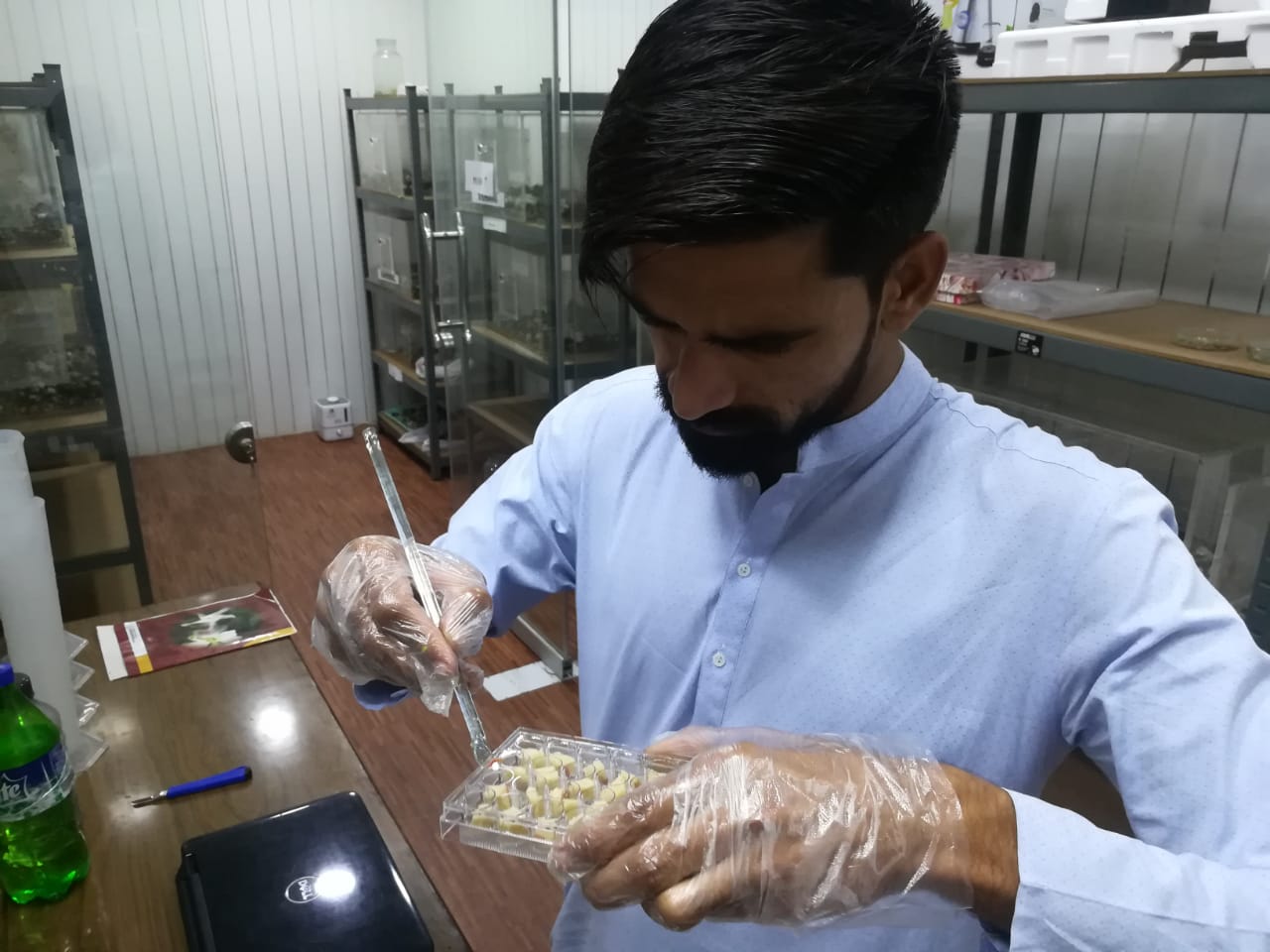


**Shifting of Larvae of Pink Bollworm on Diet**


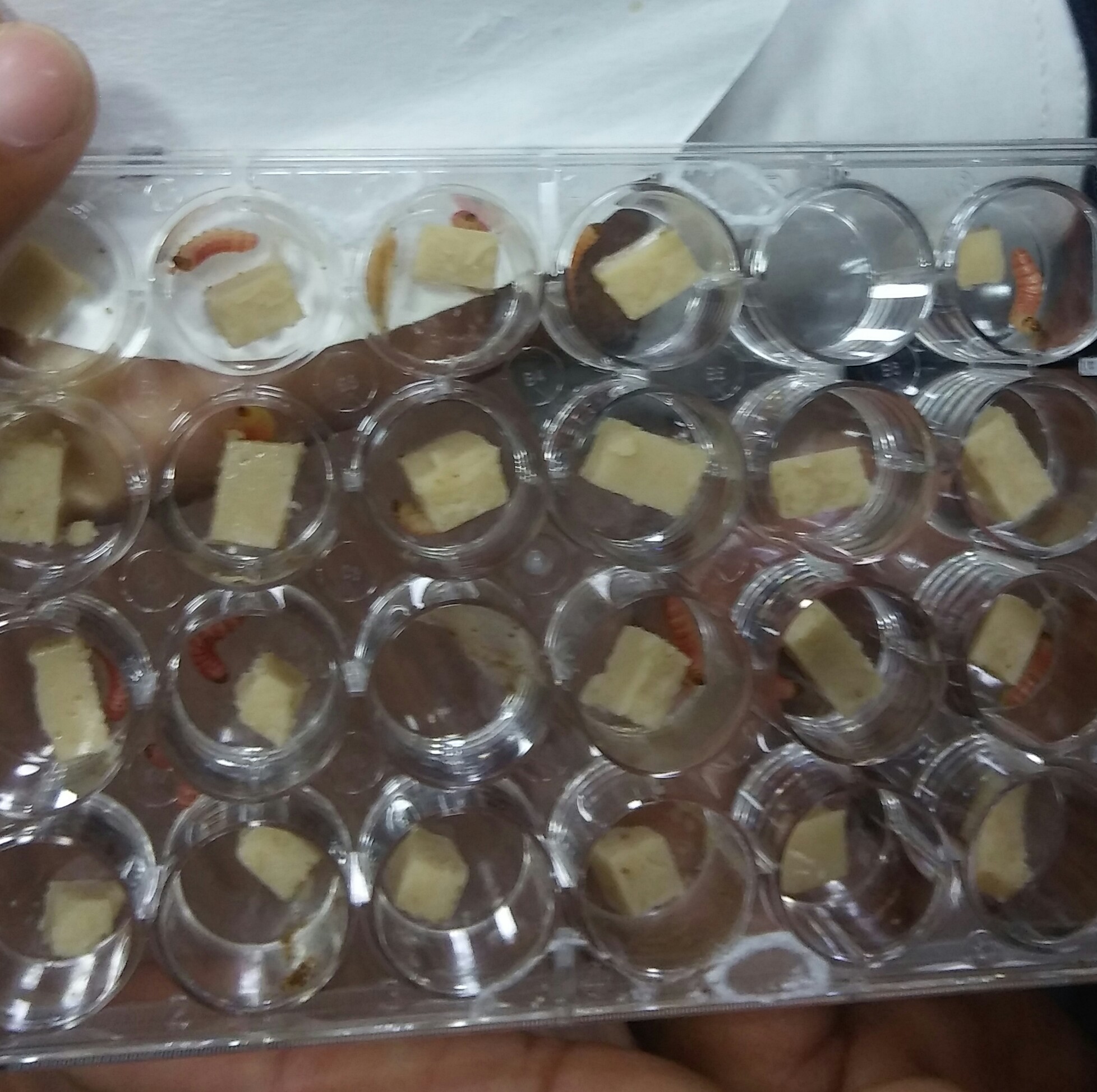


**Pink Bollworm larvae released into the 24 well culture plates.**

**Procedure for Experiment 2: Evaluate the effect of four different temperature ranges (10, 20, 30 and 40°C) on diapausing behavior and other biological parameters of pink bollworm**

The experiment was conducted to evaluate the effect of four different temperature ranges (10, 20, 30 and 40°C) on diapausing behavior and other biological parameters of pink bollworm. These temperatures were maintained in four different incubators. However, the relative humidity (40+10) and photoperiod (14:10 days) was kept constant in all incubators. A standard diet was used to rear 1^st^ instar larvae till last larval instar is near to complete development. The twenty-four last instar larvae along with standard diet was placed in 24 well culture plates. Such 12 culture plate was prepared and grouped into 4 sets each having three culture plates. Each set was used for each temperature exposure and placed under the incubator maintained at the respective temperature. Three culture plate in each set will serve as a replication. The culture plate having diet and larvae was covered with filter paper and lid of the culture plate and tighten with rubber band. Thus prepared set of cultured plates were placed inside the incubation chamber maintained at conducing temperature (27+1°C), relative humidity (65+5) and photoperiod (14:10 hours) and was observed after 15 to 30 days of treatment application. The diapausing (non-pupated larvae) and non-diapausing (pupated larvae) were counted and transformed into percent diapausing larvae. The entire experiment was carried out in CRD with three replications.

**RESULTS**

**Experiment 1: Evaluate eight modified artificial diets and a standard artificial diet for their effects on diapausing-behavior and other biological parameters of pink bollworm**

**Diets effect on number of diapausing larvae of *Pectinophora gossypiella***

ANOVA table showed that all the tested diets (treatments) had statistically significant effect (P>0.05) on diapausing larvae of *Pectinophora gossypiella* (Table 4.2).

Results of present trial showed that maximum number of diapausing larvae (15.01 larvae) was recorded on diet-1 having maximum concentration of maize oil (5.3 ml/L), wheat germ (44.5 g/kg), casein (40 g/kg), sucrose (14 g/kg), and brewer’s yeast (9.0 g/kg). The diet-2 having maize oil (4.3 ml/L), wheat germ (39.5 g/kg), casein (35 g/kg), sucrose (12 g/kg), and brewer’s yeast (7.0 g/kg) concentration less than diet-1 exhibited 12.01 larvae diapausing larvae which were statistically similar to diet-3 (11.01 larvae) having maize oil (2.3 ml/L), wheat germ (29.5 g/kg), casein (25 g/kg), sucrose (08 g/kg), and brewer’s yeast (5.0 g/kg) less than diet-2 but significantly different from all other diets. The diets-7, diet-8 and diet-9 which were vitamin based had non-significant effect on the diapausing behaviour of the pink bollworm and exhibited statistically similar number of diapausing larvae i.e., 6.01 larvae, 5.01 larvae and 4.01 larvae, respectively. The diet-4 (1.3 ml/L), wheat germ (24.5 g/kg), casein (20 g/kg), sucrose (06 g/kg), and brewer’s yeast (3.0 g/kg), diet-5 (0.3 ml/L), wheat germ (19.5 g/kg), casein (15 g/kg), sucrose (04 g/kg), and brewer’s yeast (1.0 g/kg) and diet-6 (3.3 ml/L), wheat germ (34.5 g/kg), casein (30 g/kg), sucrose (10 g/kg), and brewer’s yeast (5.0 g/kg) exhibited statistically similar number of diapausing larvae i.e., (9.01 larvae), (8.01 larvae) and (7.01 larvae), respectively (Fig 4.1).

**Table: 4.2 ANOVA Parameters regarding effect of different diets on number of diapausing larvae of *Pectinophora gossypiella*.**

| SOV | *df* | SS | *MSS* | F | P |
| --- | --- | --- | --- | --- | --- |
| Treatment | 8 | 306.66 | *38.33* | 38.33 | 0.00 |
| Error | 18 | 18.00 | *1.00* |  |  |
| Total | 26 | 324.66 |  |  |  |

Grand Mean 8.5689 CV 5.67

**Figure: 4.1. Means number of diapausing larvae of *P. gossypiella* fed on different tested diets**

**Effect of different diets on number of non-diapausing larvae of *Pectinophora gossypiella*.**

ANOVA table (4.3) showed that all the tested diets (treatments) had statistically significant effect (P>0.05) on non-diapausing larvae of *Pectinophora gossypiella*.

Results of present trial revealed that maximum number of non-diapausing larvae (20.01 larvae) was recorded on diet-9 having minimum concentration of vitamins-III (0.0025 g/kg), wheat germ (34.5 g/kg), casein (30 g/kg), sucrose (10 g/kg), and brewer’s yeast (9.0 g/kg). The diet-8 vitamin based having maximum concentration of vitamin (0.005g/kg) wheat germ (34.5 g/kg), casein (30 g/kg), sucrose (10 g/kg), and brewer’s yeast (9.0 g/kg) less than diet-9 exhibited (19.01 larvae) which were significant statistically similar to diet-9 and diet-7 having vitamin (0.002kg), wheat germ (34.5 g/kg), casein (30 g/kg), sucrose (10 g/kg), and brewer’s yeast (9.0 g/kg). The diet-2 having maize oil (4.3 ml/L), wheat germ (39.5 g/kg), casein (35 g/kg), sucrose (12 g/kg), and brewer’s yeast (7.0 g/kg) having concentration less than exhibited 12.01 non-diapausing larvae which were statistically similar to less than diet-3 (11.01 larvae) having maize oil (2.3 ml/L), wheat germ (29.5 g/kg), casein (25 g/kg), sucrose (08 g/kg), and brewer’s yeast (5.0 g/kg) (13.01larvae). The diet-6 (3.3 ml/L), wheat germ (34.5 g/kg), casein (30 g/kg), sucrose (10 g/kg), and brewer’s yeast (5.0 g/kg) and diet-7 vitamin (0.002kg), wheat germ (34.5 g/kg), casein (30 g/kg), sucrose (10 g/kg), and brewer’s yeast (9.0 g/kg) exhibited statistically non-significant and similar number of diapausing larvae i.e., (17.01 larvae) and (18.01 larvae), respectively. The diet-4 (1.3 ml/L), wheat germ (24.5 g/kg), casein (20 g/kg), sucrose (06 g/kg), and brewer’s yeast (3.0 g/kg) and diet-5 (0.3 ml/L), wheat germ (19.5 g/kg), casein (15 g/kg), sucrose (04 g/kg), and brewer’s yeast showed similar number of non-diapausing larvae i.e., (15.01 larvae) and (16.01 larvae), respectively (Figure 4.2).

**Table: 4.3: ANOVA Parameters regarding effect of different diets on number non-diapausing larvae of *Pectinophora gossypiella*.**

| **SOV** | ***df*** | **SS** | **MSS** | **F** | **P** |
| --- | --- | --- | --- | --- | --- |
| Treatment | 8 | 306.66 | 38.33 | 38.33 | 0.00 |
| Error | 18 | 18.00 | 1.00 |  |  |
| Total | 26 | 324.66 |  |  |  |

Grand Mean 15.458 CV 6.47

**Figure: 4.2. Means number of Non-diapausing larvae of *Pectinophora gossypiella*fed on different tested diets.**

**Effect of different diets on survival rate of diapausing larvae to pupae (*Pectinophora gossypiella*).**

ANOVA table (4.4) showed that all the tested diets (treatments) had statistically significant effect (P>0.05) on survival rate of diapausing larvae to pupae of *Pectinophora gossypiella*.

Results of present experiment revealed that maximum survival rate of diapausing larvae to pupae (59.98%) was recorded on diet-8 having minimum concentration of vitamins-II having maximum concentration of vitamin (0.005g/kg) wheat germ (34.5 g/kg), casein (30 g/kg), sucrose (10 g/kg), and brewer’s yeast (9.0 g/kg). The diet-9 having concentration of vitamin (0.0025 g/kg) and wheat germ (34.5 g/kg), casein (30 g/kg), sucrose (10 g/kg), and brewer’s yeast (9.0 g/kg) exhibited 49.98% survival rate of diapausing larvae to pupae which is less than diet-8, but significant statistically. The diet-4 (1.3 ml/L), wheat germ (24.5 g/kg), casein (20 g/kg), sucrose (06 g/kg), and brewer’s yeast (3.0 g/kg) diet-7 having vitamin (0.002kg), wheat germ (34.5 g/kg), casein (30 g/kg), sucrose (10 g/kg), and brewer’s yeast (9.0 g/kg) showed similar survival rate from diapausing larvae to pupae i.e., (33.31 %) and (33.31 %) which were non-significant statistically, respectively. The diet-1 having maximum concentration of maize oil (5.3 ml/L), wheat germ (44.5 g/kg), casein (40 g/kg), sucrose (14 g/kg), and brewer’s yeast (9.0 g/kg), diet-2 having maize oil (4.3 ml/L), wheat germ (39.5 g/kg), casein (35 g/kg), sucrose (12 g/kg), and brewer’s yeast (7.0 g/kg) and diet-5 (0.3 ml/L), wheat germ (19.5 g/kg), casein (15 g/kg), sucrose (04 g/kg), and brewer’s yeast (5 g/kg) exhibited statistically non-significant and similar survival rate of diapausing larvae to pupae i.e., (53.313 %) and (49.98 %) and (49.98%), respectively but significantly different from all other diets. The diet-3 having maize oil (2.3 ml/L), wheat germ (29.5 g/kg), casein (25 g/kg), sucrose (08 g/kg), and brewer’s yeast (5.0 g/kg) and diet-6 (3.3 ml/L), wheat germ (34.5 g/kg), casein (30 g/kg), sucrose (10 g/kg), and brewer’s yeast (5.0 g/kg) were statistically non-significant showed survival rate of diapausing larvae i.e., (45.44 %) and (42.84%), respectively (Fig 4.3).

**Table: 4.4: ANOVA Parameters regarding effect of different diets on survival rate of diapausing larvae to pupae of *Pectinophora gossypiella*.**

| **SOV** | ***df*** | **SS** | **MS** | **F** | **P** |
| --- | --- | --- | --- | --- | --- |
| **Treatment** | 8 | 1880.33 | 235.04 | 110 | 0.00** |
| **Error** | 18 | 38.37 | 2.13 |  |  |
| **Total** | 26 | 1918.70 |  |  |  |

Grand Mean 46.459 CV 3.14

**Figure: 4.3. Means of survival rate of diapausing larvae to pupae of *Pectinophora gossypiella*fed on different tested diets.**

**Effect of different diets on survival rate of diapausing pupae to adult of *Pectinophora gossypiella*.**

ANOVA table (4.5) showed that all the tested diets (treatments) had statistically significant effect (P>0.05) on survival rate of diapausing pupae to adult of *Pectinophora gossypiella*.

Results of present investigate revealed that maximum survival rate of diapausing pupae to adult (79.98%) was recorded on diet-3 having maize oil (2.3 ml/L), wheat germ (29.5 g/kg), casein (25 g/kg), sucrose (08 g/kg), and brewer’s yeast (5.0 g/kg). The survival rate of diapausing pupae to adult on diet-5 (74.98%) maize oil (0.3 ml/L), wheat germ (19.5 g/kg), casein (15 g/kg), sucrose (04 g/kg), and brewer’s yeast which were less than diet-3. The diet-1 having maximum concentration of maize oil (5.3 ml/L), wheat germ (44.5 g/kg), casein (40 g/kg), sucrose (14 g/kg), and brewer’s yeast (9.0 g/kg), diet-4 (1.3 ml/L), wheat germ (24.5 g/kg), casein (20 g/kg), sucrose (06 g/kg), and brewer’s yeast (3.0 g/kg) and diet-6 (3.3 ml/L), wheat germ (34.5 g/kg), casein (30 g/kg), sucrose (10 g/kg), and brewer’s yeast (5.0 g/kg) were statistically non-significant and showed survival rate of diapausing pupae to adult i.e., (64.48 %),(66.65%) and (66.65%), respectively, but statistically different from diet-2 (49.98%) having maize oil (4.3 ml/L), wheat germ (39.5 g/kg), casein (35 g/kg), sucrose (12 g/kg), and brewer’s yeast (7.0 g/kg). The diet-7 having vitamin (0.002kg), wheat germ (34.5 g/kg), casein (30 g/kg), sucrose (10 g/kg), and brewer’s yeast (9.0 g/kg) and diet-9 having concentration of vitamin (0.0025 g/kg) and wheat germ (34.5 g/kg), casein (30 g/kg), sucrose (10 g/kg), and brewer’s yeast (9.0 g/kg) showed statistically non-significant results and similar survival rate of diapausing pupae to adult i.e., (49.98%) and (49.98 %) respectively, different statistically from diet-8 (66.65%) having minimum concentration of vitamins-II having maximum concentration of vitamin (0.005g/kg) wheat germ (34.5 g/kg), casein (30 g/kg), sucrose (10 g/kg), and brewer’s yeast (9.0 g/kg) (Fig 4.4).

**Table: 4.5: ANOVA Parameters regarding effect of different diets on survival rate of diapausing pupae to adult of *Pectinophora gossypiella*.**

| **SOV** | ***df*** | **SS** | **MS** | **F** | **P** |
| --- | --- | --- | --- | --- | --- |
| **Treatment** | 8 | 2941.67 | 367.70 | 172 | 0.00** |
| **Error** | 18 | 38.37 | 2.13 |  |  |
| **Total** | 26 | 2980.04 |  |  |  |

Grand Mean 46.459 CV 3.14

**Figure: 4.4. Means of survival rate of diapausing pupae to adult of *Pectinophora gossypiella* fed on different tested diets**.

**Effect of different diets on survival rate of Non-diapausing of larvae to pupae of *Pectinophora gossypiella***

ANOVA table (4.6) showed that all the tested diets (treatments) had statistically significant effect (P>0.05) on survival rate of non-diapausing larvae to pupae of *Pectinophora gossypiella*.

Results of present study indicated that maximum survival rate of non-diapausing larvae to pupae (66.65%) was recorded on diet-1 having maximum concentration of maize oil (5.3 ml/L), wheat germ (44.5 g/kg), casein (40 g/kg), sucrose (14 g/kg), and brewer’s yeast (9.0 g/kg). The diet-2 having maize oil (4.3 ml/L), wheat germ (39.5 g/kg), casein (35 g/kg), sucrose (12 g/kg), and brewer’s yeast (7.0 g/kg), diet-4 (1.3 ml/L), wheat germ (24.5 g/kg), casein (20 g/kg), sucrose (06 g/kg), and brewer’s yeast (3.0 g/kg) and diet-6 (3.3 ml/L), wheat germ (34.5 g/kg), casein (30 g/kg), sucrose (10 g/kg), and brewer’s yeast (5.0 g/kg) were statistically non-significant and showed survival rate of non-diapausing larvae to pupae i.e., (58.31%) (53.31%) and (58.80%), respectively, but statistically different from diet-3 (38.98%) having maize oil (2.3 ml/L), wheat germ (29.5 g/kg), casein (25 g/kg), sucrose (08 g/kg), and brewer’s yeast (5.0 g/kg). The diet-8 (66.65%) having minimum concentration of vitamins-II having maximum concentration of vitamin (0.005g/kg) wheat germ (34.5 g/kg), casein (30 g/kg), sucrose (10 g/kg), and brewer’s yeast (9.0 g/kg) and diet-9 having concentration of vitamin (0.0025 g/kg) and wheat germ (34.5 g/kg), casein (30 g/kg), sucrose (10 g/kg), and brewer’s yeast (9.0 g/kg) showed statistically non-significant results and similar survival rate of non-diapausing larvae to pupae i.e., (57.88%) and (49.98 %) respectively, but different statistically from diet-7 (49.98%) having vitamin (0.002kg), wheat germ (34.5 g/kg), casein (30 g/kg), sucrose (10 g/kg), and brewer’s yeast (9.0 g/kg) (Fig 4.5).

**Table: 4.6: ANOVA Parameters regarding effect of different diets on survival rate of Non-diapausing of larvae to pupae of *Pectinophora gossypiella***

| **SOV** | ***df*** | **SS** | **MS** | **F** | **P** |
| --- | --- | --- | --- | --- | --- |
| **Treatment** | 8 | 1486.68 | 185.83 | 87.2 | 0.00** |
| **Error** | 18 | 38.37 | 2.13 |  |  |
| **Total** | 26 | 1525.06 |  |  |  |

Grand Mean 55.509 CV 2.63

**Figure: 4.5: Means non-diapausing of larvae to pupae of *Pectinophora gossypiella*fed on different tested diets.**

**Effect of different diets on survival rate of Non-diapausing pupae to adult of *Pectinophora gossypiella***

ANOVA table (4.7) showed that all the tested diets (treatments) had statistically significant effect (P>0.05) on survival rate of diapausing pupae to adult of *Pectinophora gossypiella*.

Results of present study showed that maximum survival rate of non-diapausing pupae to adult (79.98%) was recorded on diet-2 (83.69%) having maize oil (4.3 ml/L), wheat germ (39.5 g/kg), casein (35 g/kg), sucrose (12 g/kg), and brewer’s yeast (7.0 g/kg). The survival rate of non- diapausing pupae to adult on diet-1 having maximum concentration of maize oil (5.3 ml/L), wheat germ (44.5 g/kg), casein (40 g/kg), sucrose (14 g/kg), and brewer’s yeast (9.0 g/kg) and diet-3 having maize oil (2.3 ml/L), wheat germ (29.5 g/kg), casein (25 g/kg), sucrose (08 g/kg) were (83.83%) and (59.98%), respectively, but different statistically from diet-6 (79.98%).The diet-8 having minimum concentration of vitamins-II having maximum concentration of vitamin (0.005g/kg) wheat germ (34.5 g/kg), casein (30 g/kg), sucrose (10 g/kg), and brewer’s yeast (9.0 g/kg) and diet-9 having concentration of vitamin (0.0025 g/kg) and wheat germ (34.5 g/kg), casein (30 g/kg), sucrose (10 g/kg), and brewer’s yeast (9.0 g/kg) showed statistically non-significant results and similar survival rate of non-diapausing pupae adult i.e., (837.88%) and (81.80 %) respectively, but different statistically from diet-7 (77.76%) having vitamin (0.002kg), wheat germ (34.5 g/kg), casein (30 g/kg), sucrose (10 g/kg), and brewer’s yeast (9.0 g/kg) (Fig 4.6).

**Table: 4.7: ANOVA Parameters regarding effect of different diets on survival rate of Non-diapausing pupae to adult of *Pectinophora gossypiella*.**

| **SOV** | ***df*** | **SS** | **MS** | **F** | **P** |
| --- | --- | --- | --- | --- | --- |
| **Treatment** | 8 | 1401.70 | 175.21 | 82.2 | 0.00** |
| **Error** | 18 | 38.37 | 2.13 |  |  |
| **Total** | 26 | 1440.07 |  |  |  |

Grand Mean 78.286 CV 1.87

**Figure: 4.6: Means survival rate of non-diapausing pupae to adult of *Pectinophora gossypiella*fed on different tested diets.**

**Effect of different diets on weight of larvae of *Pectinophora gossypiella***

ANOVA table (4.8) showed that all the tested diets (treatments) had statistically significant effect at P>0.05 on weight of larvae of *Pectinophora gossypiella*.

Results of present investigate exhibited that maximum weight of the larvae (23.01 mg) was noted on diet-6 (3.3 ml/L), wheat germ (34.5 g/kg), casein (30 g/kg), sucrose (10 g/kg), and brewer’s yeast (5.0 g/kg). The diet-3 (38.98%) having maize oil (2.3 ml/L), wheat germ (29.5 g/kg), casein (25 g/kg), sucrose (08 g/kg), and brewer’s yeast (5.0 g/kg) exhibited (22.01mg) weight of the larvae less than diet-6 but, statistically significant. The diet-1 having maximum concentration of maize oil (5.3 ml/L), wheat germ (44.5 g/kg), casein (40 g/kg), sucrose (14 g/kg), and brewer’s yeast (9.0 g/kg) and diet-2 having maize oil (4.3 ml/L), wheat germ (39.5 g/kg), casein (35 g/kg), sucrose (12 g/kg), and brewer’s yeast (7.0 g/kg) indicated weight of the larvae i.e., 21.01 mg and 21.01 respectively which were non-significant but statistically significant from diet-4 (1.3 ml/L), wheat germ (24.5 g/kg), casein (20 g/kg), sucrose (06 g/kg), and brewer’s yeast (3.0 g/kg) and diet-5 (0.3 ml/L), wheat germ (19.5 g/kg), casein (15 g/kg), sucrose (04 g/kg), and brewer’s yeast (5.g/kg) exhibited weight of the larvae (17.01 mg). The diet-7 (49.98%) having vitamin (0.002kg), wheat germ (34.5 g/kg), casein (30 g/kg), sucrose (10 g/kg), and brewer’s yeast (9.0 g/kg) and diet-1 having maximum concentration of maize oil (5.3 ml/L), wheat germ (44.5 g/kg), casein (40 g/kg), sucrose (14 g/kg), and brewer’s yeast (9.0 g/kg). The diet-2 having maize oil (4.3 ml/L), wheat germ (39.5 g/kg), casein (35 g/kg), sucrose (12 g/kg), and brewer’s yeast (7.0 g/kg) and diet-9 having concentration of vitamin (0.0025 g/kg) and wheat germ (34.5 g/kg), casein (30 g/kg), sucrose (10 g/kg), and brewer’s yeast (9.0 g/kg) exhibited weight of the larvae i.e., (19.01%) (20.01%) which were statistically non-significant and statistically significant from diet-8 (66.65%) having minimum concentration of vitamins-II having maximum concentration of vitamin (0.005g/kg) wheat germ (34.5 g/kg), casein (30 g/kg), sucrose (10 g/kg), and brewer’s yeast (9.0 g/kg) (21.01%) (Fig 4.7).

**Table: 4.8: ANOVA Parameters regarding effect of different diets on larval weight (mg) of *Pectinophora gossypiella*.**

| **SOV** | ***df*** | **SS** | **MSS** | **F** | **P** |
| --- | --- | --- | --- | --- | --- |
| Treatment | 8 | 86.66 | 10.83 | 10.8 | 0.00** |
| Error | 18 | 18.00 | 1.00 |  |  |
| Total | 26 | 104.66 |  |  |  |

Grand Mean 20.124 CV 4.97

**Figure: 4.7 Means larval weight (mg) of *Pectinophora gossypiella*fed on different tested diets.**

**Effect of different diets on pupal weight of *Pectinophora gossypiella*.**

ANOVA table (4.9) showed that all the tested diets (treatments) had statistically significant at P>0.05 on weight of the pupae of *Pectinophora gossypiella*.

Results of present study showed that maximum weight of the pupae (21.15mg) was recorded on diet-5 (0.3 ml/L), wheat germ (19.5 g/kg), casein (15 g/kg), sucrose (04 g/kg), and brewer’s yeast (5.g/kg). The diet-1 having maximum concentration of maize oil (5.3 ml/L), wheat germ (44.5 g/kg), casein (40 g/kg), sucrose (14 g/kg), and brewer’s yeast (9.0 g/kg) and diet-2 having maize oil (4.3 ml/L), wheat germ (39.5 g/kg), casein (35 g/kg), sucrose (12 g/kg), and brewer’s yeast (7.0 g/kg) indicated weight of the pupae i.e., 19.01 mg and 18.01 respectively which were non-significant but statistically significant. diet-3 having maize oil (2.3 ml/L), wheat germ (29.5 g/kg), casein (25 g/kg), sucrose (08 g/kg), and brewer’s yeast (5.0 g/kg) and diet-6 (3.3 ml/L), wheat germ (34.5 g/kg), casein (30 g/kg), sucrose (10 g/kg), and brewer’s yeast (5.0 g/kg) revealed i. e., 16.01mg and 17.01 mg respectively and statistically non-significant. The diet-7 having vitamin (0.002kg), wheat germ (34.5 g/kg), casein (30 g/kg), sucrose (10 g/kg), and brewer’s yeast (9.0 g/kg) and diet-9 having concentration of vitamin (0.0025 g/kg) and wheat germ (34.5 g/kg), casein (30 g/kg), sucrose (10 g/kg), and brewer’s yeast (9.0 g/kg) exhibited weight of the pupae i.e., 16.01mg and 16.01mg which were statistically non-significant and statistically significant from diet-8 having minimum concentration of vitamins-II having maximum concentration of vitamin (0.005g/kg) wheat germ (34.5 g/kg), casein (30 g/kg), sucrose (10 g/kg), and brewer’s yeast (9.0 g/kg) showed (18.01mg) (Fig 4.8).

T**able: 4.9: ANOVA Parameters regarding effect of different diets on Pupal weight (mg) of *Pectinophora gossypiella*.**

| SOV | *Df* | SS | MSS | F | P |
| --- | --- | --- | --- | --- | --- |
| Treatment | 8 | 80.66 | 10.083 | 10.1 | 0.00** |
| Error | 18 | 18.00 | 1.00 |  |  |
| Total | 26 | 98.66 |  |  |  |

Grand Mean 17.902 CV 5.59

**Figure: 4.8: Means pupal weight (mg) of *Pectinophora gossypiella*fed on different tested diets on day intervals.**

**Effect of different diets on life span of *Pectinophora gossypiella* male**

ANOVA table (4.10) showed that all the tested diets (treatments) were statistically significant at P>0.05 on male days’ adult of *Pectinophora gossypiella*.

Results of present experiment results showed that maximum male days of adult (10.01days) was observed on diet-2 having maize oil (4.3 ml/L), wheat germ (39.5 g/kg), casein (35 g/kg), sucrose (12 g/kg), and brewer’s yeast (7.0 g/kg) but statistically similar to diet-1 having maximum concentration of maize oil (5.3 ml/L), wheat germ (44.5 g/kg), casein (40 g/kg), sucrose (14 g/kg), and brewer’s yeast (9.0 g/kg) which exhibited (9.01 days). The diet-3 having maize oil (2.3 ml/L), wheat germ (29.5 g/kg), casein (25 g/kg), sucrose (08 g/kg), diet-5 (0.3 ml/L), wheat germ (19.5 g/kg), casein (15 g/kg), sucrose (04 g/kg), and brewer’s yeast (5.g/kg) and diet-6 (3.3 ml/L), wheat germ (34.5 g/kg), casein (30 g/kg), sucrose (10 g/kg), and brewer’s yeast (5.0 g/kg) exhibited) i. e. 7.01 days 7.01days and 7.01 days respectively and statistically similar and non-significant but different from diet-4 (1.3 ml/L), wheat germ (24.5 g/kg), casein (20 g/kg), sucrose (06 g/kg), and brewer’s yeast (3.0 g/kg) which exhibited (8.01 days). The diet-8 having minimum concentration of vitamins-II having maximum concentration of vitamin (0.005g/kg) wheat germ (34.5 g/kg), casein (30 g/kg), sucrose (10 g/kg), and brewer’s yeast (9.0 g/kg) and diet-9 having concentration of vitamin (0.0025 g/kg) and wheat germ (34.5 g/kg), casein (30 g/kg), sucrose (10 g/kg), and brewer’s yeast (9.0 g/kg) exhibited male days of adult the i.e., 9.01 days and 8.01mg which were similar and statistically non-significant but statistically significant from diet-7 having vitamin (0.002kg), wheat germ (34.5 g/kg), casein (30 g/kg), sucrose (10 g/kg), and brewer’s yeast (9.0 g/kg) exhibited 10.01 days (Fig 4.9).

**Table: 4.10: ANOVA Parameters regarding effect of different diets on life span (days) of *Pectinophora gossypiella* male.**

| **SOV** | **DF** | **SS** | **MSS** | **F** | **P** |
| --- | --- | --- | --- | --- | --- |
| Treatment | 8 | 36.00 | 4.50 | 4.5 | 0.00** |
| Error | 18 | 18.00 | 1.00 |  |  |
| Total | 26 | 54.00 |  |  |  |

Grand Mean 8.0133 CV 4.48

**Figure: 4.9: Means life span (days) of *Pectinophora gossypiella* male on different tested diets.**

**Effect of different diets on life span of *Pectinophora gossypiella* female**

ANOVA table (4.11) showed that all the tested diets (treatments) had statistically significant at P>0.05 on female days of *Pectinophora gossypiella*.

Results of present research results showed that maximum life span of *Pectinophora gossypiella* female (10.01days) was observed on diet-2 having maize oil (4.3 ml/L), wheat germ (39.5 g/kg), casein (35 g/kg), sucrose (12 g/kg), and brewer’s yeast (7.0 g/kg). The diet-1 having maximum concentration of maize oil (5.3 ml/L), wheat germ (44.5 g/kg), casein (40 g/kg), sucrose (14 g/kg), and brewer’s yeast (9.0 g/kg) which exhibited (9.01 days), diet-4 (1.3 ml/L), wheat germ (24.5 g/kg), casein (20 g/kg), sucrose (06 g/kg), and brewer’s yeast (3.0 g/kg) and diet-6 (3.3 ml/L), wheat germ (34.5 g/kg), casein (30 g/kg), sucrose (10 g/kg), and brewer’s yeast (5.0 g/kg) exhibited) i. e., 9.01 days, 8.01 days and 9.01 days respectively and statistically similar and non-significant but different from diet-2 having maize oil (4.3 ml/L), wheat germ (39.5 g/kg), casein (35 g/kg), sucrose (12 g/kg), and brewer’s yeast (7.0 g/kg) which exhibited (10.01 days). The diet-3 having maize oil (2.3 ml/L), wheat germ (29.5 g/kg), casein (25 g/kg), sucrose (08 g/kg) and diet-5 (0.3 ml/L), wheat germ (19.5 g/kg), casein (15 g/kg), sucrose (04 g/kg), and brewer’s yeast (5.g/kg) and diet-6 (3.3 ml/L), wheat germ (34.5 g/kg), casein (30 g/kg), sucrose (10 g/kg), and brewer’s yeast (5.0 g/kg) exhibited) i. e. 7.01 days and 7.01 days respectively and statistically similar and non-significant. The diet-8 having minimum concentration of vitamins-II having maximum concentration of vitamin (0.005g/kg) wheat germ (34.5 g/kg), casein (30 g/kg), sucrose (10 g/kg), and brewer’s yeast (9.0 g/kg) and diet-9 having concentration of vitamin (0.0025 g/kg) and wheat germ (34.5 g/kg), casein (30 g/kg), sucrose (10 g/kg), and brewer’s yeast (9.0 g/kg) exhibited male days of adult the i.e., 9.01 days and 8.01mg which were similar and statistically non-significant but statistically significant from diet-7 having vitamin (0.002kg), wheat germ (34.5 g/kg), casein (30 g/kg), sucrose (10 g/kg), and brewer’s yeast (9.0 g/kg) exhibited 10.01 days (Fig 4.10).

**Table: 4.11: ANOVA Parameters regarding effect of different diets on life span (days) of *Pectinophora gossypiella* female.**

| **SOV** | ***df*** | **SS** | **MSS** | **F** | **P** |
| --- | --- | --- | --- | --- | --- |
| Treatment | 8 | 30.66 | 3.83 | 3.83 | 0.00** |
| Error | 18 | 18.00 | 1.00 |  |  |
| Total | 26 | 48.66 |  |  |  |

Grand Mean 8.5689 CV 6.67

**Figure: 4.10: Means life span (days) of *Pectinophora gossypiella* female on different tested diets.**

**Effect of different diets on fecundity of *Pectinophora gossypiella***

ANOVA table (4.12) showed that all the tested diets (treatments) had statistically significant at P>0.05 on fecundity of *Pectinophora gossypiella*.

Results of present investigation demonstrated that maximum fecundity rate (150.01 eggs) was recorded on diet-1 having maximum concentration of maize oil (5.3 ml/L), wheat germ (44.5 g/kg), casein (40 g/kg), sucrose (14 g/kg), and brewer’s yeast (9.0 g/kg) which were statistically significant from diet-4 (1.3 ml/L), wheat germ (24.5 g/kg), casein (20 g/kg), sucrose (06 g/kg), and brewer’s yeast (1.0 g/kg) (133.01 eggs). The diet-3 exhibited (120.01 eggs) having maize oil (2.3 ml/L), wheat germ (29.5 g/kg), casein (25 g/kg), sucrose (08 g/kg) and brewer’s yeast (3g/kg) statistically different from diet-5 (0.3 ml/L), wheat germ (19.5 g/kg), casein (15 g/kg), sucrose (04 g/kg), and brewer’s yeast (5.g/kg). The diet-2 having maize oil (4.3 ml/L), wheat germ (39.5 g/kg), casein (35 g/kg), sucrose (12 g/kg), and brewer’s yeast (7.0 g/kg) and diet-6 (3.3 ml/L), wheat germ (34.5 g/kg), casein (30 g/kg), sucrose (10 g/kg), and brewer’s yeast (5.0 g/kg) exhibited) i. e. 112.01 eggs and 115.01 eggs respectively and statistically significant. The diet-7 (49.98%) having vitamin (0.002kg), wheat germ (34.5 g/kg), casein (30 g/kg), sucrose (10 g/kg), and brewer’s yeast (9.0 g/kg), diet-8 having minimum concentration of vitamins-II having maximum concentration of vitamin (0.005g/kg) wheat germ (34.5 g/kg), casein (30 g/kg), sucrose (10 g/kg), and brewer’s yeast (9.0 g/kg) and diet-9 having concentration of vitamin (0.0025 g/kg) and wheat germ (34.5 g/kg), casein (30 g/kg), sucrose (10 g/kg), and brewer’s yeast (9.0 g/kg) exhibited a fecundity of 122.01 egg, 126.01 eggs and 136 eggs, respecively which were statistically significant (Fig 4.11).

**Table: 4.12: ANOVA Parameters regarding effect of different diets on fecundity of *Pectinophora gossypiella*.**

| **SOV** | ***df*** | **SS** | **MSS** | **F** | **P** |
| --- | --- | --- | --- | --- | --- |
| Treatment | 8 | 3296.67 | 412.83 | 412 | 0.00** |
| Error | 18 | 18.00 | 1.00 |  |  |
| Total | 26 | 3314.67 |  |  |  |

Grand Mean 127.12 CV 0.79

**Figure: 4.11: Means fecundity of *Pectinophora gossypiella* on different tested diets.**

**Effect of different diets on total life cycle of *Pectinophora gossypiella*.**

ANOVA table (4.13) showed that all the tested diets (treatments) had statistically significant at P>0.05 on total life cycle of *Pectinophora gossypiella*.

Results of present search proved that maximum total life cycle (47.01 days) was recorded on diet-4 (1.3 ml/L), wheat germ (24.5 g/kg), casein (20 g/kg), sucrose (06 g/kg), and brewer’s yeast (1.0 g/kg) which were statically significant to diet-3 exhibited (120.01 eggs) having maize oil (2.3 ml/L), wheat germ (29.5 g/kg), casein (25 g/kg), sucrose (08 g/kg) and brewer’s yeast (3g/kg) exhibited (46.01 days). The diet-1 having maximum concentration of maize oil (5.3 ml/L), wheat germ (44.5 g/kg), casein (40 g/kg), sucrose (14 g/kg), and brewer’s yeast (9.0 g/kg) and diet-2 having maize oil (4.3 ml/L), wheat germ (39.5 g/kg), casein (35 g/kg), sucrose (12 g/kg), and brewer’s yeast (7.0 g/kg) exhibited total life cycle of 40.01 days and 42.01 days, respectively which were statistically significant. The diet-5 (0.3 ml/L), wheat germ (19.5 g/kg), casein (15 g/kg), sucrose (04 g/kg), and brewer’s yeast (5.g/kg) and diet-6 (3.3 ml/L), wheat germ (34.5 g/kg), casein (30 g/kg), sucrose (10 g/kg), and brewer’s yeast (5.0 g/kg) exhibited total life cycle of 40.01 days and 41.01 days, respectively which were statically non-significant but different from all other diets. The diet-8 having minimum concentration of vitamins-II having maximum concentration of vitamin (0.005g/kg) wheat germ (34.5 g/kg), casein (30 g/kg), sucrose (10 g/kg), and brewer’s yeast (9.0 g/kg) and diet-9 having concentration of vitamin (0.0025 g/kg) and wheat germ (34.5 g/kg), casein (30 g/kg), sucrose (10 g/kg), and brewer’s yeast (9.0 g/kg) exhibited total life cycle of 43.01 days and 44.01 days, respectively which were similar and statistically non-significant but statistically significant from diet-7 having vitamin (0.002kg), wheat germ (34.5 g/kg), casein (30 g/kg), sucrose (10 g/kg), and brewer’s yeast (9.0 g/kg) exhibited 39.01 days (Fig 4.12).

**Table: 4.13: ANOVA Parameters regarding effect of different diets on total life cycle of *Pectinophora gossypiella*.**

| **SOV** | ***df*** | **SS** | **MSS** | **F** | **P** |
| --- | --- | --- | --- | --- | --- |
| Treatment | 8 | 186.67 | 23.33 | 23.3 | 0.00** |
| Error | 18 | 18.00 | 1.00 |  |  |
| Total | 26 | 204.67 |  |  |  |

Grand Mean 127.12 CV 0.79

**Figure: 4.12: Means total life cycle (days) of *Pectinophora gossypiella* on different tested diets.**

**Experiment 2: Evaluate the effect of four different temperature ranges (10, 20, 30 and 40°C) on diapausing behavior and other biological parameters of pink bollworm**

**Effect of different Temperatures on number diapausing larvae of *Pectinophora gossypiella***

ANOVA table (4.14) showed that all the tested Temperatures (treatments) had statistically significant effect at P>0.05 on diapausing larvae of *Pectinophora gossypiella*.

Results of present investigation exhibited that maximum number of diapausing larvae (24.01 larvae) was recorded at 10°C which was statistically different from number of diapausing larvae recorded at 20°C (13.00 larvae). These results also explained that 10°C decrease in temperature resulted in approximately 1.8 times increase in diapausing larvae of pink bollworm. However, no diapause was recorded at 30°C and 40°C as 100% larvae transformed into pupae (Fig 4.13).

**Table: 4.14: ANOVA Parameters regarding effect of different Temperatures on number diapausing larvae of *Pectinophora gossypiella*.**

| **SOV** | ***df*** | **SS** | **MS** | **F** | **P** |
| --- | --- | --- | --- | --- | --- |
| Temperatures | 3 | 1208.25 | 402.75 | 1611 | 0.00 |
| Error | 8 | 2.00 | .250 |  |  |
| Total | 11 | 1210.25 |  |  |  |

Grand Mean 9.2500 CV 5.41

**Figure: 4.13: Means number of diapausing larvae of *Pectinophora gossypiella* reared on different temperature regimes.**

**Effect of different temperature regimes on number of non-diapausing larvae of *Pectinophora gossypiella*.**

ANOVA table (4.15) showed that all the tested temperature regimes (treatments) had statistically effect significant at P>0.05 on non-diapausing larvae of *Pectinophora gossypiella*.

Results of present investigation exhibited that maximum number of non-diapausing larvae (24.01 larvae) was recorded at 10°C and 30°C which was statistically different from number of non-diapausing larvae recorded at 20°C (11.00 larvae). These results also explained that 10°C increase in temperature resulted in approximately 2.1 times increase in non-diapausing larvae of pink bollworm. However, the non-diapausing was recorded at 10°C (0.00 larvae) (Fig 4.14).

**Table: 4.15: ANOVA Parameters regarding effect of different temperature regimes on non-diapausing behavior larvae of *Pectinophora gossypiella*.**

| **SOV** | ***Df*** | **SS** | **MS** | **F** | **P** |
| --- | --- | --- | --- | --- | --- |
| Temperatures | 3 | 1208.25 | 402.75 | 1611 | 0.00 |
| Error | 8 | 2.00 | 250 |  |  |
| Total | 11 | 1210.25 |  |  |  |

Grand Mean 14.750 CV 3.39

**Figure: 4.14: Means number of non-diapausing larvae of *Pectinophora gossypiella* reared on different temperature regimes.**

**Effect of different temperature regimes on the survival rate of non-diapuasing larvae to pupae *Pectinophora gossypiella*.**

ANOVA table (4.16) showed that all the tested diets (treatments) were statistically significant at P>0.05 on survival rate of non-diapausing larvae to pupae of *Pectinophora gossypiella*.

Results of present trial showed that maximum the survival rate of non-diapuasing larvae to pupae (100.00%) was reported on temperature 30°C and 40°C. Minimum the survival rate of non-diapuasing larvae to pupae were recorded on temperature 20°C which was 26.87%. The survival rate of non-diapuasing larvae to pupae was zero percent at temperature 10°C (Fig 4.15).

**Table: 4.16: ANOVA Parameters regarding effect of of different temperature regimes on the survival rate of non-diapuasing larvae to pupae *Pectinophora gossypiella*.**

| **SOV** | ***df*** | **SS** | **MS** | **F** | **P** |
| --- | --- | --- | --- | --- | --- |
| Temperatures | 3 | 23563.7 | 7854.58 | 705 | 0.00** |
| Error | 8 | 89.1 | 11.14 |  |  |
| Total | 11 | 23652.9 |  |  |  |

Grand Mean 56.717 CV 5.89

**Figure: 4.15: Means of the survival rate of non-diapuasing larvae to pupae of *Pectinophora gossypiella*) reared on different temperature regimes.**

**Effect of different temperature regimes on the survival rate of non-diapuasing pupae to adult of *Pectinophora gossypiella*.**

ANOVA table (4.17) showed that all the tested temperature regimes (treatments) were statistically significant at P>0.05 on survival rate of non-diapuasing pupae to adult of *Pectinophora gossypiella*.

Results of present trial showed that maximum the survival rate of non-diapuasing pupae to adult (100.00%) was reported on temperature 20°C which was statistically different from percentage of non-diapausing larvae recorded at 30°C (84.72%) and 40°C (90.28%). The non-diapausing pupae to adult recorded on 10°C was zero percent (Fig 4.16).

**Table: 4.17: ANOVA Parameters regarding effect of different temperature regimes on the survival rate of non-diapuasing pupae to adult of *Pectinophora gossypiella*.**

| **SOV** | ***df*** | **SS** | **MS** | **F** | **P** |
| --- | --- | --- | --- | --- | --- |
| Temperature | 3 | 19265.0 | 6421.68 | 404 | 0.00** |
| Error | 8 | 127.3 | 15.91 |  |  |
| Total | 11 | 19392.4 |  |  |  |

Grand Mean 68.750 CV 5.80

**Figure: 4.16: Means of the survival rate of non-diapuasing pupae to adult of *Pectinophora gossypiella* reared on different temperature regimes.**

**Effect of different temperature regimes on weight larvae of *Pectinophora gossypiella*.**

ANOVA table (4.18) showed that all the tested temperatures (treatments) had statistically significant effect at P>0.05 on weight larvae of *Pectinophora gossypiella*.

Results of current search revealed that maximum of weight of larvae (21.00 mg) was recorded at 410 °C which was statistically different weight of larvae recorded at 20 °C and 30 °C (12.00 mg) and (20.00 mg). The weight of the larvae recorded at 10 °C was 0.00mg. The obtained result indicated that weight of the larvae was effected with change in the temperature (Fig 4.17).

**Table: 4.18: ANOVA Parameters regarding effect of different temperature regimes on weight (mg) larvae of Pectinophora *gossypiella*.**

| SOV | *df* | SS | MS | F | P |
| --- | --- | --- | --- | --- | --- |
| Temperatures | 3 | 875.00 | 291.667 | 1167 | 0.00 |
| Error | 8 | 2.00 | .250 |  |  |
| Total | 11 | 877.00 |  |  |  |

Grand Mean 13.500 CV 3.70

**Figure: 4.17: Means of weight of *Pectinophora gossypiella* reared on different temperature regimes.**

**Effect of different temperature regimes on pupal weight of *Pectinophora gossypiella*.**

ANOVA table (4.19) showed that all the tested temperature (treatments) were statistically significant at P>0.05 on weight pupae of *Pectinophora gossypiella*.

Results of present study exhibited that maximum of weight of larvae (19.00 mg) was recorded at 40°C which was statistically different weight of pupae recorded at 20°C and 30°C (11.00 mg) and (17.00 mg). The weight of the larvae recorded at 10°C was 0.00mg. The obtained result indicated that weight of the pupae was effected with change in the temperature (Fig. 4.18).

**Table: 4.19: ANOVA Parameters regarding effect of different temperature regimes on weight of the pupae of Pectinophora *gossypiella*.**

| **SOV** | ***df*** | **SS** | **MS** | **F** | **P** |
| --- | --- | --- | --- | --- | --- |
| Temperatures | 3 | 692.66 | 230.88 | 554 | 0.00 |
| Error | 8 | 3.333 | .417 |  |  |
| Total | 11 | 696.00 |  |  |  |

Grand Mean 12.000 CV 5.38

**Figure: 4.18: Means Pupal weight of *Pectinophora gossypiella*) reared on different temperature regimes.**

**Effect of different temperature regimes on life span *Pectinophora gossypiella* male.**

ANOVA table (4.20) showed that all the tested temperatures (treatments) had statistically significant effect at P>0.05 on male days of adult of *Pectinophora gossypiella*.

Results of current search revealed that maximum of male days (8.33 days) was recorded at 30°C which was statistically different weight of pupae recorded at 20°C and 40°C (4.00 days) and (7.67 days). The male days recorded at 10°C was 0.00 days. The obtained result indicated that male days was effected with change in the temperature (Fig. 4.19).

**Table: 4.20: ANOVA Parameters regarding effect of different temperature regimes on life span *Pectinophora gossypiella* male.**

| **SOV** | ***df*** | **SS** | **MS** | **F** | **P** |
| --- | --- | --- | --- | --- | --- |
| Temperatures | 3 | 132.66 | 44.22 | 12.1 | 0.00 |
| Error | 8 | 29.33 | 3.66 |  |  |
| Total | 11 | 162.00 |  |  |  |

Grand Mean 5.0000 CV 3.30

**Figure: 4.19: Means life span (days) of*Pectinophora gossypiella*males reared on different temperature regimes.**

**Effect of different temperature regimes on Life span of *Pectinophora gossypiella* female.**

ANOVA table (4.21) showed that all the tested temperatures (treatments) had statistically significant effect at P>0.05 on female days of the adult of *Pectinophora gossypiella*.

Results of recent trail exhibited that maximum of Life span of *Pectinophora gossypiella* female (9.67 days) was recorded at 40°C which was statistically different life span of *Pectinophora gosssypeilla* female recorded at 20°C and 30°C (4.33 days) and (9.00 days). The life span of *Pectinophora gosssypeilla* femalerecorded at 10°C was 0.00 days. The obtained result indicated that female days was effected with change in the temperature (Fig. 4.20).

**Table: 4.21: ANOVA Parameters regarding effect of different temperature regimes on female days of the adult of *Pectinophora gossypiella* pink bollworm.**

| SOV | *df* | SS | MS | F | P |
| --- | --- | --- | --- | --- | --- |
| Temperatures | 3 | 182.91 | 60.97 | 15.6 | 0.00 |
| Error | 8 | 31.33 | 3.91 |  |  |
| Total | 11 | 214.25 |  |  |  |

Grand Mean 5.7500 CV 4.42

**Figure: 4.20: Means life span of *Pectinophora gosssypeilla* female reared on different temperature regimes**.

**Effect of different temperature regimes on total cycle *Pectinophora gossypiella***.

ANOVA table (4.22) showed that all the tested temperature regimes (treatments) had statistically significant effect at P>0.05 on total life cycle of *Pectinophora gossypiella*.

Results of current investigation revealed that maximum of total life cycle (51.67 days) was recorded at 40°C which was statistically different total life cycle recorded at 20°C and 30°C (47.33 days) and (6.67 days). The female days recorded at 10°C was 0.00 days. The obtained result showed that the total life cycle was effected with change in the temperature (Fig. 4.21).

**Table: 4.22: ANOVA Parameters regarding effect of different temperature regimes on total cycle of *Pectinophora gossypiella***.

| **SOV** | ***df*** | **SS** | **MS** | **F** | **P** |
| --- | --- | --- | --- | --- | --- |
| Temperatures | 3 | 6531.00 | 2177.00 | 49.2 | 0.00 |
| Error | 8 | 354.00 | 44.25 |  |  |
| Total | 11 | 6885.00 |  |  |  |

Grand Mean 26.500 CV 5.10

**Figure: 4.21: Means total life cycle of *Pectinophora gossypiella*reared on different temperature regimes.**

**Effect of different temperature regimes on fecundity of *Pectinophora gossypiella* female.**

ANOVA table (4.23) showed that all the tested temperature (treatments) had statistically significant effect at P>0.05 on fecundity of female of *Pectinophora gossypiella*.

Results of current investigation exhibited that maximum fecundity of female (152.67eggs) was recorded at 40°C which was statistically different total life cycle recorded at30°C (141.67 egg. The female days recorded at 10°C and 10°C was 0.00 eggs. The obtained result showed that the female fecundity rate was effected with change in the temperature (Fig. 4.22).

**Table: 4.23: ANOVA Parameters regarding effect of different temperature regimes on fecundity of *Pectinophora gossypiella*female.**

| **SOV** | ***df*** | **SS** | **MS** | **F** | **P** |
| --- | --- | --- | --- | --- | --- |
| Treatment | 3 | 65155.6 | 21718.5 | 5923 | 0.00 |
| Error | 8 | 29.3 | 3.7 |  |  |
| Total | 11 | 65184.9 |  |  |  |

Grand Mean 73.583 CV 2.60

**Figure: 4.22: Means fecundity of female *Pectinophora gossypiella*reared on different temperature regimes.**

**CONCLUSION**

Pink bollworm is key pest among all pests which attack the cotton crop and it is distributed all over the world where cotton has grown. This pest shows diapausing behavior in winter months. Diapause in last instar of pink bollworm is induced by various factor including photoperiod, temperature, lipids and moisture etc. For successful and sustainable rearing of pink bollworm in laboratory for carrying out various biological experiments, there is need to investigate the extent of these factors so that these can be manipulated. Current study was conducted to assess the influence of different larval diets having different levels of diapause inducing dietary variant (proteins, carbohydrate, fats and vitamins) as well as different temperature regimes on the diapausing behaviour of *Pectinophora gossypiella.* The study consisted of two experiments. In first experiment eight larval diets with different level of diapause inducing variants were evaluated. 1^st^ instar larvae were fed on artificial modified diets till the larvae reached the last larval instar. The number of diapausing and non-diapausing larvae were counted and transformed into percent diapausing larvae. Similarly, in second experiment counted number of last instar larvae fed on normal diet was kept under different temperatures (10, 20, 30, 40°C) and number of diapausing and non-diapausing larvae was counted and transformed into percent diapausing larvae. Both experiments were carried out in CRD and were repeated thrice. Results of 1^st^ experiment showed that maximum number of diapausing larvae (15.01 larvae) and non-diapausing larvae were recorded on diet-1 and diet-9 (20.01 larvae). The maximum survival rate of diapausing larvae to pupae and pupae to adult was recorded on diet-8 (59.98%), and diet-3 (79.98%), respectively. Whereas, maximum survival rate of non-diapausing larvae to pupae and pupae to adult were exhibited on diet-1 (66.65%) and diet-2 (83.69%), respectively. The larval and pupal weights of *Pectinophora gossypiella* were maximum on diet-6 (23.01 mg) and diet-5 (21.15 mg), respectively. The life span of *Pectinophora gossypiella* male was maximum observed on diet-2 (10.01 days). The life span of *Pectinophora gossypiella* female was maximum observed on diet-2 (10.01 days). The maximum fecundity of *Pectinophora gossypiella* female was maximum recoded on diet-1(150.01 eggs/female). While maximum total life span of *Pectinophora gossypiella* was recorded on diet-4 (47.01 days). The results of second experiment indicated that the maximum diapausing (24.01 larvae) was recorded at 10°C. The maximum non- diapausing larvae was recorded (24.01 larvae) at 10 °C and 30°C. The maximum survival rate of non-diapausing larvae to pupae (100.00%) at 30°C and 40°C. Whereas, the survival rate of non-diapausing pupae to adult exhibited (100.00%) at 20°C. The larval and pupal weights of *Pectinophora gossypiella* were maximum (21.00 mg) and (19.33 mg) at 40°C, respectively. The life span of *Pectinophora gossypiella* male was maximum observed 30°C (8.33 days). The life span of *Pectinophora gossypiella* female was maximum observed 40°C (9.67 days). While maximum total life span of *Pectinophora gossypiella* was recorded on 40°C (51.67days). The maximum fecundity of *Pectinophora gossypiella* was recorded at 40°C (152.67 eggs per female). This study could be helpful for the future study of biological parameters of pink bollworm for mass rearing and could be used in the field for the management of pink bollworm in the cotton crop.

**ACTIVITY-2: STUDY DIFFERENT LIFE STAGES OF THE PBW UNDER NATURAL CONDITIONS BY PERFORMING CAGED STUDIES**

**MATERIALS ANDMETHOD**

**Flower infestation and boll damage**

Total flowers and mature green bolls were counted from 25 plants and then counted the infested flowers and damagedbolls.

**Pink bollworm damage and larval survival**

Infestation (%) of Pink bollworm larvae in field and survival of larvae in flowers and in mature and


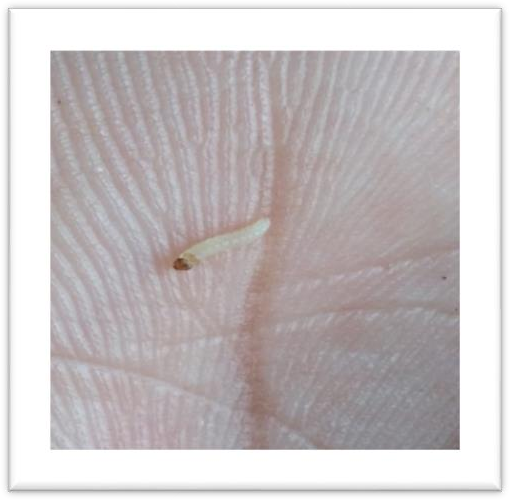

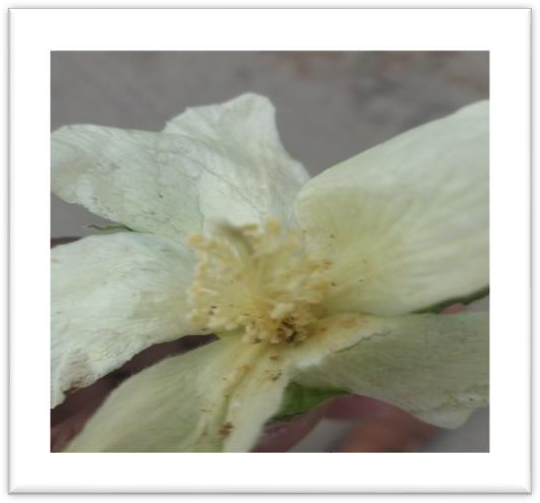

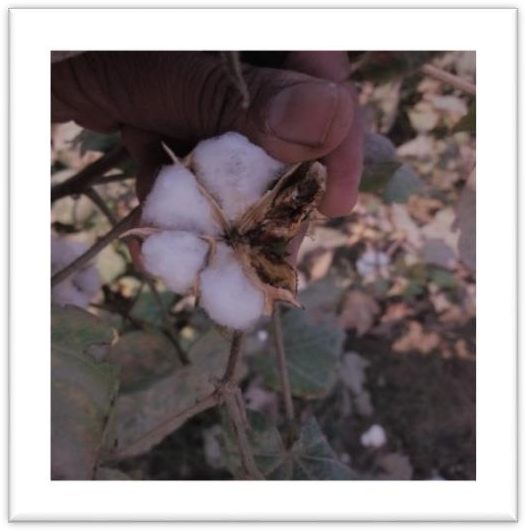
green bolls were recorded

Flower infestation by pink (2^nd^ instar) Boll damaged (4^th^ instar)

bollworm larvae in the field

**Instar/larval stage andlarvae/boll**


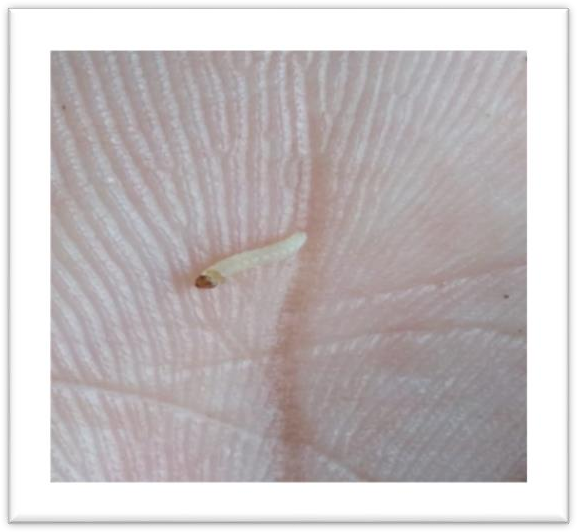

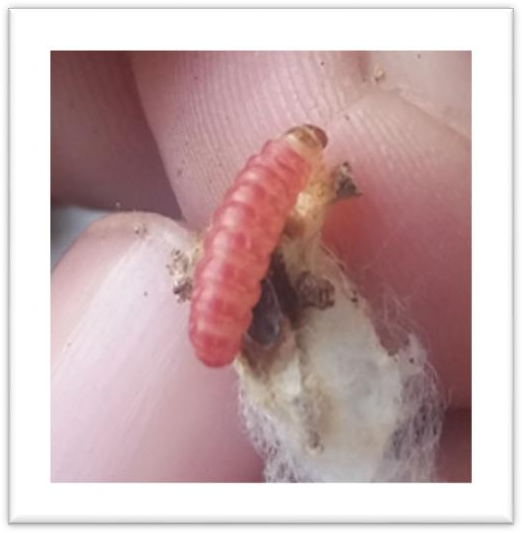
In bolls and flowers PBW larvae were collected and cheeked the instar or larval stage present in bolls or in the flowers and no. of larvae present in boll.

**1st instar larva of pink bollworm 4th instar of Pink bollworm**

**Diapausing Behaviour of Pink Bollworm larvae during on season**

During “on season”, when cotton crop was in the field, from damage bolls, diapausing larvae were counted. This is the diapausing behavior of PBW, either larvae diapause in single seed or double seed.

***Percent damage bolls***

During the on season, the left-over matured bolls were brought in the IPM Laboratory for observation of larvae and percent damage.

***Off season diapausing behavior of Pink Bollworm larvae***

During off season, mature larvae diapause in leftover bolls. Leftover bolls were collectedfromcottonstickstoevaluatethediapausingbehaviorofPinkbollwormlarvaeeither larvae diapauses in single seed or double seed. Survey of ginning factories was conducted to check the diapausing behavior of pink bollworm larvae.


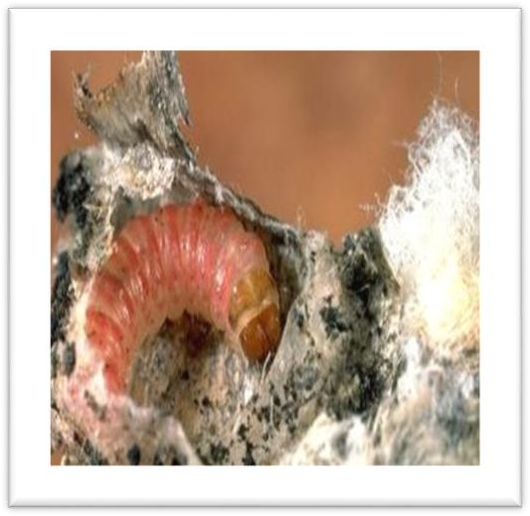

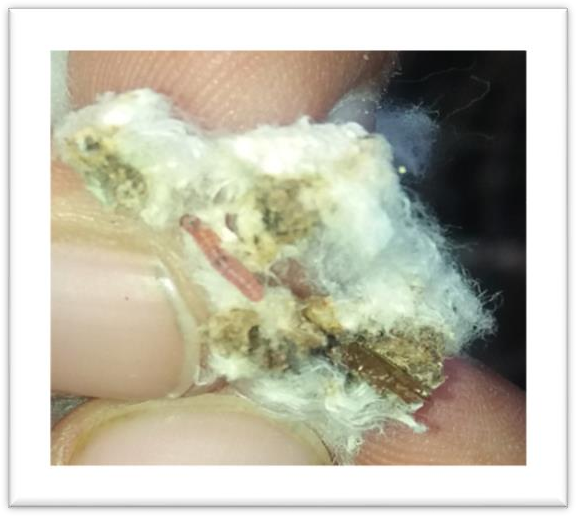
Diapausing behavior of Pink bollworm in seeds during off season (single and double seed diapause)

**Monitoring of adult emergence of Pink bollworm during offseason**


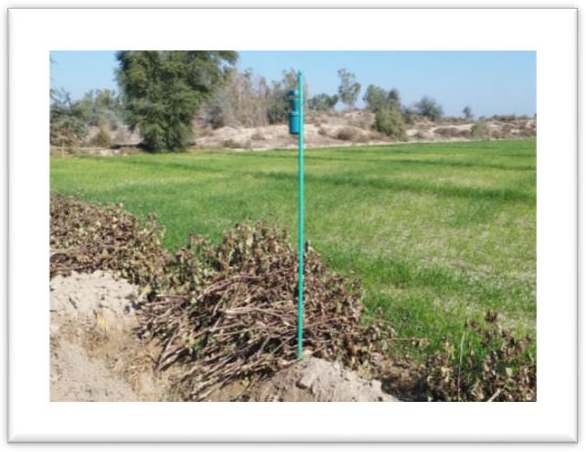

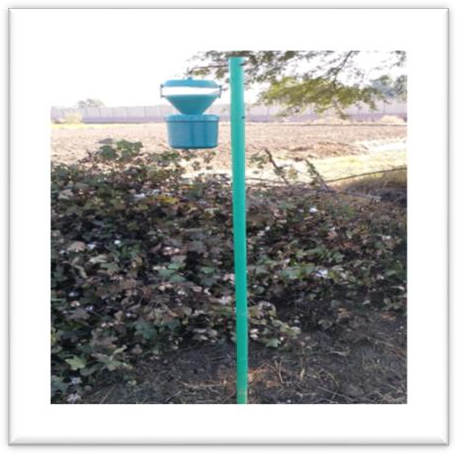
Pheromone traps were installed during the month of December-March to check the adult emergence of Pink bollworm; fortnightly observations were recorded.

Off season monitoring of Pink bollworm at different heaps

**Data collection and Sampling**

Sampling method was Mario-method, total samples were consisting of 25 plants i.e. 5 plants from 5 sampling units, whole and damaged bolls were counted.

**Statistical analysis**

The collected data was analyzed statistically by using suitable statistical software’s.

**RESULTS**

**Flower infestation by Pink bollworm**

Pink Bollworm infestation on flowers recorded from Aug-Nov 2018, and in Table 4.2.1: and Table 4.2.3: the analysis of variance showed significant results and presence of flowers in cotton crop at Layyah and Muzaffargarh, on the other hand infestation level was high on flowers and it showed little bit effect of pheromones and disrupts the adult population atLayyahbutatMuzaffargarhitshowssignificantaffects. In Table 4.2.2: and Table 4.2.4: the analysis of variance showed not highly significant results.

In Table 4.2.5: and Table 4.2.6: the mean Larvae notable of Pink Bollworm infestation on flowers which tells that in Aug and Sep. due to presence of more flowers infestation rate (4.32% and 6%/plant) is high and that time mostly attack was recorded on flowers in district Layyah and Muzaffargarh.

These results are similar to Manjunatha *et al.* (2009) who resulted that by applying early traps of pheromones flower infestation was low in Bt. Cotton during the cotton spell. Our findings also relate with Nadaf and Gloud (2007) who observed lower % damage in cotton by application of lures with insectivores, and crop remains healthy. Accordance to Khuhro *et al.* (2015) by monitoring of PBW and forecasting we can have reduced flower infestation which is depends upon the male moth population.

**Table 4.2.1 ANOVA parameters regarding the total flowers of cotton in Layyah during on season (Aug-Nov, 2018)**

| **SOV** | ***df*** | **SS** | **MS** | **F** | **P** |
| --- | --- | --- | --- | --- | --- |
| Replication | 1 | 0.302 | 0.302 |  |  |
| Treatment | 3 | 304.094 | 101.365 | 3.93 | 0.0145*** |
| Error | 3 | 77.301 | 25.767 |  |  |
| Total | 7 | 381.69 |  |  |  |
| Grand Mean 6.6430 | CV 76.41 |  |  |  |  |

Significance level (P< 0.05); ***Highly significant

**Table 4.2.2: ANOVA parameters regarding the PBW infested flowers of cotton in Layyah during on season (Aug-Nov, 2018)**

| **SOV** | ***df*** | **SS** | **MS** | **F P** |
| --- | --- | --- | --- | --- |
| Replication | 1 | 0.01445 | 0.01445 |  |
| Treatment | 3 | 0.57655 | 0.19218 | 2.30 0.045* |
| Error | 3 | 0.25055 | 0.08352 |  |
| Total | 7 | 0.84155 |  |  |
| Grand Mean 0.3675 | CV 78.64 |  |  |  |

Significance level (P< 0.05) *Significant

**Table 4.2.3: ANOVA parameters regarding the total flowers of cotton in Muzaffargarh during on season (Aug-Nov, 2018)**

| **SOV** | ***df*** | **SS** | **MS** | **F** | **P** |
| --- | --- | --- | --- | --- | --- |
| Replication | 1 | 0.365 | 0.365 |  |  |
| Treatment | 3 | 466.997 | 155.666 | 12.37 | 0.033* |
| Error | 3 | 37.764 | 12.588 |  |  |
| Total | 7 |  |  |  |  |
| Grand Mean 8.5860 | CV 41.32 |  |  |  |  |

Significance level (P< 0.05)

*Significant

**Table No. 4.2.4: ANOVA parameters regarding the infested flowers/plant of cotton by Pink bollworm at Muzaffargarh during on season (Aug-Nov, 2018)**

| **SOV** | ***df*** | **SS** | **MS** | **F** | **P** |
| --- | --- | --- | --- | --- | --- |
| Replication | 1 | 0.01445 | 0.01445 |  |  |
| Treatment | 3 | 2.15815 | 0.71938 | 28.4 | 0.010** |
| Error | 3 | 0.07575 | 0.02525 |  |  |
| Total | 7 | 2.24835 |  |  |  |
| Grand Mean 0.6225 | CV 25.53 |  |  |  |  |

Significance level (P< 0.05) **Highly Significant

**Table 4.2.5: Means (±SE) of total flowers/plant, infested flowers/Plant and percent infested flowers/plant in cotton at Layyah during on season (Aug-Nov, 2018)**

| **Flower Infestation/ On Season** | |  | **Layyah** |  |
| --- | --- | --- | --- | --- |
| **Months** | **Days** | **Total Flowers/ Plant** | **Infested Flowers**  **/ Plant** | **Percent infested flower/plant**% |
|  | 1-15 Aug. | 11.12^B^±2.1 | 0.16^C^±0.2 | 0.64^C^±0.2 |
| August | 16-31 Aug. | 18.72^A^±3 | 0.4^B^±0.2 | 1.6^B^±1.1 |
|  | 1-15 Sep. | 15.34^A^±3.4 | 1.08^A^±0.3 | 4.32^A^±1.4 |
| September | 16-30 Sep. | 5.6^BC^±1.3 | 0.4^B^±0.1 | 1.6^B^±1.1 |
|  | 1-15 Oct. | 1.6^C^±1.1 | 0.4^B^±0.19 | 1.6^B^±1.1 |
| October | 16-31 Oct. | 1.4^C^±0.69 | 0.5^B^±0.1 | 2^B^±1.3 |
|  | 1-15 Nov. | 0.12^CD^±0.2 | 0^D^±0 | 0^D^±0 |
| November | 16-31 Nov. | 0^D^±0 | 0^D^±0 | 0^D^±0 |

Means bearing identically formatted same letters are not different from each other’s at 5% probability level.

**Table 4.2.6: Means (±SE) of total flowers/plant, infested flowers/plant and percent infested flowers/plant in cotton at Muzaffargarh during on season (Aug- Nov, 2018)**

| **Flower infestation/ On Season** | |  | **Muzaffargarh** |  |
| --- | --- | --- | --- | --- |
| **Months** | **Days** | **Total Flowers/ Plant** | **Infested Flowers / Plant** | **Percent Infestation**% |
|  | 1-15 Aug. | 18.48^A^±2.1 | 0.24^C^±0.1 | 0.96^B^±0.4 |
| August | 16-31 Aug. | 22.52^A^±3.3 | 0.26^C^±0.11 | 1.04^B^±0.8 |
|  | 1-15 Sep. | 14.16^B^±1.9 | 1.5^A^±0.16 | 6^A^±1.5 |
|  | 16-30 Sep. | 8.08^BC^±1,4 | 1.08^A^±0.1 | 4^A^±1.3 |
|  | 1-15 Oct. | 3.8^C^±1,1 | 0.92^B^±0.1 | 3.68^AB^±1.8 |
| October | 16-31 Oct. | 4.68^C^±1,3 | 0.98^B^±0.12 | 3.92^AB^±1.9 |
|  | 1-15 Nov. | 0.16^D^±0,3 | 0^D^±0 | 0^C^±0 |
| November | 16-31 Nov. | 0^D^±0 | 0^D^±0 | 0^C^±0 |

Means bearing identically formatted same letters are not different from each other’s at 5% probability level.

**Boll infestation**

Pink Bollworm infestation on bolls recorded from Aug-Nov, 2018, and in Table 4.2.7: and Table 4.2.9: the analysis of variance shows significant results and presence of flowers in cotton crop at Layyah and Muzaffargarh, on the other hand infestation level was high on flowers and it showed little bit effect of pheromones and disrupts the adult population at Layyah, it shows significant affects in Table 4.2.8: and Table 4.2.10: the analysis of variance showed non-significant results.

In Table 4.2.11: and Table 4.2.12: the mean orave notable of Pink Bollworm infestation on bolls which tells that in Oct. (4.15% and 3.7%/plant) and Nov. (3.7% and 8.8% /plant) due to presence of more bolls, infestation rate is high and that time mostly attack was recorded on bolls in district Layyah and Muzaffargarh.

Hardee *et al.* (2001) reported that on B.t cotton with application of pheromones boll damage was low and Douglas *et al.* (1992) also concluded early season application of pheromones in cotton field were efficient to manage the boll damage and reduction in boll damagewasupto93%andbythislarvalincidencewasalsolowandnooflarvaeinbollswas low. Arif *et al.* (2006) use of B.t cotton can helpful in reduction of boll damage and in B.t cottonlarvaldensitywaslowduringtheseasonbecauseB.thaveresistanceagainstbollworms. And Pemsl *et al.* (2005) concluded that B.t toxin can protect pink bollworm damage and application ofpheromones.

**Table 4.2.7: ANOVA parameters regarding the total bolls/ in cotton at Layyah during on season (Aug-Nov, 2018)**

| **SOV** | ***df*** | **SS** | **MS** | **F** | **P** |
| --- | --- | --- | --- | --- | --- |
| Replication | 1 | 108.81 | 108.811 |  |  |
| Treatment | 3 | 1816.46 | 605.487 | 22.83 | 0.01** |
| Error | 3 | 79.57 | 26.523 |  |  |
| Total | 7 |  |  |  |  |
| Grand Mean 36.450 | CV 14.13 |  |  |  |  |

Significance level (P< 0.05)

**Highly Significant

**Table 4.2.8. ANOVA parameters regarding the infested bolls or boll damage/plant by Pink bollworm in cotton at Layyah during on season (Aug-Nov, 2018)**

| **SOV** | ***df*** | **SS** | **MS** | **F** | **P** |
| --- | --- | --- | --- | --- | --- |
| Replication | 1 | 0.47531 | 0.47531 |  |  |
| Treatment | 3 | 8.42104 | 2.80701 | 11.34 | 0.038* |
| Error | 3 | 0.74254 | 0.24751 |  |  |
| Total | 7 | 9.63889 |  |  |  |
| Grand Mean 1.7813 | CV 27.93 |  |  |  |  |

Significance level (P< 0.05); **Significant

**Table 4.2.9: ANOVA parameters regarding the total bolls/ in cotton at Muzaffargarh during on season (Aug-Nov, 2018)**

| **SOV** | ***df*** | **SS** | **MS** | **F** | **P** |
| --- | --- | --- | --- | --- | --- |
| Replication | 1 | 54.34 | 54.340 |  |  |
| Treatment | 3 | 1751.50 | 583.833 | 12.12 | 0.034* |
| Error | 3 | 144.53 | 48.177 |  |  |
| Total | 7 | 1950.37 |  |  |  |
| Grand Mean 37.884 | CV 18.32 |  |  |  |  |

Significance level (P< 0.05); *Significant

**Table 4.2.10: ANOVA parameters regarding infested bolls/plant by PBW at Muzaffargarh during on season (Aug-Nov, 2018)**

| **SOV** | ***df*** | **SS** | **MS** | **F** | **P** |
| --- | --- | --- | --- | --- | --- |
| Replication | 1 | 0.52020 | 0.52020 |  |  |
| Treatment | 3 | 4.07480 | 1.35827 | 4.99 | 0.109* |
| Error | 3 | 0.81620 | 0.27207 |  |  |
| Total | 7 | 5.41120 |  |  |  |
| Grand Mean 1.4300 | CV 36.48 |  |  |  |  |

Significance level (P< 0.05) NS=Non-Significant

**Table 4.2.11: Means (±SE) of total bolls/plant, infested bolls/plant and percent infested bolls/plant in cotton at Muzaffargarh during on season (Aug-Nov, 2018)**

| **Boll Infestation/On season** | |  | **Layyah** |  |
| --- | --- | --- | --- | --- |
| **Months** | **Days** | **Total Bolls/ Plant** | **Infested Bolls/ Plant** | **Percent Infested Bolls**% |
|  | 1-15 Aug. | 15.1^D^±3.1 | 0.2^D^±0.3 | 1.32^D^±0.6 |
| August | 16-31 Aug. | 20.68^C^±4.1 | 0.3^CD^±0.4 | 1.55^D^±0.4 |
|  | 1-15 Sep. | 35.76^BC^±4.7 | 1.7^C^±0.2 | 4.75^B^±1.4 |
| September | 16-30 Sep. | 47.12^B^±5.1 | 1.8^BC^±0.3 | 3.82^C^±1.1 |
|  | 1-15 Oct. | 51.24^A^±5.3 | 1.89^BC^±0.5 | 3.7^C^±1.1 |
| October | 16-31 Oct. | 50.12^A^±5.1 | 2.1^B^±0.4 | 4.15^BC^±1.4 |
|  | 1-15 Nov. | 49.01^AB^±4.9 | 2.36^B^±0.5 | 4.72^B^±1.5 |
| November | 16-31 Nov. | 44.04^B^±4.6 | 3.9^A^±0.6 | 8.8^A^±0.6 |

Means bearing identically formatted same letters are not different from each other’s at 5% probability level

**Table 4.2.12: Means (±SE) of total bolls/plant, infested bolls/plant and percent infested bolls/plant in cotton at Muzaffargarh during on season (Aug-Nov, 2018)**

| **Boll Infestation/On season** | |  | **Muzaffargarh** |  |
| --- | --- | --- | --- | --- |
| **Months** | **Days** | **Total Bolls/ Plant** | **Infested Bolls/ Plant** | **Percent Infested Bolls**% |
|  | 1-15 Aug. | 13.56^E^±2.1 | 0.16^E^±0.09 | 1.17^D^±0.5 |
| August | 16-31 Aug. | 21.44^D^±3.1 | 0.32^D^±0.2 | 1.49^C^±0.5 |
|  | 1-15 Sep. | 32.4^CD^±4.2 | 0.68^C^±0.4 | 2.09^BC^±0.9 |
| September | 16-30 Sep. | 34.08^C^±4.1 | 1.32^BC^±0.3 | 3.82^AB^±1.1 |
|  | 1-15 Oct. | 54.32^AB^±5.1 | 1.5^B^±0.4 | 2.8^B^±0.8 |
| October | 16-31 Oct. | 53.76^AB^±4.0 | 2^AB^±0.9 | 3.7^AB^±1.1 |
|  | 1-15 Nov. | 56.28^A^±5.3 | 1.36^BC^±0.5 | 2.3^B^±0.7 |
| November | 16-31 Nov. | 51.52^B^±4.9 | 2.9^A^±0.9 | 5.7^A^±1.5 |

Means bearing identically formatted same letters are not different from each other’s at 5% probability level.

**Larvae/boll**

Pink Bollworm larvae presence in bolls was checked during on season and no. of larvae present in bolls in cotton crop. In Tables 4.2.13: and 4.2.14: analysis of variance shows significant results of presence of larvae in cotton bolls. In Table 4.2.15: means show that more larvae present in Nov. (1.96 and 1.52/boll) in cotton bolls at Layyah and Muzaffargarh.

These findings were similar to Khuhro *et al.* (2015) who found that highest larval% was 3.2%/larvae in November at 3 districts of Sindh, and in October larval population was recorded up to (1.7-1.9%/boll) in Sindh. These results also tell us some worm’s remains alive in bolls and goes to resting stage and remain alive in B.t toxin in late season. Our results also relate with Wan *et al.* (2004) and with Zhang *et al.* (2001) who also observed most larvae was presents in bolls during Oct-Nov.

**Table 4.2.13: ANOVA parameters regarding larvae/boll in cotton crop at Layyah, during on season during on season (Aug-Nov, 2018)**

| **Source** | ***df*** | **SS** | **MS** | **F** | **P** |
| --- | --- | --- | --- | --- | --- |
| Replication | 1 | 0.01531 | 0.01531 |  |  |
| Treatment | 3 | 2.79874 | 0.93291 | 265.60 | 0.0004*** |
| Error | 3 | 0.01054 | 0.00351 |  |  |
| Total | 7 | 2.82459 |  |  |  |
| Grand Mean 1.1538 | CV 5.14 |  |  |  |  |

Significance level (P< 0.05); ***Highly significant

**Table 4.2.14: ANOVA parameters regarding larvae/boll in cotton crop at Muzaffargarh during on season (Aug-Nov, 2018)**

| **Source** | ***df*** | **SS** | **MS F P** |
| --- | --- | --- | --- |
| Replication | 1 | 0.01445 | 0.01445 |
| Treatment | 3 | 1.51175 | 0.50392 32.48 0.0087*** |
| Error | 3 | 0.04655 | 0.01552 |
| Total | 7 | 1.57275 |  |
| Grand Mean 0.9525 | CV 13.08 |  |  |

Significance level (P< 0.05); ***Highly Significant

**Table 4.2.15: Mean (±SE) regarding PBW larvae/boll in cotton crop at Layyah and Muzaffargarh during on season during on season (Aug-Nov, 2018)**

| **On season** | | **PBW Larvae / Boll** | |
| --- | --- | --- | --- |
| **Months** | **Days** | **Layyah** | **Muzaffargarh** |
|  | 1-15 Aug. | 0.2^DE^±0.04 | 0.16^D^±0.06 |
| August | 16-31 Aug. | 0.24^D^±0.05 | 0.28^C^±0.01 |
|  | 1-15 Sep. | 1.18^C^±0.04 | 1.12^BC^±0.02 |
| September | 16-30 Sep. | 1.28^BC^±0.04 | 1.08^BC^±0.08 |
|  | 1-15 Oct. | 1.3^B^±0.03 | 1.16^B^±0.1 |
| October | 16-31 Oct. | 1.31^B^±0.05 | 1.1^B^±0.02 |
|  | 1-15 Nov. | 1.76^AB^±0.03 | 1.2^AB^±0.05 |
| November | 16-31 Nov. | 1.96^A^±0.05 | 1.52^A^±0.06 |

Means bearing identically formatted same letters are not different from each other’s at 5% probability level.

**Instar/larval stage**

Pink Bollworm larval stage or instar present in cotton bolls during on season in cotton crop. Analysis of variance regarding 1^st^ instar/boll showed non-significant results (Table 4.2.16). However, analysis of variance regarding 2^nd^, 3^rd^, and 4th instar/boll showed significant results (Table 4.2.17, 4.2.18 & 4.2.19). 1^st^ and 3^rd^ instar was not present in bolls or in flowers. No. of 1^st^ and 3^rd^ instar was low as compared to 2^nd^ and 4^th^ instar, (Table 4.2.20) at Layyah. If we look on analysis regarding Muzaffargarh, all larval stages shows non-significant results about presence of larval stages in flowers and bolls (Table 4.2.21, 4.2.22, 4.2.23 & 4.2.24).

Tables 4.2.20 and 4.2.25 show mean values and ave. no.of larval stages present in flowers and bolls in Layyah and Muzaffargarh. Both tables tell that 1^st^ and 2^nd^ instar present in flowers during Aug and Sep and 3^rd^ and 4^th^ instar present in mature bolls in cotton crop at Layyah and Muzaffargarh. Presented data similar Shah *et al.* (2013) to show the measurements of pink bollworm, *P. gossypiella* head capsules width of different larval instars collected from infested cotton bolls. The mean average of head capsule width recorded (0.17, 0.38, 0.77 and 1.75) mm. at the first, second, third and fourth instar, respectively Kreema *et al* 2015. The current investigation results agree with those finding of Yones, *et al.* (2011).

**Table 4.2.16: ANOVA parameters regarding 1^st^ instar or larval stage/boll in cotton crop at Layyah during on season (Aug-Nov, 2018)**

| **SOV** | ***df*** | **SS** | **MS** | **F** | **P** |
| --- | --- | --- | --- | --- | --- |
| Replication | 1 | 0.12500 | 0.12500 |  |  |
| Treatment | 3 | 1.37500 | 0.45833 | 3.67 | 0.157^NS^ |
| Error | 3 | 0.37500 | 0.12500 |  |  |
| Total | 7 | 1.87500 |  |  |  |
| Grand Mean 0.6250 | CV 56.57 |  |  |  |  |
| Significance level (P< | 0.05) |  |  |  | NS=Non-Significant |

**Table 4.2.17: ANOVA parameters regarding 2nd instar/boll in cotton crop at Layyah during on season (Aug-Nov, 2018)**

| **SOV** | ***df*** | **SS** | **MS** | **F** | **P** |
| --- | --- | --- | --- | --- | --- |
| Replication | 1 | 0.02000 | 0.02000 |  |  |
| Treatment | 3 | 1.54000 | 0.51333 | 77.00 | 0.002*** |
| Error | 3 | 0.02000 | 0.00667 |  |  |
| Total | 7 | 1.58000 |  |  |  |
| Grand Mean 0.7500 | CV 10.89 |  |  |  |  |

Significance level (P< 0.05); ***Highly Significant

**Table 4.2.18: ANOVA parameters regarding 3^rd^ instar/boll in cotton crop at Layyah during on season (Aug-Nov, 2018)**

| **SOV** | ***df*** | **SS** | **MS** | **F** | **P** |
| --- | --- | --- | --- | --- | --- |
| Replication | 1 | 0.10811 | 0.10811 |  |  |
| Treatment | 3 | 0.25204 | 0.08401 | 2.03 | 0.288* |
| Error | 3 | 0.12434 | 0.04145 |  |  |
| Total | 7 | 0.48449 |  |  |  |
| Grand Mean 1.0013 | CV 20.33 |  |  |  |  |

Significance level (P< 0.05) NS=Non-significant

**Table 4.2.19: ANOVA parameters regarding 4^th^ instar/boll in cotton crop at Layyah during on season (Aug-Nov, 2018)**

| **SOV** | ***df*** | **SS** | **MS** | **F** | **P** |
| --- | --- | --- | --- | --- | --- |
| Replication | 1 | 0.00151 | 0.00151 |  |  |
| Treatment | 3 | 2.95154 | 0.98385 | 834.36 | 0.000*** |
| Error | 3 | 0.00354 | 0.00118 |  |  |
| Total | 7 | 2.95659 |  |  |  |
| Grand Mean 0.9863 | CV 3.48 |  |  |  |  |

Significance level (P< 0.05); ***Highly Significant

**Table 4.2.20: Mean (±SE) regarding instar/larval stage of Pink bollworm cotton bolls in Layyah during on season during on season (Aug-Nov, 2018)**

| **Instar, Larval Stage/ on season** | |  | **Instar** | |  |
| --- | --- | --- | --- | --- | --- |
| **Layyah** | | | | | |
| **Months** | **Days** | **1st** | **2nd** | **3^rd^** | **4th** |
|  | 1-15 Aug. | 1^A^±0.2 | 0.8^BC^±0.05 | 0.4^C^±0.1 | 0^D^±0 |
| August | 16-31 Aug. | 1^A^±0.3 | 1^AB^±0.3 | 1^B^±0.2 | 0^D^±0 |
|  | 1-15 Sep. | 1^A^±0.2 | 1^AB^±0.3 | 1^B^±0.2 | 1^C^±0.03 |
| September | 16-30 Sep. | 1^A^±0.2 | 1.2^A^±0.8 | 1.3^A^±0.1 | 1^C^±0.03 |
|  | 1-15 Oct. | 1^A^±0.2 | 1^AB^±0.2 | 1^AB^±0.3 | 1.4^B^±0.02 |
| October | 16-31 Oct. | 0^B^±0.2 | 1^AB^±0.2 | 1.1^AB^±0.1 | 1.3^B^±0.04 |
|  | 1-15 Nov. | 0^B^±0 | 0^C^±0 | 1.14^AB^±0.1 | 1.6^AB^±0.06 |
| November | 16-31 Nov. | 0^B^±0 | 0^C^±0 | 1.07^AB^±0.2 | 1.59^A^±0.08 |

Means bearing identically formatted same letters are not different from each other at 5% probability level.

| **Table 4.2.21: ANOVA parameters regarding 1st instar/boll of PBW at Muzaffargarh during cotton season (Aug-Nov, in 2018)** | | | | | | |
| --- | --- | --- | --- | --- | --- | --- |
| **SOV** | ***df*** | **SS** |  | **MS** | **F** | **P** |
| Replication | 1 | 0.00000 |  | 0.00000 |  |  |
| Treatment | 3 | 1.50000 |  | 0.50000 | M | M |
| Error | 3 | 0.00000 |  | 0.00000 |  |  |
| Total | 7 | 1.50000 |  |  |  |  |
| Grand Mean 0.7500 |  |  |  |  |  |  |

Significance level (P< 0.05); ^NS^Non-significant

**Table 4.2.22: ANOVA parameters regarding 2^nd^ instar/boll of PBW at Muzaffargarh during cotton season (Aug-Nov, 2018)**

| **SOV** | ***df*** | **SS** | **MS** | **F** | **P** |
| --- | --- | --- | --- | --- | --- |
| Replication | 1 | 0.00500 | 0.00500 |  |  |
| Treatment | 3 | 0.01500 | 0.00500 | 1.00 | 0.50^NS^ |
| Error | 3 | 0.01500 | 0.00500 |  |  |
| Total | 7 | 0.03500 |  |  |  |
| Grand Mean 0.9750 | CV 7.25 |  |  |  |  |

Significance level (P< 0.05); ^NS^Non-significant

**Table 4.2.23: ANOVA parameters regarding 3^rd^ instar/boll of PBW at Muzaffargarh during on season (Aug-Nov, 2018)**

| **SOV** | ***df*** | **SS** | **MS** | **F** | **P** |
| --- | --- | --- | --- | --- | --- |
| Replication | 1 | 0.02000 | 0.02000 |  |  |
| Treatment | 3 | 0.06000 | 0.02000 | 1.00 | 0.50^NS^ |
| Error | 3 | 0.06000 | 0.02000 |  |  |
| Total | 7 | 0.14000 |  |  |  |
| Grand Mean 0.9500 | CV 14.89 |  |  |  |  |

Significance level (P< 0.05); NS=Non-significant

**Table 4.2.24: ANOVA parameters regarding 4th instar/boll of PBW at Muzaffargarh during on season (Aug-Nov, 2018)**

| **SOV** | ***df*** | **SS** | **MS** | **F** | **P** |
| --- | --- | --- | --- | --- | --- |
| Replication | 1 | 0.20480 | 0.20480 |  |  |
| Treatment | 3 | 0.47440 | 0.15813 | 1.42 | 0.39^NS^ |
| Error | 3 | 0.33440 | 0.11147 |  |  |
| Total | 7 | 1.01360 |  |  |  |
| Grand Mean 0.9100 | CV 36.69 |  |  |  |  |

Significance level (P< 0.05) NS=Non-significant

**Table 4.2.25: Mean (±SE) no table regarding instar/larval stage of Pink Bollworm in cotton bolls in Layyah during on season (Aug-Nov, 2018)**

| **Instar, Larval Stage/ on season** | |  | **Instar** | |  |
| --- | --- | --- | --- | --- | --- |
| **Muzaffargarh** | | | | | |
| **Months** | **Days** | **1^st^** | **2nd** | **3rd** | **4th** |
|  | 1-15 Aug. | 1^A^±0.02 | 0.8^AB^±0.05 | 0.6^B^±0.1 | 0^C^±0.02 |
| August | 16-31 Aug. | 1^A^±0.02 | 1^A^±0.02 | 1^A^±0.02 | 1^B^±0.02 |
|  | 1-15 Sep. | 1^A^±0.02 | 1^A^±0.02 | 1^A^±0.03 | 1^B^±0.3 |
| September | 16-30 Sep. | 1^A^±0.02 | 1^A^±0.02 | 1^A^±0.03 | 1^B^±0.02 |
|  | 1-15 Oct. | 1^A^±0.02 | 1^A^±0.02 | 1^A^±0.02 | 1^B^±0.03 |
| October | 16-31 Oct. | 1^A^±0.02 | 1^A^±0.02 | 1^A^±0.03 | 1^B^±0.03 |
|  | 1-15 Nov. | 0^B^±0 | 1^A^±0.02 | 1^A^±0.03 | 1^B^±0.03 |
| November | 16-31 Nov. | 0^B^±0 | 1^A^±0.02 | 1^A^±0.02 | 1.28^A^±0.03 |

Means bearing identically formatted same letters are not different from each other’s at 5% probability level.

**Diapausing behavior of Pink bollworm**

Diapause of PBW is part of its life cycle and in winter season 4^th^ instar goes to diapause or in resting stage when condition will be favorable, these larvae emerge as adult’s moths and start laying eggs on cotton crop. Table 4.2.25 and 4.2.26 depict the analysis of variance that showed significant results and Fig 4.2.1 shows that number of NDSDL were more as compared to NSSDL at Layyah. On the other hand, in Tables 4.2.27 and 4.2.28 at Muzaffargarh diapausing behavior of PBW same as in Layyah. Fig.4.2.2 also showed that NDSDLwere more than NSSDL at Muzaffargarh. Chaudhari *et al.* (2006) revealed that different larvae initiate to get ready for offseason diapause in end days of August and this diapausing stage hastens very swift after mid-September as lengthsof the days start to shorten. By late autumn, if bolls are present, it is not odd to have different larvae on one boll might be seen. These most recent bolls on the dorsal part of the plant are normally immature, have poor quality of lint and in production no participation of this lint.

**Table 4.2.25: ANOVA parameters regarding NSSDL (No. of single seed diapausing larvae) /plant of PBW in cotton at Layyah during on season (Aug-Nov, 2018)**

| **SOV** | ***df*** | **SS** | **MS** | **F** | **P** |
| --- | --- | --- | --- | --- | --- |
| Replication | 1 | 0.00405 | 0.00405 |  |  |
| Treatment | 3 | 0.03775 | 0.01258 | 21.57 | 0.015*** |
| Error | 3 | 0.00175 | 0.00058 |  |  |
| Total | 7 | 0.04355 |  |  |  |
| Grand Mean 0.0775 | CV 31.16 |  |  |  |  |

Significance level (P< 0.05); ***Highly significant

**Table 4.2.26: ANOVA parameters regarding NDSDL (No. of double seed diapausing larvae)/plant of PBW in cotton crop at Layyah during on season (Aug-Nov, 2018)**

| **SOV** | ***df*** | **SS** | **MS** | **F** | **P** |
| --- | --- | --- | --- | --- | --- |
| Replication | 1 | 0.00911 | 0.00911 |  |  |
| Treatment | 3 | 0.24464 | 0.08155 | 26.20 | 0.011*** |
| Error | 3 | 0.00934 | 0.00311 |  |  |
| Total | 7 | 0.26309 |  |  |  |
| Grand Mean 0.1913 | CV 29.17 |  |  |  |  |

Significance level (<P= 0.05); ***Highly significant

**Table 4.2.27: ANOVA parameters regarding NSSDL (No. of single seed diapausing larvae)/plant of PBW in cotton at Muzaffargarh during on season (Aug- Nov, 2018)**

| **SOV** | ***df*** | **SS** | **MS** | **F** | **P** |
| --- | --- | --- | --- | --- | --- |
| Replication | 1 | 0.00001 | 0.00001 |  |  |
| Treatment | 3 | 0.09814 | 0.03271 | 153.94 | 0.000*** |
| Error | 3 | 0.00064 | 0.00021 |  |  |
| Total | 7 | 0.09879 |  |  |  |
| Grand Mean 0.0838 | CV 17.41 |  |  |  |  |

Significance level (P< 0.05); ***Highly significant

**Table 4.2.28. ANOVA parameters regarding NDSDL (No. of double seed diapausing larvae)/ Plant of PBW in cotton at Muzaffargarh during on season (Aug- Nov, 2018**

| **SOV** | ***df*** | **SS** | **MS** | **F** | **P** |
| --- | --- | --- | --- | --- | --- |
| Replication | 1 | 0.00500 | 0.00500 |  |  |
| Treatment | 3 | 0.30780 | 0.10260 | 35.79 | 0.007*** |
| Error | 3 | 0.00860 | 0.00287 |  |  |
| Total | 7 | 0.32140 |  |  |  |
| Grand Mean 0.1650 CV 32.45 |  |  |  |  |  |

Significance level (P<0.05); ***Highly significant

**Diapause**


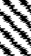

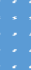

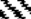

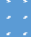

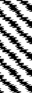

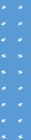

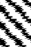

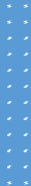

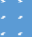

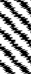

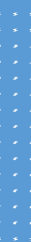

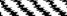

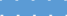


**0.7**

**0.6**

**0.5**

**0.4**

**0.3**

**0.2**

**0.1**

**0**

Diapausing Behaviour

NSSDL

NDSDL

Linear(NDSDL)

A

A

AB

AB

A

B

B

B

AB

BC

AB

**1-15Aug. 16-31 1-15Sep. 16-30**

**Aug. Sep.**

**1-15Oct.**

**Onseason**

**16-31 1-15Nov.**

**Oct.**

**August September October**

**16-31**

**Nov.**

**November**

**Fig. 4.2.1. Diapausing Behavior of Pink bollworm NSSDL and NDSDL (No. of single seed diapausing larvae and No. of double seed diapausing larvae) in Layyah**

**0.7**


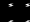

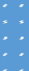

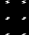


**A**

AB

**B**

**B**

AB

BC

BC

**1-15**

**Aug.**

**16-31 1-15Sep.**

**Aug.**

**August**

**16-30 1-15Oct.**

**Sep.**

**September**

**16-31 1-15Nov. 16-31**

**Oct. Nov.**

**October**

**November**

**0.6**

Max. Daipause

**0.5**

**0.4**

**0.3**

**0.2**

**0.1**

**0**

**Diapausing Behaviour**

**A**

On season

NSSDL NDSDL Linear(NDSDL)

**Fig. 4.2.2: Diapausing Behavior of Pink bollworm NSSDL and NDSDL (No. of single seed diapausing larvae and No. of double seed diapausing larvae) in Muzaffargarh**

**Larvae under plant canopy**

Sometimes during PBW attack larva fall to soil or sometimes green bolls fell down on soil and larvae remain alive and ultimately diapauses in soil. Our results show non-significant results in tables 4.2.29 and 4.2.30 analysis of variance about presence of larvae in soil atLayyah and Muzaffargarh. Mean tables 4.2.31 of both locations show that presence of larvae in soil but in very less numbers.

**Table 4.2.29: ANOVA parameters regarding larvae under plant canopy (LuPC)/plant of PBW in cotton crop at Layyah during on season (Aug-Nov, 2018)**

| **SOV** | ***df*** | **SS** | **MS** | **F** | **P** |
| --- | --- | --- | --- | --- | --- |
| Replication | 1 | 0.04205 | 0.04205 |  |  |
| Treatment | 3 | 0.23170 | 0.07723 | 1.36 | 0.40^NS^ |
| Error | 3 | 0.17045 | 0.05682 |  |  |
| Total | 7 | 0.44420 |  |  |  |
| Grand Mean 0.2100 CV 113.51 |  |  |  |  |  |

Significance level (P< 0.05) NS=Non-significant

**Table 4.2.30: ANOVA parameters regarding larvae under plant canopy (LuPC)/Plant of PBW in cotton crop at Muzaffargarh during on season (Aug-Nov, 2018)**

| **SOV** | ***df*** | **SS** | **MS** | **F P** |
| --- | --- | --- | --- | --- |
| Relocation | 1 | 0.10580 | 0.10580 |  |
| Treatment | 3 | 0.38545 | 0.12848 | 1.330.410^NS^ |
| Error | 3 | 0.29050 | 0.09683 |  |
| Total | 7 | 0.78175 |  |  |
| Grand Mean 0.2325 CV 133.84 |  |  |  |  |

Significance level (P< 0.05) NS=Non-significant

**Table 4.2.31: Mean (±SE) table of larvae under plant canopy/plant of PBW in cotton crop at Layyah and Muzaffargarh during on season (Aug-Nov, 2018)**

| **On Season** | | **Larvae under Plant Canopy** | |
| --- | --- | --- | --- |
| **Months** | **Days** | **Layyah** | **Muzaffargarh** |
|  | 1-15 Aug. | 0^D^±0 | 0^E^±0 |
| August | 16-31 Aug. | 0^D^±0 | 0^E^±0 |
|  | 1-15 Sep. | 0.8^B^±0.1 | 0.08^D^±0.1 |
| September | 16-30 Sep. | 0.15^C^±0.2 | 0.07^D^±0.2 |
|  | 1-15 Oct. | 0.16^C^±0.2 | 1.01^A^±0.1 |
| October | 16-31 Oct. | 0.2^A^±0.3 | 0.12^C^±0.1 |
|  | 1-15 Nov. | 0.17^C^±0.1 | 0.3^B^±0.1 |
| November | 16-31 Nov. | 0.2^A^±0.1 | 0.28^B^±0.1 |

Means bearing identically formatted same letters are not different from each other’s at 5% probability level.

**Damage/100 Bolls**

Bolls was collected from field after every 15 days interval then damage bolls was counted at both location analysis shows significant results about damage% in tables 4.2.32and

4.2.33. In table 4.2.34 mean table show that at both location damage % was high during Oct- Nov. (21/100bolls and 15/100bolls- 26/100bolls and 19/100bolls) at Layyah and Muzaffargarh.

**Table 4.2.32: ANOVA parameters regarding damage (%) in cotton crop at Layyah during on season (Aug-Nov, 2018)**

| **SOV** | ***df*** | **SS** | **MS** | **F** | **P** |
| --- | --- | --- | --- | --- | --- |
| Replication | 1 | 6.125 | 6.125 |  |  |
| Treatment | 3 | 481.375 | 160.458 | 65.27 | 0.003*** |
| Error | 3 | 7.375 | 2.458 |  |  |
| Total | 7 | 494.875 |  |  |  |
| Grand Mean 11.125 | CV 14.09 |  |  |  |  |

Significance level (P< 0.05); ***Highly significant

**Table 4.2.33: ANOVA parameters regarding damage % in cotton crop at Layyah during on season (Aug-Nov, 2018)**

| **SOV** | ***df*** | **SS** | **MS** | **F** | **P** |
| --- | --- | --- | --- | --- | --- |
| Replication | 1 | 10.125 | 10.1250 |  |  |
| Treatment | 3 | 225.375 | 75.1250 | 66.78 | 0.003*** |
| Error | 3 | 3.375 | 1.1250 |  |  |
| Total | 7 | 238.875 |  |  |  |
| Grand Mean 8.8750 | CV 11.95 |  |  |  |  |

Significance level (P< 0.05); ***Highly significant

**Table 4.2.34. Mean (±SE) comparison table of damage% at Layyah and Muzaffargarh during on season (Aug-Nov, 2018)**

| **Damage %/Infestation %** | | **Damage /100 bolls %** | |
| --- | --- | --- | --- |
| **Months** | **Days** | **Layyah** | **Muzaffargarh** |
|  | 1-15 Aug. | 3^E^±1.1 | 2^E^±0.7 |
| August | 16-31 Aug. | 3^E^±1.1 | 3^E^±1.1 |
|  | 1-15 Sep. | 6^D^±1.5 | 6^D^±1.5 |
| September | 16-30 Sep. | 7^D^±1.7 | 7^D^±1.7 |
|  | 1-15 Oct. | 11^C^±1.9 | 8^D^±1.7 |
| October | 16-31 Oct. | 12^C^±2.1 | 11^C^±1.9 |
|  | 1-15 Nov. | 21^B^±2.7 | 15^B^±2.1 |
| November | 16-31 Nov. | 26^A^±2.5 | 19^A^±2.3 |

Means bearing identically formatted same letters are not different from each other’s at 5% probability level.

**OFF SEASON**

**Total old bolls/stick**

During off season PBW larvae remained alive in old bolls which present on the cotton sticks, cotton sticks used by farmers as fuel in spring and summer season. Table 4.2.35: and 4.2.38: shows significant results about presence of old bolls on cotton sticks in different cotton stick heaps at Layyah and Muzaffargarh. Table 4.2.36: and 4.2.39 analysis of variance shows significant results regarding presence of PBW larvae in old bolls. Table 4.2.37: and 4.2.40: the analysis of variance showed non-significant results regarding old bolls which had no larvae at Layyah and Muzaffargarh.

Table 4.2.41: and 4.2.42: Means show total old bolls, infested bolls with larvae and infested bolls without larvae in Layyah and Muzaffargarh, in these tables means or average showed that in early months due to low temperature infested bolls with larvae were more than infested bolls without larvae, as temperature rises PBW larvae converts into adult and left bollsduring March. The results are supported by Srinvasa Rao (2004) who described that highest larval incidence of pink bollworm was detected at close to February. On contrast to this Gupta *et al.* (1990) witnessed the peak larval populations from the 2^nd^ week of October to the second week of December.

**Table 4.2.35: ANOVA parameters regarding total old bolls/stick in different sticks heaps at Layyah during off season (Dec-Mar, 2018-19)**

| **SOV** | ***df*** | **SS** | **MS** | **F** | **P** |
| --- | --- | --- | --- | --- | --- |
| Replication | 1 | 0.07220 | 0.07220 |  |  |
| Treatment | 3 | 5.04920 | 1.68307 | 9.32 | 0.049* |
| Error | 3 | 0.54180 | 0.18060 |  |  |
| Total | 7 | 5.66320 |  |  |  |
| Grand Mean 4.2200 | CV 10.07 |  |  |  |  |

Significance level (P< 0.05); *Significant

**Table 4.2.36: ANOVA parameters regarding infested boll with larvae (IBWL)/stick in different sticks heaps at Layyah during off season (Dec-Mar, 2018-19)**

| **SOV** | ***df*** | **SS** | **MS** | **F** | **P** |
| --- | --- | --- | --- | --- | --- |
| Replication | 1 | 0.01125 | 0.01125 |  |  |
| Treatment | 3 | 1.30495 | 0.43498 | 19.97 | 0.017*** |
| Error | 3 | 0.06535 | 0.02178 |  |  |
| Total | 7 | 1.38155 |  |  |  |
| Grand Mean 1.4725 | CV 10.02 |  |  |  |  |

Significance level (P< 0.05); ***Highly significant

**Table 4.2.37: ANOVA parameters regarding infested boll without larvae (IBWtL)/stick in different sticks heap at Layyah during off season (Dec-Mar, 2018-19)**

| **SOV** | ***df*** | **SS** | **MS** | **F** | **P** |
| --- | --- | --- | --- | --- | --- |
| R | 1 | 0.02645 | 0.02645 |  |  |
| T | 3 | 2.34575 | 0.78192 | 7.38 | 0.067^NS^ |
| Error | 3 | 0.31775 | 0.10592 |  |  |
| Total | 7 | 2.68995 |  |  |  |
| Grand Mean 2.2175 | CV 14.68 |  |  |  |  |

Significance level (P< 0.05) NS=Non-significant

T**able 4.2.38: ANOVA parameters regarding total old bolls/stick in different sticks heaps at Muzaffargarh during off season (Dec-Mar, 2018-19)**

| **SOV** | ***df*** | **SS** | **MS** | **F** | **P** |
| --- | --- | --- | --- | --- | --- |
| Replication | 1 | 0.08611 | 0.08611 |  |  |
| Treatment | 3 | 6.44304 | 2.14768 | 21.67 | 0.015* |
| Error | 3 | 0.29734 | 0.09911 |  |  |
| Total | 7 | 6.82649 |  |  |  |
| Grand Mean 4.7813 CV 6.58 |  |  |  |  |  |

Significance level (P< 0.05); ***Highly significant

**Table 4.2.39: ANOVA parameters regarding infested boll with larvae (IBWL)/stick in different sticks heaps at Muzaffargarh during off season (Dec-Mar, 2018-19**

| **SOV** | ***df*** | **SS** | **MS** | **F** | **P** |
| --- | --- | --- | --- | --- | --- |
| Replication | 1 | 0.10125 | 0.10125 |  |  |
| Treatment | 3 | 8.14975 | 2.71658 | 44.35 | 0.005*** |
| Error | 3 | 0.18375 | 0.06125 |  |  |
| Total | 7 | 8.43475 |  |  |  |
| Grand Mean 2.2175 CV 11.16 |  |  |  |  |  |

Significance level (P< 0.05); ***Highly significant

**Table 4.2.40: ANOVA parameters regarding infested boll without larvae (IBWtL)/stick in different sticks heaps at Muzaffargarh during off season (Dec-Mar, 2018-19)**

| **SOV** | ***df*** | **SS** | **MS** | **F** | **P** |
| --- | --- | --- | --- | --- | --- |
| Replication | 1 | 0.04205 | 0.04205 |  |  |
| Treatment | 3 | 1.91095 | 0.63698 | 1.34 | 0.407^NS^ |
| Error | 3 | 1.42615 | 0.47538\ |  |  |
| Total | 7 | 3.37915 |  |  |  |
| Grand Mean 2.1925 CV 31.45 |  |  |  |  |  |

Significance level (<P= 0.05) NS=Non-Significant

**Table 4.2.41: Mean (±SE) of total bolls/stick, IBWL and IBWtL in different cotton heaps at Layyah during off season during off season (Dec-Mar, 2018-19)**

| **Off season** | |  |  | **Layyah** |  |
| --- | --- | --- | --- | --- | --- |
| **Months** | **Days** |  | **Total Bolls/stick** | **IBWL/Stick** | **IBWtL/Stick** |
|  | 1-15 Dec. |  | 5.6^AB^±0.3 | 3.92^A^±0.1 | 1.52^C^±0.2 |
| December | 16-31 Dec. |  | 5.96^A^±0.4 | 3.52^AB^±0.1 | 2.44^B^±0.3 |
|  | 1-15 Jan. |  | 5.56^AB^±0.4 | 2.84^B^±0.2 | 2.72^AB^±0.3 |
| January | 16-31 Jan. |  | 4.84^B^±0.3 | 2.24^BC^±0.1 | 1.32^C^±0.2 |
|  | 1-15 Feb. |  | 4.88^B^±0.3 | 1.36^C^±0.1 | 2.92^AB^±0.3 |
| February | 16-31 Feb. |  | 4.71^B^±0.4 | 1.56^C^±0.1 | 3.12^A^±0.2 |
|  | 1-15 Mar. |  | 3.5^C^±0.2 | 1.2^CD^±0.1 | 1.9^BC^±0.1 |
| March | 16-31 Mar. |  | 3.2^C^±0.3 | 1.1^D^±0.1 | 1.6^BC^±0.1 |

Means bearing identically formatted same letters are not different from each other’s at 5% probability level.

**Table 4.2.42: Mean (±SE) of total bolls/stick, IBWL and IBWtL in different cotton heaps at Muzaffargarh during off season (Dec-Mar, 2018-19)**

| **Infestation on old Bolls/ Off season** | |  | **Muzaffargarh** |  |
| --- | --- | --- | --- | --- |
| **Months** | **Days** | **Total Bolls/stick** | **IBWL** | **IBWtL** |
|  | 1-15 Dec. | 5.6^AB^±0.2 | 3.92^A^±0.1 | 1.52^C^±0.2 |
| December | 16-31 Dec. | 5.96^A^±0.2 | 3.52^AB^±0.1 | 2.44^BC^±0.3 |
|  | 1-15 Jan. | 5.56^AB^±0.2 | 2.84^B^±0.08 | 2.72^B^±0.2 |
| January | 16-31 Jan. | 4.84^B^±0.3 | 2.24^BC^±0.09 | 1.32^C^±0.1 |
|  | 1-15 Feb. | 4.88^B^±0.3 | 1.36^C^±0.08 | 2.92^AB^±0.2 |
| February | 16-31 Feb. | 4.71^B^±0.3 | 1.56^C^±0.09 | 3.12^A^±0.3 |
|  | 1-15 Mar. | 3.5^BC^±0.2 | 1.2^C^±0.09 | 1.9^C^±0.2 |
| March | 16-31 Mar. | 3.2^C^±0.2 | 1.1^C^±0.08 | 1.6^CD^±0.1 |

Means bearing identically formatted same letters are not different from each other’s at 5% probability level.

**Pink Bollworm larvae/old boll**

In old bolls larvae/boll was counted and mostly old infested bolls had PBW larvae. Table 4.2.43: and 4.2.44: shows significant analysis of variance regarding larvae/boll at both Layyah and Muzaffargarh.

In Table 4.2.45: means show that number of larvae was more in December in old bolls at Layyah and Muzaffargarh. Documented that population of PBW remained in left over standingcotton throughout the year (Mallah *et al.* 2000), 35 per cent larvae survived in the lower part of the stalk and the remaining rest on cotton stalks kept horizontally on the floor.

**Table 4.2.43: ANOVA parameters regarding PBW larvae in old bolls of cotton remaining in cotton sticks in different heaps at Layyah during off season (Dec-Mar, 2018-19)**

| **SOV** | ***df*** | **SS** | **MS** | **F** | **P** |
| --- | --- | --- | --- | --- | --- |
| Replication | 1 | 0.11045 | 0.11045 |  |  |
| Treatment | 3 | 0.98895 | 0.32965 | 9.64 | 0.047* |
| Error | 3 | 0.10255 | 0.03418 |  |  |
| Total | 7 | 1.20195 |  |  |  |
| Grand Mean 0.9225 | CV 20.04 |  |  |  |  |

Significance level (P<0.05); *Significant

**Table 4.2.44: ANOVA parameters regarding PBW larvae in old bolls of cotton remaining in cotton sticks in different heaps at Muzaffargarh during off season (Dec- Mar, 2018-19)**

| **SOV** | ***df*** | **SS** | **MS** | **F** | **P** |
| --- | --- | --- | --- | --- | --- |
| Replication | 1 | 0.03645 | 0.03645 |  |  |
| Treatment | 3 | 1.20015 | 0.40005 | 62.02 | 0.003*** |
| Error | 3 | 0.01935 | 0.00645 |  |  |
| Total | 7 | 1.25595 |  |  |  |
| Grand Mean 0.7725 | CV 10.40 |  |  |  |  |

Significance level (P< 0.05); ***Highly significant

**Table 4.2.45: Mean (±SE) comparison table of PBW larvae in old bolls in cotton sticks in heaps at Muzaffargarh and Layyah during of season (Dec-Mar, 2018-19)**

| **Off Season** | | **Larvae/ Boll** | |
| --- | --- | --- | --- |
| **Months** | **Days** | **Layyah** | **Muzaffargarh** |
|  | 1-15 Dec. | 1.6^A^±0.1 | 1.16^A^±0.1 |
| December | 16-31 Dec. | 1.12^B^±0.1 | 1.12^AB^±0.1 |
|  | 1-15 Jan. | 1.16^B^±0.1 | 1.10^B^±0.1 |
| January | 16-31 Jan. | 1^BC^±0.1 | 1^B^±0.1 |
|  | 1-15 Feb. | 0.8^C^±0.09 | 0.8^C^±0.09 |
| February | 16-31 Feb. | 0.9^C^±0.08 | 0.7^C^±0.06 |
|  | 1-15 Mar. | 0.6^D^±0.05 | 0.3^D^±0.04 |
| March | 16-31 Mar. | 0.2^E^±0.02 | 0^E^±0 |

Means bearing identically formatted same letters are not different from each other’s at 5% probability level.

**Instar/larval stage of Pink bollworm in old bolls**

During off season PBW larvae remained alive in old bolls so instar or larval stage in bolls was counted. 1^st^ and 2^nd^ instar were not present in old bolls tables 4.2.46 and 4.2.47 analysis variance show significant results. Mean 3^rd^ instar was present in old bolls, on the other hand 4^th^ instar also present and in tables 4.2.48 and 4.2.49 analysis of variance show significant presence of 4^th^ instar larvae.

Table 4.2.50 and 4.2.451 shows mean or average no. of instar of PBW larvae in old bolls, from both location data, we observed that due to change in climate no. of 3^rd^ and 4^th^ instar larvae were minimum. PBW larvae can also stay alive in damaged seeds but they go through diapause long-lasting from 75 to 315 days, which was conked out when the larvae were exposed to high moisture or after the seeds were sown in field and irrigation done (Beltranand Garcia,1983).

**Table 4.2.46: ANOVA parameters regarding 3^rd^ instar/larval stage of Pink bollworm in old boll at Layyah during off season (Dec-Mar, 2018-19)**

| **SOV** | ***df*** | **SS** | **MS** | **F** | **P** |
| --- | --- | --- | --- | --- | --- |
| Replication | 1 | 0.02645 | 0.02645 |  |  |
| Treatment | 3 | 1.15895 | 0.38632 | 23.87 | 0.013*** |
| Error | 3 | 0.04855 | 0.01618 |  |  |
| Total | 7 | 1.23395 |  |  |  |
| Grand Mean 0.8975 | CV 14.17 |  |  |  |  |

Significance level (P< 0.05); ***Highly significant

**Table 4.2.47: ANOVA parameters regarding 4^th^ instar/larval stage of Pink bollworm in old boll at Layyah during off season (Dec-Mar, 2018-19)**

| **SOV** | ***df*** | **SS** | **MS** | **F** | **P** |
| --- | --- | --- | --- | --- | --- |
| Replication | 1 | 0.13005 | 0.13005 |  |  |
| Treatment | 3 | 6.98945 | 2.32982 | 168.62 | 0.0008*** |
| Error | 3 | 0.04145 | 0.01382 |  |  |
| Total | 7 | 7.16095 |  |  |  |
| Grand Mean 1.9025 | CV 6.18 |  |  |  |  |

Significance level (P< 0.05); ***Highly significant

**Table 4.2.48: ANOVA parameters regarding 3^rd^ instar/larval stage of Pink bollworm in old boll at Muzaffargarh during off season (Dec-Mar, 2018-19)**

| **SOV** | ***df*** | **SS** | **MS** | **F** | **P** |
| --- | --- | --- | --- | --- | --- |
| Replication | 1 | 0.34031 | 0.34031 |  |  |
| Treatment | 3 | 1.11984 | 0.37328 | 4.39 | 0.1280^NS^ |
| Error | 3 | 0.25534 | 0.08511 |  |  |
| Total | 7 | 1.71549 |  |  |  |
| Grand Mean 0.7088 | CV 41.16 |  |  |  |  |

Significance level (P< 0.05); NS=Non-significant

**Table 4.2.49: ANOVA parameters regarding 4^th^ instar/larval stage of Pink bollworm in old boll at Muzaffargarh during off season (Dec-Mar, 2018-19)**

| **SOV** | ***df*** | **SS** | **MS** | **F** | **P** |
| --- | --- | --- | --- | --- | --- |
| R | 1 | 0.32805 | 0.32805 |  |  |
| T | 3 | 8.06545 | 2.68848 | 106.90 | 0.0015*** |
| Error | 3 | 0.07545 | 0.02515 |  |  |
| Total | 7 | 8.46895 |  |  |  |
| Grand Mean 1.8025 | CV 8.80 |  |  |  |  |

Significance level (P< 0.05); ***Highly significant

**Table 4.2.50: Mean (±SE) table regarding instar/larval stage of Pink bollworm in old boll at Layyah during off season (Dec-Mar, 2018-19)**

**Instar**

**OffSeason Layyah**

**Months Days 3rd 4th**

|  | 1-15 Dec. | 1.4^A^±0.1 | 3.29^A^±0.2 |
| --- | --- | --- | --- |
| December | 16-31 Dec. | 1.06^B^±0.1 | 3.1^AB^±0.2 |
|  | 1-15 Jan. | 1.12^B^±0.1 | 2.48^B^±0.3 |
| January | 16-31 Jan. | 1^BC^±0.1 | 2.1^BC^±0.2 |
|  | 1-15 Feb. | 1^BC^±0.1 | 1.45^C^±0.1 |
| February | 16-31 Feb. | 1.1^B^±0.1 | 1.4^C^±0.1 |
|  | 1-15 Mar. | 0.3^C^±0.1 | 0.9^D^±0.1 |
| March | 16-31 Mar. | 0.2^CD^±0.1 | 0.5^E^±0.1 |

Means bearing identically formatted same letters are not different from each other’s at 5% probability level.

**Table 4.2.51: Mean (±SE) table regarding instar/larval stage of Pink bollworm in old boll at Muzaffargarh during off season (Dec-Mar, 2018-19)**

| **Off Season** | | **Instar** | |
| --- | --- | --- | --- |
| **Muzaffargarh** | | | |
| **Months** | **Days** | **3rd** | **4th** |
|  | 1-15 Dec. | 1.14^A^±0.1 | 3.29^A^±0.2 |
| December | 16-31 Dec. | 1.01^AB^±0.1 | 3.1^AB^±0.2 |
|  | 1-15 Jan. | 1.12^A^±0.1 | 2.38^B^±0.2 |
| January | 16-31 Jan. | 1^B^±0.09 | 2.1^BC^±0.1 |
|  | 1-15 Feb. | 1.04^AB^±0.09 | 1.45^C^±0.1 |
| February | 16-31 Feb. | 0^D^±0 | 1^D^±0.09 |
|  | 1-15 Mar. | 0.4^C^±0.02 | 0.9^E^±0.05 |
| March | 16-31 Mar. | 0^D^±0 | 0.2^F^±0.02 |

Means bearing identically formatted same letters are not different from each other’s at 5% probability level.

**Diapausing behavior of Pink bollworm**

During off season PBW larvae rest in diapause and wait for favorable environment, our results shows that during off season many PBW larvae were in diapause condition. Table 4.2.52: and 4.2.54: analysis of variance of NSSDL (No. of single seed diapausing larvae)/plant were significance; it means NSSDL was present during off season. Table 4.2.53: and 4.2.55: analysis of variance shows significant results about NDSDL (No. of double seed diapausing larvae)/plant of PBW larvae in old bolls. Fig. 4.2.3: and 4.2.4: show the typical symptom or behavior of PBW larvae where larvae mostly diapause in double seed.

Present results were similar to Sarwar (2017) who observed that in winter season when cotton is not present in the field then alive larvae go in to old bolls and remained alive in cocoon. In December, in partially open bolls these larvae entered and go to resting stage and these larvae joined two seeds and make their home for rest during off season when March comes temperature goes high these converts into adults and cause damage by producing adults.

Our results also similar to Metcalf and Metcalf (1992) they observed that the hibernating or resting larvae mostly live in double seed. These larvae come out when conditionsweregoodfortheminMarchandAprilbuttheycanremainaliveupto2.5yearsin resting stage. Effective population was builds up in 4 months after moth emergence in cotton. From hibernation population emergence of population was more dangerous.

**Table 4.2.52: ANOVA parameters regarding NSSDL (No. of single seed diapausing lavae/ plant of PBW in cotton at Layyah during off season (Dec-Mar, 2018-19)**

| **Source** | ***df*** | **SS** | **MS** | **F** | **P** |
| --- | --- | --- | --- | --- | --- |
| Replication | 1 | 0.01125 | 0.01125 |  |  |
| Treatment | 3 | 0.90375 | 0.30125 | 130.04 | 0.0011*** |
| Error | 3 | 0.00695 | 0.00232 |  |  |
| Total | 7 | 0.92195 |  |  |  |
| Grand Mean 0.5575 | CV 8.63 |  |  |  |  |

Significance level (<P= 0.05); ***Highly significant

**Table 4.2.53: ANOVA parameters regarding NDSDL (No. of double seed daipausing larvae plant of PBW in cotton crop at Layyah during off season (Dec-Mar, 2018-19)**

| **Source** | ***df*** | **SS** | **MS** | **F** | **P** |
| --- | --- | --- | --- | --- | --- |
| Replication | 1 | 0.50000 | 0.50000 |  |  |
| Treatment | 3 | 7.21540 | 2.40513 | 51.39 | 0.0045*** |
| Error | 3 | 0.14040 | 0.04680 |  |  |
| Total | 7 | 7.85580 |  |  |  |
| Grand Mean 1.5750 | CV 13.74 |  |  |  |  |

Significance level (<P=0.05); ***Highly significant

**Table 4.2.54: ANOVA parameters regarding NSSDL (No. of single seed diapausing larvae)/plant of PBW in cotton at Muzaffargarh during off season (Dec- Mar, 2018-19)**

| **Source** | ***df*** | **SS** | **MS** | **F** | **P** |
| --- | --- | --- | --- | --- | --- |
| Replication | 1 | 0.00011 | 0.00011 |  |  |
| Treatment | 3 | 0.90974 | 0.30325 | 51.29 | 0.0045*** |
| Error | 3 | 0.01774 | 0.00591 |  |  |
| Total | 7 | 0.92759 |  |  |  |
| Grand Mean 0.5562 | CV 13.82 |  |  |  |  |

Significance level (P< 0.05); ***Highly significant

**Table 4.2.55: ANOVA parameters regarding NDSDL (No. of double seed diapausing larvae/plant of PBW in cotton at Muzaffargarh during off season (Dec- Mar, 2018-19)**

| **Source** | ***df*** | **SS** | **MS** | **F** | **P** |
| --- | --- | --- | --- | --- | --- |
| Replication | 1 | 0.36551 | 0.36551 |  |  |
| Treatment | 3 | 6.31264 | 2.10421 | 141.74 | 0.0010*** |
| Error | 3 | 0.04454 | 0.01485 |  |  |
| Total | 7 | 6.72269 |  |  |  |
| Grand Mean 1.5113 | CV 8.06 |  |  |  |  |

Significance level (P< 0.05); ***Highly significant

4

**A**

**Diapausing Behaviour**

**NSSDL NDSDL Linear(NDSDL)**

**A**

**AB**

**AB**

**B**

**B**

**A**

**A**

**A**

**AB**

**BC**

**BC**

**BC**

**BC**

**C C**

**1-15**

**Dec.**

**16-31 1-15 Jan. 16-31**

**Dec. Jan.**

**1-15**

**Feb.**

**16-31**

**Feb.**

**1-15**

**Mar.**

**December January February**

**16-31**

**Mar.**

**March**

**3.5**

**3**

**Max. Daipause**

**2.5**

**2**

**1.5**

**1**

**0.5**

**0**

**-0.5**

**Fig. 4.2.3: Comparison between NSSDL (No. of single seed diapausing larvae)/plant and NDSDL (No. of double seed diapausing larvae)/plant of PBW in cotton crop at Layyah during off season**

**4**

**Daipausing Behaviuor of PBW**

**A**

NSSDL

NDSDL Linear(NDSDL)

**A**

**AB**

**AB**

**B**

**A**

**A**

**B**

**A**

**AB**

**BC**

**BC BC**

**C**

**C**

**D**

**1-15 16-31**

**Dec. Dec.**

**1-15**

**Jan.**

**16-31 1-15 16-31 1-15**

**Jan. Feb. Feb. Mar.**

**December**

**January**

**Off Season**

**February**

**16-31**

**Mar.**

**March**

**3.5**

**3**

**2.5**

**Max. Daipause**

**2**

**1.5**

**1**

**0.5**

**0**

**-0.5**

**Fig. 4.2.4: Comparison between NSSDL (No.of single seed diapausing larvae)/plant and NDSDL (No. of double seed diapausing larvae)/plant of PBW in cotton crop at Muzaffargarh during offseason.**

**PBW Larvae in Soil**

In table 4.2.56: analysis of variance showed significance presence of PBW larvae in soil at Layyah, on the other hand at Muzaffargarh its non-significant results in table 4.2.57. Table **4.2.58**: means showed that no. of PBW larvae at Layyah was more in soil as compared to Muzaffargarh.

**Table 4.2.56: ANOVA parameters regarding PBW larvae in soil under sticks heaps of cotton at Layyah during off season (Dec-Mar, 2018-19)**

| **Source** | ***df*** | **SS** | **MS** | **F** | **P** |
| --- | --- | --- | --- | --- | --- |
| Replication | 1 | 0.00661 | 0.00661 |  |  |
| Treatment | 3 | 0.67874 | 0.22625 | 14.31 | 0.0278** |
| Error | 3 | 0.04744 | 0.01581 |  |  |
| Total | 7 | 0.73279 |  |  |  |
| Grand Mean 0.4438 | CV 28.34 |  |  |  |  |

Significance level (P< 0.05); **Significant

**Table 4.2.57: ANOVA parameters regarding PBW larvae in soil under sticks heaps of cotton at Muzaffargarh during off season (Dec-Mar, 2018-19)**

| **Source** | ***df*** | **SS** | **MS** | **F** | **P** |
| --- | --- | --- | --- | --- | --- |
| Replication | 1 | 0.14045 | 0.14045 |  |  |
| Treatment | 3 | 0.23830 | 0.07943 | 1.61 | 0.3523^NS^ |
| Error | 3 | 0.14785 | 0.04928 |  |  |
| Total | 7 | 0.52660 |  |  |  |
| Grand Mean 0.4700 | CV 47.23 |  |  |  |  |

Significance level (P< 0.05) NS=Non-significant

**Table 4.2.58: Mean (±SE) comparison table regarding PBW larvae in soil under sticks heaps of cotton at Layyah and Muzaffargarh during off season (Dec-Mar, 2018-19)**

| **Off season** | | **Larvae in Soil** | |
| --- | --- | --- | --- |
| **Months** | **Days** | **Layyah** | **Muzaffargarh** |
|  | 1-15 Dec. | 0.96^A^±0.1 | 0.89^A^±0.1 |
| December | 16-31 Dec. | 0.7^B^±0.09 | 0.6^B^±0.09 |
|  | 1-15 Jan. | 0.6^C^±0.08 | 0.5^C^±0.06 |
| January | 16-31 Jan. | 0.56^C^±0.06 | 0.47^D^±0.05 |
|  | 1-15 Feb. | 0.23^E^±0.03 | 0.32^E^±0.05 |
| February | 16-31 Feb. | 0.4^D^±0.05 | 0.28^F^±0.02 |
|  | 1-15 Mar. | 0.1^F^±0.02 | 0.7^G^±0.2 |
| March | 16-31 Mar. | 0^G^±0 | 0^H^±0 |

Means bearing identically formatted same letters are not different from each other’s at 5% probability level.

**Adult male moth population**

During off season PBW adult population was low due to low temperature, in table 4.2.59: analysis of variance showed significance presence of adult male population at Layyah, but in Table 4.2.60: at Muzaffargarh it shown non-significant results. Table 4.2.61: showed mean comparison of adult population at both location, data showed due to increase in temperature PBW adult population was increased.

Jha and Bisen (1994) recorded that seasonal incidence of pink bollworm was largely influenced by the weather factors. However, the results presented, similar to [Ahmad](https://scialert.net/fulltextmobile/?doi=pjbs.2000.2119.2121&1729969_ja) [(1979)](https://scialert.net/fulltextmobile/?doi=pjbs.2000.2119.2121&1729969_ja) who documented the peak moth population in the month of Fe-Mar. and minimum population in month of April. He additional described a small population during May-December. This movement of moth populations is self-contradictory to the current findings that could be due to climatic conditions under which the experiment has been conducted.

**Table 4.2.59: ANOVA parameters regarding Adult male moths captured in traps during off season in Layyah during off season (Dec-Mar, 2018-19)**

| **Source** | ***df*** | **SS** | **MS** | **F** | **P** |
| --- | --- | --- | --- | --- | --- |
| Replication | 1 | 0.3741 | 0.37411 |  |  |
| Treatment | 3 | 29.9317 | 9.97725 | 23.00 | 0.0143*** |
| Error | 3 | 1.3011 | 0.43371 |  |  |
| Total | 7 | 31.6070 |  |  |  |
| Grand Mean 6.7738 | CV 9.72 |  |  |  |  |

Significance level (P< 0.05); ***Highly significant

**Table 4.2.60: ANOVA parameters regarding Adult male moths captured in traps during off season in Muzaffargarh during off season (Dec-Mar, 2018-19)**

| **Source** | ***df*** | **SS** | **MS** | **F** | **P** |
| --- | --- | --- | --- | --- | --- |
| Replication | 1 | 4.3512 | 4.35125 |  |  |
| Treatment | 3 | 8.7737 | 2.92458 | 2.34 | 0.2512^NS^ |
| Error | 3 | 3.7437 | 1.24792 |  |  |
| Total | 7 | 16.8687 |  |  |  |
| Grand Mean 7.2125 | CV 15.49 |  |  |  |  |

Significance level(P<0.05); ^NS^Non-significant

**Table 4.2.61: Mean (±SE) comparison table regarding adult males captured in traps in different cotton heaps at Layyah and Muzaffargarh during off season (Dec-Mar, 2018-19)**

| **Off season** | | **Adult Male Moth captures in Trap** | |
| --- | --- | --- | --- |
| **Months** | **Days** | **Layyah** | **Muzaffargarh** |
|  | 1-15 Dec. | 6.06^BC^±0.4 | 6.9^C^±0.7 |
| December | 16-31 Dec. | 5.2^CD^±0.5 | 6.4^C^±0.6 |
|  | 1-15 Jan. | 5.1^C^±0.6 | 5.4^D^±0.5 |
| January | 16-31 Jan. | 3.6^D^±0.4 | 6.3^C^±0.4 |
|  | 1-15 Feb. | 7.7^B^±0.6 | 6.4^C^±0.6 |
| February | 16-31 Feb. | 7.73^B^±0.5 | 9.1^AB^±1.1 |
|  | 1-15 Mar. | 9.1^AB^±0.6 | 7.2^B^±0.9 |
| March | 16-31 Mar. | 9.7^A^±0.6 | 10^A^±1.2 |

Means bearing identically formatted same letters are not different from each other’s at 5% probability level.

**ACTIVITY 3: EFFECT OF DIFFERENT STRESSES (TEMPERATURE, HUMIDITY, PHOTOPERIOD) ON THE BIOLOGY OF PINK BOLLWORM**

**METHODOLOGY**

**Experiment number 1**

First experiment was done to investigate the effect of temperature on the biology of pink bollworm. Different parameters of pink bollworm biology were studied under different but constant temperature. Five different but constant temperature were selected to check the stress of temperature. Selected temperature were 21°C, 24°C, 27°C, 30°C and 33°C. Reared adults were subjected to each temperature and the longevity of adults was checked under each temperature range. After the longevity the fecundity of females was subjected to these temperature range. Incubation period and hatching % were done in incubator that was set to each different range of temperature separately.

**Experiment number 2**

This experiment was performed to investigate the stress of relative humidity on the development. For this 5 different range of relative humidity were selected such as 40%, 50%, 60%, 70% and 80% RH humidifier (black decker) was used to maintain the different levels of relative humidity the % of RH was monitored with the help of humidity sensor. Each parameter of pink bollworm biology was observed independently under these level of humidity. Data about the stress of very low and high RH (%) was recorded for the statistical analysis.

**Experiment number 3**

To evaluate the stress of photoperiod, the selected insects were exposed to different length of light and darkness. Like the previous two experiments 5 different ranges such as 12:12, 13:11, 14:10, 15:9 and 16:8 L:D of photoperiod were selected to check the stress on different parameters of pink bollworm biology.

**Experiment number 4**

This Experiment was conducted to check the stress of chemical against pink bollworm. Lambda-cyhalothrin commonly known as karate was selected to evaluate the effect of chemical stress on different biological parameters of pink bollworm. 5 Different concentration of lambda-cyhalothrin were prepared such as 0.5ppm, 1ppm, 1.5ppm, 2ppm and 2.5 ppm. Each amount of lambda-cyhalothrin was prepared in 100 ml of water. Solution of chemical was applied to different stages of pink bollworm using a sprayer. Efficacy of applied chemical was observed after 6, 12, 18 and 24 hours. Mortality was checked after every 3, 6 and 12 hours. The changes in the biological parameters were noticed and data was collected to apply statistical analysis.

**RESULTS**

**Effect of temperature on the adult longevity regarding different temperature level under laboratory condition**

Analysis of variance of parameters regarding the adult longevity indicated that different level of temperature had significant effect on the pupal periods of Pink bollworms (F= 79.6; P< 0.0000). It was calculated that the maximum adult longevity with mean value of (14.83) was recorded at 27°C that was followed by (10.16), (10.00), (9.33) and (6.83) for, 23, 30, 21 and 33°C respectively (Table 4.3.2).

**Table 4.3.1: ANOVA parameters and LSD All-Pairwise Comparisons regarding Effect of temperature on the adult longevity**

**ANOVA parameters**

| **SOV** | **DF** | **SS** | **MSS** | **F** | **P** |
| --- | --- | --- | --- | --- | --- |
| Treatments | 4 | 100.767 | 25.1917 | 79.6 | 0.0000 |
| Error | 10 | 3.167 | 0.3167 |  |  |
| Total | 14 | 103.933 |  |  |  |

Grand Mean 10.233 CV 5.50

**LSD All-Pairwise Comparisons for adult longevity on different temperature level**

| **Temperature** | **Mean** | **Homogenous groups** |
| --- | --- | --- |
| 21°C | 9.333 | B |
| 23°C | 10.167 | B |
| 27°C | 14.833 | A |
| 30°C | 10.000 | B |
| 33°C | 6.833 | C |

Values having the same alphabets showing that they are not significant

Alpha 0.05 Standard Error for Comparison 0.4595

Critical T Value 2.228 Critical Value for Comparison 1.0238

**Fecundity of adult female pink bollworm under the effect of different temperature level in laboratory condition**

Analysis of variance of parameters regarding the fecundity indicated that different level of temperature had significant effect on the fecundity of Pink bollworms (F= 16.3; P< 0.0002). It was calculated that the maximum adult longevity with mean value of (29.66) was recorded at 27°C that was followed by (22.00), (21.66), (17.33) and (12.00) for, 23, 30, 21 and 33°C respectively (Table 4.3.2).

**Table: 4.3.2: ANOVA parameters and LSD All-Pairwise Comparisons regarding effect of temperature on fecundity**

**ANOVA parameters**

| **SOV** | **DF** | **SS** | **MSS** | **F** | **P** |
| --- | --- | --- | --- | --- | --- |
| Treatments | 4 | 509.73 | 127.43 | 16.3 | 0.0002 |
| Error | 10 | 78.00 | 7.80 |  |  |
| Total | 14 | 587.73 |  |  |  |

Grand Mean 20.533 CV 13.60

**LSD All-Pairwise Comparisons for fecundity on different temperatures**

| **Treatment** | **Mean** | **Homogenous groups** |
| --- | --- | --- |
| 21°C | 17.333 | B |
| 23°C | 22.000 | B |
| 27°C | 29.667 | A |
| 30°C | 21.667 | B |
| 33°C | 12.000 | C |

The values having the same alphabet indicating that they are not significantly different.

Alpha 0.05 Standard Error for Comparison 2.2804

Critical T Value 2.228 Critical Value for Comparison 5.0809

**Effect on the incubation period of eggs of pink regarding different temperature level under laboratory condition**

Analysis of variance of parameters regarding the incubation indicated that different level of temperature had significant effect on the incubation of Pink bollworms (F= 15.8; P< 0.0003). It was calculated that the maximum incubation with mean value of (8.16) was recorded at 21°C that was followed by (5.16), (4.83), (4.16) and (2.83) for, 30, 23, 33 and 27°C respectively (Table 4.3.3).

**Table: 4.3.3 ANOVA parameters and LSD All-Pairwise Comparisons regarding effect of temperature on the incubation period of eggs**

**ANOVA parameters**

| **SOV** | **DF** | **SS** | **MSS** | **F** | **P** |
| --- | --- | --- | --- | --- | --- |
| Treatments | 4 | 46.4000 | 11.6000 | 15.8 | 0.0003 |
| Error | 10 | 7.3333 | 0.7333 |  |  |
| Total | 14 | 53.7333 |  |  |  |

Grand Mean 5.0333 CV 17.01

**LSD All-Pairwise Comparisons egg-incubation period on different temperature**

| **Treatment** | **Mean** | **Homogenous groups** |
| --- | --- | --- |
| 21°C | 8.1667 | A |
| 23°C | 4.8333 | B |
| 27°C | 2.8333 | C |
| 30°C | 5.1667 | B |
| 33°C | 4.1667 | BC |

The values having the same alphabet indicating that they are not significantly different

Alpha 0.05 Standard Error for Comparison 0.6992

Critical T Value 2.228 Critical Value for Comparison 1.5579

**Hatching % of eggs regarding different temperature level under laboratory condition for pink bollworm**

Analysis of variance of parameters regarding the hatching indicated that different level of temperature had significant effect on the incubation of Pink bollworms (F= 100.0; P< 0.0000). It was calculated that the maximum hatching with mean value of (63.66) was recorded at 27°C that was followed by (51.32), (45.25), (30.92) and (20.36) for, 30, 23, 33 and 21°C respectively (Table 4.3.4).

**Table: 4.3.4: ANOVA parameters and LSD All-Pairwise Comparisons regarding effect of temperature on egg hatching**

**ANOVA parameters**

| **SOV** | **DF** | **SS** | **MSS** | **F** | **P** |
| --- | --- | --- | --- | --- | --- |
| Treatments | 4 | 3471.06 | 867.766 | 100 | 0.0000 |
| Error | 10 | 86.58 | 8.658 |  |  |
| Total | 14 | 3557.64 |  |  |  |

Grand Mean 42.306 CV 6.96

**LSD All-Pairwise Comparisons of egg-hatching on different temperatures**

| **Treatment** | **Mean** | **Homogenous groups** |
| --- | --- | --- |
| 21°C | 20.367 | E |
| 23°C | 30.927 | D |
| 27°C | 63.667 | A |
| 30°C | 51.320 | B |
| 33°C | 45.250 | C |

The values having the same alphabet indicating that they are not significantly different

Alpha 0.05 Standard Error for Comparison 2.4025

Critical T Value 2.228 Critical Value for Comparison 5.3531

**Duration of larval period effected by temperature regarding different temperature level under laboratory condition**

Analysis of variance of parameters regarding the larval period indicated that different level of temperature had significant effect on the larval period of Pink bollworms (F= 22.6; P< 0.0001). It was calculated that the maximum larval period with mean value of (17.50) was recorded at 21°C that was followed by (14.33), (14.16), (13.50) and (10.83) for, 23, 33, 30 and 27°C respectively (Table 4.3.5).

**Table 4.3.5: ANOVA parameters and LSD All-Pairwise Comparisons regarding effect of temperature on larval period**

**ANOVA parameters**

| **SOV** | **DF** | **SS** | **MSS** | **F** | **P** |
| --- | --- | --- | --- | --- | --- |
| Treatments | 4 | 67.9333 | 16.9833 | 22.6 | 0.0001 |
| Error | 10 | 7.5000 | 0.7500 |  |  |
| Total | 14 | 75.43 |  |  |  |

Grand Mean 14.067 CV 6.16

**LSD All-Pairwise Comparisons of larval period on different temperatures**

| **Treatment** | **Mean** | **Homogenous groups** |
| --- | --- | --- |
| 21°C | 17.500 | A |
| 23°C | 14.333 | B |
| 27°C | 10.833 | C |
| 30°C | 13.500 | BC |
| 33°C | 14.167 | B |

The values having the same alphabet indicating that they are not significantly different; Alpha = 0.05

Standard Error for Comparison = 0.7071; Critical T Value = 2.228; Critical Value for Comparison = 1.5755

**Pink bollworm larvae diapausing under the effect of different temperature level under laboratory condition**

Analysis of variance of parameters regarding the larval diapause indicated that different level of temperature had significant effect on the larval period of Pink bollworms (F= 129; P< 0.0000. It was calculated that the maximum larval period with mean value of 43.50 was recorded at 21°C that was followed by 28.99, 21.37, 15.89 and 11.51 for, 23, 30, 33 and 27°C respectively ) (Table 4.3.6).

**Table 4.3.6: ANOVA parameters and LSD All-Pairwise Comparisons regarding effect of temperature on larval diapause**

**ANOVA parameters**

| **SOV** | **DF** | **SS** | **MSS** | **F** | **P** |
| --- | --- | --- | --- | --- | --- |
| Treatments | 4 | 1901.96 | 475.490 | 129 | 0.0000 |
| Error | 10 | 36.78 | 3.678 |  |  |
| Total | 14 | 1938.74 |  |  |  |

Grand Mean 24.259 CV 7.91

**LSD All-Pairwise Comparisons of diapausing larvae on different temperatures**

| **Treatment** | **Mean** | **Homogenous groups** |
| --- | --- | --- |
| 21°C | 43.517 | A |
| 23°C | 28.993 | B |
| 27°C | 11.517 | E |
| 30°C | 21.377 | C |
| 33°C | 15.890 | D |

The values having the same alphabet indicating that they are not significantly different

Alpha 0.05 Standard Error for Comparison 1.5659

Critical T Value 2.228 Critical Value for Comparison 3.4890

**Change in pink bollworm larval weight regarding different temperature level under laboratory condition**

Analysis of variance of parameters regarding the larval weightindicated that different level of temperature has significant effect on the larval weight of Pink bollworms (F= 70.3; P< 0.0000). It was calculated that the maximum larval weight mean with mean value of (19.20) was recorded at 21°C that was followed by (16.26), (11.67), (14.73) and (9.63) for, 23, 30, 33 and 27°C respectively (Table 4.3.7).

**Table 4.3.7: ANOVA parameters and LSD All-Pairwise Comparisons regarding effect of temperature on larval weight**

**ANOVA parameters**

| **SOV** | **DF** | **SS** | **MSS** | **F** | **P** |
| --- | --- | --- | --- | --- | --- |
| Treatments | 4 | 170.106 | 42.5264 | 70.3 | 0.0000 |
| Error | 10 | 6.050 | 0.6050 |  |  |
| Total | 14 | 176.156 |  |  |  |

Grand Mean 14.301 CV 5.44

**LSD All-Pairwise Comparisons of larval weight on different temperatures**

| **Treatment** | **Mean** | **Homogenous groups** |
| --- | --- | --- |
| 21°C | 19.200 | A |
| 23°C | 16.260 | B |
| 27°C | 9.633 | E |
| 30°C | 11.677 | D |
| 33°C | 14.737 | C |

The values having the same alphabet indicating that they are not significantly different

Alpha 0.05 Standard Error for Comparison 0.6351

Critical T Value 2.228 Critical Value for Comparison 1.4151

**Pink bollworm larval mortality effected by different temperature level under laboratory condition**

Analysis of variance of parameters regarding the larval mortality indicated that different level of temperature had significant effect on the larval mortality of Pink bollworms (F= 302; P< 0.0000). It was calculated that the maximum larval weight mean with mean value of (51.10) was recorded at 33°C that was followed by (39.76), (28.79), (21.92) and (16.25) for, 21, 23, 30 and 27°C respectively (Table 4.4.8).

**Table 4.3.8: ANOVA parameters and LSD All-Pairwise Comparisons regarding effect of temperature on larval mortality**

**ANOVA parameters**

| **SOV** | **DF** | **SS** | **MSS** | **F** | **P** |
| --- | --- | --- | --- | --- | --- |
| Treatments | 4 | 2352.07 | 588.018 | 302 | 0.0000 |
| Error | 10 | 19.49 | 1.949 |  |  |
| Total | 14 | 2371.56 |  |  |  |

Grand Mean 31.568 CV 4.42

**LSD All-Pairwise Comparisons of larval mortality on different temperatures**

| **Treatment** | **Mean** | **Homogenous groups** |
| --- | --- | --- |
| 21°C | 39.760 | B |
| 23°C | 28.793 | C |
| 27°C | 16.257 | E |
| 30°C | 21.923 | D |
| 33°C | 51.107 | A |

The values having the same alphabet indicating that they are not significantly different

Alpha 0.05 Standard Error for Comparison 1.1398

Critical T Value 2.228 Critical Value for Comparison 2.5396

**Pupal recovery of pink bollworm regarding different temperature level under laboratory condition**

Analysis of variance of parameters regarding the pupal recovery indicated that different level of temperature had significant effect on the pupal recovery of Pink bollworms (F= 173; P< 0.0000). It was calculated that the maximum pupal recovery mean with mean value of (82.70) was recorded at 27°C that was followed by (52.26), (39.56), (30.65) and (20.91) for, 30, 33, 23 and 21°C respectively (Table 4.3.9).

**Table 4.3.9: ANOVA parameters and LSD All-Pairwise Comparisons regarding effect of temperature on pupal recovery**

**ANOVA parameters**

| **SOV** | **DF** | **SS** | **MSS** | **F** | **P** |
| --- | --- | --- | --- | --- | --- |
| Treatments | 4 | 6867.91 | 1716.98 | 173 | 0.0000 |
| Error | 10 | 99.51 | 9.95 |  |  |
| Total | 14 | 6967.42 |  |  |  |

Grand Mean 45.220 CV 6.98

**LSD All-Pairwise Comparisons of pupal recovery on different temperatures**

| **Treatment** | **Mean** | **Homogenous groups** |
| --- | --- | --- |
| 21°C | 20.917 | E |
| 23°C | 30.657 | D |
| 27°C | 82.703 | A |
| 30°C | 52.260 | B |
| 33°C | 39.563 | C |

The values having the same alphabet indicating that they are not significantly different

Alpha 0.05 Standard Error for Comparison 2.5757

Critical T Value 2.228 Critical Value for Comparison 5.7390

**Duration of the pupal period under different temperature level checked under control laboratory condition**

Analysis of variance of parameters regarding the pupal period indicated that different level of temperature had significant effect on the pupal period of Pink bollworms (F= 71.7; P< 0.0000). It was calculated that the maximum pupal period mean with mean value of (10.0) was recorded at 21°C that was followed by (8.33), (7.50), (6.16) and (4.50) for, 23, 33, 30 and 27°C respectively (Table 4.3.10).

**Table 4.3.10: ANOVA parameters and LSD All-Pairwise Comparisons regarding effect of temperature on pupal period**

**ANOVA parameters**

| **SOV** | **DF** | **SS** | **MSS** | **F** | **P** |
| --- | --- | --- | --- | --- | --- |
| Treatments | 4 | 52.5667 | 13.1417 | 71.7 | 0.0000 |
| Error | 10 | 1.8333 | 0.1833 |  |  |
| Total | 14 | 54.4000 |  |  |  |

Grand Mean 7.3000 CV 5.87

**LSD All-Pairwise Comparisons of pupal periodon different temperature**

| **Treatment** | **Mean** | **Homogenous groups** |
| --- | --- | --- |
| 21°C | 10.000 | A |
| 23°C | 8.333 | B |
| 27°C | 4.500 | E |
| 30°C | 6.167 | D |
| 33°C | 7.500 | C |

The values having the same alphabet indicating that they are not significantly different

Alpha 0.05 Standard Error for Comparison 0.3496

Critical T Value 2.228 Critical Value for Comparison 0.7790

**Pink bollworm pupal weight regarding different temperature level 21, 24, 27, 30 and 33°C under laboratory condition**

Analysis of variance of parameters regarding the pupal weight indicated that different level of temperature had significant effect on the pupal weight of Pink bollworms (F= 28.7; P< 0.0000). It was calculated that the maximum pupal weight mean with mean value of (17.44) was recorded at 27°C that was followed by (13.85), (12.56), (11.56) and (10.50) for, 23, 30, 21 and 33°C respectively (Table 4.3.11).

**Table 4.3.11: ANOVA parameters and LSD All-Pairwise Comparisons regarding effect of temperature on pupal weight**

**ANOVA parameters**

| **SOV** | **DF** | **SS** | **MSS** | **F** | **P** |
| --- | --- | --- | --- | --- | --- |
| Treatments | 4 | 86.2310 | 21.5578 | 28.7 | 0.0000 |
| Error | 10 | 7.5087 | 0.7509 |  |  |
| Total | 14 | 93.7398 |  |  |  |

Grand Mean 13.185 CV 6.57

**LSD All-Pairwise Comparisons of pupal weighton different temperatures**

| **Treatment** | **Mean** | **Homogenous groups** |
| --- | --- | --- |
| 21°C | 11.560 | CD |
| 23°C | 13.850 | B |
| 27°C | 17.440 | A |
| 30°C | 12.567 | BC |
| 33°C | 10.507 | D |

The values having the same alphabet indicating that they are not significantly different

Alpha 0.05 Standard Error for Comparison 0.7075

Critical T Value 2.228 Critical Value for Comparison 1.5764

**Observation of the pupal length against different temperature level when tested in laboratory under control condition**

Analysis of variance of parameters regarding the pupal length indicated that different level of temperature had significant effect on the pupal length of Pink bollworms (F= 35.6; P< 0.0000) (Table 4.3.12). It was calculated that the maximum pupal lengthwith mean value of (7.74) was recorded at 27°C that was followed by (6.29), (6.04), (4.29) and (3.73) for, 30, 33, 23 and 21°C respectively (Table 4.3.12).

**Table 4.3.12: ANOVA parameters and LSD All-Pairwise Comparisons regarding effect of temperature on pupal length**

**ANOVA parameters**

| **SOV** | **DF** | **SS** | **MSS** | **F** | **P** |
| --- | --- | --- | --- | --- | --- |
| Treatments | 4 | 31.3825 | 7.84562 | 35.6 | 0.0000 |
| Error | 10 | 2.2067 | 0.22067 |  |  |
| Total | 14 | 33.5892 |  |  |  |

Grand Mean 5.6200 CV 8.36

**LSD All-Pairwise Comparisons of pupal length on different temperatures**

| **Treatment** | **Mean** | **Homogenous groups** |
| --- | --- | --- |
| 21°C | 3.7300 | C |
| 23°C | 4.2933 | C |
| 27°C | 7.7400 | A |
| 30°C | 6.2967 | B |
| 33°C | 6.0400 | B |

The values having the same alphabet indicating that they are not significantly different

Alpha 0.05 Standard Error for Comparison 0.3836

Critical T Value 2.228 Critical Value for Comparison 0.8546

**Effect of temperature on the pink bollworm pupal mortality under laboratory conditions**

Analysis of variance of parameters regarding the pupal mortality indicated that different level of temperature had significant effect on the pupal mortality of Pink bollworms (F= 230; P< 0.0000) (Table 4.3.13). It was calculated that the maximum pupal mortality with mean value of (59.03) was recorded at 33°C that was followed by (45.44), (29.37), (23.58) and (17.30) for, 21, 23, 30 and 27°C respectively (Table 4.3.13).

**Table 4.3.13: ANOVA parameters and LSD All-Pairwise Comparisons regarding effect of temperature on pupal mortality**

**ANOVA parameters**

| **SOV** | **DF** | **SS** | **MSS** | **F** | **P** |
| --- | --- | --- | --- | --- | --- |
| Treatments | 4 | 3485.26 | 871.314 | 230 | 0.0000 |
| Error | 10 | 37.90 | 3.790 |  |  |
| Total | 14 | 3523.16 |  |  |  |

Grand Mean 34.950 CV 5.57

**LSD All-Pairwise Comparisons of pupal mortality on different temperatures**

| **Treatment** | **Mean** | **Homogenous groups** |
| --- | --- | --- |
| 21°C | 45.443 | B |
| 23°C | 29.377 | C |
| 27°C | 17.307 | E |
| 30°C | 23.587 | D |
| 33°C | 59.037 | A |

The values having the same alphabet indicating that they are not significantly different

Alpha 0.05 Standard Error for Comparison 1.5896

Critical T Value 2.228 Critical Value for Comparison 3.5419

**Effect of different temperature level on the life span of pink bollworm adults under laboratory condition**

Analysis of variance of parameters regarding the adult mortality indicated that different level of temperature had significant effect adult mortality of Pink bollworms (F= 145; P< 0.0000) (Table 4.3.14). It was calculated that the maximum adult mortalitywith mean value of (61.92) was recorded at 33°C that was followed by (47.48), (36.51), (24.99) and (17.92) for, 21, 23, 30 and 27°C respectively (Table 4.3.14).

**Table 4.3.14: ANOVA parameters and LSD All-Pairwise Comparisons regarding effect of temperature on adult mortality**

**ANOVA parameters**

| **SOV** | **DF** | **SS** | **MSS** | **F** | **P** |
| --- | --- | --- | --- | --- | --- |
| Treatments | 4 | 3709.03 | 927.257 | 145 | 0.0000 |
| Error | 10 | 64.16 | 6.416 |  |  |
| Total | 14 | 3773.19 |  |  |  |

Grand Mean 37.766 CV 6.71

**LSD All-Pairwise Comparisons of adult mortality on different temperatures**

| **Treatment** | **Mean** | **Homogenous groups** |
| --- | --- | --- |
| 21°C | 47.480 | B |
| 23°C | 36.517 | C |
| 27°C | 17.920 | E |
| 30°C | 24.993 | D |
| 33°C | 61.920 | A |

The values having the same alphabet indicating that they are not significantly different

Alpha 0.05 Standard Error for Comparison 2.0682

Critical T Value 2.228 Critical Value for Comparison 4.6082

**Adult longevity tested under different level of R: H level under laboratory condition**

Analysis of variance of parameters regarding the adult longevity indicated that different level of RH had significant effect on the adult longevity of Pink bollworms (F= 20.6; P< 0.0000) (Table 4.3.15). It was calculated that the maximum adult longevity with mean value of (13.83) was recorded at 60% RH that was followed by (11.00), (9.33), (8.33) and (6.33) for, 70, 40, 50 and 80% RH respectively (Table 4.3.15).

**Table 4.3.15: ANOVA parameters and LSD All-Pairwise Comparisons regarding effect of different R: H levels on adult longevity**

**ANOVA parameters**

| **SOV** | **DF** | **SS** | **MSS** | **F** | **P** |
| --- | --- | --- | --- | --- | --- |
| Treatments | 4 | 96.267 | 24.0667 | 20.6 | 0.0001 |
| Error | 10 | 11.667 | 1.1667 |  |  |
| Total | 14 | 107.933 |  |  |  |

Grand Mean 9.7667 CV 11.06

**LSD All-Pairwise Comparisons of adult longevity on different R: H levels**

| **R: H %** | **Mean** | **Homogenous groups** |
| --- | --- | --- |
| 40 | 9.333 | BC |
| 50 | 8.333 | C |
| 60 | 13.833 | A |
| 70 | 11.000 | B |
| 80 | 6.333 | D |

Mean values having the same alphabet showing that results are not significant.

Alpha 0.05 Standard Error for Comparison 0.8819

Critical T Value 2.228 Critical Value for Comparison 1.9650

**Effect of five levels of relative humidity on fecundity of pink bollworm level under laboratory condition**

Analysis of variance of parameters regarding the fecundity indicated that different level of RH has significant effect on the fecundity of Pink bollworms (F= 126; P< 0.0000) (Table 4.3.16). It was calculated that the maximum fecundity with mean value of (40.33) was recorded at 60% RH that was followed by (25.00), (21.66), (18.66) and (13.33) for, 70, 50, 40 and 80% RH respectively (Table 4.3.16).

**Table 4.3.16: ANOVA parameters and LSD All-Pairwise Comparisons regarding effect of different relative humidity levels on fecundity**

**ANOVA parameters**

| **SOV** | **DF** | **SS** | **MSS** | **F** | **P** |
| --- | --- | --- | --- | --- | --- |
| Treatments | 4 | 1245.73 | 311.433 | 126 | 0.0000 |
| Error | 10 | 24.67 | 2.467 |  |  |
| Total | 14 | 1270.40 |  |  |  |

Grand Mean 23.800 CV 6.60

**LSD All-Pairwise Comparisons of means**

| **R: H %** | **Mean** | **Homogenous groups** |
| --- | --- | --- |
| 40 | 18.667 | D |
| 50 | 21.667 | C |
| 60 | 40.333 | A |
| 70 | 25.000 | B |
| 80 | 13.333 | E |

Mean values having the same alphabet showing that results are not significant.

Alpha 0.05 Standard Error for Comparison 1.2824

Critical T Value 2.228 Critical Value for Comparison 2.8573

**Effect of different relative humidity levels on eggs-incubation period of pink bollworm (*pectinophora gossypiella*) under laboratory condition**

Analysis of variance of parameters regarding the incubation period of eggs indicated that different level of R: H had significant effect on the incubation period of eggs of Pink bollworms (F= 18.7; P< 0.0001) (Table 4.3.17). It was calculated that the minimum incubation period of eggs with mean value of (2.00) was recorded at 60% R: H that was followed by (3.50), (4.83), (5.33) and (9.16) for, 50, 40, 70 and 80% R: H respectively (Table 4.3.17).

**Table 4.3.17: ANOVA parameters and LSD All-Pairwise Comparisons regarding effect of relative humidity levels on eggs-incubation period**

**ANOVA parameters**

| **SOV** | **DF** | **SS** | **MSS** | **F** | **P** |
| --- | --- | --- | --- | --- | --- |
| Treatments | 4 | 86.2333 | 21.5583 | 18.7 | 0.0001 |
| Error | 10 | 11.5000 | 1.1500 |  |  |
| Total | 14 | 97.7333 |  |  |  |

Grand Mean 4.9667 CV 21.59

**LSD All-Pairwise Comparisons of means**

| **R: H %** | **Mean** | **Homogenous groups** |
| --- | --- | --- |
| 40 | 4.8333 | B |
| 50 | 3.5000 | BC |
| 60 | 2.0000 | C |
| 70 | 5.3333 | B |
| 80 | 9.1667 | A |

Mean values having the same alphabet showing that results are not significant.

Alpha 0.05 Standard Error for Comparison 0.8756

Critical T Value 2.228 Critical Value for Comparison 1.9509

**Egg-hatching ofpink bollworm number on different level of relative humidity R: Hunder laboratory condition**

Analysis of variance of parameters regarding the hatchability of eggs indicated that different level of RH had significant effect on the hatchability of eggs of Pink bollworms (F= 187; P< 0.0000) (Table 4.3.18). It was calculated that the maximum hatchability of eggs with mean value of (73.85) was recorded at 60% RH that was followed by (37.95), (33.18), (21.14) and (14.29) for, 70, 50, 40 and 80% RH respectively (Table 4.3.18).

**Table 4.3.18: ANOVA parameters and LSD All-Pairwise Comparisons regarding effect of relative humidity levels on egg-hatching**

**ANOVA parameters**

| **SOV** | **DF** | **SS** | **MSS** | **F** | **P** |
| --- | --- | --- | --- | --- | --- |
| Treatments | 4 | 6410.29 | 1602.57 | 187 | 0.0000 |
| Error | 10 | 85.71 | 8.57 |  |  |
| Total | 14 | 6496.00 |  |  |  |

Grand Mean 36.084 CV 8.11

**LSD All-Pairwise Comparisons of means**

| **R: H %** | **Mean** | **Homogenous groups** |
| --- | --- | --- |
| 40 | 21.140 | C |
| 50 | 33.180 | B |
| 60 | 73.853 | A |
| 70 | 37.957 | B |
| 80 | 14.290 | D |

Mean values having the same alphabet showing that results are not significant.

Alpha 0.05 Standard Error for Comparison 2.3904

Critical T Value 2.228 Critical Value for Comparison 5.3261

**Pink bollworm larval duration on different relative humidity level (RH) under laboratory condition**

Analysis of variance of parameters regarding the larval period indicated that different level of RH had significant effect on the larval period of Pink bollworms (F= 34.3; P< 0.0000) (Table 4.3.19). It was calculated that the maximum larval period with mean value of (16.83) was recorded at 80% RH that was followed by (14.16), (13.50), (13.16) and (10.50) for, 40, 70, 50 and 60% RH respectively (Table 4.3.19).

**Table 4.3.19: ANOVA parameters and LSD All-Pairwise Comparisons regarding effect of relative humidity levels on larval period**

**ANOVA parameters**

| **SOV** | **DF** | **SS** | **MSS** | **F** | **P** |
| --- | --- | --- | --- | --- | --- |
| Treatments | 4 | 61.7333 | 15.4333 | 34.3 | 0.0000 |
| Error | 10 | 4.5000 | 0.4500 |  |  |
| Total | 14 | 66.2333 |  |  |  |

Grand Mean 13.633 CV 4.92

**LSD All-Pairwise Comparisons of means**

| **R: H %** | **Mean** | **Homogenous groups** |
| --- | --- | --- |
| 40 | 14.167 | D |
| 50 | 13.167 | B |
| 60 | 10.500 | C |
| 70 | 13.500 | B |
| 80 | 16.833 | A |

Mean values having the same alphabet showing that results are not significant.

Alpha 0.05 Standard Error for Comparison 0.5477

Critical T Value 2.228 Critical Value for Comparison 1.2204

**Effect of different levels of Relative humidity (RH) on the life span (days) of *pectinophora gossypiella* adults under laboratory condition**

Analysis of variance of parameters regarding the larval weight indicated that different level of RH had significant effect on the larval weight of Pink bollworms (F= 29.5; P< 0.0000) (Table 4.3.20). It was calculated that the maximum larval weight with mean value of (15.54) was recorded at 80% RH that was followed by (14.11), (12.77), (11.21) and (6.58) for, 40, 70, 50 and 60% RH respectively (Table 4.3.20).

**Table 4.3.20: ANOVA parameters and LSD All-Pairwise Comparisons regarding effect of relative humidity levels on life span of *pectinophora gossypiella* adults**

**ANOVA parameters**

| **SOV** | **DF** | **SS** | **MSS** | **F** | **P** |
| --- | --- | --- | --- | --- | --- |
| Treatments | 4 | 142.942 | 35.7355 | 29.5 | 0.0000 |
| Error | 10 | 12.108 | 1.2108 |  |  |
| Total | 14 | 155.050 |  |  |  |

Grand Mean 12.045 CV 9.14

**LSD All-Pairwise Comparisons of means**

| **R: H %** | **Mean** | **Homogenous groups** |
| --- | --- | --- |
| 40 | 14.117 | AB |
| 50 | 11.210 | C |
| 60 | 6.580 | D |
| 70 | 12.773 | BC |
| 80 | 15.547 | A |

Mean values having the same alphabet showing that results are not significant.

Alpha 0.05 Standard Error for Comparison 0.8985

Critical T Value 2.228 Critical Value for Comparison 2.0019

**Effect of different level of relative humidity (RH) on the pink bollworm larval mortality under laboratory condition**

Analysis of variance of parameters regarding the larval mortality indicated that different level of RH had significant effect on the larval mortality of Pink bollworms (F=597; P< 0.0000) (Table 4.3.21). It was calculated that the maximum larval mortality with mean value of (80.66) was recorded at 80% RH that was followed by (60.22), (50.35), (41.21) and (17.48) for, 40, 50, 70 and 60% RH respectively (Table 4.3.21).

**Table 4.3.21: ANOVA parameters and LSD All-Pairwise Comparisons regarding effect of relative humidity levels on larval mortality**

**ANOVA parameters**

| **SOV** | **DF** | **SS** | **MSS** | **F** | **P** |
| --- | --- | --- | --- | --- | --- |
| Treatments | 4 | 6537.44 | 1634.36 | 597 | 0.0000 |
| Error | 10 | 27.36 | 2.74 |  |  |
| Total | 14 | 6564.80 |  |  |  |

Grand Mean 49.989 CV 3.31

**LSD All-Pairwise Comparisons of means**

| **R: H %** | **Mean** | **Homogenous groups** |
| --- | --- | --- |
| 40 | 60.22 | B |
| 50 | 50.35 | C |
| 60 | 17.48 | E |
| 70 | 41.21 | D |
| 80 | 80.66 | A |

Mean values having the same alphabet showing that results are not significant.

Alpha 0.05 Standard Error for Comparison 1.3506

Critical T Value 2.228 Critical Value for Comparison 3.0093

**Effect of relative humidity on the pupal recovery regarding different RH level under laboratory condition**

Analysis of variance of parameters regarding the pupal recovery indicated that different level of RH had significant effect on the pupal recovery of Pink bollworms (F= 376; P< 0.0000) (Table 4.3.22). It was calculated that the maximum pupal recovery with mean value of (81.10) was recorded at 60% RH that was followed by (67.78), (47.88), (39.68) and (31.65) for, 70, 50, 40 and 80% RH respectively (Table 4.3.22).

**Table 4.3.22: ANOVA parameters and LSD All-Pairwise Comparisons regarding effect of relative humidity levels onpupal recovery**

**ANOVA parameters**

| **SOV** | **DF** | **SS** | **MSS** | **F** | **P** |
| --- | --- | --- | --- | --- | --- |
| Treatments | 4 | 4997.10 | 1249.28 | 376 | 0.0000 |
| Error | 10 | 33.24 | 3.32 |  |  |
| Total | 14 | 5030.34 |  |  |  |

Grand Mean 53.621 CV 3.40

**LSD All-Pairwise Comparisons of means**

| **R: H %** | **Mean** | **Homogenous groups** |
| --- | --- | --- |
| 40 | 39.68 | D |
| 50 | 47.88 | C |
| 60 | 81.10 | A |
| 70 | 67.78 | B |
| 80 | 31.65 | E |

Mean values having the same alphabet showing that results are not significant.

Alpha 0.05 Standard Error for Comparison 1.4885

Critical T Value 2.228 Critical Value for Comparison 3.3167

**Effect of different levels of relative humidity on pupal period during laboratory condition**

Analysis of variance of parameters regarding the pupal period indicated that different level of RH had significant effect on the pupal period of Pink bollworms (F= 22.4; P< 0.0000) (Table 4.3.23). It was calculated that the minimum pupal period with mean value of (4.33) was recorded at 60% RH that was followed by (6.50), (7.16), (8.66) and (9.66) for, 70, 50, 40 and 80% RH respectively (Table 4.3.23).

**Table 4.3.23: ANOVA parameters and LSD All-Pairwise Comparisons regarding effect of relative humidity levels on pupal period**

**ANOVA parameters**

| **SOV** | **DF** | **SS** | **MSS** | **F** | **P** |
| --- | --- | --- | --- | --- | --- |
| Treatments | 4 | 50.7667 | 12.6917 | 22.4 | 0.0001 |
| Error | 10 | 5.6667 | 0.5667 |  |  |
| Total | 14 | 56.4333 |  |  |  |

Grand Mean 7.2667 CV 10.36

**LSD All-Pairwise Comparisons means**

| **R: H %** | **Mean** | **Homogenous groups** |
| --- | --- | --- |
| 40 | 8.6667 | A |
| 50 | 7.1667 | B |
| 60 | 4.3333 | C |
| 70 | 6.5000 | D |
| 80 | 9.6667 | A |

Mean values having the same alphabet showing that results are not significant

Alpha 0.05 Standard Error for Comparison 0.6146

Critical T Value 2.228 Critical Value for Comparison 1.3695

**Impact of different level of relative humidity (RH) on the percentage of diapausing larvae of pink bollworm under laboratory condition**

Analysis of variance of parameters regarding the diapausing larvae indicated that different level of RH had significant effect on the diapausing larvae of Pink bollworms (F= 166; P< 0.0000) (Table 4.3.24). It was calculated that the minimum diapausing larvae with mean value of (10.44) was recorded at 60% RH that was followed by (22.29), (30.45), (41.13) and (66.46) for, 70, 50, 40 and 80% RH respectively (Table 4.3.24).

**Table 4.3.24: ANOVA parameters and LSD All-Pairwise Comparisons regarding effect of relative humidity levels on diapausing larvae**

**ANOVA parameters**

| **SOV** | **DF** | **SS** | **MSS** | **F** | **P** |
| --- | --- | --- | --- | --- | --- |
| Treatments | 4 | 4693.08 | 1173.27 | 166 | 0.0000 |
| Error | 10 | 70.85 | 7.09 |  |  |
| Total | 14 | 4763.93 |  |  |  |

Grand Mean 33.365 CV 7.98

**LSD All-Pairwise Comparisons means**

| **R: H %** | **Mean** | **Homogenous groups** |
| --- | --- | --- |
| 40 | 41.173 | B |
| 50 | 30.450 | C |
| 60 | 10.443 | E |
| 70 | 22.293 | D |
| 80 | 62.467 | A |

Mean values having the same alphabet showing that results are not significant.

Alpha 0.05 Standard Error for Comparison 2.1734

Critical T Value 2.228 Critical Value for Comparison 4.8426

**Effect of different levels of relative humidity (RH) on pupal weight of pink bollworm calculated under laboratory condition**

Analysis of variance of parameters regarding the pupal weight indicated that different level of RH had significant effect on the pupal weight of Pink bollworms (F= 7.51; P< 0.0000) (Table 4.3.25). It was calculated that the maximum pupal weight with mean value of (18.51) was recorded at 60% RH that was followed by (17.22.), (16.51), (15.33) and (15.09) for, 70, 50, 80 and 40% RH respectively (Table 4.3.25).

**Table 4.3.25: ANOVA parameters and LSD All-Pairwise Comparisons regarding effect of relative humidity levels on pupal weight**

**ANOVA parameters**

| **SOV** | **DF** | **SS** | **MSS** | **F** | **P** |
| --- | --- | --- | --- | --- | --- |
| Treatments | 4 | 23.7840 | 5.94599 | 7.51 | 0.0046 |
| Error | 10 | 7.9206 | 0.79206 |  |  |
| Total | 14 | 31.7046 |  |  |  |

Grand Mean 16.535 CV 5.38

**LSD All-Pairwise Comparisons of means**

| **R: H %** | **Mean** | **Homogenous groups** |
| --- | --- | --- |
| 40 | 15.090 | C |
| 50 | 16.517 | BC |
| 60 | 18.513 | A |
| 70 | 17.223 | AB |
| 80 | 15.330 | C |

Mean values having the same alphabet showing that results are not significant.

Alpha 0.05 Standard Error for Comparison 0.5238

Critical T Value 2.228 Critical Value for Comparison 1.1671

**Pink bollworm pupal length under laboratory condition against different levels of relative humidity (RH)**

Analysis of variance of parameters regarding the pupal length indicated that different level of RH had significant effect on the pupal length of Pink bollworms (F= 27.7; P< 0.0000) (Table 4.3.26). It was calculated that the maximum pupal length with mean value of (8.00) was recorded at 60% RH that was followed by (5.58), (5.15), (3.81) and (2.88) for, 70, 50, 40 and 80% RH respectively (Table 4.3.26).

**Table 4.3.26: ANOVA parameters and LSD All-Pairwise Comparisons regarding effect of relative humidity levels on pupal length**

**ANOVA parameters**

| **SOV** | **DF** | **SS** | **MSS** | **F** | **P** |
| --- | --- | --- | --- | --- | --- |
| Treatments | 4 | 45.5647 | 11.3912 | 27.7 | 0.0000 |
| Error | 10 | 4.1154 | 0.4115 |  |  |
| Total | 14 | 49.6801 |  |  |  |

Grand Mean 5.0873 CV 12.61

**LSD All-Pairwise Comparisons means**

| **R: H %** | **Mean** | **Homogenous groups** |
| --- | --- | --- |
| 40 | 3.8167 | C |
| 50 | 5.1533 | B |
| 60 | 8.0000 | A |
| 70 | 5.5800 | B |
| 80 | 2.8867 | C |

Mean values having the same alphabet showing that results are not significant.

Alpha 0.05 Standard Error for Comparison 1.9302

Critical T Value 2.228 Critical Value for Comparison 4.3008

**Effect of different levels of relative humidity on the mortality of pink bollworm pupae under laboratory conditions**

Analysis of variance of parameters regarding the pupal mortality indicated that different level of RH had significant effect on the pupal mortality of Pink bollworms (F= 232; P< 0.0000) (Table 4.3.27). It was calculated that the maximum pupal mortality with mean value of (75.03) was recorded at 80% RH that was followed by (58.99), (47.89), (38.03) and (20.10) for, 40, 50, 70 and 60% RH respectively (Table 4.3.27).

**Table 4.3.27: ANOVA parameters and LSD All-Pairwise Comparisons regarding effect of relative humidity levels on pupal mortality**

**ANOVA parameters**

| **SOV** | **DF** | **SS** | **MSS** | **F** | **P** |
| --- | --- | --- | --- | --- | --- |
| Treatments | 4 | 5185.54 | 1296.38 | 232 | 0.0000 |
| Error | 10 | 55.89 | 5.59 |  |  |
| Total | 14 | 5241.42 |  |  |  |

Grand Mean 48.019 CV 4.92

**LSD All-Pairwise Comparisons of means**

| **R: H %** | **Mean** | **Homogenous groups** |
| --- | --- | --- |
| 40 | 58.997 | B |
| 50 | 47.897 | C |
| 60 | 20.107 | E |
| 70 | 38.063 | D |
| 80 | 75.033 | A |

Mean values having the same alphabet showing that results are not significant.

Alpha 0.05 Standard Error for Comparison 1.3506

Critical T Value 2.228 Critical Value for Comparison 3.0093

**Pink bollworm adult mortality when subjected to five different levels of RH under laboratory condition**

Analysis of variance of parameters regarding the adult mortality indicated that different level of RH had significant effect on the adult mortality of Pink bollworms (F= 55.9; P< 0.0000) (Table 4.3.28). It was calculated that the maximum adult mortality with mean value of (63.09) was recorded at 40% RH that was followed by (55.51), (47.39), (35.17) and (21.44) for, 80, 50, 70 and 60% RH respectively (Table 4.3.28).

**Table 4.3.28: ANOVA parameters and LSD All-Pairwise Comparisons regarding effect of relative humidity levels on adult mortality**

**ANOVA parameters**

| **SOV** | **DF** | **SS** | **MSS** | **F** | **P** |
| --- | --- | --- | --- | --- | --- |
| Treatments | 4 | 3282.37 | 820.594 | 55.9 | 0.0000 |
| Error | 10 | 146.90 | 14.690 |  |  |
| Total | 14 | 3429.27 |  |  |  |

Grand Mean 44.526 CV 8.61

**LSD All-Pairwise Comparisons of means**

| **R: H %** | **Mean** | **Homogenous groups** |
| --- | --- | --- |
| 40 | 63.097 | A |
| 50 | 47.397 | C |
| 60 | 21.443 | E |
| 70 | 35.177 | D |
| 80 | 55.517 | B |

Mean values having the same alphabet showing that results are not significant.

Alpha 0.05 Standard Error for Comparison 3.1294

Critical T Value 2.228 Critical Value for Comparison 6.9728

**Effect of Lambda-cyhalothrin on the adult longevity regarding different concentration under laboratory conditions.**

Analysis of variance of parameters regarding the adult longevity indicated that different concentrations of Lambda-cyhalothrin had significant effect on the adult longevity of Pink bollworms (F= 155; P< 0.0000) (Table 4.3.29). It was calculated that the maximum adult longevity with mean value of (17.33) was recorded at 0.5ppm that was followed by, (14.50), (10.50), (7.00) and (2.33) for, 1, 1.5, 2 and 2.5 ppm respectively (Table 4.3.29).

**Table 4.3.29: ANOVA parameters and LSD All-Pairwise Comparisons regarding the effect of Lambda-cyhalothrin on adult longevity**

**ANOVA parameters**

| **SOV** | **DF** | **SS** | **MSS** | **F** | **P** |
| --- | --- | --- | --- | --- | --- |
| Treatments | 4 | 424.500 | 106.125 | 155 | 0.0000 |
| Error | 10 | 6.833 | 0.683 |  |  |
| Total | 14 | 431.333 |  |  |  |

Grand Mean 10.333 CV 8.00

**LSD All-Pairwise Comparisons Test for adult longevity**

| **Lambda Conc. (ppm)** | **Mean** | **Homogenous groups** |
| --- | --- | --- |
| 0.5 | 17.333 | A |
| 1 | 14.500 | B |
| 1.5 | 10.500 | C |
| 2 | 7.000 | D |
| 2.5 | 2.333 | E |

Values having the same alphabets showing that they are not significant

Alpha 0.05 Standard Error for Comparison 0.6749

Critical T Value 2.228 Critical Value for Comparison 1.5039

**Effect of Lambda-cyhalothrin on the fecundity pink bollworm under laboratory conditions.**

Analysis of variance of parameters regarding the fecundity indicated that different concentrations of Lambda-cyhalothrin had significant effect on the fecundity of Pink bollworms (F= 237; P< 0.0000) (Table 4.3.30). It was calculated that the maximum fecundity with mean value of (24.83) was recorded at 0.5ppm that was followed by (19.33), (13.00), (9.33) and (5.00) for, 1, 1.5, 2 and 2.5ppm respectively (Table 4.3.30).

**Table 4.3.30: ANOVA parameters and LSD All-Pairwise Comparisons regarding the effect of Lambda-cyhalothrin on fecundity**

**ANOVA parameters**

| **SOV** | **DF** | **SS** | **MSS** | **F** | **P** |
| --- | --- | --- | --- | --- | --- |
| Treatments | 4 | 696.400 | 174.100 | 237 | 0.0000 |
| Error | 10 | 7.333 | 0.733 |  |  |
| Total | 14 | 703.733 |  |  |  |

Grand Mean 14.133 CV 6.06

**LSD All-Pairwise Comparisons Test for fecundity**

| **Lambda Conc. (ppm)** | **Mean** | **Homogenous groups** |
| --- | --- | --- |
| 0.5 | 24.000 | A |
| 1 | 19.333 | B |
| 1.5 | 13.000 | C |
| 2 | 9.333 | D |
| 2.5 | 5.000 | E |

Values having the same alphabets showing that they are not significant

Alpha 0.05 Standard Error for Comparison 0.6992

Critical T Value 2.228 Critical Value for Comparison 1.5579

**Effect of Lambda-cyhalothrin on incubation period of eggs under laboratory conditions.**

Analysis of variance of parameters regarding the incubation period indicated that different concentrations of Lambda-cyhalothrin had significant effect on the incubation period of Pink bollworms (F= 11.9; P< 0.0000) (Table 4.3.31). It was calculated that the minimum incubation period with mean value of (4.83) was recorded at 0.5ppm that was followed by (6.00), (6.00), (7.50) and (9.50) for, 1, 1.5, 2 and 2.5ppm respectively (Table 4.3.31).

**Table: 4.3.31: ANOVA parameters and LSD All-Pairwise Comparisons regarding the effect of Lambda-cyhalothrin on incubation period**

**ANOVA parameters**

| **SOV** | **DF** | **SS** | **MSS** | **F** | **P** |
| --- | --- | --- | --- | --- | --- |
| Treatments | 4 | 38.7667 | 9.69167 | 11.9 | 0.0008 |
| Error | 10 | 8.1667 | 0.81667 |  |  |
| Total | 14 | 46.9333 |  |  |  |

Values having the same alphabets showing that they are not significant

Grand Mean 6.7667 CV 13.36

**LSD All-Pairwise Comparisons Test for incubation period**

| **Lambda Conc. (ppm)** | **Mean** | **Homogenous groups** |
| --- | --- | --- |
| 0.5 | 4.83 | C |
| 1 | 6.00 | BC |
| 1.5 | 6.00 | BC |
| 2 | 7.50 | B |
| 2.5 | 9.50 | A |

Alpha 0.05 Standard Error for Comparison 0.7379

Critical T Value 2.228 Critical Value for Comparison 1.6441

**Effect of different concentration of Lambda-cyhalothrin on the hatchability of eggs under laboratory conditions.**

Analysis of variance of parameters regarding the hatchability of eggs indicated that different concentrations of Lambda-cyhalothrin had significant effect on the hatchability of eggs of Pink bollworms (F= 301; P< 0.0000) (Table 4.3.32). It was calculated that the hatchability of eggs with mean value of (68.96) was recorded at 0.5ppm that was followed by (37.95), (30.66), (21.55) and (11.30) for, 1, 1.5, 2 and 2.5 ppm respectively (Table 4.3.32).

**Table: 4.3.32: ANOVA parameters and LSD All-Pairwise Comparisons regarding the effect of Lambda-cyhalothrin on hatching percentage**

**ANOVA parameters**

| **SOV** | **DF** | **SS** | **MSS** | **F** | **P** |
| --- | --- | --- | --- | --- | --- |
| Treatments | 4 | 5757.98 | 1439.49 | 301 | 0.0000 |
| Error | 10 | 47.84 | 4.78 |  |  |
| Total | 14 | 5805.82 |  |  |  |

Grand Mean 34.087 CV 6.42

**LSD All-Pairwise Comparisons Test for hatching %**

| **Lambda Conc. (ppm)** | **Mean** | **Homogenous groups** |
| --- | --- | --- |
| 0.5 | 68.963 | A |
| 1 | 37.957 | B |
| 1.5 | 30.663 | C |
| 2 | 21.50 | D |
| 2.5 | 11.30 | E |

Values having the same alphabets showing that they are not significant

Alpha 0.05 Standard Error for Comparison 1.7858

Critical T Value 2.228 Critical Value for Comparison 3.9791

**Effect of different concentrations of Lambda-cyhalothrin on the duration of larval period (days) when tested under laboratory conditions.**

Analysis of variance of parameters regarding the larval period indicated that different concentrations of Lambda-cyhalothrin had significant effect on the larval period of Pink bollworms (F= 27.9; P< 0.0000) (Table 4.3.33). It was calculated that the maximum larval period with mean value of (16.33) was recorded at 2.5ppm that was followed by (14.83), (13.33), (13.50) and (11.50) for, 2, 1.5, 1 and 0.5 ppm respectively (Table 4.3.33).

**Table 4.3.33: ANOVA parameters and LSD All-Pairwise Comparisons regarding the effect of Lambda-cyhalothrin on larval period**

**ANOVA parameters**

| **SOV** | **DF** | **SS** | **MSS** | **F** | **P** |
| --- | --- | --- | --- | --- | --- |
| Treatments | 4 | 39.1000 | 9.77500 | 27.9 | 0.0000 |
| Error | 10 | 3.5000 | 0.35000 |  |  |
| Total | 14 | 42.6000 |  |  |  |

Grand Mean 13.900 CV 4.26

**LSD All-Pairwise Comparisons Test for period**

| **Lambda Conc. (ppm)** | **Mean** | **Homogenous groups** |
| --- | --- | --- |
| 0.5 | 11.500 | D |
| 1 | 13.500 | C |
| 1.5 | 13.333 | C |
| 2 | 14.833 | B |
| 2.5 | 16.333 | A |

Values having the same alphabets showing that they are not significant

Alpha 0.05 Standard Error for Comparison 0.4830

Critical T Value 2.228 Critical Value for Comparison 1.0763

**Effect of different concentrations of Lambda-cyhalothrin on weight of pink bollworm larvae under the conditions of laboratory.**

Analysis of variance of parameters regarding the larval weight indicated that different concentrations of Lambda-cyhalothrin has significant effect on the larval weight of Pink bollworms (F= 40.5; P< 0.0000) (Table 4.3.34). It was calculated that the minimum larval weight with mean value of (8.05) was recorded at 0.5ppm that was followed by (10.69), (11.41), (12.38) and (17.04) for, 1, 1.5, 2 and 2.5ppm respectively (Table 4.3.34).

**Table 4.3.34: ANOVA parameters and LSD All-Pairwise Comparisons regarding the effect of Lambda-cyhalothrin on larval weight**

**ANOVA parameters**

| **SOV** | **DF** | **SS** | **MSS** | **F** | **P** |
| --- | --- | --- | --- | --- | --- |
| Treatments | 4 | 129.513 | 32.3782 | 40.5 | 0.0000 |
| Error | 10 | 7.999 | 0.7999 |  |  |
| Total | 14 | 137.512 |  |  |  |

Grand Mean 11.917 CV 7.51

**LSD All-Pairwise Comparisons Test for weight**

| **Lambda Conc. (ppm)** | **Mean** | **Homogenous groups** |
| --- | --- | --- |
| 0.5 | 8.053 | D |
| 1 | 10.697 | C |
| 1.5 | 11.410 | BC |
| 2 | 12.383 | B |
| 2.5 | 17.043 | A |

Values having the same alphabets showing that they are not significant

Alpha 0.05 Standard Error for Comparison 0.7303

Critical T Value 2.228 Critical Value for Comparison 1.6271

**Assessment of the larval mortality of pink bollworm under the effect of five different concentration of Lambda-cyhalothrin tested under laboratory conditions.**

Analysis of variance of parameters regarding the larval mortality indicated that different concentrations of Lambda-cyhalothrin had significant effect on the larval mortality of Pink bollworms (F= 301; P< 0.0000) (Table 4.3.35). It was calculated that the maximum larval mortality with mean value of (80.21) was recorded at 2.5ppm that was followed by (63.81), (49.22), (40.51) and (17.48) for, 2, 1, 2 and 0.5 ppm respectively (Table 4.3.35).

**Table 4.3.35: ANOVA parameters and LSD All-Pairwise Comparisons regarding the effect of Lambda-cyhalothrin on larval mortality**

**ANOVA parameters**

| **SOV** | **DF** | **SS** | **MSS** | **F** | **P** |
| --- | --- | --- | --- | --- | --- |
| Treatments | 4 | 6754.02 | 1688.51 | 301 | 0.0000 |
| Error | 10 | 56.01 | 5.60 |  |  |
| Total | 14 | 6810.03 |  |  |  |

Grand Mean 50.252 CV 4.71

**LSD All-Pairwise Comparisons Test for mortality**

| **Lambda Conc. (ppm)** | **Mean** | **Homogenous groups** |
| --- | --- | --- |
| 0.5 | 17.487 | E |
| 1 | 40.513 | D |
| 1.5 | 49.227 | C |
| 2 | 63.817 | B |
| 2.5 | 80.217 | A |

Values having the same alphabets showing that they are not significant

Alpha 0.05 Standard Error for Comparison 1.9323

Critical T Value 2.228 Critical Value for Comparison 4.3054

**Effect of five different concentration of Lambda-cyhalothrin on the pupal recovery when tested under laboratory conditions.**

Analysis of variance of parameters regarding the pupal emergence% indicated that different concentrations of Lambda-cyhalothrin had significant effect on the pupal emergence% of Pink bollworms (F= 194; P< 0.0000) (Table 4.3.36). It was calculated that the maximum pupal emergence% with mean value of (80.73) was recorded at 0.5ppm that was followed by (52.84), (41.92), (31.65) and (24.07) for, 1, 1.5, 2 and 2.5 ppm respectively (Table 4.3.36).

**Table 4.3.36: ANOVA parameters and LSD All-Pairwise Comparisons regarding the effect of Lambda-cyhalothrin on pupal recovery**

**ANOVA parameters**

| **SOV** | **DF** | **SS** | **MSS** | **F** | **P** |
| --- | --- | --- | --- | --- | --- |
| Treatments | 4 | 5868.17 | 1467.04 | 194 | 0.0000 |
| Error | 10 | 75.72 | 7.57 |  |  |
| Total | 14 | 5943.89 |  |  |  |

Grand Mean 46.247 CV 5.95

**LSD All-Pairwise Comparisons Test for pupal recovery**

| **Lambda Conc. (ppm)** | **Mean** | **Homogenous groups** |
| --- | --- | --- |
| 0.5 | 80.733 | A |
| 1 | 52.847 | B |
| 1.5 | 41.923 | C |
| 2 | 31.653 | D |
| 2.5 | 24.077 | E |

Values having the same alphabets showing that they are not significant

Alpha 0.05 Standard Error for Comparison 2.2468

Critical T Value 2.228 Critical Value for Comparison 5.0063

**Effect of Lambda-cyhalothrin on the duration of pupal period (days) of pink bollworm regarding different concentration under laboratory conditions.**

Analysis of variance of parameters regarding the pupal period indicated that different concentrations of Lambda-cyhalothrin had significant effect on the pupal period of Pink bollworms (F= 60.7; P< 0.0000) (Table 4.3.37). It was calculated that the maximum pupal period with mean value of (12.00) was recorded at 2.5ppm that was followed by (9.83), (8.00), (6.50) and (4.50) for, 2, 1.5, 1 and 0.5ppm respectively (Table 4.3.37).

**Table: 4.3.37: ANOVA parameters and LSD All-Pairwise Comparisons regarding the effect of Lambda-cyhalothrin on pupal period**

**ANOVA parameters**

| **SOV** | **DF** | **SS** | **MSS** | **F** | **P** |
| --- | --- | --- | --- | --- | --- |
| Treatments | 4 | 101.167 | 25.2917 | 60.7 | 0.0000 |
| Error | 10 | 4.167 | 0.4167 |  |  |
| Total | 14 | 105.333 |  |  |  |

Grand Mean 10.233 CV 5.50

**LSD All-Pairwise Comparisons Test for pupal period**

| **Lambda Conc. (ppm)** | **Mean** | **Homogenous groups** |
| --- | --- | --- |
| 0.5 | 4.500 | E |
| 1 | 6.500 | D |
| 1.5 | 8.000 | C |
| 2 | 9.833 | B |
| 2.5 | 12.000 | A |

Values having the same alphabets showing that they are not significant

Alpha 0.05 Standard Error for Comparison 0.5270

Critical T Value 2.228 Critical Value for Comparison 1.1743

**Effect of different concentration of Lambda-cyhalothrin on the larval diapausing under laboratory conditions.**

Analysis of variance of parameters regarding the larval diapausing indicated that different concentrations of Lambda-cyhalothrin had significant effect on the larval diapausing of Pink bollworms (F= 67.2; P< 0.0000) (Table 4.3.38). It was calculated that the maximum larval diapausing with mean value of (54.22) was recorded at 2.5ppm that was followed by (35.45), (24.74), (16.73) and (9.03) for, 2, 1.5, 1 and 0.5ppm respectively (Table 4.3.38).

**Table: 4.3.38: ANOVA parameters and LSD All-Pairwise Comparisons regarding the effect of Lambda-cyhalothrin on larval diapausing**

**ANOVA parameters**

| **SOV** | **DF** | **SS** | **MSS** | **F** | **P** |
| --- | --- | --- | --- | --- | --- |
| Treatments | 4 | 3721.40 | 930.350 | 67.2 | 0.0000 |
| Error | 10 | 138.42 | 13.842 |  |  |
| Total | 14 | 3859.82 |  |  |  |

Grand Mean 28.037 CV 13.27

**LSD All-Pairwise Comparisons Test for diapausing larvae**

| **Lambda Conc. (ppm)** | **Mean** | **Homogenous groups** |
| --- | --- | --- |
| 0.5 | 9.030 | E |
| 1 | 16.737 | D |
| 1.5 | 24.747 | C |
| 2 | 35.450 | B |
| 2.5 | 54.223 | A |

Values having the same alphabets showing that they are not significant

Alpha 0.05 Standard Error for Comparison 3.0378

Critical T Value 2.228 Critical Value for Comparison 6.7686

**Influence of different concentration Lambda-cyhalothrin on the pink bollworm pupal weight.**

Analysis of variance of parameters regarding the pupal weight indicated that different concentrations of Lambda-cyhalothrin had significant effect on the pupal weight of Pink bollworms (F= 54.4; P< 0.0000) (Table 4.3.39). It was calculated that the maximum pupal weight with mean value of (15.62) was recorded at 0.5ppm that was followed by (13.21), (9.48), (7.93) and (6.90) for, 1, 1.5, 2 and 2.5ppm respectively (Table 4.3.39).

**Table 4.3.39: ANOVA parameters and LSD All-Pairwise Comparisons regarding the effect of Lambda-cyhalothrin on pupal weight**

**ANOVA parameters**

| **SOV** | **DF** | **SS** | **MSS** | **F** | **P** |
| --- | --- | --- | --- | --- | --- |
| Treatments | 4 | 162.302 | 40.5755 | 54.4 | 0.0000 |
| Error | 10 | 7.461 | 0.7461 |  |  |
| Total | 14 | 169.763 |  |  |  |

Grand Mean 10.633 CV 8.12

**LSD All-Pairwise Comparisons Test for pupal weight**

| **Lambda Conc. (ppm)** | **Mean** | **Homogenous groups** |
| --- | --- | --- |
| 0.5 | 15.627 | A |
| 1 | 13.213 | B |
| 1.5 | 9.487 | C |
| 2 | 7.930 | CD |
| 2.5 | 6.907 | D |

Values having the same alphabets showing that they are not significant

Alpha 0.05 Standard Error for Comparison 0.7053

Critical T Value 2.228 Critical Value for Comparison 1.5714

**Assessment of pupal length of pink bollworm when tested with different concentration of Lambda-cyhalothrin under laboratory conditions.**

Analysis of variance of parameters regarding pupal length indicated that different concentrations of Lambda-cyhalothrin had significant effect on the pupal length of Pink bollworms (F= 21.6; P< 0.0000) (Table 4.3.40). It was calculated that the maximum pupal length with mean value of (6.85) was recorded at 0.5ppm that was followed by (5.33), (4.48), (3.77) and (3.04) for, 1, 1.5, 2 and 2.5ppm respectively (Table 4.3.40).

**Table 4.3.40: ANOVA parameters and LSD All-Pairwise Comparisons regarding the effect of Lambda-cyhalothrin on pupal length**

**ANOVA parameters**

| **SOV** | **DF** | **SS** | **MSS** | **F** | **P** |
| --- | --- | --- | --- | --- | --- |
| Treatments | 4 | 26.0375 | 6.50938 | 21.6 | 0.0001 |
| Error | 10 | 3.0130 | 0.30130 |  |  |
| Total | 14 | 29.0505 |  |  |  |

Grand Mean 4.6967 CV 11.69

**LSD All-Pairwise Comparisons Test for pupal length**

| **Lambda Conc. (ppm)** | **Mean** | **Homogenous groups** |
| --- | --- | --- |
| 0.5 | 6.8533 | A |
| 1 | 5.3300 | B |
| 1.5 | 4.4800 | BC |
| 2 | 3.7767 | CD |
| 2.5 | 3.0433 | D |

Values having the same alphabets showing that they are not significant

Alpha 0.05 Standard Error for Comparison 0.4482

Critical T Value 2.228 Critical Value for Comparison 0.9986

**Evaluation of the pink bollworm pupal mortality against the action of Lambda-cyhalothrin at five different concentration tested under laboratory conditions.**

Analysis of variance of parameters regarding the pupal mortality indicated that different concentrations of Lambda-cyhalothrin had significant effect on the pupal mortality of Pink bollworms (F= 87.9; P< 0.0000) (Table 4.3.41). It was calculated that the maximum pupal mortality with mean value of (64.62) was recorded at 2.5ppm that was followed by (50.63), (44.86), (24.71) and (16.48) for, 2, 1.5, 1 and 0.5ppm respectively (Table 4.3.41).

**Table: 4.3.41: ANOVA parameters and LSD All-Pairwise Comparisons regarding the effect of Lambda-cyhalothrin on pupal mortality**

**ANOVA parameters**

| **SOV** | **DF** | **SS** | **MSS** | **F** | **P** |
| --- | --- | --- | --- | --- | --- |
| Treatments | 4 | 4588.42 | 1147.11 | 87.9 | 0.0000 |
| Error | 10 | 130.43 | 13.04 |  |  |
| Total | 14 | 4718.86 |  |  |  |

Grand Mean 40.263 CV 8.97

**LSD All-Pairwise Comparisons Test for pupal mortality**

| **Lambda Conc. (ppm)** | **Mean** | **Homogenous groups** |
| --- | --- | --- |
| 0.5 | 16.487 | E |
| 1 | 24.710 | D |
| 1.5 | 44.860 | C |
| 2 | 50.633 | B |
| 2.5 | 64.627 | A |

Values having the same alphabets showing that they are not significant

Alpha 0.05 Standard Error for Comparison 2.9488

Critical T Value 2.228 Critical Value for Comparison 6.5704

**Adult mortality under the action of Lambda-cyhalothrin at five different concentrations tested under laboratory conditions.**

Analysis of variance of parameters regarding the adult mortality indicated that different concentrations of Lambda-cyhalothrin had significant effect on the adult mortality of Pink bollworms (F= 117; P< 0.0000) (Table 4.3.42). It was calculated that the maximum adult mortality with mean value of (70.74) was recorded at 2.5ppm that was followed by (59.62), (45.55), (29.63) and (16.77) for, 2, 1.5, 1 and 0.5ppm respectively (Table 4.3.42).

**Table: 4.3.42: ANOVA parameters and LSD All-Pairwise Comparisons regarding the effect of Lambda-cyhalothrin on adult mortality**

**ANOVA parameters**

| **SOV** | **DF** | **SS** | **MSS** | **F** | **P** |
| --- | --- | --- | --- | --- | --- |
| Treatments | 4 | 5724.17 | 1431.04 | 117 | 0.0000 |
| Error | 10 | 122.61 |  |  |  |
| Total | 14 | 5846.78 |  |  |  |

Grand Mean 44.465 CV 7.87

**LSD All-Pairwise Comparisons Test for adult mortality**

| **Lambda Conc. (ppm)** | **Mean** | **Homogenous groups** |
| --- | --- | --- |
| 0.5 | 16.777 | E |
| 1 | 29.630 | D |
| 1.5 | 45.553 | C |
| 2 | 59.623 | B |
| 2.5 | 70.740 | A |

Values having the same alphabets showing that they are not significant

Alpha 0.05 Standard Error for Comparison 2.8590

Critical T Value 2.228 Critical Value for Comparison 6.3702

**Effect of different levels of photoperiod on the adult longevity**

Analysis of variance of parameters regarding the adult longevity indicated that different level of photoperiod had significant effect on the adult longevity of Pink bollworms (F= 32.1; P< 0.0000) (Table 4.3.43). It was calculated that the maximum adult longevity with mean value of (16.50) was recorded at 14:10 (L: D), that was followed by (14.50), (12.50), (9.83) and (7.83) for, 15: 9, 13: 11, 12: 12 and 16:8 (L: D) respectively (Table 4.3.43).

**Table 4.3.43: ANOVA parameters and LSD All-Pairwise Comparisons regarding the effect of different levels of photoperiod on adult longevity**

**ANOVA parameters**

| **SOV** | **DF** | **SS** | **MSS** | **F** | **P** |
| --- | --- | --- | --- | --- | --- |
| Treatments | 4 | 145.600 | 36.4000 | 32.1 | 0.0000 |
| Error | 10 | 11.333 | 1.1333 |  |  |
| Total | 14 | 156.933 |  |  |  |

Grand Mean 12.233 CV 8.70

**LSD All-Pairwise Comparisons Test for adult longevity**

| **Photoperiod (L: D)** | **Mean** | **Homogenous group** |
| --- | --- | --- |
| 12: 12 | 9.833 | D |
| 13: 11 | 12.500 | C |
| 14:10 | 16.500 | A |
| 15:9 | 14.500 | B |
| 16:8 | 7.833 | E |

Values having the same alphabets showing that they are not significant

Alpha 0.05 Standard Error for Comparison 0.8692

Critical T Value 2.228 Critical Value for Comparison 1.9368

**Effect of different levels of photoperiod on fecundity of adult female of pink bollworm**

Analysis of variance of parameters regarding the fecundity indicated that different level of photoperiod had significant effect on the fecundity of Pink bollworms (F= 102; P< 0.0000) (Table 4.3.44). It was calculated that the maximum fecundity with mean value of (28.00) was recorded at 14:10 (L: D), that was followed by (22.00), (20.00), (15.00) and (12.66) for, 15: 9, 13: 11, 12: 12 and 16:8 (L: D) respectively (Table 4.3.44).

**Table: 4.3.44: ANOVA parameters and LSD All-Pairwise Comparisons regarding the effect of different levels of photoperiod on fecundity**

**ANOVA parameters**

| **SOV** | **DF** | **SS** | **MSS** | **F** | **P** |
| --- | --- | --- | --- | --- | --- |
| Treatments | 4 | 437.067 | 109.267 | 102 | 0.0000 |
| Error | 10 | 10.667 | 1.067 |  |  |
| Total | 14 | 447.733 |  |  |  |

Grand Mean 19.533 CV 5.29

**LSD All-Pairwise Comparisons Test forfecundity**

| **Photoperiod (L: D)** | **Mean** | **Homogenous group** |
| --- | --- | --- |
| 12: 12 | 15.000 | D |
| 13: 11 | 20.000 | C |
| 14:10 | 28.000 | A |
| 15:9 | 22.000 | B |
| 16:8 | 12.667 | E |

Values having the same alphabets showing that they are not significant

Alpha 0.05 Standard Error for Comparison 0.8433

Critical T Value 2.228 Critical Value for Comparison 1.8789

**Effect of different levels of photoperiod on incubation of eggs of pink bollworm**

Analysis of variance of parameters regarding the incubation of eggs indicated that different level of photoperiod had significant effect on the incubation period Pink bollworms (F= 10.4; P< 0.0000) (Table 4.3.45). It was calculated that the maximum incubation period with mean value of (6.83) was recorded at 12: 12 (L: D), that was followed by (4.83), (4.66), (4.66) and (2.33) for, 16:8 15: 9, 13: 11 and 14: 10 (L: D) respectively (Table 4.3.45).

**Table 4.3.45: ANOVA parameters and LSD All-Pairwise Comparisons regarding the effect of different levels of photoperiod on incubation period**

**ANOVA parameters**

| **SOV** | **DF** | **SS** | **MSS** | **F** | **P** |
| --- | --- | --- | --- | --- | --- |
| Treatments | 4 | 30.5000 | 7.62500 | 10.4 | 0.0014 |
| Error | 10 | 7.3333 | 0.73333 |  |  |
| Total | 14 | 37.8333 |  |  |  |

Grand Mean 4.6667 CV 18.35

**LSD All-Pairwise Comparisons Test forincubation**

| **Photoperiod (L: D)** | **Mean** | **Homogenous group** |
| --- | --- | --- |
| 12: 12 | 6.8333 | A |
| 13: 11 | 4.6667 | B |
| 14:10 | 2.3333 | C |
| 15:9 | 4.6667 | B |
| 16:8 | 4.8333 | B |

Values having the same alphabets showing that they are not significant

Alpha 0.05 Standard Error for Comparison 0.6992

Critical T Value 2.228 Critical Value for Comparison 1.5579

**Effect of different levels of photoperiod on hatchability% of eggs**

Analysis of variance of parameters regarding the egg-hatching indicated that different level of photoperiod had significant effect on the hatchability% of eggs of Pink bollworms (F= 57.5; P< 0.0000) (Table 4.3.46). It was calculated that the maximum hatchability% of eggs with mean value of (62.62) was recorded at 14:10 (L: D), that was followed by (45.4), (35.22), (31.04) and (19.30) for, 15:9, 16:8 13: 11 and12: 12 (L: D) respectively (Table 4.3.46).

**Table 4.3.46: ANOVA parameters and LSD All-Pairwise Comparisons regarding the effect of different levels of photoperiod on hatching% of eggs**

**ANOVA parameters**

| **SOV** | **DF** | **SS** | **MSS** | **F** | **P** |
| --- | --- | --- | --- | --- | --- |
| Treatments | 4 | 3193.01 | 798.253 | 57.5 | 0.0000 |
| Error | 10 | 138.87 | 13.887 |  |  |
| Total | 14 | 3331.88 |  |  |  |

Grand Mean 38.717 CV 9.62

**LSD All-Pairwise Comparisons Test for hatching% of eggs**

| **Photoperiod (L: D)** | **Mean** | **Homogenous group** |
| --- | --- | --- |
| 12: 12 | 19.300 | D |
| 13: 11 | 31.040 | C |
| 14:10 | 62.623 | A |
| 15:9 | 45.400 | B |
| 16:8 | 35.223 | C |

Values having th e same alphabets showing that they are not significant

Alpha 0.05 Standard Error for Comparison 3.0427

Critical T Value 2.228 Critical Value for Comparison 6.7795

**Effect of different levels of photoperiod on larval period (days)**

Analysis of variance of parameters regarding the larval period indicated that different level of photoperiod had significant effect on the larval period of Pink bollworms (F= 10.7; P< 0.0012) (Table 4.3.47). It was calculated that the maximum larval period with mean value of (15.00) was recorded at 15:09 (L: D), that was followed by (14.83), (14.50), (13.50) and (10.83) for, 12: 12, 16: 8, 13: 11 and 14:10 (L: D) respectively (Table 4.3.47).

**Table 4.3.47: ANOVA parameters and LSD All-Pairwise Comparisons regarding the effect of different levels of photoperiod on larval period**

**ANOVA parameters**

| **SOV** | **DF** | **SS** | **MSS** | **F** | **P** |
| --- | --- | --- | --- | --- | --- |
| Treatments | 4 | 35.6000 | 8.90000 | 10.7 | 0.0012 |
| Error | 10 | 8.3333 | 0.83333 |  |  |
| Total | 14 | 43.9333 |  |  |  |

Grand Mean 13.733 CV 6.65

**LSD All-Pairwise Comparisons Test for larval period**

| **Photoperiod (L: D)** | **Mean** | **Homogenous group** |
| --- | --- | --- |
| 12: 12 | 14.833 | A |
| 13: 11 | 13.500 | C |
| 14:10 | 10.833 | B |
| 15:9 | 15.000 | E |
| 16:8 | 14.500 | D |

Values having the same alphabets showing that they are not significant

Alpha 0.05 Standard Error for Comparison 0.7454

Critical T Value 2.228 Critical Value for Comparison 1.6608

**Effect of different levels of photoperiod on the percentage diapausing larvae of pink bollworm**

Analysis of variance of parameters regarding the diapausing larvae% indicated that different level of photoperiod had significant effect on the diapausing larvae% of Pink bollworms (F= 136; P< 0.0000) (Table 4.3.48). It was calculated that the maximum diapausing larvae (%) with mean value of (29.36) was recorded at 12:12 (L: D), that was followed by (21.23), (17.99), (13.95) and (9.22) for, 13: 11, 16: 8, 15: 9 and 14:10 (L: D) respectively (Table 4.3.48).

**Table 4.3.48: ANOVA parameters and LSD All-Pairwise Comparisons regarding the effect of different levels of photoperiod on diapausing larvae (%)**

**ANOVA parameters**

| **SOV** | **DF** | **SS** | **MSS** | **F** | **P** |
| --- | --- | --- | --- | --- | --- |
| Treatments | 4 | 698.752 | 174.688 | 136 | 0.0000 |
| Error | 10 | 12.891 | 1.289 |  |  |
| Total | 14 | 711.644 |  |  |  |

Grand Mean 18.371 CV 6.18

**LSD All-Pairwise Comparisons Test for diapausing larvae (%)**

| **Photoperiod (L: D)** | **Mean** | **Homogenous group** |
| --- | --- | --- |
| 12: 12 | 29.363 | A |
| 13: 11 | 21.323 | B |
| 14:10 | 9.220 | E |
| 15:9 | 13.957 | D |
| 16:8 | 17.993 | C |

Values having the same alphabets showing that they are not significant

Alpha 0.05 Standard Error for Comparison 0.9270

Critical T Value 2.228 Critical Value for Comparison 2.0656

**Effect of different levels of photoperiod on larvae weight**

Analysis of variance of parameters regarding the larval weight indicated that different level of photoperiod had significant effect on the larval weight of Pink bollworms (F= 92.6; P< 0.0000) (Table 4.3.49). It was calculated that the maximum larval weight with mean value of (19.57) was recorded at 12:12 (L: D), that was followed by (16.63), (15.08), (13.48) and (10.06) for, 13: 11, 16: 8, 15: 9 and 14:10 (L: D) respectively (Table 4.3.49).

**Table 4.3.49: ANOVA parameters and LSD All-Pairwise Comparisons regarding the effect of different levels of photoperiod on larval weight**

**ANOVA parameters**

| **SOV** | **DF** | **SS** | **MSS** | **F** | **P** |
| --- | --- | --- | --- | --- | --- |
| Treatments | 4 | 150.755 | 37.6887 | 92.6 | 0.0000 |
| Error | 10 | 4.070 | 0.4070 |  |  |
| Total | 14 | 154.825 |  |  |  |

Grand Mean 14.963 CV 4.26

**LSD All-Pairwise Comparisons Test forlarval weight**

| **Photoperiod (L: D)** | **Mean** | **Homogenous group** |
| --- | --- | --- |
| 12: 12 | 19.573 | A |
| 13: 11 | 16.630 | B |
| 14:10 | 10.063 | E |
| 15:9 | 13.480 | D |
| 16:8 | 15.070 | C |

Values having the same alphabets showing that they are not significant

Alpha 0.05 Standard Error for Comparison 0.5209

Critical T Value 2.228 Critical Value for Comparison 1.1606

**Effect of different levels of photoperiod on the larval mortality**

Analysis of variance of parameters regarding the larval mortality indicated that different level of photoperiod had significant effect on the larval mortality of Pink bollworms (F= 130; P< 0.0000) (Table 4.3.50). It was calculated that the maximum larval mortality with mean value of (38.84) was recorded at 16:8 (L: D), that was followed by (29.22), (24.29), (21.18) and (17.77) for, 12: 12, 13: 11, 15: 9 and 14:10 (L: D) respectively (Table 4.3.50).

**Table 4.3.50: ANOVA parameters and LSD All-Pairwise Comparisons regarding the effect of different levels of photoperiod on larval mortality**

**ANOVA parameters**

| **SOV** | **DF** | **SS** | **MSS** | **F** | **P** |
| --- | --- | --- | --- | --- | --- |
| Treatments | 4 | 806.186 | 201.547 | 130 | 0.0000 |
| Error | 10 | 15.453 | 1.545 |  |  |
| Total | 14 | 821.640 |  |  |  |

Grand Mean 26.261 CV 4.73

**LSD All-Pairwise Comparisons Test for larval mortality**

| **Photoperiod (L: D)** | **Mean** | **Homogenous group** |
| --- | --- | --- |
| 12: 12 | 29.220 | B |
| 13: 11 | 24.290 | C |
| 14:10 | 17.773 | E |
| 15:9 | 21.180 | D |
| 16:8 | 38.840 | A |

Values having the same alphabets showing that they are not significant

Alpha 0.05 Standard Error for Comparison 1.0150

Critical T Value 2.228 Critical Value for Comparison 2.2616

**Effect of photoperiod on the pupal recovery**

Analysis of variance of parameters regarding the pupal recovery indicated that different level of photoperiod had significant effect on the pupal recovery of Pink bollworms (F= 105; P< 0.0000) (Table 4.3.51). It was calculated that the maximum pupal recovery with mean value of (75.26) was recorded at 14:10 (L: D), that was followed by (51.92), (42.47), (39.07) and (29.37) for, 15: 9, 16: 8, 13: 11 and 12: 12 (L: D) respectively (Table 4.3.51).

**Table 4.3.51: ANOVA parameters and LSD All-Pairwise Comparisons regarding the effect of different levels of photoperiod on pupal recovery**

**ANOVA parameters**

| **SOV** | **DF** | **SS** | **MSS** | **F** | **P** |
| --- | --- | --- | --- | --- | --- |
| Treatments | 4 | 3645.89 | 911.473 | 105 | 0.0000 |
| Error | 10 | 86.64 | 8.664 |  |  |
| Total | 14 | 3732.53 |  |  |  |

Grand Mean 47.621 CV 6.18

**LSD All-Pairwise Comparisons Test forpupal recovery**

| **Photoperiod (L: D)** | **Mean** | **Homogenous group** |
| --- | --- | --- |
| 12: 12 | 29.370 | D |
| 13: 11 | 39.073 | C |
| 14:10 | 75.263 | A |
| 15:9 | 51.927 | B |
| 16:8 | 42.473 | C |

Values having the same alphabets showing that they are not significant

Alpha 0.05 Standard Error for Comparison 2.4034

Critical T Value 2.228 Critical Value for Comparison 5.3550

**Effect of different levels of photoperiod on pupal period (days)**

Analysis of variance of parameters regarding the pupal period indicated that different level of photoperiod had significant effect on the pupal period of Pink bollworms (F= 19.9; P< 0.0001) (Table 4.3.52). It was calculated that the maximum pupal period with mean value of (8.66) was recorded at 12:12 (L: D), that was followed by (7.83), (6.33), (6.33) and (4.83) for, 16: 8, 13: 11, 15: 9 and 14:10 (L: D) respectively (Table 4.3.52).

**Table 4.3.52: ANOVA parameters and LSD All-Pairwise Comparisons regarding the effect of different levels of photoperiod on pupal period**

**ANOVA parameters**

| **SOV** | **DF** | **SS** | **MSS** | **F** | **P** |
| --- | --- | --- | --- | --- | --- |
| Treatments | 4 | 26.5667 | 6.64167 | 19.9 | 0.0001 |
| Error | 10 | 3.3333 | 0.33333 |  |  |
| Total | 14 | 29.9000 |  |  |  |

Grand Mean 6.8000 CV 8.49

**LSD All-Pairwise Comparisons Test forpupal period**

| **Photoperiod (L: D)** | **Mean** | **Homogenous group** |
| --- | --- | --- |
| 12: 12 | 8.6667 | A |
| 13: 11 | 6.3333 | B |
| 14:10 | 4.8333 | C |
| 15:9 | 6.3333 | B |
| 16:8 | 7.8333 | A |

Values having the same alphabets showing that they are not significant

Alpha 0.05 Standard Error for Comparison 0.4714

Critical T Value 2.228 Critical Value for Comparison 1.0504

**Effect of photoperiod on the pupal weight**

Analysis of variance of parameters regarding the pupal weight indicated that different level of photoperiod had significant effect on the pupal weight of Pink bollworms (F= 42.3; P< 0.0000) (Table 4.3.53). It was calculated that the maximum pupal weight with mean value of (18.06) was recorded at 14:10 (L: D), that was followed by (13.74), (12.66), (12.25) and (9.83) for, 13: 11, 15:9 12: 12 and 16:8 (L: D) respectively (Table 4.3.53).

**Table 4.3.53: ANOVA parameters and LSD All-Pairwise Comparisons regarding the effect of different levels of photoperiod on pupal weight**

**ANOVA parameters**

| **SOV** | **DF** | **SS** | **MSS** | **F** | **P** |
| --- | --- | --- | --- | --- | --- |
| Treatments | 4 | 109.224 | 27.3059 | 42.3 | 0.0000 |
| Error | 10 | 6.450 | 0.6450 |  |  |
| Total | 14 | 115.673 |  |  |  |

Grand Mean 13.307 CV 6.03

**LSD All-Pairwise Comparisons Test forpupal weight**

| **Photoperiod (L: D)** | **Mean** | **Homogenous group** |
| --- | --- | --- |
| 12: 12 | 12.257 | C |
| 13: 11 | 13.743 | B |
| 14:10 | 18.067 | A |
| 15:9 | 12.627 | BC |
| 16:8 | 9.843 | D |

Values having the same alphabets showing that they are not significant

Alpha 0.05 Standard Error for Comparison 0.6557

Critical T Value 2.228 Critical Value for Comparison 1.4610

**Effect of different levels of photoperiod on pupae length**

Analysis of variance of parameters regarding the pupal length indicated that different level of photoperiod had significant effect on the pupal length of Pink bollworms (F= 5.94; P< 0.0000) (Table 4.3.54). It was calculated that the maximum pupal length with mean value of (8.41) was recorded at 14:10 (L: D), that was followed by (5.85), (5.68), (5.51) and (3.84) for 13: 11, 15: 9, 16:8 and 12: 12 (L: D) respectively (Table 4.3.54).

**Table 4.3.54: ANOVA parameters and LSD All-Pairwise Comparisons regarding the effect of different levels of photoperiod on pupal length**

**ANOVA parameters**

| **SOV** | **DF** | **SS** | **MSS** | **F** | **P** |
| --- | --- | --- | --- | --- | --- |
| Treatments | 4 | 32.17 | 8.04249 | 5.94 | 0.0103 |
| Error | 10 | 13.5445 | 1.35445 |  |  |
| Total | 14 | 45.7144 |  |  |  |

Grand Mean 5.8620 CV 19.85

**LSD All-Pairwise Comparisons Test for pupal length**

| **Photoperiod (L: D)** | **Mean** | **Homogenous group** |
| --- | --- | --- |
| 12: 12 | 3.8467 | C |
| 13: 11 | 5.8500 | B |
| 14:10 | 8.4133 | A |
| 15:9 | 5.6867 | D |
| 16:8 | 5.5133 | D |

Values having the same alphabets showing that they are not significant

Alpha 0.05 Standard Error for Comparison 0.9502

Critical T Value 2.228 Critical Value for Comparison 2.1173

**Effect of photoperiod on the pupal mortality**

Analysis of variance of parameters regarding the pupal mortality indicated that different level of photoperiod had significant effect on the pupal mortality of Pink bollworms (F= 155; P< 0.0000) (Table 4.3.55). It was calculated that the maximum pupal mortality with mean value of (47.36) was recorded at 16:8 (L: D), that was followed by (37.07), (28.78), (22.63) and (17.74) 12: 12, 13: 11, 15: 9, and 14:10 (L: D) respectively (Table 4.3.55).

**Table 4.3.55: ANOVA parameters and LSD All-Pairwise Comparisons regarding the effect of different levels of photoperiod on pupal mortality**

**ANOVA parameters**

| **SOV** | **DF** | **SS** | **MSS** | **F** | **P** |
| --- | --- | --- | --- | --- | --- |
| Treatments | 4 | 1664.50 | 416.126 | 155 | 0.0000 |
| Error | 10 | 26.85 | 2.685 |  |  |
| Total | 14 | 1691.36 |  |  |  |

Grand Mean 30.719 CV 5.33

**LSD All-Pairwise Comparisons Test for pupal mortality**

| **Photoperiod (L: D)** | **Mean** | **Homogenous group** |
| --- | --- | --- |
| 12: 12 | 37.073 | B |
| 13: 11 | 28.787 | C |
| 14:10 | 17.743 | E |
| 15:9 | 22.630 | D |
| 16:8 | 47.360 | A |

Values having the same alphabets showing that they are not significant

Alpha 0.05 Standard Error for Comparison 1.3380

Critical T Value 2.228 Critical Value for Comparison 2.9813

**Effect of different levels of photoperiod on adult mortality**

Analysis of variance of parameters regarding the adult mortality indicated that different level of photoperiod had significant effect on the adult mortality of Pink bollworms (F= 70.7; P< 0.0000) (Table 4.3.56). It was calculated that the maximum adult mortality with mean value of (48.84) was recorded at 16:8 (L: D), that was followed by (40.02), (35.69), (29.30) and (19.25) for, 12: 12, 13: 11, 15: 9, and 14:10 (L: D) respectively (Table 4.3.56).

**Table 4.3.56: ANOVA parameters and LSD All-Pairwise Comparisons regarding the effect of different levels of photoperiod on** **adult mortality**

**ANOVA parameters**

| **SOV** | **DF** | **SS** | **MSS** | **F** | **P** |
| --- | --- | --- | --- | --- | --- |
| Treatments | 4 | 1490.89 | 372.724 | 70.7 | 0.0000 |
| Error | 10 | 52.69 | 5.269 |  |  |
| Total | 14 | 1543.59 |  |  |  |

Grand Mean 34.623 CV 6.63

**LSD All-Pairwise Comparisons Test for adult mortality**

| **Photoperiod (L: D)** | **Mean** | **Homogenous group** |
| --- | --- | --- |
| 12: 12 | 40.023 | B |
| 13: 11 | 35.697 | C |
| 14:10 | 19.253 | E |
| 15:9 | 29.303 | D |
| 16:8 | 48.840 | A |

Values having the same alphabets showing that they are not significant

Alpha 0.05 Standard Error for Comparison 1.8742

Critical T Value 2.228 Critical Value for Comparison 4.1761

**ACTIVITY-4:** **Determine the Diapausing Behaviour of Pink Bollworm in Field Cage Studies**

**Methodology**

To study the diapausing behavior of PBW, field-cage study was carried out. The field-cages (1 m x 1 m x 1 m) were made of wooden bars and adjusted with cloth on four sides and its lid-surface. The lower surface was made of wooden platter. A three inch thick layer of pulverized sterilized soil was made on lower surface inside the field cage. A fine mesh iron sieve was placed on the soil layer. The soil layer was re-established after having the data on soil-diapausing larvae in previous soil-layer. One hundred infested mature-unopened and opened bolls (50 each) were picked from cotton field on fortnightly basis from August to January (2018-19). During cotton season, unopened-bolls/opened-bolls were collected from plants while after harvesting (after cotton season), unopened-bolls/opened-bolls were picked from the heaves of cotton-sticks. Every time, such collected unopened-bolls were dissected while opened-boll were observed and number of single and double seeds having 4^th^ instar larvae were counted. After counting, the opened and unopened bolls with 4^th^ instar larvae were placed on sieve above soil inside the field-cage. The field cages were kept in the field or near the site of their collection. After ten (10) days, the number of pupated and non-pupated larvae inside bolls/seeds and in soil were counted and larvae not pupating (non-pupated) after ten (10) days were assumed to be in diapause (Lukefahb *et al.,* 1964). Same experiment was repeated during 2019-20 using same methodology.

**RESULTS**

**YEAR 2018-19**

The values of ANOVA parameter explain that Percentage of diapausing-larvae, double-seed diapausing-larvae, single-seed diapausing-larvae, diapausing-larvae in soil, pupated-larvae, double-seed pupating-larvae, single-seed pupating-larvae and pupating-larvae in soil varied significantly during different dates of observation (p < 0.05) (Table 4.4.1). The results revealed that diapausing behavior of the 4^th^ instar larvae of pink bollworm was found inconsistent between its diapausing months. All the 4^th^ instar larvae (100%) underwent pupation (Figure 4.4.1) and no larvae were found in diapausing condition during the months of August and September (Figure 4.4.1). In October-2018, 45.88-48.27% of the total larvae experienced diapause while 100% of the 4^th^ instar larvae demonstrated diapause in the months of November, December and January (Figure 4.4.1). During the month of October, 19.63-21.16%, 16.29-18.48% and 8.64-10.06% of the diapausing larvae were found in diapause in double-seed, single-seed and soil, respectively (Figure 4.4.1). In the month of November, 72.37-73.39%, 12.20-13.25% and 14.38-14.41% of the diapausing larvae experienced diapause in double-seed, single-seed and soil, respectively (Figure 4.4.1). In the month of December 61.99-64.62%, 30.38-32.31% and 5.0-5.69% of the diapausing larvae were found in diapause in double-seed, single-seed and soil, respectively (Figure 4.4.1). Unlikely, during the month of January, more 4^th^ instar larvae were found in diapause in single seed (52-03-63.59%) as compared to those diapausing in double seed (34.58-44.99%). About 1.82-2.97% of diapausing larvae were found in diapause in soil in the month of January. From the month of October to January, the percentage of 4^th^ instar larvae diapausing in double seed decreased while percentage of 4^th^ instar larvae diapausing in single seed increased gradually (Figure 4.4.1). In the months of August and September, all the 4^th^ instar larvae underwent pupation out of which 92.82-94.59% were found pupated in soil and 5.41-7.18% were found pupated in bolls. The percent larvae pupated in bolls were found only in double seed (100%) and none of the larvae were found pupated in single seed (0%) inside the bolls (Figure 4.4.2). In the month of October, 51.73-54.11% larvae were found in pupation (Figure 26) while 45.89-48.27% larvae were found in diapause (Figure 4.4.2). Out of pupating larvae, 79.45-80.18% and 19.82-20.54% larvae pupated in soil and bolls, respectively. Out of the total larvae pupated in bolls, 88.37-90.24% and 9.76-11.63% were found pupated in double and single seed, respectively (Figure 26). In the month November, December and January, no larvae exhibited pupation neither in soil nor in bolls (single or double seed) (Figure 4.4.2).

**Table 4.4.1: ANOVA parameters regarding the diapausing and pupation behavior of 4^th^ instar larvae of pink bollworm during different observation dates during 2018-19.**

| Source of Variation | *df* | Total Diapausing Larvae (%) | | | |  | Double Seeded Diapausing Larvae (%) | | | |
| --- | --- | --- | --- | --- | --- | --- | --- | --- | --- | --- |
|  |  | SS | MS | F | p |  | SS | MS | F | p |
| Dates | 11 | 72843.3 | 6622.118 | 26049.31 | 0 |  | 29535.85 | 2685.077 | 7757.505 | 0 |
| Error | 24 | 6.101153 | 0.254215 |  |  |  | 8.307032 | 0.346126 |  |  |
| Total | 35 | 72849.4 |  |  |  |  | 29544.15 |  |  |  |
| Source of Variation | *df* | Single Seeded Diapausing Larvae (%) | | | |  | Soil Diapausing Larvae (%) | | | |
|  |  | SS | MS | F | p |  | SS | MS | F | p |
| Dates | 11 | 14724.46 | 1338.587 | 3792.449 | 0 |  | 987.8246 | 89.80224 | 546.4981 | 0 |
| Error | 24 | 8.471068 | 0.352961 |  |  |  | 3.943753 | 0.164323 |  |  |
| Total | 35 | 14732.93 |  |  |  |  | 991.7684 |  |  |  |
| Source of Variation | *df* | Total Pupated Larvae (%) | | | |  | Larvae pupated in soil (%) | | | |
|  |  | SS | MS | F | p |  | SS | MS | F | p |
| Dates | 11 | 72843.3 | 6622.118 | 26049.31 | 0 |  | 72348.43 | 6577.13 | 165612.9 | 0 |
| Error | 24 | 6.101153 | 0.254215 |  |  |  | 0.953133 | 0.039714 |  |  |
| Total | 35 | 72849.4 |  |  |  |  | 72349.38 |  |  |  |
| Source of Variation | *df* | Larvae Pupated inside boll (%) | | | |  | Larvae Pupated in Double seed (%) | | | |
|  |  | SS | MS | F | p |  | SS | MS | F | p |
| Dates | 11 | 1849.074 | 168.0976 | 4232.719 | 0 |  | 1491.315 | 135.574 | 4228.928 | 0 |
| Error | 24 | 0.953133 | 0.039714 |  |  |  | 0.769409 | 0.032059 |  |  |
| Total | 35 | 1850.027 |  |  |  |  | 1492.084 |  |  |  |
| Source of Variation | *df* | Larvae Pupated in Single seed (%) | | | | | | | | |
|  |  | SS | | MS | | | F | | p | |
| Dates | 11 | 23.34492 | | 2.122265 | | | 5150.942 | | 0 | |
| Error | 24 | 0.009888 | | 0.000412 | | |  | |  | |
| Total | 35 | 23.35481 | |  | | |  | |  | |

*Df = Degree of freedom; SS = Sum of square; MS = Mean sum of square; F = Calculated F-value; p = Probability value*

**Figure 4.4.1:** Percent total diapausing-larvae, double-seed diapausing-larvae, single-seed diapausing larvae and larvae diapausing in soil observed during various dates of months for the year of 2018-19. (The bars indicate the standard error and mean values having different identical-styled-letters don not different from each other at probability values of 5%. DL = diapausing larvae; DSDL = double-seed diapausing-larvae; SSDL = single-seed diapausing-larvae; SODL = larvae diapausing in soil).

**Figure 4.4.2.** Percent total pupating-larvae, double-seed pupating-larvae, single-seed pupating larvae and larvae pupating in soil observed during various dates of months for the year of 2018-19. (TPL = total pupating-larvae; DSPL = double-seed pupating -larvae; SSPL = single-seed pupating-larvae; PLS = larvae pupating in soil; PLB = Larvae pupating inside bolls).

**YEAR 2019-20**

The values of ANOVA parameter explain that Percentage of diapausing-larvae, double-seed diapausing-larvae, single-seed diapausing-larvae, diapausing-larvae in soil, pupated-larvae, double-seed pupating-larvae, single-seed pupating-larvae and pupating-larvae in soil varied significantly during different dates of observation (p < 0.05) (Table 4.4.2). The results revealed that diapausing behavior of the 4^th^ instar larvae of pink bollworm was found inconsistent between its diapausing months. All the 4^th^ instar larvae (100%) underwent pupation (Figure 4.4.3) and no larvae were found in diapausing condition during the months of August and September (Figure 4.4.3). In October-2019, 47.02-49.41% of the total larvae experienced diapause while 100% of the 4^th^ instar larvae demonstrated diapause in the months of November, December and January (Figure 4.4.3). During the month of October, 20.15-21.77%, 16.65-18.82% and 8.82-10.20% of the diapausing larvae were found in diapause in double-seed, single-seed and soil, respectively (Figure 4.4.3). In the month of November, 72.06-73.09%, 12.43-13.49% and 14.44-14.47% of the diapausing larvae experienced diapause in double-seed, single-seed and soil, respectively (Figure 4.4.3). In the month of December 61.88-64.45%, 30.33-32.23% and 5.21-5.88% of the diapausing larvae were found in diapause in double-seed, single-seed and soil, respectively (Figure 4.4.3). Unlikely, during the month of January, more 4^th^ instar larvae were found in diapause in single seed (51.62-60.21%) as compared to those diapausing in double seed (39.04-45.16%). About 0.92-3.22% of diapausing larvae were found in diapause in soil in the month of January. From the month of October to January, the percentage of 4^th^ instar larvae diapausing in double seed decreased while percentage of 4^th^ instar larvae diapausing in single seed increased gradually (Figure 4.4.3).

In the months of August and September, all the 4^th^ instar larvae underwent pupation out of which 92.95-94.92% were found pupated in soil and 5.08-7.05% were found pupated in bolls. The percent larvae pupated in bolls were found only in double seed (100%) and none of the larvae were found pupated in single seed (0%) inside the bolls (Figure 26). In the month of October, 50.59-52.98% larvae were found in pupation (Figure 26) while 47.02-49.41% larvae were found in diapause (Figure 4.4.4). Out of pupating larvae, 79.38-80.12% and 19.88-20.61% larvae pupated in soil and bolls, respectively. Out of the total larvae pupated in bolls, 88.38-90.25% and 9.75-11.62% were found pupated in double and single seed, respectively (Figure 26). In the month November, December and January, no larvae exhibited pupation neither in soil nor in bolls (single or double seed) (Figure 4.4.4).

**Table 4.4.2: ANOVA parameters regarding the diapausing and pupation behavior of 4^th^ instar larvae of pink bollworm during different observation dates during 2019-20.**

| Source of Variation | *df* | Total Diapausing Larvae (%) | | | |  | Double Seeded Diapausing Larvae (%) | | | |
| --- | --- | --- | --- | --- | --- | --- | --- | --- | --- | --- |
|  |  | SS | MS | F | p |  | SS | MS | F | p |
| Dates | 11 | 72702.9 | 6609.4 | 185890 | 0.00 |  | 29362.04 | 2669.28 | 2283.80 | 0.00 |
| Error | 24 | 0.9 | 0.0 |  |  |  | 28.05 | 1.17 |  |  |
| Total | 35 | 72703.7 |  |  |  |  | 29390.10 |  |  |  |
| Source of Variation | *df* | Single Seeded Diapausing Larvae (%) | | | |  | Soil Diapausing Larvae (%) | | | |
|  |  | SS | MS | F | p |  | SS | MS | F | p |
| Dates | 11 | 13705.38 | 1245.94 | 1262.07 | 0.00 |  | 1021.069 | 92.8244 | 776.452 | 0.00 |
| Error | 24 | 23.69 | 0.99 |  |  |  | 2.869 | 0.1195 |  |  |
| Total | 35 | 13729.07 |  |  |  |  | 1023.938 |  |  |  |
| Source of Variation | *df* | Total Pupated Larvae (%) | | | |  | Larvae pupated in soil (%) | | | |
|  |  | SS | MS | F | p |  | SS | MS | F | p |
| Dates | 11 | 72702.89 | 6609.35 | 185890 | 0.00 |  | 72567.32 | 6597.03 | 138632 | 0.00 |
| Error | 24 | 0.85 | 0.04 |  |  |  | 1.14 | 0.05 |  |  |
| Total | 35 | 72703.74 |  |  |  |  | 72568.46 |  |  |  |
| Source of Variation | *df* | Larvae Pupated inside boll (%) | | | |  | Larvae Pupated in Double seed (%) | | | |
|  |  | SS | MS | F | p |  | SS | MS | F | p |
| Dates | 11 | 1858.396 | 168.945 | 3550.27 | 0.00 |  | 84161.65 | 7651.06 | 7.860828E+15 | 0.00 |
| Error | 24 | 1.142 | 0.048 |  |  |  | 0.00 | 0.00 |  |  |
| Total | 35 | 1859.538 |  |  |  |  | 84161.65 |  |  |  |
| Source of Variation | *df* | Larvae Pupated in Single seed (%) | | | | | | | | |
|  |  | SS | | MS | | | F | | p | |
| Dates | 11 | 576.8501 | | 52.4409 | | | 4.367127E+15 | | 0.00 | |
| Error | 24 | 0.0000 | | 0.0000 | | |  | |  | |
| Total | 35 | 576.8501 | |  | | |  | |  | |

*Df = Degree of freedom; SS = Sum of square; MS = Mean sum of square; F = Calculated F-value; p = Probability value*

**Figure 4.4.3:** Percent total diapausing-larvae, double-seed diapausing-larvae, single-seed diapausing larvae and larvae diapausing in soil observed during various dates of months for the year of 2019-20. DL = diapausing larvae; DSDL = double-seed diapausing-larvae; SSDL = single-seed diapausing-larvae; SODL = larvae diapausing in soil)

**Figure 4.4.4.** Percent total pupating-larvae, double-seed pupating-larvae, single-seed pupating larvae and larvae pupating in soil observed during various dates of months for the year of 2019-20. (TPL = total pupating-larvae; DSPL = double-seed pupating -larvae; SSPL = single-seed pupating-larvae; PLS = larvae pupating in soil; PLB = Larvae pupating inside bolls)

**OBJECTIVE-5:** AUGMENTATION OF BT GENE EFFICACY THROUGH CHEMICAL APPLICATION

- ACTIVITY-1: SCREENING DIFFERENT PGR, PGPRS AND PLANT GROWTH PATHWAYS REGULATORS (CHEMICALS/ PHYTOHORMONES) ON BT COTTON AGAINST PBW

**EFFECT OF PLANT ACTIVATORS ON Bt GENE EXPRESSION**

**5.1.1. MATERIAL AND METHODS**

**Plant source, treatment and insect rearing:**

Seeds of Bt cultivars and non Bt MNH 786 were sown at Learning Research Centre green houses, Post Graduate Agricultural Research Station (PARS) campus, University of Agriculture Faisalabad for taking fresh samples of leaf for expression analysis of *Cry1Ac* and for insect bioassay. The Bt cotton plants were treated with solutions of 0.5 mM jasmonic acid on Seedlings of cotton plants were grown in green house. *Pectinophora gossypiella* insects were collected from cotton fields and then kept in controlled condition. The larvae was fed on artificial diet at 25^0^C ± 2^0^C and 70% ± 5% Relative Humidity, with 14L:10 d hour photoperiod. From each genotype three plants were selected randomly to take samples of flower and bolls for bioassay. A marketable quantification Kit (QuantiPlate^TM^ Kit, EnvroLogix, Inc., Portland, ME) was used to measure the amount of *Cry1Ac* present in the leaves sample

**Expression of *Cry1Ac* by Polymerase Chain Reaction (PCR):**

Total RNA from leaves of non Bt and Bt plants were isolated by the TriZol LS-Reagent® method (Invitrogen- Carlsbad, MI, USA). Amplification of proteinase inhibitorsdefensive gene into the genome of transgenic cotton plants were performed using polymerase chain reaction. Complementary DNA (cDNA) of transgenic cotton plants were used for amplification of proteinase inhibitor gene. Eight μl amplified PCR products of gene were run on 2% agarose gel along with 1kb ladder. All three gels were visualized under UV light and analyzed by 1.5 % agarose gel electrophoresis in 1X TAE buffer.

**5.1.2. RESULTS**

**Effect of JA/MEJA and SA/BTH Treatment on *genes* expression:**
[truncated: 136,461 more chars]
